# Duodenal mucosal mitochondrial gene expression is associated with delayed gastric emptying in diabetic gastroenteropathy

**DOI:** 10.1172/jci.insight.143596

**Published:** 2021-01-25

**Authors:** Susrutha Puthanmadhom Narayanan, Daniel O’Brien, Mayank Sharma, Karl Miller, Peter Adams, João F. Passos, Alfonso Eirin, Tamas Ordog, Adil E. Bharucha

**Affiliations:** 1 Division of Gastroenterology and Hepatology, Department of Medicine, Mayo Clinic, Rochester, Minnesota, USA.; 2 Department of Biomedical Statistics and Informatics, Mayo Clinic, Rochester, Minnesota, USA.; 3 Sanford Burnham Prebys Medical Discovery Institute, San Diego, California, USA.; 4 Department of Physiology and Biomedical Engineering and; 5 Division of Nephrology & Hypertension Research, Department of Medicine, Mayo Clinic, Rochester, Minnesota, USA.

**Keywords:** Endocrinology, Gastroenterology, Diabetes, Mitochondria, Translation

## Abstract

Hindered by a limited understanding of the mechanisms responsible for diabetic gastroenteropathy (DGE), management is symptomatic. We investigated the duodenal mucosal expression of protein-coding genes and microRNAs (miRNA) in DGE and related them to clinical features. The diabetic phenotype, gastric emptying, mRNA, and miRNA expression and ultrastructure of duodenal mucosal biopsies were compared in 39 DGE patients and 21 controls. Among 3175 differentially expressed genes (FDR < 0.05), several mitochondrial DNA–encoded (mtDNA-encoded) genes (12 of 13 protein coding genes involved in oxidative phosphorylation [OXPHOS], both rRNAs and 9 of 22 transfer RNAs) were downregulated; conversely, nuclear DNA–encoded (nDNA-encoded) mitochondrial genes (OXPHOS) were upregulated in DGE. The promoters of differentially expressed genes were enriched in motifs for transcription factors (e.g., NRF1), which regulate mitochondrial biogenesis. Seventeen of 30 differentially expressed miRNAs targeted differentially expressed mitochondrial genes. Mitochondrial density was reduced and correlated with expression of 9 mtDNA OXPHOS genes. Uncovered by principal component (PC) analysis of 70 OXPHOS genes, PC1 was associated with neuropathy (*P* = 0.01) and delayed gastric emptying (*P* < 0.05). In DGE, mtDNA- and nDNA-encoded mitochondrial genes are reduced and increased — associated with reduced mitochondrial density, neuropathy, and delayed gastric emptying — and correlated with cognate miRNAs. These findings suggest that mitochondrial disturbances may contribute to delayed gastric emptying in DGE.

## Graphical abstract

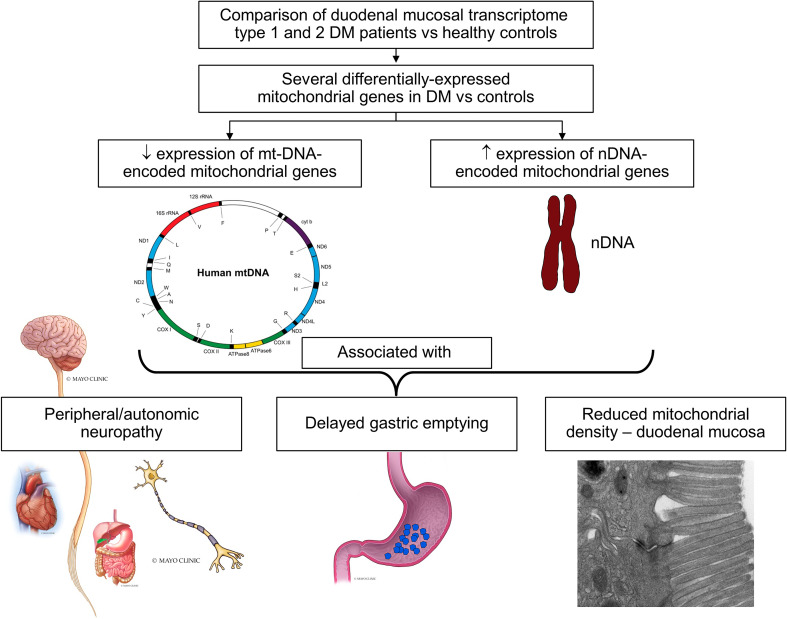

## Introduction

Up to 50% of patients with diabetes mellitus (DM) have delayed gastric emptying (1–3). Diabetic gastroenteropathy (DGE) encompasses dyspepsia (i.e., mild-to-moderate indigestion, with or without accelerated or mildly delayed gastric emptying); gastroparesis, which is characterized by moderate-to-severe upper gastrointestinal symptoms and markedly delayed gastric emptying; and asymptomatic delay in gastric emptying. Building on classical concepts, which emphasized the contributions of hyperglycemia and autonomic dysfunctions, more recent studies have highlighted the role of injury, partly immune-mediated, and dysfunctions in the enteric neuromuscular apparatus (i.e., nerves, interstitial cells of Cajal, and smooth muscle) (3). Dysimmunity and gastric motor disturbances in DGE may reflect epigenetic dysregulation (4). Derived primarily from animal models and, to a limited extent, in patients with DGE, our insight into the mechanisms responsible for neuronal loss and gut dysmotility (e.g., apoptosis, oxidative stress, advanced glycation end products, reduced expression of neuronal nitric oxide signaling, and neuroimmune mechanisms) (5) lags our understanding of the pathogenesis of other DM complications (e.g., neuropathy) (3, 6). Limited by invasiveness, only 1 study compared the transcriptome in full-thickness stomach biopsies between DM patients with delayed gastric emptying and DM controls, who were assumed to have normal gastric emptying (7). In that study, patients with delayed gastric emptying had reduced mRNA expression of several genes related to innate immunity (7). Some DGE patients also have increased chemosensitivity during enteral lipid infusion, which is correlated with the severity of daily symptoms (8). The specific pathogenic mechanisms responsible for DGE are unclear; metabolic, endocrine, and inflammatory changes are probably involved. These disturbances have similar and diverse effects on vital functions (e.g., hepatocyte metabolism, cardiac output, skeletal muscle contraction, insulin production, and neuronal health) in DM (9). More broadly, studies conducted in blood or peripheral mononuclear cells provide compelling evidence that DM has systemic effects on epigenetic regulation, inflammation, and immunity (4, 10, 11). Here, we utilized duodenal mucosal biopsies as surrogates for gastric neuromuscular tissue, which cannot be routinely sampled, to compare the expression of mRNAs and microRNAs (miRNA), which are potent regulators of mRNA stability, between patients with DGE and healthy controls, as well as to investigate the relationship between the transcriptome and clinical features in DGE. All patients with DGE had symptoms of dyspepsia and were categorized, based on the gastric emptying of solids, into DM patients with normal or delayed gastric emptying.

## Results

### Clinical features, gastric emptying, and endoscopy.

The age, sex distribution, and BMI were not significantly different between 24 controls and 40 patients of whom 23 and 17 had type 1 and 2 DM, respectively (Table 1). Similar to the comparison between expression of mitochondrial genes and in vivo parameters (e.g., gastric emptying), the 22 and 5 DM patients with normal and rapid GE, respectively, are combined in Table 1. Of the 24 controls, 18 (75%), 2 (8%), and 4 (17%), respectively, had normal, delayed, and rapid GE. Thirty patients had peripheral neuropathy, 16 had nephropathy, and 16 had retinopathy (12 patients had only peripheral neuropathy, 5 had only autonomic neuropathy, and 13 had both peripheral and autonomic neuropathy). Medications for DM included insulin (32 patients), metformin (11 patients), sulfonylureas (2 patients), thiazolidinediones (1 patient), DPP-IV inhibitors (2 patients), SGLT2 inhibitor (1 patient), and GLP1 receptor agonist (1 patient). None of the participants were taking NSAIDs, systemic corticosteroids, or other immunosuppressive drugs. Fourteen patients were on acid suppressants; 6 were taking nortriptyline or amitriptyline, up to 50 mg daily; and 2 were taking gabapentin, up to 300 mg daily.

Patients predominantly had dyspepsia (*n* = 26), nausea and vomiting (*n* = 10), or abdominal pain or bloating (*n* = 4) (Table 1). The HbA1c, DM duration, and gastrointestinal symptoms were not significantly different between DGE patients with delayed versus nondelayed GE (Table 1).

### Endoscopy.

Among 40 patients and 21 controls who underwent upper endoscopy, 15 patients and 12 controls had normal findings. Other participants had fundic or gastric polyps (7 patients and 1 control), retained food in the stomach (9 patients), experienced gastric or antral erythema or erosions (7 patients and 4 controls), had a gastric ulcer (1 control), and experienced esophagitis (2 patients and 3 controls). However, no participants had endoscopically visible duodenal mucosal injury.

### mRNA expression of genes.

**Figure 1:**
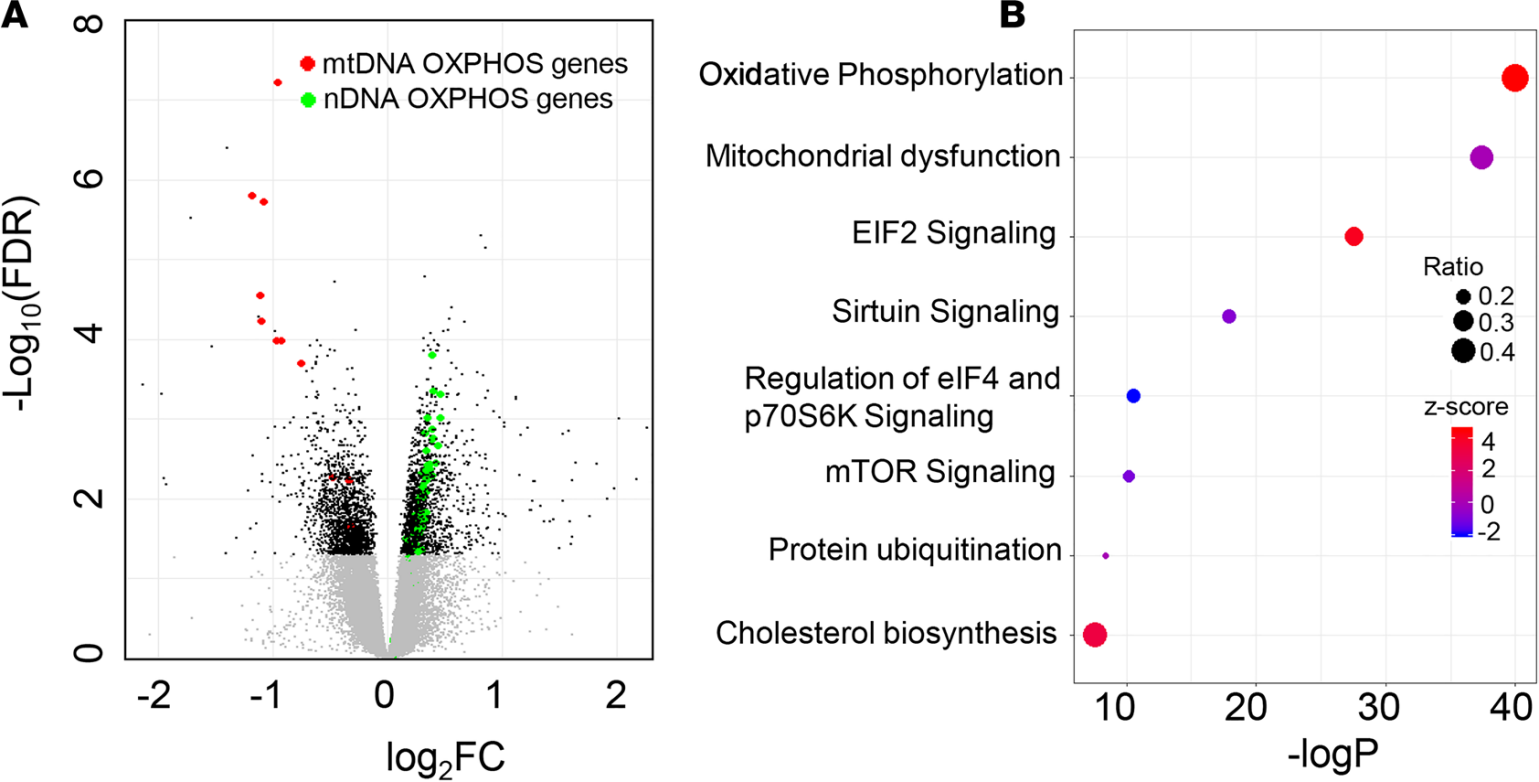
Comparison of duodenal mucosal transcriptome in DGE versus healthy controls. (**A**) Volcano plot of all differentially expressed genes between DGE (*n* = 39) and healthy controls (*n* = 21) analyzed with EdgeR. Gray and black symbols represent genes with FDR ≥ 0.05 and < 0.05, respectively. (**B**) Canonical pathways that are significantly enriched with genes that are differentially expressed between DGE (*n* = 39) and healthy controls (*n* = 21) based on IPA analysis. The bubble size represents the ratio of enrichment, which is the number of genes that belong to the pathway that were differentially expressed in this dataset expressed as a ratio of the total number of genes known to be associated with that pathway. The bubble color represents the *z* score, which is the direction of change in the pathway that is derived from the direction of FC in each gene that was differentially expressed in the pathway. DGE, diabetic gastroenteropathy.

There were 3175 differentially expressed genes (FDR < 0.05) in patients versus controls. Out of these, 1558 and 1617 genes were down- and upregulated, respectively, in DGE (Figure 1A and Supplemental Table 1; supplemental material available online with this article; https://doi.org/10.1172/jci.insight.143596DS1). Among these, 86 genes were differentially expressed with a |log_2_ fold change| (|log_2_FC|) ≥ 1. In the Ingenuity Pathway Analysis (IPA) (https://digitalinsights.qiagen.com) and Kyoto Encyclopedia of Genes and Genomes (KEGG) (https://www.genome.jp/kegg) analysis, the most significant pathways were oxidative phosphorylation (OXPHOS) and mitochondrial dysfunction. The KEGG analysis also identified pathways that pertain to ribosomes and proteasomes. The IPA identified signaling by EIF2, sirtuins, EIF4, p70S6K, mTOR, and protein ubiquitination pathways (Figure 1B).

### Expression of mitochondrial DNA–encoded genes.

**Figure 2:**
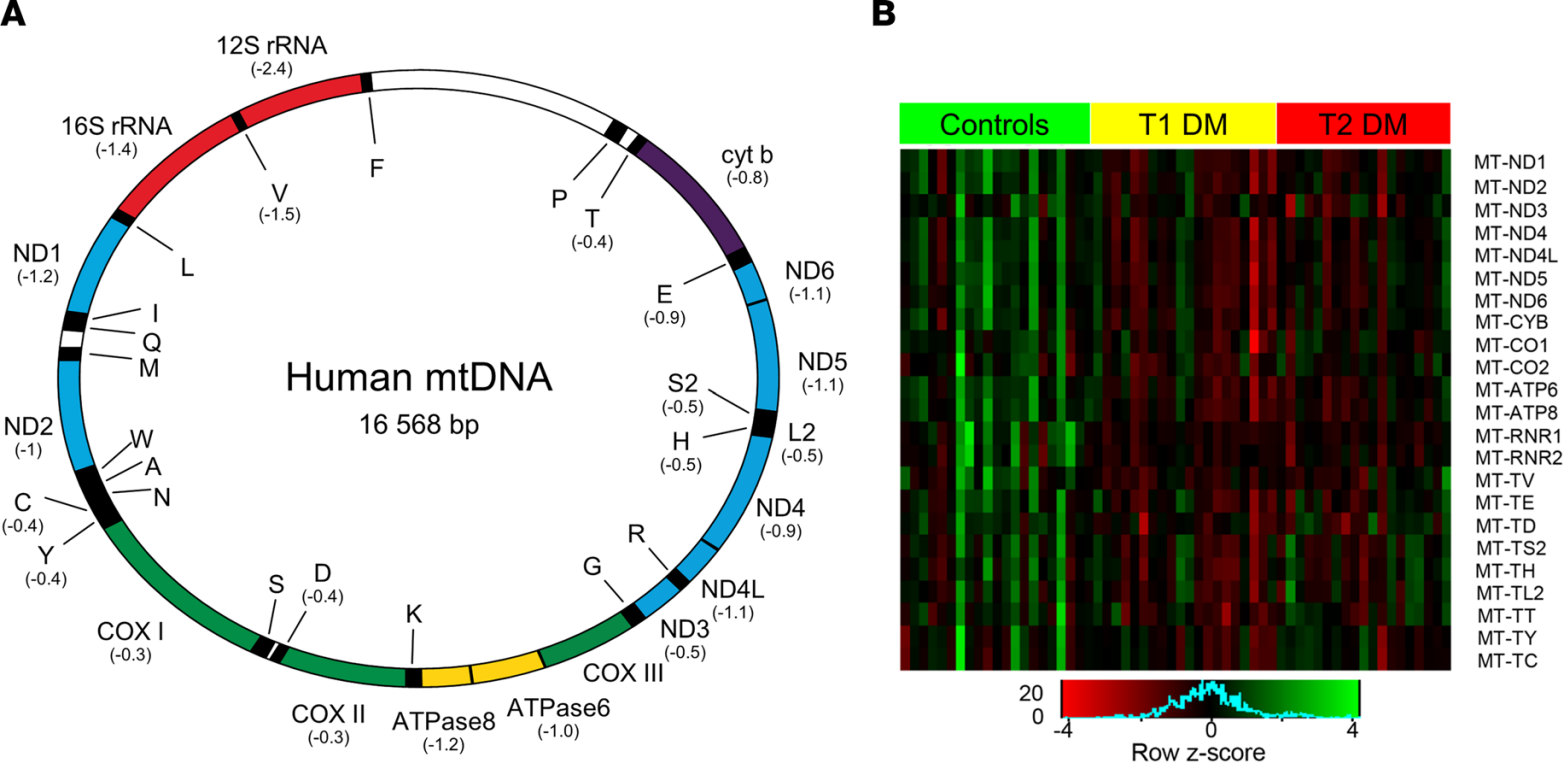
Expression of mitochondrial genes encoded by mitochondrial DNA in DGE (*n* = 39) versus healthy controls (*n* = 21). (**A**) The mitochondrial genome; log_2_FC for individual genes that were significantly differentially expressed are in parentheses. (**B**) A heatmap depicting gene expression (log_RPKM+1_) values for differentially expressed mtDNA genes in patients with DGE (*n* = 39) and healthy controls (*n* = 21). DGE, diabetic gastroenteropathy; RPKM, reads per kilobases per million; mtDNA, mitochondrial DNA.

Compared with controls, the mRNA expression for 12 of 13 mitochondrial DNA–encoded (mtDNA-encoded) protein-coding genes, which are components of complexes I, III, IV, and V of the electron transport chain, both mtDNA-encoded rRNAs, which are components of the mitochondrial ribosome and 9 of 22 mtDNA-encoded transfer RNAs (tRNAs), were all reduced in DGE (FDR < 0.05 for each gene) (Table 2 and Figure 2, A and B). Among these differentially expressed genes, 7 protein-coding genes (*MT-ND1*, *MT-ND2*, *MT-ND4L*, *MT-ND5*, *MT-ND6*, *MT-ATP6*, *MT-ATP8*), both rRNAs and 1 tRNA (*MT-TV*) were differentially expressed with a log_2_FC less than or equal to –1.

### Expression of nDNA genes encoding for mitochondrial proteins.

**Figure 3:**
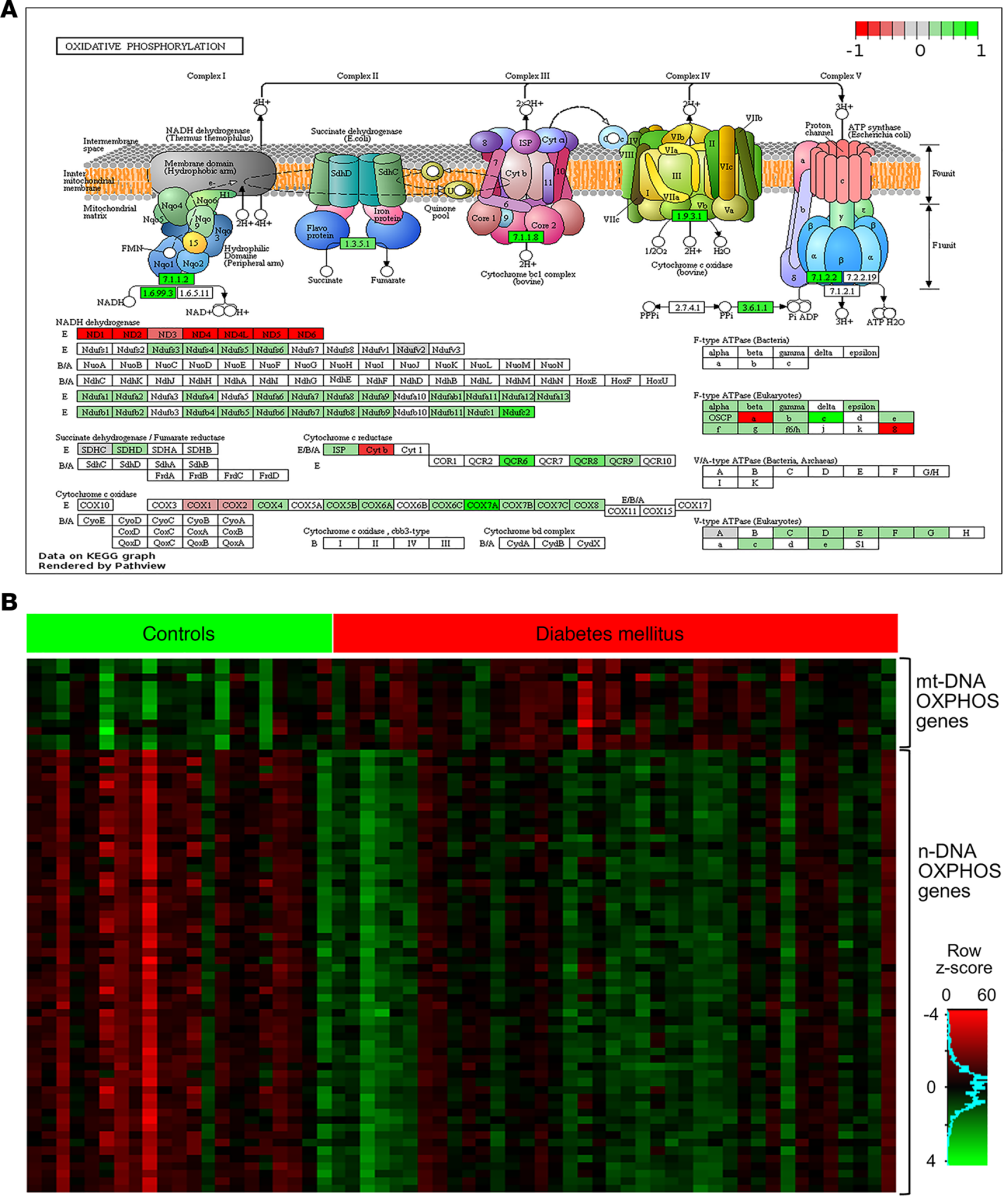
Differentially expressed oxidative phosphorylation genes in DGE (*n* = 39) versus healthy controls (*n* = 21). (**A**) The machinery for oxidative phosphorylation (i.e., complexes 1–4) of the electron transport chain and complex 5 (i.e., ATP synthase). Below, the genes with a FDR < 0.05 are colored in shades of red and green based on the log_2_FC for the gene. Figure created using Pathview (https://academic.oup.com/nar/article-lookup/doi/10.1093/nar/gkx372). (**B**) A heatmap of gene expression values log_RPKM+1_ for differentially expressed OXPHOS genes in patients with DGE (*n* = 39) and healthy controls (*n* = 21). OXPHOS, oxidative phosphorylation; DGE, diabetic gastroenteropathy; RPKM, reads per kilobases per million.

**Figure 4:**
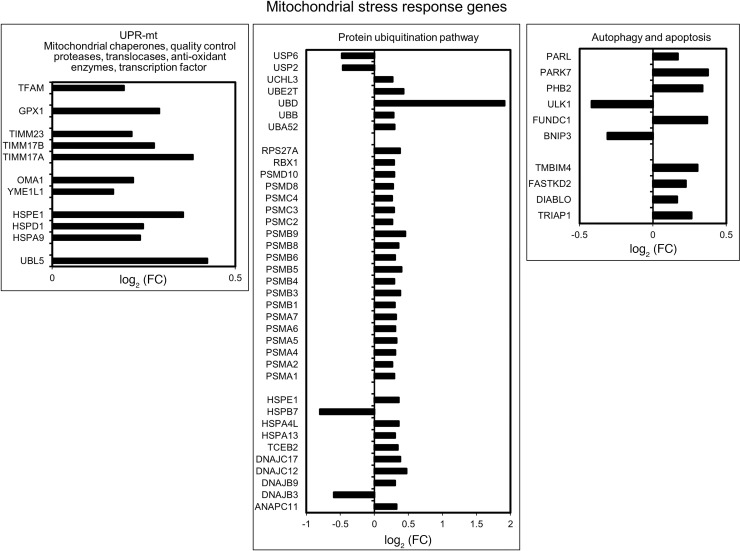
Mitochondrial stress response genes. Mitochondrial stress response genes that are differentially expressed in DGE (*n* = 39) versus healthy controls (*n* = 21) analyzed with EdgeR.

Of 1158 mitochondrial proteins in MitoCarta 2.0, 1145 proteins are encoded by nuclear DNA. Of these 1145 nuclear genes, 255 (22%) were upregulated (FDR < 0.05) in DGE. However, FC were modest and ranged from 1.1 to 1.6 (Supplemental Tables 2 and 3). The overlap was significant with a hypergeometric test *P* value of 1.8 × 10^–79^. These nDNA-encoded genes subserve mitochondrial functions such as OXPHOS (Figure 3), carbohydrate metabolism, mitochondrial stress response, protein translocation into mitochondria, mitochondrial translation machinery, mitochondrial iron sulfur cluster assembly, and antioxidant processes (Supplemental Table 3). The mitochondrial stress response genes that were upregulated in DGE include mitochondrial chaperones (*HSP60*, *HSP10*, *GRP75*), mitochondrial quality control proteases (*YME1L1*, *OMA1*), antioxidant genes (*GPX1*, *GSTA3*, *PRDX1*, *PRDX2*, *PRDX3*), translocases of inner and outer mitochondrial membrane (*TIMM23*, *TIMM17A*, *TIMM17B*), transcription factor A, and mitochondrial (*TFAM*). Among mitophagy-related genes, *ULK1* and *BNIP3* were downregulated, and *FUNDC1*, *PHB2*, *PARK7*, and *PARL* were upregulated (Figure 4).

### Other differentially expressed genes.

Expression of several proteasomal lid, base, and core subunit genes were increased in DGE. Other genes necessary for protein ubiquitination — such as *UBB*, *UBD*, and *UBE2T* (an E2 enzyme) — were also upregulated in DGE. Among deubiquitinating enzymes, *UCHL3* had greater expression, while *USP2* and *USP6* had lower expression in DGE.

Thirty-six genes coding for ribosomal proteins of the larger 60S subunit, and 24 genes coding for small (40S) ribosomal proteins, were upregulated in patients versus controls. None of the ribosomal rRNAs were differentially expressed, although multiple small nucleolar RNAs (snoRNAs) involved in rRNA maturation were upregulated in DM (Supplemental Table 1). Eukaryotic translation initiation factors *EIF3I*, *EIF3K*, *EIF3L*, *EIF4A1*, and *EIF4A3*; the translation repressor protein *EIF4EBP1*; and several tRNA synthetases were all upregulated in DGE

### Type 1 versus type 2 DM.

**Figure 5:**
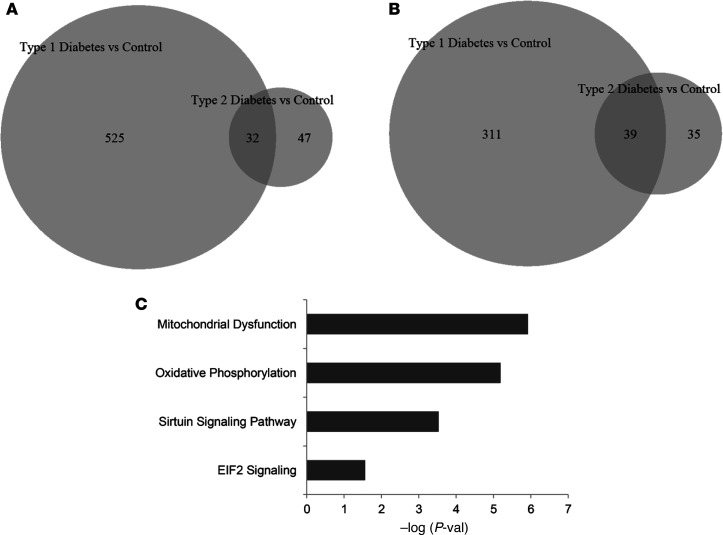
Differentially expressed genes in type 1 and/or type 2 DM. (**A**) Of 557 upregulated genes in type 1 DM (versus controls), 32 were also upregulated in type 2 DM (versus controls); 47 genes were only upregulated in type 2 DM (versus controls). (**B**) The overlap between downregulated, differentially expressed genes between type 1 and type 2 DM. Data are based on 22, 17, and 21 participants, including patients with type 1 DM, patients with type 2 DM, and controls, respectively. (**C**) The pathways that are significantly enriched with the 71 differentially expressed genes in T1D (versus controls) and T2D (versus controls). Observe that the overlapping pathways pertain to mitochondrial dysfunction and oxidative phosphorylation, which suggests that these disturbances are common to both type 1 and type 2 DM.

Thirty-two genes were upregulated and 39 genes were downregulated in both type 1 and type 2 DM, versus controls (Supplemental Table 4). These overlapping genes were linked to mitochondrial dysfunction and OXPHOS, sirtuin signaling, and EIF2 signaling (Figure 5).

### Principal component analysis.

**Figure 6:**
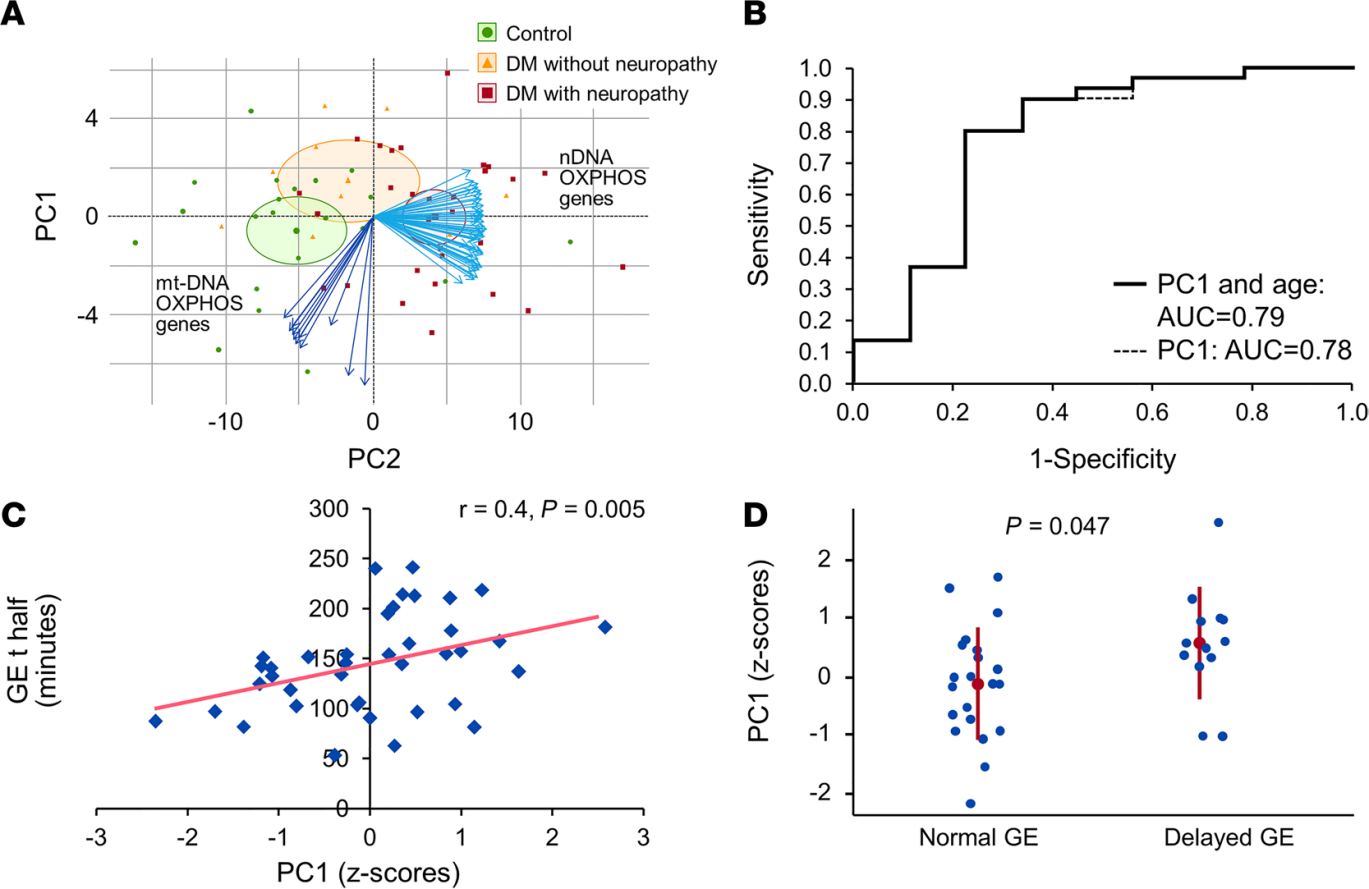
Relationship between principal components analysis of OXPHOS genes, neuropathy, and delayed GE. (**A**) The principal component analysis of 70 OXPHOS genes segregated DM patients from controls and separately DM patients with (*n* = 30) versus without neuropathy (*n* = 9). The blue arrows represent 58 nuclear and 12 mitochondrial DNA–encoded OXPHOS genes that were used in the principal component analysis. (**B**) The receiver operating curves indicates that PC1 discriminated between DGE patients with (*n* = 30) versus without neuropathy (*n* = 9) with an AUC of 0.78 (alone) or 0.79 (PC1 + age). (**C** and **D**) The *z* scores for PC1 were correlated with the GE t half. PC1 scores were significantly greater in DGE patients with delayed (*n* = 13) compared with normal GE (*n* = 21); Wilcoxon test *P* < 0.05. OXPHOS, oxidative phosphorylation; DGE, diabetic gastroenteropathy; PC1, Principal Component 1; GE t half, Gastric emptying half time.

A principal component analysis of the probit transformed normalized gene expression data for the 70 OXPHOS genes that were differentially expressed between DGE and segregated controls from DGE. The first 3 principal components (PC1, PC2, and PC3) together explained 89% variability in the expression data. PC1 alone explained 76% of this variability, suggesting a high degree of correlation among the expressions of individual OXPHOS genes. Among patients, (a) a greater PC1 score was associated with reduced and increased expression of 10 of 12 mtDNA (*P* < 0.05) and all 58 nDNA OXPHOS genes (*P* < 0.0001) (Figure 6 and Supplemental Table 5), (b) a higher PC1 score significantly predicted the presence of neuropathy (AUC = 0.79, *P* = 0.01) after adjusting for age, (c) the PC1 score was correlated (*r* = 0.4, *P* = 0.005) with longer gastric emptying half time (t half), and (d) the PC1 score was greater (*P* < 0.05) in patients with delayed (*n* = 13) compared with normal (*n* = 21) gastric emptying (Figure 6), predicting 20% (*P* < 0.01) of the variance (adjusted *R^2^* value) in gastric emptying in the linear regression model. The parameter estimate in this model suggests that 1 SD increase in the PC1 score is associated with an increase of 19 minutes in GE t half. The PC1 score was not correlated with DM duration or complications, or HbA1c.

### miRNA analysis.

Six of 30 differentially expressed (*P* < 0.05) miRNAs were upregulated in DGE (Supplemental Table 6). The miRNA target filter identified 248 anticorrelated miRNA-mRNA pairs in our data (Supplemental Table 7). Of these, 36 miRNA-mRNA pairs included 17 miRNAs that target mRNAs, which code for mitochondrial proteins (Table 3). Except for miR-7974 (log_2_FC = 0.7), which is the only miRNA that targets a mtDNA-encoded gene, the expression of all these miRNAs was reduced in DGE (log_2_FC ranging from –0.74 to –0.53), consistent with the upregulation of their targets. The miRNAs that were predicted to regulate differentially expressed OXPHOS genes include hsa-miR–101-3p, hsa-miR–193b-3p, hsa-miR–29c-3p, hsa-miR–451a, hsa-miR–582-5p, and hsa-miR–7974.

### Motif analysis.

**Figure 7:**
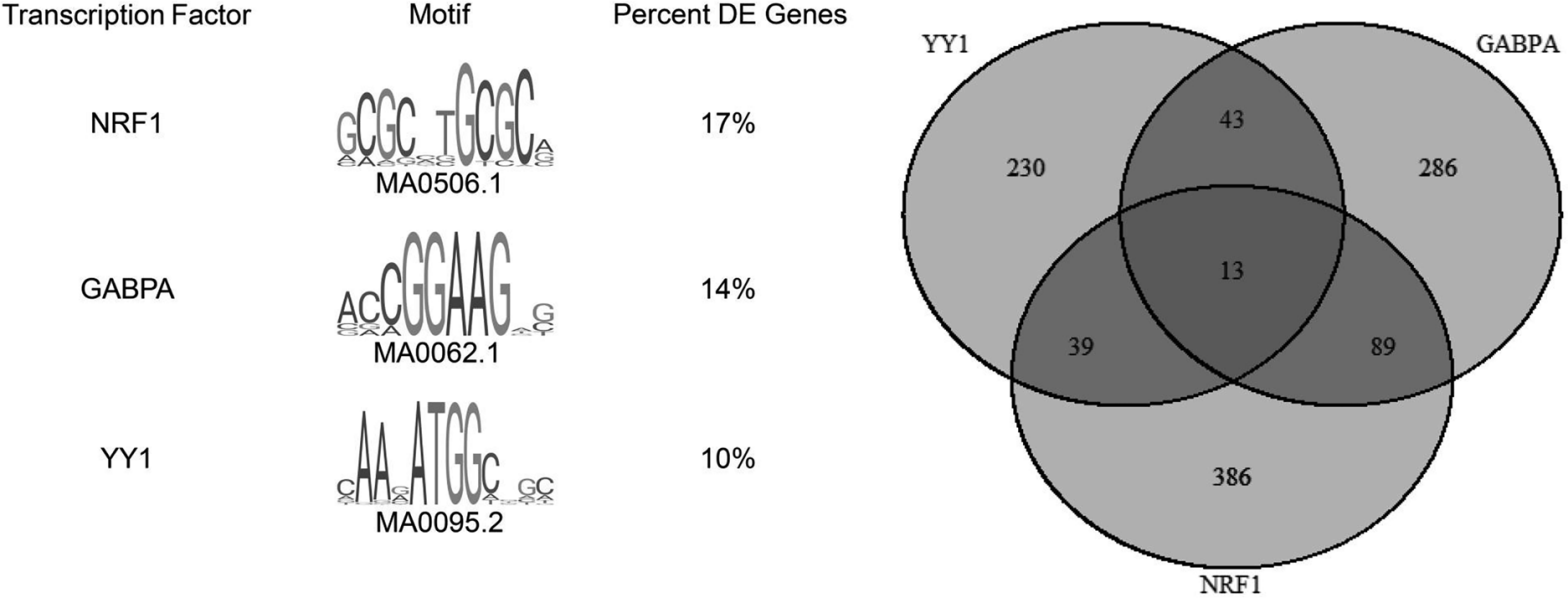
FIMO analysis. FIMO analysis for NRF1, GABPA, and YY1 in the core promoter sequences of the differentially expressed genes between DGE (*n* = 39) and healthy controls (*n* = 21). Venn diagram shows the number of genes (out of the 3175 differentially expressed genes) whose promoters have binding sites for these 3 transcription factors.

There were 118 enriched motifs in the core promoters of 3175 differentially expressed genes (E value [estimate of the expected number of motifs with the given log likelihood ratio or higher, and with the same width and site count, that one would find in a similarly sized set of random sequences] < 0.05, JASPAR 2018 core vertebrate nonredundant database [see Methods]) (Supplemental Table 8). Among the most enriched motifs — excluding general transcription factors — were NRF1, GABPA, YY1, CREB1, and the MEF2 family of transcription factors associated with expression of mitochondrial genes. NRF1, GABPA, and YY1 are predicted to bind 17%, 14%, and 10% of the promoters, respectively (Figure 7 and Supplemental Tables 9–11). Other transcription factors (e.g., KLF, ETS, Fox, and AP-1 families) were also identified. These results were confirmed using a second transcription factor network analysis, WebGestalt (WebGestalt.org) that queried 3175 differentially expressed genes against curated GSEA databases (http://www.webgestalt.org/) for transcription factor target genes. We found significant enrichment for many of the same transcription factors, including NRF1, GABPA, YY1, ELK1, and USF.

### Mitochondrial morphology.

**Figure 8:**
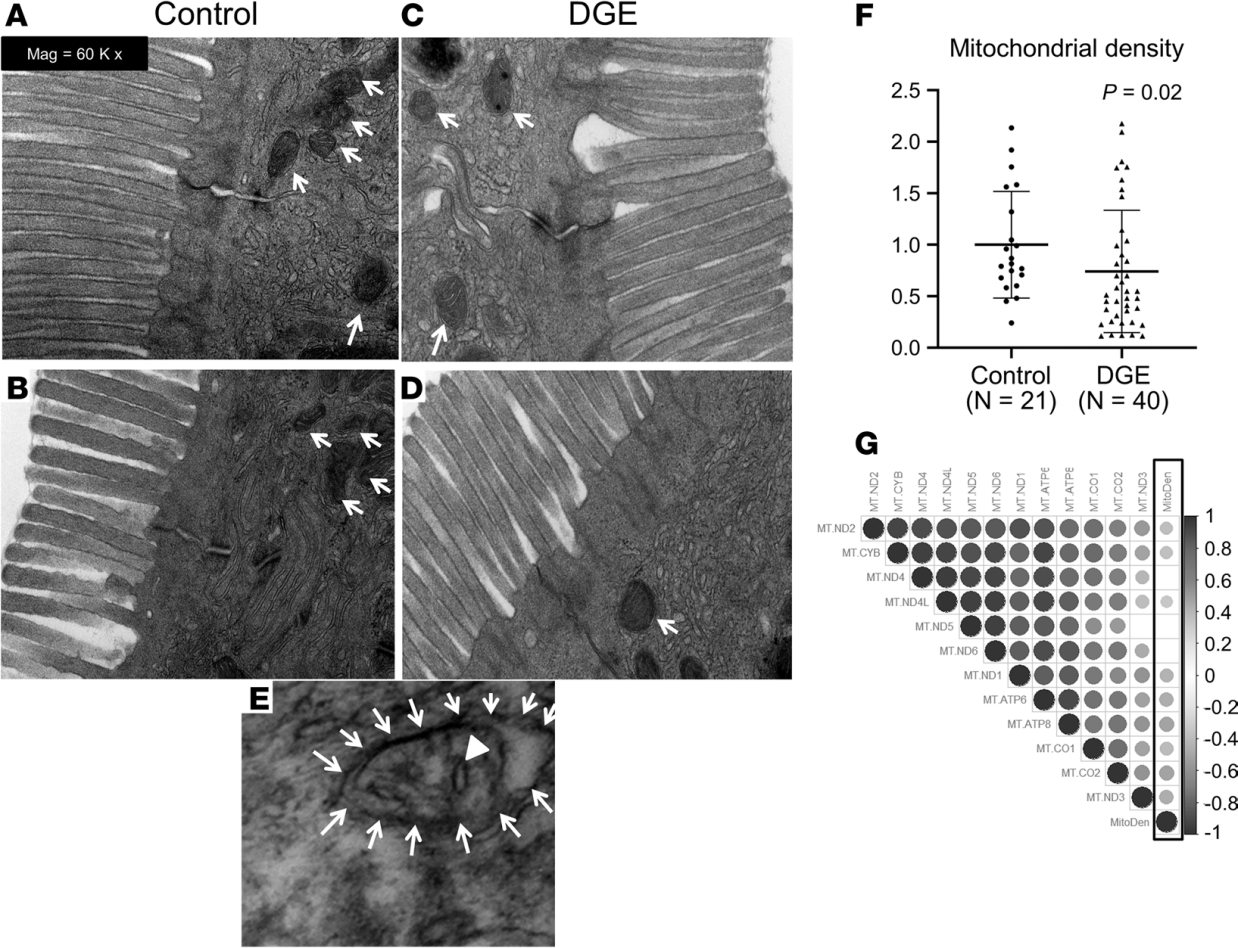
Electron microscopy of duodenal mucosal biopsies in controls and DGE. (**A**–**D**) Compared with healthy controls (**A** and **B**), there are fewer mitochondria, denoted with arrows (i.e., reduced mitochondrial density) in DGE (**C** and **D**). (**E**) A mitochondrion engulfed by a vacuole (arrows), and disintegrating cristae (arrowhead) in a patient with DGE. (**F**) Mitochondrial density in controls and DGE patients (analysis of 30 images in controls [*n* = 21] and DGE patient [*n* = 40], Wilcoxon test). Results were normalized by the mean value of the control samples set to 1 unit. (**G**) The Spearman correlation coefficients, represented by the color and size of the circles, between the expression of differentially expressed mtDNA OXPHOS genes and mitochondrial density (surrounded by black rectangle). Empty cells represent insignificant correlations (*P* value cut off = 0.05); DGE, diabetic gastroenteropathy; mtDNA, mitochondrial DNA; OXPHOS, oxidative phosphorylation. Total original magnification, 60,000×.

Assessed in 40 DM patients and 21 controls, the average mitochondrial area (controls, 75164 nm^2^ [30919 nm^2^]; DM, 81617 nm^2^ [41359 nm^2^]), average matrix density (controls, 0.014 [0.004]; DM, 0.013 [0.004]), crista density (controls, 1.3 [0.7] per mitochondria; DM, 1.3 [0.96] per mitochondria), and mitochondrial circumference (controls, 1008 nm [199 nm]; DM, 1050 nm [255 nm]) (brackets indicate SD) were not significantly different between DGE and controls. Likewise, these parameters were not different between patients with normal or rapid versus delayed GE (data not shown). The mitochondrial density (i.e., number of mitochondria per image averaged over 30 fields in each participant) was significantly lower in DM patients than controls (Figures 8, A–D and F; *P* = 0.02). Some patients but no controls had morphologic alterations such as mitochondrial vacuolization and disorientation of cristae (Figure 8E). Among DM patients, the mitochondrial density was correlated with the expression of 9 of 12 mtDNA OXPHOS genes that were downregulated in DM patients (Figure 8G).

## Discussion

There were 3175 differentially expressed duodenal mucosal genes between DGE and healthy controls. The pathways that were most significantly enriched with the differentially expressed genes were OXPHOS and mitochondrial dysfunction. Compared with controls, the duodenal mucosal expression of several mtDNA-encoded and nDNA-encoded genes were reduced and increased in DGE. A principal component, which was defined by reduced expression of 10 of 12 mtDNA and increased expression of all 58 nDNA OXPHOS genes, was associated with a neuropathy and with delayed GE in DGE. Mitochondrial density was significantly lower in DGE patients than controls and correlated with the reduced expression of mtDNA-encoded OXPHOS genes in DGE patients. These differences in mtDNA- and nDNA-encoded mitochondrial genes were inversely correlated with several differentially expressed miRNAs. Together, these findings suggest a role for clinically relevant mitochondrial disturbances in DGE. These inferences are based on associations; they do not confirm cause and effect. Because we did not study DM patients without gastrointestinal symptoms, which disturbances are related to DM per se are unknown.

Among DGE patients, the reduced mucosal expression of mtDNA and increased expression of nDNA-encoded OXPHOS genes was associated with a neuropathy and with delayed gastric emptying. The differences in mucosal OXPHOS gene expression are consistent with the widespread manifestations of mitochondrial injury (e.g., on hepatocyte metabolism, cardiac output, skeletal muscle contraction, insulin production, and neuronal health) in DM (9, 12). More broadly, studies conducted in blood or peripheral mononuclear cells provide compelling evidence that DM has systemic effects on epigenetic regulation, inflammation, and immunity (4, 10, 11). Although mucosal biopsies contained mRNA for *VIP*, which is the most abundant neuronal population in the submucosal plexus, and glial markers (e.g., *Sox10*, *GFAP*, and *S100B)*, the observed differences in expression of mitochondrial genes probably reflect effects on duodenal mucosa rather than nerves. Because assessment of myenteric neurons requires full-thickness gastrointestinal biopsies, which are seldom performed in clinical practice, or even in research, mucosal biopsies provide a minimally invasive marker of clinically relevant mitochondrial disturbances in DGE. However, the use of duodenal mucosal biopsies as surrogate markers of full-thickness biopsies needs to be validated. In the stomach, there are few intrinsic primary afferent neurons in the submucosa; peristalsis is programmed by slow waves initiated by the interstitial cells of Cajal. By comparison, the findings of duodenal mucosal biopsies are probably more relevant to motor function because nerves in the small intestine serve as sensory terminals of intrinsic primary afferent neurons and the afferent limb of the peristaltic reflex (13), as well as the vagal reflexes regulating gastric emptying in response to chemical signals (14). In addition to assessing the mitochondria, assessment of the morphology and density of duodenal mucosal nerves may provide a useful surrogate marker of autonomic neuropathy in patients with type 1 DM gastroparesis akin to the stomach (15).

Among the mtDNA genes, the changes are substantial; the expression of 12 of 13 protein coding genes, both rRNAs and 9 of 22 tRNAs, was reduced. These findings could be explained by reduced mtDNA copy number (e.g., due to loss of mitochondria), mtDNA mutations, damage to mtDNA, impaired transcription (16), or a combination. Similar disturbances have been implicated to cause end-organ dysfunction in DM patients and animal models (17). The activity of mitochondrial electron transport chain complexes, which are partly encoded by mtDNA, are also impaired in the skeletal muscle of T2DM patients (18), as well as in the kidneys (19) and dorsal root ganglia of diabetic rats (20). Perhaps retrograde signaling from the mitochondria to the nucleus explains why reduced mtDNA gene expression was associated with increased expression of several nDNA genes that code for mitochondrial proteins (21). This retrograde signaling is mediated by 4 mechanisms (i.e., biogenesis, mitochondrial fusion/fission, mitophagy, and movement) that serve to restore mitochondrial homeostasis (21). For example, aging is accompanied by reduced cytochrome c–oxidase (COX) activity, which is encoded by mtDNA, and increased SDH complex activity, which is entirely coded by nDNA (22). This mitochondria-nuclear crosstalk is conserved among species. Indeed, several nuclear genes that are involved in mitochondrial biogenesis were upregulated, to a modest extent (log_2_FC < 1), both in rho^0^ (i.e., mtDNA devoid) yeast cells (23) and in this study, possibly in response to a primary reduction in mitochondrial density. This list includes components of respiratory complexes (*COX5B*, *COX14*), cytochrome oxidase assembly factors (*PET100*), ubiquinone synthesis genes (*COQ5*, *COQ3*), mitochondrial ribosomal proteins (*MRPL17*, *MRPL13*, *MRPL3*, *MRPL36*, *MRPL27*, *MRPL35*), and translocases of the inner and outer mitochondrial membranes (*TIMM17*, *TOMM6*). The expression of selected TCA genes (e.g., isocitrate dehydrogenase 1 [*IDH1*] and malate dehydrogenase 1 [*MDH1*]) that serve to provide metabolic intermediates for anabolic processes was also increased (23, 24). Several upregulated nDNA genes that subserve OXPHOS and TCA (e.g., *ATP5J*, *COX7B*, *NDUFB1*, *NDUFB8*, *SDHC*, and *FH*) are also increased in DM patients with rapidly progressive nephropathy (25).

The expression of mtDNA and nDNA OXPHOS genes was negatively and positively correlated, respectively, with PC1, which suggests that PC1 reflects the severity of the mitochondrion-nucleus imbalance. The PC1 score predicted a neuropathy and prolonged gastric emptying t half in DGE. We suspect that the mitochondrion-nucleus imbalance in duodenal mucosal gene expression, which was more severe in patients with neuropathy, reflects a global imbalance in mitochondrial gene expression in this subset of DM patients. Indeed, prolonged mitochondrial retrograde signaling and impairment of OXPHOS cause neuronal dysfunction (26). Oxidative stress and abnormal mitochondrial functions play key roles in the pathogenesis of diabetic neuropathy and degenerative neuropathies (6, 27). Several emerging therapeutic options for restoring mitochondrial function have entered clinical trials (28).

It seems likely that oxidative stress associated with suboptimal glycemic control, which has also been demonstrated in the gut (29), damages the mtDNA, as observed in this study, and decreases duodenal mucosal expression of mtDNA genes in DGE. Hyperglycemia increases the production of pyruvate. As a result, more reducing equivalents enter the electron transport chain, resulting in mitochondrial membrane hyperpolarization and generation of free radicals (30). Mitochondria are the predominant source of reactive oxygen species in DM (31). mtDNA is particularly susceptible to oxidative damage because it is in close proximity to the electron transport chain and repair capacity is limited. mtDNA damage could then lead to mitochondrial depletion via increased mitophagy (32). Indeed, some mitophagy-related genes (*FUNDC1*, *PHB2*, *PARK7*, *PARL*) were upregulated in the DGE samples.

Several nucleus-encoded mitochondrial stress response protein genes such as *HSP60*, *HSP10*, and *MT-HSP70* (mitochondrial chaperonins); *YME1L1* and *OMA1* (quality control proteases); and *GPX1*, *PRDX3*, *PRDX4*, and *PRDX5* (antioxidant genes) were also upregulated in DGE. These alterations are consistent with the mitochondrial unfolded protein response (UPR-mt), which is induced by depletion of mtDNA and the resulting imbalance in the ratio of mitochondrial and nuclear proteins (33). The serum and saliva concentrations of mitochondrial chaperonin HSP60, a component of the UPR-mt, is also increased in DM (34). Proteasome-based protein ubiquitination represents yet another mitochondrial quality control pathway (35), which may explain the upregulation of most proteasomal subunits and other related genes (e.g., ubiquitin B, ubiquitin D, ubiquitin conjugating enzyme E2 T) in DGE.

The motif analysis of differentially expressed genes identified binding sites for several transcription factors, of which NRF1, GABPA, YY1, CREB, and MEF2 family of transcription factors regulate mitochondrial biogenesis (36). The translocation of TFAM, which activates transcription of mtDNA, into the mitochondria is impaired in rats with diabetic retinopathy (37). It is conceivable that similar mechanisms might be responsible for lower mtDNA expression despite higher *TFAM* mRNA levels in DGE.

Of the 30 differentially expressed miRNAs in this study, some with predicted mitochondrial gene targets have been previously linked with DM and its complications. For example, miR-29c was downregulated and associated with atherosclerotic plaques in patients with DM, as well as in STZ-induced DM rats (type1 DM) and Zucker rats (type 2 DM) (38). The expression of miR–15a-5p in urinary exosomes was associated with diabetic nephropathy (39). The expression of miR–190a-5p in lumbar spinal dorsal horns was lower in STZ-induced diabetic mice with neuropathic pain than in healthy mice (40). In addition to the predicted targets in Table 3, miR–101-3p and miR–127-5p — which were downregulated in this study — also control the expression of the mitochondrial ATP-synthase subunit ATP5B, which was also upregulated in DGE (41, 42). Through posttranscriptional regulation of mitochondria-related genes, these differentially expressed miRNAs may partly explain the altered duodenal mucosal expression of mtDNA- and nDNA-encoded mitochondrial genes and reduced mitochondrial density.

To conclude, DGE is associated with reduced duodenal mucosal expression of mtDNA genes, with a concomitant increase in nDNA-encoded mitochondrial genes. These differences are associated with clinical manifestations (i.e., neuropathy and more prolonged GE) and may be partly explained by differential expression of selected miRNAs. Further experiments should investigate the mechanisms responsible for altered expression of mitochondrial genes in DM and the contribution of specific genes to complications, specifically gastroenteropathy and neuropathy.

## Methods

### Study design and participants.

From June 2014 to April 2017, 40 patients with DGE and 24 healthy volunteers aged between 18 and 70 years were, respectively, identified from our clinical practice and by public advertisement. No participants had significant systemic diseases that may interfere with study objectives or pose safety concerns; neither did they have gastrointestinal surgery other than appendectomy, cholecystectomy, hysterectomy, or inguinal hernia repair. No patients were taking medications that can affect gastrointestinal motility (e.g., amylin analogs, metoclopramide, and opioids), and no patients were pregnant.

### Clinical features.

All patients had symptoms of dyspepsia, as determined by a gastroenterologist. Gastrointestinal symptoms over the preceding 2 weeks were evaluated with the Patient Assessment of Upper Gastrointestinal Disorders-Symptom Severity (PAGI-SYM) questionnaire and summarized as subscores (43). Designed to study patients with a spectrum of symptom severity, the inclusion criteria did not specify a minimum severity for gastrointestinal symptoms. Diabetic neuropathy was defined by abnormal physical examination findings (e.g., diminished or absent reflexes and reduced sensation for light touch, vibration, or pinprick), by abnormal electromyography, or by autonomic (i.e., vagal, sudomotor, or adrenergic) dysfunctions on standardized autonomic function testing where available. Nephropathy was defined by one of the following criteria: (a) a serum creatinine greater than 123.76 μmol/L in males and 106.08 μmol/L in females, (b) proteinuria (i.e., urine protein/osmolarity ratio ≥ 0.2 on a spot urine specimen or urine albumin excretion > 300 mg/day), or (c) microalbuminuria (i.e., urine albumin excretion between 30 and 300 mg/day). A retinopathy was defined by physical examination.

### Gastric emptying and small intestinal transit.

Gastric emptying of solids was assessed with a meal (296 kcal; 32% proteins, 35% fats, and 33% carbohydrates) with 2 eggs labeled with technetium Tc^99m^ (99m technetium pertechnetate) sulfur colloid (1 mCi) and served on 1 slice of bread with milk (8). Rapid and delayed emptying were defined as ≥ 36% emptied at 1 hour and as < 76% emptied at 4 hour, respectively.

### Autonomic functions.

Cardiovagal, adrenergic, and sudomotor functions were evaluated with standardized techniques (8). A semiquantitative composite autonomic severity score (CASS) ranging from 0 to 10 was calculated by combining sudomotor (range, 0–3), cardiovagal (range, 0–3), and adrenergic (range, 0–4) scores that were adjusted for age and sex.

### Upper gastrointestinal endoscopy.

An investigator performed an upper gastrointestinal endoscopy and obtained mucosal biopsies from the second part of the duodenum.

### RNA extraction and sequencing.

RNA was extracted from freshly frozen duodenal tissue using TRIzol (Ambion) and chloroform. After centrifugation (17,000 x *g* for 15 minutes at 4°C), the aqueous phase was purified using an RNeasy Mini Kit (Qiagen) following the manufacturer’s instructions. Total RNA was analyzed using the Agilent Bioanalyzer 2100, and samples had an RNA integrity number of at least 7. The total RNA underwent a size selection process, optimized for mRNA products, and was sequenced on an Illumina HiSeq 4000. The RNA sequencing (RNASeq) files from this experiment can be accessed in GEO under the accession number GSE151497. After sequencing, the mRNA data were processed using Mayo Clinic’s standard RNASeq application, MAPR-Seq v2 (44). The transcriptome used for this analysis was Ensembl’s GRCh38.78 reference. After evaluating mapping percentages, total reads, gene body distribution, and other quality metrics, 21 controls and 39 patient samples looked satisfactory. The Bioconductor edgeR package identified differentially expressed genes with a FDR of 0.05 (45). The GEO datasets (and corresponding secure token numbers) are accessible at https://www.ncbi.nlm.nih.gov/geo; the accession numbers are GSE151497 (secure token, mpanqoukvxerdad), GSE151495 (secure token, ubsxkumgblsvfib), and GSE151496 (secure token, arereoogfnqffov).

### Functional analysis.

IPA and KEGG pathway enrichment analysis were used to determine pathways enriched with the differentially expressed genes. Furthermore, MitoCarta 2.0, a database of 1158 proteins with mitochondrial localization, was used as a reference for nDNA- and mtDNA-encoded mitochondrial genes (46), and a hypergeometric test was performed to assess the significance of overlap between this database and differentially expressed genes in the study.

### Motif analysis.

Core promoter sequences of differentially expressed genes were determined using RSAT (47) with a cutoff of ± 100 bp from the transcription start site. The transcription factor binding motifs in these sequences were determined with Analysis of Motif Enrichment (AME) in the MEME suite (48) using default parameters and were confirmed using a second approach, transcription factor network analysis, in WebGestalt (49) using default parameters. Frequency and location of motifs of interest were quantified with Find Individual Motif Occurrences (FIMO) scanning of core promoter sequences using default parameters (50).

### miRNA sequencing.

The miRNA data were analyzed with the CAP-miRSeq workflow (51), which uses Cutadapt to trim adapter regions and then aligns the data to reference data using MiRDeep2 (52) to detect potentially novel and known miRNAs. The miRNA differential expression analysis was performed using Bioconductor edgeR (45). MicroRNAs were considered to be differentially expressed based on the level of significance of the call (*P* < 0.05) and the magnitude of change of the call |log_2_(FC)| > 0.5.

### Prediction of miRNA targets and construction of integrated miRNA-mRNA networks.

The microRNA Target Filter function in IPA was used to identify regulatory miRNA-target interactions (Ingenuity Systems; http://www.ingenuity.com), similar to previous publications (53). For this purpose, the lists of differentially expressed miRNAs and differentially expressed genes were uploaded to IPA. Experimentally validated and highly predicted targets of the uploaded miRNAs were identified within the list of differentially expressed genes by the microRNA Target Filter function in IPA. This function searches 4 miRNA target databases (TargetScan, miRecords, Tarbase, or Ingenuity expert findings) to determine potential miRNA targets. Subsequently, pairs with anticorrelated expression trends were filtered and selected, and they were used to create a regulatory miRNA-mRNA network.

### Electron microscopy.

The morphology of mitochondria was assessed with digital electron microscopy (JEOL 1400 Transmission Electron Microscopy) at Mayo Clinic. Duodenal mucosal biopsy samples were preserved in Trump’s fixative solution (4% formaldehyde and 0.1% glutaraldehyde in 0.1M phosphate buffer) (Mayo Clinic) overnight at room temperature, mounted on mesh grids, and stained with aqueous uranyl acetate and lead citrate. Thirty fields were randomly visualized at 60,000× in each sample. A region of interest was manually traced around mucosal mitochondria that were fully contained within the borders of the electron microscopy. The ImageJ program (Version 1.5, NIH) was used to measure mitochondrial circumference, area, and density (number of mitochondria per field); matrix density (1/mean gray values); and cristae density (number of cristae per mitochondrion) in all fields (54). The results were averaged per participant.

### Statistics.

The clinical features were compared with Wilcoxon rank sum and Fisher’s exact test. All continuous variables are expressed as mean ± SD, unless specified otherwise. A principal component analysis of 70 differentially expressed OXPHOS genes between patients and controls was performed using probit-transformed normalized expression data using the prcomp function in R. A logistic regression model assessed whether these principal components explained peripheral or autonomic neuropathy. A linear regression model assessed whether these principal components explained gastric emptying t half in DGE. Wilcoxon rank sum test compared the PC1 scores between patients with normal and delayed GE. A *P* value less than 0.05 was considered significant. Statistical analysis was performed using JMP Pro 14 (SAS Institute) and R-3.6.1.

### Study approval.

This study was approved by the Mayo Clinic IRB. Written informed consent was received from participants prior to inclusion in the study.

## Author contributions

SPN analyzed and interpreted the data and drafted the manuscript. DO analyzed and interpreted the data. MS contributed to acquisition and analysis of data. KM interpreted the data and drafted the manuscript. PA interpreted the data. JFP interpreted the data. AE analyzed and interpreted the data and drafted the manuscript. TO interpreted the data and drafted the manuscript. AEB contributed to study concept/design; acquisition, analysis, and interpretation of data; and drafting of the manuscript. All authors critically reviewed the manuscript for important intellectual content.

## Supplementary Material

Supplemental data

## Figures and Tables

**Table 1:**
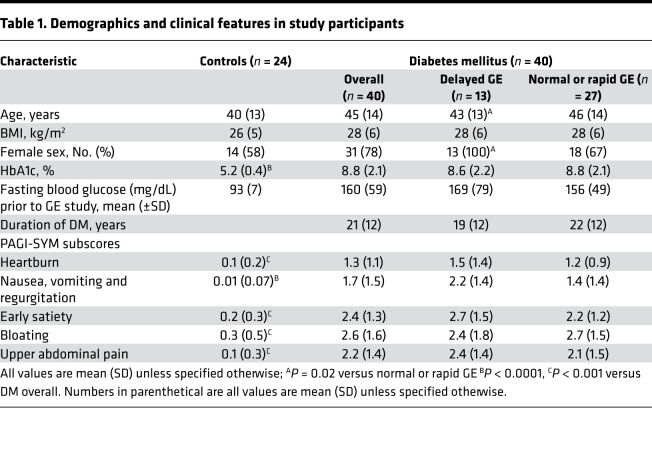
Demographics and clinical features in study participants

**Table 2:**
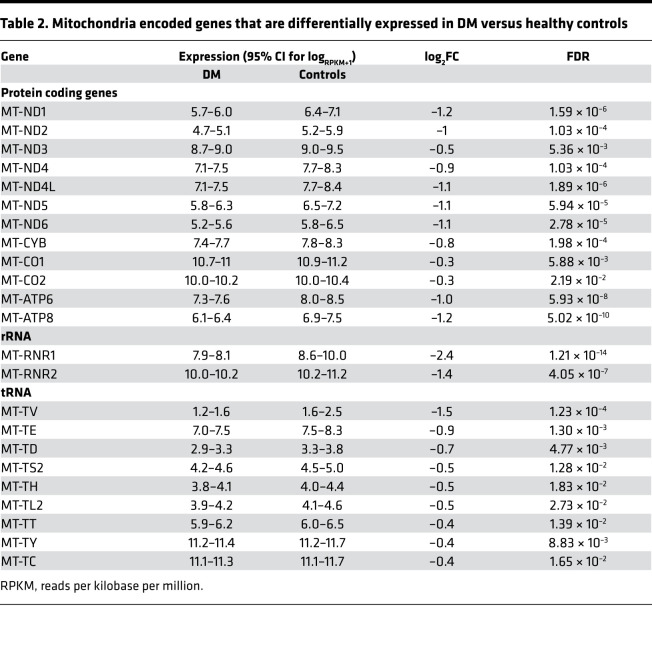
Mitochondria encoded genes that are differentially expressed in DM versus healthy controls

**Table 3:**
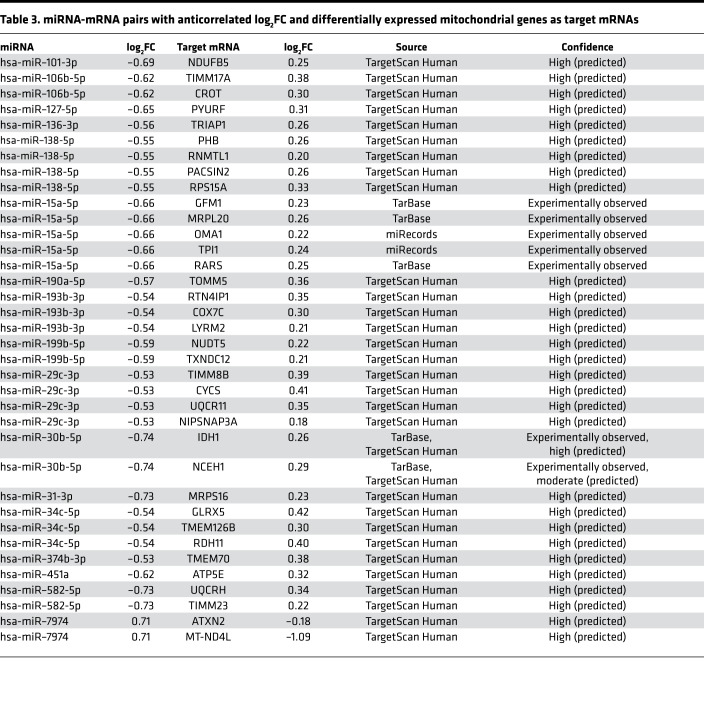
miRNA-mRNA pairs with anticorrelated log_2_FC and differentially expressed mitochondrial genes as target mRNAs

## Version 1 01/25/2021

Electronic publication
