# Supplementary material for: Duodenal mucosal mitochondrial gene expression is associated with delayed gastric emptying in diabetic gastroenteropathy
Source: JCI Insight. 2021 Jan 25;6(2):e143596. doi: 10.1172/jci.insight.143596 (PMC7934845; doi:10.1172/jci.insight.143596)
Supplement: Supplemental data [file jciinsight-6-143596-s037.pdf]

Supplementary Table 1. List of all differentially expressed genes (N = 3175) between DGE and healthy controls

Gene symbol	Ensembl ID	Length	log2FC	P value	FDR
RNA28S5	ENSG00000226958	432	-5.38228	1.45E-27	3.95E-23
RNA45S5	ENSG00000225840	599	-4.12477	3.18E-24	4.35E-20
RN7SL438P	ENSG00000241774	271	-3.967	1.02E-11	3.48E-08
U3	ENSG00000199666	215	-3.67525	4.1E-06	0.00082
KRT5	ENSG00000186081	4292	-3.18203	4.61E-07	0.000208
RP11-846F4.9	ENSG00000264316	781	-3.13693	3.64E-09	5.86E-06
EEF1A1P42	ENSG00000277043	1331	-2.83984	8.81E-08	7.77E-05
LINC00939	ENSG00000249267	6471	-2.64337	3.39E-10	7.72E-07
MTRNR2L1	ENSG00000256618	1553	-2.4514	2.39E-10	5.94E-07
AL353644.10	ENSG00000277048	57	-2.43427	3.37E-20	3.07E-16
MT-RNR1	ENSG00000211459	954	-2.37334	1.77E-18	1.21E-14
MTRNR2L10	ENSG00000256045	1530	-2.13777	1.02E-06	0.000366
AL161626.1	ENSG00000241781	57	-1.96921	1.53E-06	0.00048
NOS2P1	ENSG00000264216	1647	-1.94841	0.000129	0.005521
RP11-475J5.6	ENSG00000270906	1375	-1.93253	0.000185	0.006705
MTRNR2L12	ENSG00000269028	1049	-1.71922	1.64E-09	2.98E-06
MT-TV	ENSG00000210077	69	-1.53028	2.02E-07	0.000123
KRT13	ENSG00000171401	2913	-1.41572	0.005425	0.047709
MTATP8P1	ENSG00000240409	207	-1.40279	1.22E-15	6.68E-12
MT-RNR2	ENSG00000210082	1559	-1.3983	1.48E-10	4.05E-07
PDIA3P2	ENSG00000224677	158	-1.32399	0.00267	0.030946
RP11-249C24.10	ENSG00000260823	444	-1.2183	0.000246	0.007896
RP11-846F4.6	ENSG00000240103	2247	-1.19995	0.001757	0.023972
MT-ATP8	ENSG00000228253	207	-1.19839	1.1E-13	5.02E-10
MT-ND1	ENSG00000198888	956	-1.18933	7.58E-10	1.59E-06
FTH1P8	ENSG00000219507	514	-1.17179	3.59E-05	0.002905
AL353644.4	ENSG00000275104	180	-1.15406	0.005401	0.047601
AGAP7P	ENSG00000264204	2062	-1.15104	0.001349	0.020377
MTRNR2L4	ENSG00000232196	1762	-1.12172	4.71E-08	5.16E-05
MT-ND6	ENSG00000198695	525	-1.11467	2.34E-08	2.78E-05
MT-ND5	ENSG00000198786	1812	-1.10773	5.86E-08	5.94E-05
FMO1	ENSG00000010932	3602	-1.09699	9.92E-05	0.004942
RP11-320N7.2	ENSG00000256969	588	-1.09378	0.000155	0.006058
MT-ND4L	ENSG00000212907	297	-1.08607	9.66E-10	1.89E-06
MTATP6P1	ENSG00000248527	681	-1.08058	5.71E-13	2.23E-09
RP11-378E13.3	ENSG00000276054	735	-1.0466	1.5E-05	0.001714
AC092071.1	ENSG00000269353	549	-1.022	0.000163	0.006205

NOS2	ENSG00000007171	4176	-1.00671	0.000339	0.009347
RP11-354P11.4	ENSG00000264125	697	-0.9862	8.45E-06	0.001248
MTND1P23	ENSG00000225972	372	-0.98074	9.4E-08	7.95E-05
RP11-958N24.2	ENSG00000227827	9932	-0.98011	0.005696	0.048985
MT-ND2	ENSG00000198763	1042	-0.9705	1.38E-07	0.000103
MT-ATP6	ENSG00000198899	681	-0.96332	1.95E-11	5.93E-08
MIR7846	ENSG00000275459	96	-0.96055	6.05E-05	0.003803
GSTA3	ENSG00000174156	1025	-0.9426	0.000458	0.011014
CYP4F23P	ENSG00000269516	1579	-0.93144	0.000176	0.006546
MT-ND4	ENSG00000198886	1378	-0.93061	1.42E-07	0.000103
RP1-66C13.4	ENSG00000266202	582	-0.92236	5.96E-05	0.0038
RN7SL743P	ENSG00000243954	298	-0.91401	3.92E-05	0.002999
MIR4728	ENSG00000265178	67	-0.91209	9.01E-05	0.004712
EGR3	ENSG00000179388	4855	-0.90241	0.000187	0.006739
MT-TE	ENSG00000210194	69	-0.89899	9.14E-06	0.001298
RP11-640A1.4	ENSG00000267487	259	-0.87907	8.33E-05	0.004484
SNRPCP14	ENSG00000254805	205	-0.86572	0.000321	0.009043
RN7SL141P	ENSG00000243398	266	-0.84761	6.99E-06	0.001124
RPL17P44	ENSG00000241088	550	-0.83795	0.000317	0.008988
CTGLF12P	ENSG00000265018	2400	-0.83588	3.6E-05	0.002905
RP11-7K24.3	ENSG00000261068	2027	-0.82211	5.13E-06	0.000947
bP-2171C21.6	ENSG00000279720	501	-0.81742	0.005851	0.049804
MUC3A	ENSG00000169894	11374	-0.81136	7.32E-05	0.004222
HSPB7	ENSG00000173641	3910	-0.79779	0.002543	0.029915
SLC34A2	ENSG00000157765	4699	-0.79661	0.000298	0.008696
RN7SL105P	ENSG00000243704	299	-0.78951	0.000338	0.009337
RP11-139E19.3	ENSG00000278351	254	-0.77869	3.25E-05	0.00276
RP11-64K12.10	ENSG00000261374	1594	-0.77345	0.000533	0.01207
RP11-443P15.2	ENSG00000171658	2628	-0.76646	0.00012	0.005334
MIR1199	ENSG00000277805	119	-0.76192	0.000511	0.011785
AC009961.5	ENSG00000227055	251	-0.75968	0.000242	0.007844
MT-CYB	ENSG00000198727	1141	-0.75704	4.27E-07	0.000198
MIR2116	ENSG00000253030	80	-0.74716	5.54E-05	0.00368
XXbac-B476C20.14	ENSG00000235445	361	-0.74634	0.000172	0.006425
AL354822.1	ENSG00000278384	3027	-0.74532	0.001804	0.024323
DISP2	ENSG00000140323	5171	-0.74391	8.81E-06	0.001294
RNU4-78P	ENSG00000222872	126	-0.74295	0.000342	0.009378
RP11-255M6.1	ENSG00000271044	630	-0.73955	0.00017	0.006394
SEMA6C	ENSG00000143434	5809	-0.73752	7.08E-05	0.00412
RP11-64K12.4	ENSG00000259368	482	-0.73541	0.000189	0.006778
MTND5P11	ENSG00000248923	1812	-0.73374	0.000285	0.008537

TEKT4P2	ENSG00000188681	2309	-0.72598	0.002885	0.032374
NANOGP6	ENSG00000227351	847	-0.72285	0.00016	0.006123
VN2R11P	ENSG00000267447	676	-0.71943	0.003254	0.034852
AP001623.1	ENSG00000216197	72	-0.71792	0.001472	0.021563
MUC12	ENSG00000205277	17130	-0.71791	1.59E-05	0.001783
CTAGE9	ENSG00000236761	2334	-0.71377	0.001225	0.019341
RP11-268J15.5	ENSG00000116883	1838	-0.70458	1.97E-06	0.000529
Y_RNA	ENSG00000206645	102	-0.70456	9.24E-06	0.001298
RP4-704D23.1	ENSG00000234593	579	-0.70349	0.001297	0.019929
RP11-345P4.6	ENSG00000269227	502	-0.70287	6.25E-07	0.000255
AC005753.1	ENSG00000278925	618	-0.70149	0.000983	0.016953
RN7SKP25	ENSG00000199319	308	-0.70093	2.89E-05	0.002574
UGT1A12P	ENSG00000228949	853	-0.69963	0.00078	0.014891
RP11-40E6.1	ENSG00000275741	518	-0.6985	0.000581	0.012703
LIN28A	ENSG00000131914	4008	-0.69528	5.13E-06	0.000947
RP11-58K22.4	ENSG00000255092	567	-0.69273	5.19E-06	0.000947
MT-TD	ENSG00000210154	68	-0.69024	9.31E-05	0.004774
SP5	ENSG00000204335	2060	-0.68897	0.001819	0.024458
CYP2D7	ENSG00000205702	2260	-0.68782	0.000641	0.013406
BBOX1-AS1	ENSG00000254560	1312	-0.6842	0.001713	0.023598
RP11-404P21.5	ENSG00000258412	460	-0.68357	0.000218	0.007399
RP11-731D1.3	ENSG00000250350	163	-0.68256	1.38E-05	0.001625
BMS1P7	ENSG00000270025	1052	-0.6803	1.91E-07	0.000119
MIR4668	ENSG00000266315	70	-0.67696	0.00035	0.009516
RP11-194N12.2	ENSG00000267222	684	-0.67365	0.002108	0.026829
RNA5SP151	ENSG00000202502	117	-0.67272	0.000759	0.014594
RP11-131M6.4	ENSG00000274162	511	-0.67211	0.004475	0.042391
RP11-138H8.8	ENSG00000274297	588	-0.67086	1.82E-05	0.001926
LARGE-IT1	ENSG00000232081	558	-0.66913	0.001183	0.018947
RP11-567P19.1	ENSG00000275927	556	-0.66681	4.35E-05	0.003181
MTND2P28	ENSG00000225630	1044	-0.66497	0.002521	0.029831
RNU4-22P	ENSG00000222990	166	-0.66484	0.000149	0.005883
RP11-336K24.5	ENSG00000224276	401	-0.65921	0.000511	0.011785
RP11-96C23.11	ENSG00000261011	1740	-0.65702	9.31E-07	0.00034
AC245033.1	ENSG00000221095	84	-0.65673	0.000362	0.009657
RP11-83B20.6	ENSG00000279334	415	-0.65171	2.21E-05	0.002133
Metazoa_SRP	ENSG00000277396	262	-0.65168	5.46E-07	0.000231
RP11-104L21.3	ENSG00000273160	303	-0.64858	0.001518	0.021908
RP4-669P10.19	ENSG00000227370	456	-0.64743	0.000593	0.012846
AC092117.2	ENSG00000265864	78	-0.64507	4.4E-06	0.000852
AGAP10	ENSG00000204172	2848	-0.64499	1.71E-05	0.001862

CTAGE4	ENSG00000225932	2588	-0.64215	0.003863	0.038717
RP11-395B7.2	ENSG00000274993	2555	-0.64192	0.000953	0.016723
ACTBP9	ENSG00000266920	1099	-0.64178	0.003238	0.034763
RNU1-134P	ENSG00000199805	165	-0.64025	0.00062	0.013137
CTSLP7	ENSG00000266217	394	-0.636	0.00216	0.027203
CTD-2215L10.1	ENSG00000250619	805	-0.63545	0.00138	0.020627
RP11-345P4.7	ENSG00000269737	1422	-0.63515	1.42E-05	0.001653
CERS1	ENSG00000223802	3753	-0.63129	0.000241	0.007839
AL445183.1	ENSG00000279054	48	-0.63085	0.001155	0.018751
AC010525.4	ENSG00000267117	441	-0.63037	0.001159	0.018793
RP11-138H8.2	ENSG00000259532	472	-0.62993	5.51E-05	0.00368
RP4-687K1.2	ENSG00000259109	242	-0.6279	0.000157	0.006092
RP11-321A17.4	ENSG00000263709	323	-0.62581	2.57E-05	0.002377
RHD	ENSG00000187010	3313	-0.62547	0.000623	0.013182
CTD-2011F17.2	ENSG00000255421	285	-0.62514	2.17E-05	0.002118
ZNF503	ENSG00000165655	3347	-0.6248	4.36E-06	0.000851
RP3-395C13.1	ENSG00000218631	616	-0.62441	0.000848	0.015519
AGAP11	ENSG00000271880	3078	-0.62335	4.01E-07	0.000192
FAM53B-AS1	ENSG00000233334	1760	-0.62186	1.97E-06	0.000529
RP11-96C23.14	ENSG00000272508	1238	-0.62184	4.63E-07	0.000208
AC026449.1	ENSG00000280329	63	-0.62113	5.5E-05	0.00368
RNU6-876P	ENSG00000212597	104	-0.61973	0.000751	0.014502
MAB21L3	ENSG00000173212	3256	-0.61964	3.43E-07	0.000168
BMS1P2	ENSG00000251079	2153	-0.61599	1.41E-06	0.000454
RP11-29B2.6	ENSG00000276957	536	-0.61321	0.004746	0.043885
ESRG	ENSG00000265992	3140	-0.61279	1.55E-05	0.001752
RP11-69I8.3	ENSG00000227220	495	-0.61273	0.002865	0.032315
RP3-405J10.4	ENSG00000257256	325	-0.6124	1.48E-07	0.000104
OR2A4	ENSG00000180658	933	-0.61202	0.000566	0.012509
RP13-36G14.3	ENSG00000203397	510	-0.61193	0.000477	0.011301
RP11-144G6.12	ENSG00000254929	1625	-0.61135	6.82E-06	0.001124
BMS1P3	ENSG00000240089	932	-0.61082	2.13E-06	0.000545
ZNF503-AS2	ENSG00000237149	3348	-0.60905	5.86E-05	0.003767
BMS1P1	ENSG00000204177	4341	-0.60566	6.39E-06	0.001092
SNX18P25	ENSG00000249337	758	-0.6047	0.003608	0.037219
HMGB1P21	ENSG00000248909	581	-0.6034	1.81E-06	0.000504
IRS2	ENSG00000185950	8138	-0.60261	7.31E-06	0.001143
AE000658.30	ENSG00000256350	198	-0.60144	0.005598	0.048489
MIR6753	ENSG00000275022	164	-0.60039	0.000649	0.013494
RP11-91P24.5	ENSG00000254691	551	-0.59977	0.00013	0.005521
RP11-8L18.3	ENSG00000229235	243	-0.59893	0.000412	0.010356

AC108004.3	ENSG00000241525	1223	-0.59734	0.002917	0.032577
TRIM36-IT1	ENSG00000250472	881	-0.59592	0.000846	0.0155
CHAD	ENSG00000136457	1802	-0.59515	0.000491	0.01155
DNAJB3	ENSG00000227802	1266	-0.59385	0.0003	0.008696
FO538757.2	ENSG00000279457	1982	-0.59366	1.06E-05	0.001398
RN7SKP273	ENSG00000223324	314	-0.59302	0.00032	0.009038
ADH4	ENSG00000198099	2453	-0.59258	0.000434	0.010653
MIR614	ENSG00000207817	90	-0.59215	0.00385	0.03861
PLS1-AS1	ENSG00000239641	346	-0.59001	5.56E-05	0.00368
SEMA5A	ENSG00000112902	12097	-0.58977	0.002374	0.028731
RP11-543P15.3	ENSG00000275850	2400	-0.58852	0.000649	0.013494
RPS12P27	ENSG00000240435	337	-0.58749	2.56E-05	0.00237
CTD-2201E9.2	ENSG00000250001	543	-0.5867	0.004658	0.043313
HMGB1P8	ENSG00000259699	949	-0.58599	1.09E-05	0.001412
PEBP4	ENSG00000134020	1329	-0.58539	0.000429	0.010594
RP11-44N21.3	ENSG00000280244	461	-0.58446	0.00202	0.026207
RP3-465N24.5	ENSG00000261349	459	-0.58445	0.00387	0.038751
MIR941-1	ENSG00000215946	72	-0.5843	0.000952	0.016723
RP11-96C23.13	ENSG00000151303	4560	-0.58412	1.9E-07	0.000119
CTD-2600O9.1	ENSG00000187185	2114	-0.58285	0.001004	0.017189
CTB-36O1.7	ENSG00000244921	747	-0.58182	0.000729	0.014283
MIR4296	ENSG00000264572	88	-0.58177	0.000251	0.007968
CTD-2104P17.2	ENSG00000264932	563	-0.58176	9.23E-06	0.001298
POU5F1	ENSG00000204531	2881	-0.58161	0.000637	0.013347
CTA-243E7.1	ENSG00000279110	13120	-0.58065	5.22E-05	0.003557
AC091492.2	ENSG00000225026	272	-0.57964	0.000116	0.005253
TBCAP2	ENSG00000235829	326	-0.57953	0.001057	0.017779
SUMO2P14	ENSG00000271096	293	-0.57704	9.49E-06	0.001311
CTD-2036J7.1	ENSG00000241026	483	-0.57579	0.000599	0.012867
OR2A7	ENSG00000243896	1116	-0.5756	0.000446	0.01086
RP13-726E6.1	ENSG00000254757	450	-0.57467	0.000972	0.016885
MIR6124	ENSG00000275373	85	-0.57407	0.000415	0.010389
AC008671.1	ENSG00000222732	114	-0.57397	0.00169	0.023367
RNU6-960P	ENSG00000200926	120	-0.57301	0.000378	0.009926
CTC-461H2.2	ENSG00000267611	187	-0.57264	0.004961	0.045081
VWCE	ENSG00000167992	4874	-0.57191	0.001496	0.021727
SCN9A	ENSG00000169432	10364	-0.57176	0.003295	0.035126
CTB-186G2.4	ENSG00000267375	242	-0.57071	4.26E-05	0.003151
PPIAP2	ENSG00000227379	492	-0.57061	0.000107	0.005039
SNORA11	ENSG00000221245	127	-0.57016	0.002654	0.030787
MTND2P2	ENSG00000229954	945	-0.56962	0.000511	0.011785

RPL17P11	ENSG00000233445	544	-0.56922	0.000729	0.014283
RP11-494K3.2	ENSG00000229657	562	-0.56911	0.001444	0.021278
RP11-820K3.3	ENSG00000203437	781	-0.56767	0.000228	0.007603
RP11-13N13.5	ENSG00000264188	336	-0.56763	0.00335	0.035539
ZNF415P1	ENSG00000266127	1166	-0.56615	0.001191	0.019039
RP11-214D15.2	ENSG00000227175	380	-0.56543	7.04E-06	0.001126
RP11-295B17.6	ENSG00000271437	538	-0.56538	0.00077	0.014749
RP11-429D19.1	ENSG00000259771	448	-0.56419	0.000192	0.006838
TEX35	ENSG00000240021	4038	-0.56413	0.000178	0.006569
C17orf97	ENSG00000187624	2505	-0.56294	0.005476	0.047906
CRHR2	ENSG00000106113	3984	-0.56246	0.000726	0.014282
RP11-8P13.1	ENSG00000244743	316	-0.56233	0.000547	0.012199
RP11-478C1.7	ENSG00000251148	563	-0.56	0.001417	0.021037
CTD-2373H9.5	ENSG00000267491	945	-0.55687	0.000329	0.009198
RN7SL749P	ENSG00000242853	290	-0.55666	0.000371	0.009816
SLC37A2	ENSG00000134955	6575	-0.55586	0.002302	0.028164
RP11-59H7.3	ENSG00000259732	633	-0.55548	0.000268	0.00824
CTA-221G9.12	ENSG00000203280	957	-0.55502	0.000186	0.006728
AC011243.1	ENSG00000223430	415	-0.55471	0.00463	0.043164
AC011288.2	ENSG00000229618	989	-0.55435	0.001943	0.025399
RP11-91P24.1	ENSG00000241782	474	-0.55157	0.001894	0.025074
RP11-46F15.2	ENSG00000238260	622	-0.5515	0.004782	0.044011
RP11-432I5.8	ENSG00000261513	777	-0.55066	0.00052	0.011906
UGT1A13P	ENSG00000234143	762	-0.55023	0.000248	0.007946
PLEKHN1	ENSG00000187583	2833	-0.54886	0.003067	0.033692
RP11-4B16.3	ENSG00000275178	361	-0.54797	0.000164	0.006222
RP11-93O14.2	ENSG00000261131	321	-0.54664	0.000297	0.008696
RP11-454L9.2	ENSG00000259318	294	-0.54622	2.85E-05	0.002559
Vault	ENSG00000252283	102	-0.54586	0.001161	0.018805
RP11-230L22.4	ENSG00000237336	722	-0.54565	0.000222	0.007437
RP11-750H9.7	ENSG00000270072	430	-0.54516	0.000117	0.005271
RNA5SP212	ENSG00000212525	107	-0.54483	0.000378	0.009926
TRBV29OR9-2	ENSG00000223394	333	-0.54414	9.49E-06	0.001311
RPL21P123	ENSG00000240531	407	-0.54398	0.002527	0.029853
RP11-274A11.3	ENSG00000279294	2059	-0.54396	6.31E-05	0.003815
MUC17	ENSG00000169876	14579	-0.54394	0.000147	0.005854
AC005037.6	ENSG00000235318	298	-0.5438	0.000878	0.015873
RP11-384K6.2	ENSG00000225892	2486	-0.54357	1E-04	0.004942
Z69890.1	ENSG00000206168	339	-0.54281	0.001156	0.018751
MIR5687	ENSG00000265135	77	-0.54252	0.00069	0.013872
RP11-356M20.3	ENSG00000259735	497	-0.54217	0.000263	0.008179

HAPLN4	ENSG00000187664	3569	-0.542	0.000389	0.010045
GS1-115G20.1	ENSG00000230470	423	-0.53835	0.001143	0.018636
MTND1P11	ENSG00000228166	876	-0.53795	0.005123	0.046091
MT-TS2	ENSG00000210184	59	-0.53771	0.00059	0.012791
CTD-2639E6.9	ENSG00000267898	946	-0.53725	0.002213	0.027613
FGF18	ENSG00000156427	1986	-0.53665	0.000382	0.009964
RPL7AP15	ENSG00000236806	795	-0.53626	0.00021	0.007234
RNU6-212P	ENSG00000199512	104	-0.53608	0.003436	0.036161
RP3-405J10.2	ENSG00000257531	354	-0.5358	4.72E-05	0.00335
CTA-243E7.3	ENSG00000279548	2059	-0.53553	0.000357	0.0096
RP11-640L9.1	ENSG00000279727	1344	-0.53527	0.000302	0.008715
RP11-875O11.2	ENSG00000246130	2636	-0.53521	0.000297	0.008696
MTND5P14	ENSG00000230225	1267	-0.53493	0.00025	0.007959
CTD-2024P10.1	ENSG00000250234	407	-0.53364	0.000346	0.009458
AC009095.4	ENSG00000237718	522	-0.5334	0.002229	0.027667
SLC35E1P1	ENSG00000238286	864	-0.53339	0.00011	0.005099
RP11-146N23.4	ENSG00000232978	409	-0.53317	2.9E-05	0.002574
MAPK15	ENSG00000181085	3056	-0.53212	0.001581	0.022448
LINC01555	ENSG00000180869	2884	-0.53199	0.000929	0.016496
RP11-420B22.1	ENSG00000253335	545	-0.53192	0.000622	0.013167
KCTD9P4	ENSG00000214264	1166	-0.53139	0.001306	0.020039
RP11-95G17.2	ENSG00000272472	1384	-0.53123	0.000828	0.015324
AC116533.3	ENSG00000266493	91	-0.52957	0.000467	0.011143
AC011816.1	ENSG00000234073	546	-0.52864	0.000631	0.01327
RP11-274A11.5	ENSG00000279681	506	-0.52856	0.002394	0.028847
SMG6-IT1	ENSG00000278941	374	-0.52499	0.000519	0.011906
RP11-400F19.18	ENSG00000267632	1736	-0.52405	5.28E-05	0.003583
RP11-343B5.1	ENSG00000233597	576	-0.5238	0.000144	0.0058
AP000487.4	ENSG00000254495	394	-0.52377	0.000706	0.014023
GLTSCR1	ENSG00000063169	7507	-0.52337	1.17E-05	0.001472
KB-1205A7.1	ENSG00000254112	319	-0.52309	0.00018	0.006608
ANKRD24	ENSG00000089847	4474	-0.52166	9.98E-06	0.001358
RP11-680F8.4	ENSG00000259644	1346	-0.52072	3.24E-07	0.000161
CTC-507E2.2	ENSG00000267592	225	-0.51953	0.001578	0.022412
SLC30A10	ENSG00000196660	5770	-0.51929	0.000407	0.010313
AL109767.1	ENSG00000235785	564	-0.51908	0.00025	0.007959
AC010525.5	ENSG00000267689	195	-0.51905	0.000733	0.014328
CYP2S1	ENSG00000167600	2874	-0.519	0.000341	0.009378
RP11-1102P22.3	ENSG00000279410	1370	-0.5187	4.68E-05	0.003332
HSPA8P5	ENSG00000256356	2069	-0.518	0.000691	0.013877
RP11-284F21.9	ENSG00000272068	3222	-0.51754	7.35E-05	0.004224

RGS11	ENSG00000076344	4798	-0.5173	0.001238	0.019429
MIR4665	ENSG00000263575	79	-0.51691	0.000719	0.014196
RP5-1071N3.1	ENSG00000231105	2479	-0.51663	5.89E-05	0.003778
CTA-243E7.2	ENSG00000279006	3000	-0.51641	4.82E-05	0.003409
RP11-351O1.3	ENSG00000224412	178	-0.51574	0.005007	0.045356
XXbac-B476C20.13	ENSG00000234913	2493	-0.51538	0.001749	0.023914
AC010203.1	ENSG00000221770	83	-0.51514	0.000702	0.013974
RP11-430B1.1	ENSG00000259398	381	-0.51455	0.004396	0.041985
RPS20P15	ENSG00000227523	311	-0.51438	0.00182	0.024465
NSRP1P1	ENSG00000235613	1693	-0.51387	0.002605	0.030415
RP11-519G16.3	ENSG00000259354	3886	-0.51334	6.66E-05	0.003993
FKSG52	ENSG00000280447	375	-0.51303	0.000103	0.004987
RP11-290H9.4	ENSG00000263293	713	-0.51165	0.00153	0.022014
NR4A3	ENSG00000119508	6314	-0.51119	0.003654	0.037526
IQSEC3	ENSG00000120645	7686	-0.51107	0.005657	0.048806
RP11-618N24.1	ENSG00000261635	725	-0.5098	3.59E-05	0.002905
SLC43A2	ENSG00000167703	10606	-0.50945	0.001124	0.018464
RP4-718P11.1	ENSG00000271426	646	-0.50928	0.003501	0.03665
RP11-792A8.1	ENSG00000236928	521	-0.50849	0.001236	0.019421
RP11-1228E12.1	ENSG00000280279	2096	-0.50731	0.000283	0.008487
RP11-214N15.5	ENSG00000234017	241	-0.50617	8.32E-05	0.004484
MTND2P11	ENSG00000237124	648	-0.50515	0.003879	0.038789
RP11-79M19.2	ENSG00000236426	761	-0.50496	0.000408	0.010313
RP11-332H18.3	ENSG00000266934	1329	-0.50493	0.001767	0.024008
RP11-459I19.1	ENSG00000272555	455	-0.50491	0.000936	0.016547
RP11-68I18.10	ENSG00000261168	1536	-0.50463	0.005313	0.047152
RP1-122P22.4	ENSG00000268628	1648	-0.50444	0.000601	0.012882
RP11-820K3.2	ENSG00000225349	533	-0.50347	0.000238	0.007776
AC093162.5	ENSG00000246575	704	-0.50307	0.000255	0.008025
RP11-9F11.3	ENSG00000279241	583	-0.50272	0.000109	0.00508
ENO1P3	ENSG00000243986	1224	-0.50261	0.002903	0.032472
RP11-667M19.9	ENSG00000255710	1030	-0.50184	0.000453	0.010972
RP11-93O14.3	ENSG00000280376	485	-0.50173	0.001333	0.020271
GRB7	ENSG00000141738	3597	-0.5015	0.000299	0.008696
RP11-730A19.5	ENSG00000228330	793	-0.50095	0.0023	0.028154
RP11-10J21.4	ENSG00000253307	397	-0.4996	0.004072	0.040034
UPF3AP3	ENSG00000234709	769	-0.49945	2.36E-07	0.000134
LINC01358	ENSG00000230812	747	-0.49928	0.005244	0.046852
CTD-2373H9.3	ENSG00000279339	2889	-0.49829	0.000796	0.015076
RNU6-125P	ENSG00000207234	107	-0.49802	0.001289	0.01987
TLX2	ENSG00000115297	2775	-0.49694	0.000668	0.013662

RP11-539G18.1	ENSG00000249019	287	-0.49691	0.000668	0.013662
RPS12P26	ENSG00000225193	396	-0.49623	0.00083	0.015324
RP11-417L19.5	ENSG00000279283	1740	-0.49615	9.29E-05	0.004774
MT-TH	ENSG00000210176	69	-0.49576	0.001103	0.018281
SNORA67	ENSG00000201619	141	-0.49567	0.00342	0.036088
RP11-434D2.3	ENSG00000267075	4582	-0.49537	0.003041	0.033543
RP11-1102P22.1	ENSG00000279415	978	-0.49496	0.000782	0.014919
RP11-353H3.1	ENSG00000223916	515	-0.49471	0.004444	0.042221
GATM-AS1	ENSG00000275672	1424	-0.49447	0.005712	0.049092
SHOX	ENSG00000185960	4384	-0.49428	0.005797	0.0495
FGF5	ENSG00000138675	5998	-0.49369	0.000609	0.012984
ERP29P1	ENSG00000233347	706	-0.49354	0.00423	0.041038
AC114812.9	ENSG00000248114	730	-0.49304	0.002646	0.030718
CTB-151G24.1	ENSG00000267696	595	-0.49236	0.000927	0.016478
XXbac-B476C20.11	ENSG00000236890	210	-0.49207	0.001654	0.023098
RN7SL329P	ENSG00000242170	299	-0.49155	0.003172	0.034388
RP11-439C15.5	ENSG00000270988	497	-0.49151	0.000134	0.005613
L3MBTL4-AS1	ENSG00000264707	2044	-0.48971	0.000142	0.005755
RN7SKP22	ENSG00000201967	293	-0.48936	0.005766	0.049322
MIR1914	ENSG00000272045	80	-0.48934	0.005098	0.045939
RP5-1022P6.3	ENSG00000233578	642	-0.48847	4.4E-05	0.003193
MIR4802	ENSG00000263642	80	-0.48833	0.000819	0.015231
CTD-2515A14.1	ENSG00000261669	579	-0.48818	4.3E-05	0.003163
YWHAQP6	ENSG00000249986	304	-0.48818	0.00144	0.021273
RNF144A-AS1	ENSG00000228203	4648	-0.488	0.000103	0.004987
HMGN1P20	ENSG00000239539	300	-0.48773	0.000448	0.010901
CTD-2611O12.6	ENSG00000268593	306	-0.48744	0.001168	0.01884
XXbac-B476C20.10	ENSG00000235617	277	-0.48702	0.005376	0.047464
RP11-435O5.6	ENSG00000271314	456	-0.48661	0.002902	0.032472
MT-ND3	ENSG00000198840	346	-0.48573	0.000122	0.005357
RP11-458F8.4	ENSG00000273142	3441	-0.48571	0.003315	0.035295
RP11-512F24.1	ENSG00000232499	1029	-0.48546	0.000238	0.007776
DRD4	ENSG00000069696	1477	-0.48545	0.000521	0.011926
LRP1	ENSG00000123384	20839	-0.48511	6.18E-05	0.003815
FP325331.1	ENSG00000278881	1380	-0.48481	0.0006	0.012881
AP000679.2	ENSG00000176984	2081	-0.48459	0.001882	0.024963
RPL21P44	ENSG00000229585	472	-0.48394	0.000253	0.008002
RP11-104L21.2	ENSG00000233411	303	-0.48266	0.002116	0.026871
RP11-274B21.2	ENSG00000243302	1150	-0.48211	6.42E-05	0.003869
SLC22A20	ENSG00000197847	3983	-0.48111	0.00012	0.005334
RP11-291L19.1	ENSG00000236872	325	-0.48079	0.003841	0.038553

RN7SKP154	ENSG00000222068	290	-0.4804	0.005605	0.048489
RP11-320P11.1	ENSG00000203647	431	-0.48007	0.00029	0.008607
RP4-631H13.6	ENSG00000230953	175	-0.47988	4.33E-05	0.003171
NPIP	ENSG00000169203	4331	-0.4796	0.002274	0.02792
RP11-803D5.1	ENSG00000279191	3380	-0.479	0.001204	0.019123
RP11-235E17.3	ENSG00000263338	560	-0.47744	0.002399	0.028867
FMR1-IT1	ENSG00000236337	343	-0.47739	0.001274	0.019704
USP6	ENSG00000129204	11270	-0.47673	0.004602	0.042944
RP11-1J11.1	ENSG00000249170	978	-0.47645	0.000568	0.012524
NPIPB3	ENSG00000169246	4717	-0.47625	0.004826	0.044248
AC114812.5	ENSG00000178836	1041	-0.47575	0.00253	0.029855
RAI14	ENSG00000039560	7687	-0.47468	1.09E-05	0.001412
GS1-124K5.13	ENSG00000279785	1194	-0.47375	0.002552	0.029982
RP11-466A19.1	ENSG00000266718	2480	-0.47303	0.00057	0.012549
MT-TL2	ENSG00000210191	71	-0.47299	0.002166	0.027253
RP11-627G23.1	ENSG00000255545	8255	-0.47251	0.00155	0.022148
RP11-139K1.2	ENSG00000227560	390	-0.47233	0.000524	0.011967
CYP4F3	ENSG00000186529	5860	-0.47192	0.00143	0.021175
LDHAL6B	ENSG00000171989	1693	-0.47141	0.000444	0.010822
RP11-454K7.1	ENSG00000257900	548	-0.47093	1.71E-05	0.001862
AC012513.4	ENSG00000236478	1137	-0.47053	0.000142	0.005755
RSF1-IT2	ENSG00000254985	566	-0.47035	0.000329	0.009198
RP11-504I13.3	ENSG00000263982	398	-0.47034	0.005851	0.049804
RP11-391L3.5	ENSG00000260229	497	-0.46969	0.000334	0.009281
RP11-507J18.5	ENSG00000279665	575	-0.46906	0.000264	0.008179
BCL9L	ENSG00000186174	10470	-0.46869	5.98E-05	0.003801
RP11-6N17.3	ENSG00000266601	335	-0.46839	0.000797	0.015084
PCDH11Y	ENSG00000099715	11672	-0.46805	0.000395	0.010139
RNU7-18P	ENSG00000252174	62	-0.46759	0.003431	0.036144
CTD-3195I5.3	ENSG00000262692	291	-0.46695	0.001627	0.022832
RP11-57A1.1	ENSG00000263680	833	-0.4668	0.002846	0.032169
KLF3-AS1	ENSG00000231160	4830	-0.46602	1.54E-08	1.91E-05
Z85986.1	ENSG00000264878	82	-0.46594	0.00509	0.045918
RP11-583F2.5	ENSG00000266598	537	-0.4659	8.14E-05	0.004448
AC010999.1	ENSG00000222586	73	-0.4655	0.001135	0.018531
RP11-102M11.1	ENSG00000240695	730	-0.4653	8.06E-05	0.004442
KB-1958F4.1	ENSG00000270077	554	-0.46516	0.004714	0.043661
TPM3P6	ENSG00000250731	742	-0.46487	0.000878	0.015873
CHMP4BP1	ENSG00000258469	588	-0.4646	0.000132	0.005569
RCN3	ENSG00000142552	2093	-0.46432	0.000497	0.011616
CDC42-IT1	ENSG00000230068	692	-0.46429	0.000528	0.012001

RP11-314N13.9	ENSG00000280162	3658	-0.46403	0.000404	0.010275
RPL19P12	ENSG00000224415	591	-0.464	3.8E-05	0.002962
HMGN1P2	ENSG00000229046	310	-0.46278	0.001612	0.022761
AC016910.1	ENSG00000232377	1046	-0.46255	0.002063	0.026538
RP11-110I1.11	ENSG00000254428	523	-0.46244	7.92E-06	0.001189
RP6-99M1.3	ENSG00000269902	528	-0.4622	0.001239	0.019429
CTC-499B15.6	ENSG00000266922	491	-0.46211	0.003797	0.038332
USP2	ENSG00000036672	4538	-0.46166	0.000106	0.005029
RP11-193H22.2	ENSG00000219448	5191	-0.46071	0.000412	0.010356
RP11-141B14.2	ENSG00000279957	1173	-0.46057	0.000111	0.00512
RP11-1348G14.8	ENSG00000275807	1539	-0.46057	6.76E-05	0.004015
RP11-13N13.6	ENSG00000265656	447	-0.46043	0.001613	0.022761
AC008985.1	ENSG00000278999	2239	-0.46025	0.001789	0.024188
BTF3L4P1	ENSG00000232260	468	-0.46011	0.002159	0.027203
PDX1	ENSG00000139515	2501	-0.45985	0.000375	0.009902
RP11-296A18.6	ENSG00000236434	435	-0.45972	0.004286	0.041335
FKSG70	ENSG00000279849	375	-0.45949	1.24E-06	0.000419
HIF1A-AS2	ENSG00000258667	533	-0.45934	0.002529	0.029855
FKSG51	ENSG00000280230	375	-0.45917	2.6E-07	0.000139
RP11-100G15.3	ENSG00000228069	779	-0.45914	0.000124	0.005421
AC094019.4	ENSG00000236732	472	-0.45832	0.000747	0.014464
RP11-62H7.2	ENSG00000233609	1442	-0.45761	0.000665	0.013659
RP4-695O20.1	ENSG00000280384	2045	-0.45705	4.85E-05	0.00341
EFHD1	ENSG00000115468	3052	-0.45694	0.005539	0.048173
CTC-425O23.2	ENSG00000205041	1462	-0.45691	0.000298	0.008696
RP11-855A2.3	ENSG00000266717	709	-0.45658	0.00089	0.016015
RP11-1250I15.1	ENSG00000279995	224	-0.45654	0.002236	0.027677
RP11-21L23.2	ENSG00000261578	4192	-0.45614	0.005296	0.047087
RP3-395M20.7	ENSG00000225931	1297	-0.45575	0.00026	0.008111
PLEC	ENSG00000178209	16991	-0.45562	0.001121	0.018442
RPL23AP64	ENSG00000240970	435	-0.45555	7.1E-06	0.001129
CAMK2A	ENSG00000070808	6150	-0.45555	0.003725	0.037892
SMIM5	ENSG00000204323	5117	-0.45548	0.00477	0.043956
RP11-78F17.1	ENSG00000263551	1356	-0.45529	0.000273	0.00834
RP11-354E23.4	ENSG00000279502	619	-0.45503	0.000176	0.006546
TDGF1P1	ENSG00000227988	527	-0.45393	0.005435	0.047734
RP11-181G12.2	ENSG00000182873	1734	-0.45324	0.004006	0.039655
IRS1	ENSG00000169047	9953	-0.45291	6.23E-05	0.003815
XPNPEP2	ENSG00000122121	3500	-0.45282	0.000882	0.015928
RP11-339B21.13	ENSG00000272696	573	-0.45232	0.000122	0.005346
RN7SL220P	ENSG00000266467	300	-0.45197	0.001657	0.023119

GAPDHP51	ENSG00000236973	1000	-0.45163	0.002889	0.032374
RP11-286N3.2	ENSG00000264000	456	-0.45147	0.001174	0.018886
AC092755.4	ENSG00000227161	97	-0.45131	0.004767	0.043956
CTD-2283N19.1	ENSG00000251330	514	-0.45093	0.00113	0.018506
AC132812.1	ENSG00000274483	101	-0.45053	0.000224	0.007494
RP11-284F21.10	ENSG00000272405	3222	-0.45027	2.82E-06	0.000664
CTA-797E19.1	ENSG00000254484	422	-0.45014	0.004368	0.041746
RP13-128O4.3	ENSG00000250995	1062	-0.44991	0.000182	0.006639
CTA-228A9.3	ENSG00000272720	1433	-0.4499	0.000182	0.006639
RP1-253P7.4	ENSG00000197815	1815	-0.44986	9.71E-05	0.004879
NCKAP5-IT1	ENSG00000232474	554	-0.44919	0.005637	0.048698
LNX1-AS1	ENSG00000250930	1381	-0.44859	0.000901	0.016151
AC016909.1	ENSG00000233729	1711	-0.44831	0.002745	0.031391
TDGF1P6	ENSG00000241438	448	-0.44829	0.003726	0.037892
RP11-230B22.1	ENSG00000235545	1228	-0.44751	0.001728	0.023706
ALS2CL	ENSG00000178038	9057	-0.44724	0.00293	0.03267
LINC01001	ENSG00000230724	3823	-0.447	0.000514	0.011831
RP11-19G24.1	ENSG00000274308	814	-0.44699	0.004463	0.042328
SNORA76	ENSG00000252904	137	-0.44661	0.002044	0.026409
CTD-2017D11.2	ENSG00000279425	2189	-0.44656	0.000774	0.014787
RP11-391H12.8	ENSG00000276434	509	-0.44646	0.001275	0.019712
MTND2P16	ENSG00000230818	799	-0.44639	0.004752	0.043908
CTA-305I2.1	ENSG00000261535	801	-0.44635	0.001504	0.021793
RP11-82O19.1	ENSG00000263657	576	-0.44603	0.000122	0.005346
RP11-360N9.3	ENSG00000279361	1200	-0.44583	0.001817	0.024449
RP11-350G8.5	ENSG00000228013	1756	-0.44582	0.00512	0.046083
RP11-533K9.4	ENSG00000278486	643	-0.44562	0.005379	0.04747
PER1	ENSG00000179094	7002	-0.44556	0.00496	0.045081
RP11-575M22.1	ENSG00000279512	2049	-0.4452	0.005358	0.047356
MIR4523	ENSG00000263719	69	-0.44487	0.002089	0.026712
RPS2P41	ENSG00000244060	603	-0.44469	0.003233	0.034723
RN7SKP54	ENSG00000222636	269	-0.44466	0.000753	0.014543
AC002128.5	ENSG00000271366	618	-0.44455	3.69E-06	0.000781
RP11-274B21.4	ENSG00000230715	403	-0.44421	0.000518	0.011906
RP11-274A11.4	ENSG00000279841	1846	-0.44379	0.000206	0.007163
RP11-259F16.3	ENSG00000233163	377	-0.44376	0.002831	0.032079
AC073326.3	ENSG00000228540	557	-0.44331	0.001121	0.018442
CTD-2288F12.1	ENSG00000260352	463	-0.44302	0.002223	0.027667
CTB-129O4.1	ENSG00000248367	393	-0.44243	0.005668	0.048872
NEAT1_3	ENSG00000277599	159	-0.44239	0.00036	0.009633
SPDYE5	ENSG00000170092	3325	-0.44225	0.000807	0.015185

ILF2P1	ENSG00000244226	1168	-0.44187	0.000454	0.010981
DOHH	ENSG00000129932	2387	-0.44147	0.001323	0.020206
RN7SL57P	ENSG00000242256	290	-0.44142	0.002451	0.029245
GS1-115G20.2	ENSG00000232036	1325	-0.44089	0.003254	0.034852
CTC-435M10.12	ENSG00000277744	449	-0.44056	0.002878	0.032351
NEAT1	ENSG00000245532	22767	-0.44053	0.002191	0.027418
RP1-128O3.5	ENSG00000233511	187	-0.44051	0.001955	0.025466
RP11-504I13.2	ENSG00000280205	3765	-0.44032	0.001324	0.020206
RP11-159F24.6	ENSG00000248554	572	-0.43997	0.001412	0.021006
CTC-786C10.1	ENSG00000262601	777	-0.43989	0.002608	0.030428
AL049794.1	ENSG00000280240	1864	-0.43955	0.00225	0.02778
TBX3	ENSG00000135111	5950	-0.4392	0.000819	0.015231
AC114730.8	ENSG00000215692	472	-0.43898	0.005678	0.048919
RP11-452H21.2	ENSG00000254649	365	-0.43855	0.002366	0.028681
CTD-2262B20.1	ENSG00000259630	406	-0.43746	0.000187	0.006737
TDRD12	ENSG00000173809	7011	-0.43745	0.003391	0.035884
RP11-354E23.3	ENSG00000280166	619	-0.43683	0.000771	0.014757
LRRN4	ENSG00000125872	2692	-0.43679	0.003881	0.038794
FKBP1AP2	ENSG00000259691	283	-0.43615	0.003056	0.033645
TNRC18	ENSG00000182095	12572	-0.43585	3.11E-05	0.002705
RP11-336K24.12	ENSG00000273002	2157	-0.4356	0.00039	0.010071
RP11-84D1.1	ENSG00000229162	820	-0.43473	0.000539	0.012135
RP11-757C15.4	ENSG00000255296	560	-0.43358	0.005485	0.047931
RP4-678D15.1	ENSG00000271774	724	-0.43343	0.005134	0.046148
RP11-552C15.1	ENSG00000260601	520	-0.43292	0.003269	0.034935
AL590762.11	ENSG00000213018	432	-0.43242	0.002524	0.029834
RP11-503N18.5	ENSG00000251229	900	-0.43228	0.00081	0.015195
RP4-752I6.1	ENSG00000241169	428	-0.43223	0.000611	0.013009
Z84812.4	ENSG00000260803	773	-0.4321	0.000608	0.01298
MIR6838	ENSG00000277792	56	-0.43167	0.001938	0.025399
RN7SL23P	ENSG00000240823	275	-0.43121	0.002905	0.032472
CXCR5	ENSG00000160683	4431	-0.4312	8.29E-05	0.004484
LINC00598	ENSG00000215483	2489	-0.4311	0.000658	0.013581
RP11-525G13.2	ENSG00000236364	675	-0.43062	0.000441	0.01077
RP11-1102P22.2	ENSG00000279620	1722	-0.43056	0.002143	0.027085
MIDN	ENSG00000167470	4492	-0.43044	3.77E-05	0.002954
RP11-98L12.2	ENSG00000258578	465	-0.43006	0.005291	0.047073
RP1-59D14.1	ENSG00000262456	1065	-0.42997	3.24E-05	0.00276
RP11-332O19.2	ENSG00000237827	400	-0.42996	0.001326	0.020227
SPACA4	ENSG00000177202	972	-0.42989	0.0037	0.037765
RP11-689C9.1	ENSG00000272461	657	-0.42972	0.001523	0.021932

RP11-242C19.2	ENSG00000241886	562	-0.42959	0.002358	0.028628
RP11-493E12.2	ENSG00000273302	791	-0.42926	0.000156	0.006081
CTD-2561B21.8	ENSG00000280351	1798	-0.42884	0.000979	0.016923
RPL13P6	ENSG00000213176	626	-0.42869	0.004576	0.042832
RP11-158H5.8	ENSG00000274184	2021	-0.42861	0.001522	0.021932
AC007389.3	ENSG00000235725	1656	-0.42853	0.000647	0.013481
ZNF587P1	ENSG00000223965	1386	-0.42812	0.003061	0.033666
TCL6	ENSG00000187621	10566	-0.42778	6.9E-06	0.001124
AC127904.2	ENSG00000230732	1109	-0.4274	0.000144	0.005789
RN7SL535P	ENSG00000239419	296	-0.42706	0.001361	0.020437
IGFBP4	ENSG00000141753	2200	-0.42692	0.000472	0.011228
NPIPA3	ENSG00000224712	2188	-0.42678	0.004059	0.03995
CTD-2154B17.4	ENSG00000272324	377	-0.42675	4.3E-05	0.003163
RP11-47A8.5	ENSG00000272933	1349	-0.42636	2.16E-05	0.002118
RNU6-82P	ENSG00000200840	107	-0.42632	0.003308	0.035237
CTD-3220F14.2	ENSG00000267682	375	-0.42619	0.000673	0.013708
DNM1P41	ENSG00000280038	3350	-0.42606	0.000125	0.005449
MIR3679	ENSG00000263813	68	-0.42513	0.000308	0.008832
EIF4A1P11	ENSG00000231564	222	-0.42488	0.001444	0.021278
XIAP-AS1	ENSG00000237331	482	-0.42484	0.001091	0.018163
LRP4	ENSG00000134569	8597	-0.42476	0.000127	0.005505
FAR1-IT1	ENSG00000254791	368	-0.4238	0.000316	0.008954
RP11-57H14.5	ENSG00000279315	1286	-0.42348	0.004406	0.042036
RP1-197B17.6	ENSG00000277874	1343	-0.42338	0.002255	0.027817
AC137934.1	ENSG00000260923	3154	-0.42325	0.000136	0.005622
RP11-214J9.1	ENSG00000235440	820	-0.42223	0.000248	0.007941
ITPK1-AS1	ENSG00000258730	4701	-0.42214	0.000635	0.013325
RP11-154B12.3	ENSG00000259177	458	-0.42213	0.001952	0.025456
YRDCP2	ENSG00000236745	642	-0.4221	0.003832	0.0385
RPL31P58	ENSG00000243053	379	-0.42204	0.003171	0.034387
HNRNPA1P17	ENSG00000243188	924	-0.4215	0.003047	0.033583
RP11-545N8.3	ENSG00000259125	879	-0.42146	5.15E-05	0.003529
TUBBP10	ENSG00000226147	1301	-0.42139	0.001764	0.024003
RP11-236P24.3	ENSG00000234019	615	-0.42133	0.002158	0.027203
RP11-16C1.1	ENSG00000265342	372	-0.42038	0.005312	0.047152
RP11-660M5.1	ENSG00000271172	511	-0.42026	0.004467	0.042347
MT-TT	ENSG00000210195	66	-0.42026	0.000693	0.013899
RP11-166P13.4	ENSG00000263089	4281	-0.42024	6.26E-05	0.003815
NEAT1_2	ENSG00000278050	105	-0.41963	0.000854	0.015591
MT-TY	ENSG00000210144	66	-0.41902	0.000309	0.008834
EGLN3P1	ENSG00000256134	720	-0.41888	0.003829	0.038493

CTD-2013N17.6	ENSG00000280332	1999	-0.41871	0.00041	0.010339
ULK1	ENSG00000177169	7608	-0.41841	0.000165	0.006251
OMP	ENSG00000254550	492	-0.41823	0.005083	0.045871
RP11-288E14.2	ENSG00000234106	349	-0.41777	0.000654	0.013555
NODAL	ENSG00000156574	1693	-0.41764	0.002367	0.028682
RP11-174G6.1	ENSG00000214772	657	-0.41752	0.000125	0.005421
DIP2A-IT1	ENSG00000223692	428	-0.4175	0.001195	0.019056
RPL12P44	ENSG00000227123	502	-0.41713	0.001941	0.025399
RP11-45A17.2	ENSG00000277959	332	-0.41707	0.002203	0.027516
FTH1P4	ENSG00000242992	553	-0.41706	0.000597	0.012861
RP11-342M21.2	ENSG00000259202	472	-0.41647	0.004469	0.042353
TLE1P1	ENSG00000228158	589	-0.41645	0.005116	0.046071
LTBP4	ENSG00000090006	8108	-0.41611	6.69E-05	0.003996
RP11-1250I15.2	ENSG00000278900	587	-0.41603	0.005599	0.048489
SNORD39	ENSG00000264379	76	-0.41582	0.001264	0.019589
BACH1-AS1	ENSG00000232118	879	-0.41547	9.38E-05	0.004783
RP11-703G6.1	ENSG00000249614	812	-0.41522	1.77E-05	0.001906
IGF2BP2-AS1	ENSG00000163915	3415	-0.41381	0.001148	0.018667
RP11-266L9.6	ENSG00000263326	676	-0.41381	0.004952	0.045037
RP11-936I5.1	ENSG00000266998	3419	-0.41317	0.000971	0.016885
PFN1P7	ENSG00000227212	399	-0.41266	0.003031	0.033442
ARHGAP26-IT1	ENSG00000230789	388	-0.41263	0.00237	0.0287
CTA-243E7.4	ENSG00000279085	5388	-0.41261	0.000368	0.009769
AC073333.1	ENSG00000280130	381	-0.41257	0.000104	0.005013
LARGE-AS1	ENSG00000224973	2929	-0.41251	0.002275	0.02792
RP11-666A20.4	ENSG00000248787	427	-0.41214	0.001388	0.020712
RP11-933H2.4	ENSG00000250608	4200	-0.41212	0.000142	0.005755
POU5F1P5	ENSG00000236375	658	-0.41209	0.002058	0.026507
RP5-1158E12.3	ENSG00000231566	375	-0.41172	0.002821	0.03202
MNT	ENSG00000070444	8728	-0.41114	3.93E-06	0.000804
CTC-251D13.1	ENSG00000271795	3317	-0.41076	0.005254	0.046884
RP11-312B8.2	ENSG00000227950	286	-0.41037	0.003167	0.034378
RP11-67A1.4	ENSG00000279621	1141	-0.40988	0.000128	0.005521
RP11-466A19.8	ENSG00000265222	927	-0.40974	0.000665	0.013659
RP11-108M12.3	ENSG00000258592	1577	-0.40903	0.001837	0.024626
RP11-81A1.4	ENSG00000279394	2260	-0.40893	0.001554	0.022162
AC064852.5	ENSG00000213055	676	-0.40823	0.003243	0.034763
KLF3P1	ENSG00000253218	1022	-0.40818	0.001843	0.024678
AP000253.1	ENSG00000234509	2233	-0.40767	0.002184	0.027382
RP11-697K23.3	ENSG00000279017	1025	-0.40757	0.004784	0.044011
SH3D21	ENSG00000214193	4474	-0.40738	0.00086	0.015677

RP11-298J20.3	ENSG00000226899	830	-0.40725	0.000667	0.013662
RP11-507K2.6	ENSG00000278576	3178	-0.4071	0.000109	0.005083
RBPMSLP	ENSG00000234159	502	-0.40671	0.001164	0.018824
RP11-96D1.9	ENSG00000262514	1187	-0.40627	0.000129	0.005521
TPT1P10	ENSG00000180221	496	-0.40551	0.00185	0.024726
LA16c-361A3.3	ENSG00000261207	565	-0.40499	0.00378	0.038253
DAPK1-IT1	ENSG00000236709	702	-0.40484	0.005294	0.047084
AC068039.4	ENSG00000228389	2102	-0.40484	1.84E-05	0.001937
CH507-528H12.1	ENSG00000280441	2197	-0.40461	0.005446	0.047752
C1orf233	ENSG00000228594	2086	-0.40391	0.005209	0.046622
AUTS2	ENSG00000158321	10420	-0.40337	0.000241	0.007839
BCL3	ENSG00000069399	3242	-0.40327	0.00013	0.005521
WARS2-IT1	ENSG00000224238	2060	-0.40327	0.000549	0.012232
RP11-390K5.6	ENSG00000270060	574	-0.40323	2.14E-05	0.00211
RPL7L1P2	ENSG00000231490	739	-0.40313	0.004579	0.042832
GTF2IP1	ENSG00000277053	7453	-0.40282	1.87E-05	0.00195
YWHAQP5	ENSG00000236564	1382	-0.40248	0.000111	0.00512
EID3	ENSG00000255150	1464	-0.4023	0.002842	0.032143
RP11-104N10.2	ENSG00000274627	5421	-0.40057	0.000289	0.0086
RP1-145M24.1	ENSG00000254708	886	-0.40011	0.004315	0.041449
LL0XNC01-36H8.1	ENSG00000279155	817	-0.39944	0.00079	0.015025
MTND4P19	ENSG00000230338	478	-0.39936	0.000808	0.015185
ZNF865	ENSG00000261221	5275	-0.39888	0.000658	0.013582
RP11-108L7.14	ENSG00000273476	448	-0.39835	0.001201	0.0191
RP11-192H23.6	ENSG00000265287	1083	-0.39827	0.00083	0.015324
SLC6A7	ENSG00000011083	4391	-0.3982	0.001667	0.023196
CTD-2357A8.2	ENSG00000279570	714	-0.39814	0.000185	0.006705
FKSG56	ENSG00000279007	375	-0.39757	1.92E-05	0.00197
AJ271736.1	ENSG00000276543	68	-0.39744	0.003594	0.037149
RP11-138I1.3	ENSG00000266651	304	-0.39725	4.16E-05	0.003099
RP11-304F15.7	ENSG00000262837	416	-0.39685	0.001786	0.02418
SLC10A5	ENSG00000253598	2568	-0.39668	0.000451	0.010932
ZMYND19P1	ENSG00000258917	787	-0.39667	0.00414	0.040469
RP11-436H11.5	ENSG00000251456	558	-0.39661	0.003704	0.037778
RP11-52A20.2	ENSG00000272033	1893	-0.39647	0.00223	0.027667
HNRNPA1P35	ENSG00000225695	963	-0.39585	0.004288	0.041337
RP4-635A23.4	ENSG00000238061	973	-0.3958	0.000504	0.011747
CCDC17	ENSG00000159588	3323	-0.3958	0.000332	0.009256
RP1-122P22.2	ENSG00000233895	1089	-0.39535	2.86E-06	0.000668
AC004951.6	ENSG00000228434	1866	-0.39533	0.001258	0.019567
RP11-326K13.5	ENSG00000274275	325	-0.3949	0.004866	0.044482

FLJ42393	ENSG00000279891	2270	-0.39486	4.44E-05	0.003209
SLC9A1	ENSG00000090020	5974	-0.3948	0.001033	0.017454
RP13-638C3.2	ENSG00000262652	594	-0.39408	0.003402	0.035952
PRRT2	ENSG00000167371	3318	-0.39349	0.000335	0.009298
SPTLC3	ENSG00000172296	8520	-0.3934	0.00027	0.008267
RP11-709D24.6	ENSG00000261442	1543	-0.39245	0.001479	0.021614
MIR6773	ENSG00000276151	74	-0.39233	0.003841	0.038553
SLC6A20	ENSG00000163817	6651	-0.39194	0.001064	0.017885
RP11-243J16.7	ENSG00000236559	374	-0.39189	0.00096	0.016775
RPL7P14	ENSG00000232994	741	-0.39187	0.002028	0.026273
HMGN1P8	ENSG00000241120	288	-0.39134	8.64E-05	0.004578
RP11-314D7.2	ENSG00000258123	1256	-0.39126	7.75E-05	0.004361
ERVH-1	ENSG00000251292	639	-0.39119	0.002876	0.032351
RP11-54A4.2	ENSG00000237781	665	-0.39062	0.002478	0.029474
RP11-466A19.3	ENSG00000266599	408	-0.39059	9.97E-05	0.004942
MAGI1-IT1	ENSG00000272610	2567	-0.39036	0.001521	0.021932
RP11-46I1.2	ENSG00000257864	573	-0.39027	0.005442	0.047752
RP11-76G10.1	ENSG00000250354	680	-0.39025	0.004784	0.044011
RP11-44D5.2	ENSG00000279465	660	-0.39016	0.004947	0.04503
MPRIPP1	ENSG00000214820	1089	-0.38961	0.00011	0.005099
LINC00893	ENSG00000241769	3604	-0.38948	5.63E-05	0.003692
RP11-307C18.1	ENSG00000272950	578	-0.38916	0.004752	0.043908
AC013275.2	ENSG00000231013	2089	-0.38871	0.004371	0.041753
AC004813.1	ENSG00000279777	375	-0.38835	0.000624	0.013182
MT-TC	ENSG00000210140	66	-0.38769	0.000937	0.016547
ARHGEF26-AS1	ENSG00000243069	5998	-0.38758	7.38E-05	0.004224
BCORL1	ENSG00000085185	7996	-0.38756	8.51E-05	0.004546
ABTB1	ENSG00000114626	6149	-0.38697	0.000198	0.006983
KDM6B	ENSG00000132510	7258	-0.38608	0.000233	0.007689
RP3-525L6.2	ENSG00000220920	359	-0.38582	2.22E-05	0.002133
UPK3BP1	ENSG00000242435	509	-0.38542	0.00568	0.048919
CDC42BPG	ENSG00000171219	6112	-0.38467	0.000802	0.015136
RP11-8P13.5	ENSG00000274976	426	-0.38452	0.003005	0.033226
AGRN	ENSG00000188157	10758	-0.3844	0.004031	0.03982
CTB-25B13.9	ENSG00000267092	457	-0.38425	0.004556	0.042762
NRTN	ENSG00000171119	1109	-0.38397	0.003436	0.036161
FOSL1P1	ENSG00000248394	816	-0.38391	0.002618	0.030523
FAM229A	ENSG00000225828	2845	-0.3839	0.000229	0.007611
UPK2	ENSG00000110375	1106	-0.3838	0.001391	0.020743
RP11-80P20.3	ENSG00000280399	3691	-0.38361	0.005759	0.049297
ITGB4	ENSG00000132470	6761	-0.38336	0.002184	0.027382

MBD6	ENSG00000166987	5646	-0.38335	3.74E-05	0.002942
AGAP4	ENSG00000188234	3474	-0.38329	2.91E-05	0.002574
RP11-474I11.8	ENSG00000264829	352	-0.38282	0.00405	0.039927
CCT8P1	ENSG00000226015	1657	-0.38201	0.002712	0.031279
RP4-758J24.6	ENSG00000280378	1755	-0.3819	0.000581	0.012703
RP13-554M15.7	ENSG00000280287	4219	-0.38179	0.001293	0.019886
CATSPER2	ENSG00000166762	8403	-0.38174	0.002517	0.029831
CDC42P1	ENSG00000234933	548	-0.38163	0.001498	0.021728
RP11-530C5.2	ENSG00000258044	667	-0.38132	0.000983	0.016953
RP11-484D2.4	ENSG00000254577	183	-0.3813	0.000969	0.016885
DUSP8P5	ENSG00000235316	1815	-0.38104	0.001038	0.017521
RP11-342K2.1	ENSG00000279133	3819	-0.3809	0.000105	0.005013
AIG1P1	ENSG00000249089	148	-0.3807	0.001549	0.022148
RP11-642A1.1	ENSG00000280326	1518	-0.38054	0.003635	0.037414
RP4-740C4.5	ENSG00000269896	2407	-0.38037	0.000669	0.013666
MDN1	ENSG00000112159	18597	-0.38017	0.00396	0.039361
AJ271736.10	ENSG00000270726	773	-0.37987	0.001709	0.02356
H2AFZP3	ENSG00000218502	387	-0.37972	0.0032	0.034527
RP11-506K6.4	ENSG00000272320	1989	-0.37969	0.000579	0.012684
RP11-17G12.3	ENSG00000256746	536	-0.37958	0.000203	0.007139
RP11-511H9.3	ENSG00000258230	642	-0.37957	0.001762	0.024001
SCOCP1	ENSG00000258896	349	-0.37934	0.004987	0.045238
SEMA4B	ENSG00000185033	7006	-0.37922	0.000928	0.016487
RP11-49O14.3	ENSG00000230815	424	-0.37902	0.004268	0.041215
RP11-20I23.8	ENSG00000269937	3626	-0.37878	6.69E-05	0.003996
RP11-571F15.2	ENSG00000227383	327	-0.3786	0.003619	0.037298
ABTB2	ENSG00000166016	4973	-0.37853	0.001336	0.020271
SGK223	ENSG00000275342	4904	-0.37819	0.000629	0.013228
HHLA1	ENSG00000132297	6143	-0.37785	0.001738	0.023815
RP11-855A2.1	ENSG00000279573	1978	-0.37749	0.000533	0.01207
RP11-318K15.2	ENSG00000254325	673	-0.3774	0.001419	0.021063
AL450226.2	ENSG00000225084	710	-0.37655	0.005815	0.049591
WIPF3	ENSG00000122574	6065	-0.37549	0.003515	0.036726
RP11-505K9.5	ENSG00000274677	612	-0.37537	0.000376	0.009902
MTND4P20	ENSG00000233377	869	-0.37513	0.001209	0.019167
DCUN1D2-AS	ENSG00000233613	373	-0.3751	0.00368	0.037651
RP11-391L3.3	ENSG00000279476	2794	-0.37477	0.005054	0.045667
RP11-71L14.3	ENSG00000279693	2938	-0.37477	0.005263	0.046916
RN7SL657P	ENSG00000264293	293	-0.37443	0.003517	0.036726
RP4-569M23.4	ENSG00000273451	1006	-0.37434	0.002823	0.03202
KAZN	ENSG00000189337	12644	-0.37423	3.55E-06	0.000768

DUOX1	ENSG00000137857	8784	-0.37413	0.000111	0.00512
BAI1	ENSG00000181790	7356	-0.37403	0.005487	0.047931
RP11-888D10.4	ENSG00000273284	1003	-0.37394	0.001495	0.021726
LRP4-AS1	ENSG00000247675	1614	-0.3736	0.003262	0.034894
GRID2IP	ENSG00000215045	4134	-0.37339	0.003632	0.037412
RNU6-1212P	ENSG00000252832	104	-0.37332	0.003905	0.038995
SPINK9	ENSG00000204909	841	-0.37301	0.00022	0.007419
AC069063.1	ENSG00000279131	66	-0.37289	0.00402	0.039752
GATSL2	ENSG00000274070	1363	-0.37259	0.001727	0.023704
RP11-1136G11.8	ENSG00000257808	481	-0.37211	0.001676	0.023278
RP11-681L8.1	ENSG00000254044	770	-0.37203	0.004192	0.040763
RP11-274B21.3	ENSG00000243679	754	-0.37175	0.00441	0.042036
HMGN2P35	ENSG00000232875	270	-0.37128	0.000261	0.008127
AGRP	ENSG00000159723	764	-0.37054	0.003097	0.033909
AC002066.1	ENSG00000237813	1213	-0.37023	0.003192	0.034477
RNU6-1016P	ENSG00000252498	94	-0.36997	0.003921	0.039095
RP11-49I11.4	ENSG00000274849	449	-0.36986	0.000433	0.01064
RP11-1055B8.4	ENSG00000262877	1565	-0.36971	0.000497	0.011616
RP5-1184F4.5	ENSG00000236772	986	-0.36945	9.98E-05	0.004942
FBRSL1	ENSG00000112787	9311	-0.36919	0.000355	0.00956
RPL35P3	ENSG00000219902	371	-0.36914	0.000429	0.010594
SLC17A4	ENSG00000146039	4633	-0.36908	0.003612	0.037239
DGKD	ENSG00000077044	9771	-0.36896	1.57E-05	0.001763
RP11-474D14.2	ENSG00000226318	763	-0.36859	0.003215	0.034651
SAP25	ENSG00000205307	1388	-0.36824	0.003174	0.034393
RP11-459F6.3	ENSG00000260927	577	-0.36806	0.003674	0.037619
RP4-614O4.13	ENSG00000279253	1461	-0.36798	0.000136	0.005622
ZFPM1	ENSG00000179588	6110	-0.36766	0.000656	0.013567
MICALCL	ENSG00000133808	3448	-0.36754	0.000578	0.01268
MICALL2	ENSG00000164877	8843	-0.36743	0.001898	0.025099
PRSS48	ENSG00000189099	989	-0.36698	0.001644	0.023003
RP11-218E20.3	ENSG00000258711	4714	-0.36696	0.00162	0.022793
RP11-368N21.5	ENSG00000279583	1008	-0.36667	0.000941	0.016589
NUTM2G	ENSG00000188152	3906	-0.36647	0.000289	0.0086
RP11-391L3.4	ENSG00000279021	968	-0.36637	0.000823	0.015262
RP11-166N17.3	ENSG00000226842	466	-0.36637	0.000449	0.010901
FBXW4P1	ENSG00000230701	2239	-0.36632	0.004107	0.040231
TPM3P7	ENSG00000187536	732	-0.36584	0.002097	0.026732
UBE2V2P1	ENSG00000226255	398	-0.3658	0.004117	0.040306
RP3-508I15.21	ENSG00000272669	491	-0.36573	0.001346	0.020366
PPP5D1	ENSG00000230510	5703	-0.36569	0.000693	0.013899

RP11-649A18.4	ENSG00000263786	717	-0.36543	0.002718	0.031326
MIR1260B	ENSG00000266192	108	-0.36541	0.004343	0.041581
MAST3	ENSG00000099308	6772	-0.36528	0.000206	0.007163
ENO1-IT1	ENSG00000236269	744	-0.36524	0.001993	0.025885
YPEL3	ENSG00000090238	3181	-0.3648	0.000422	0.010497
SMIM18	ENSG00000253457	905	-0.36467	0.001641	0.022974
SPDYE1	ENSG00000136206	2649	-0.36425	0.001319	0.020157
MIR6859-1	ENSG00000278739	68	-0.3642	0.000795	0.015076
RP11-39K24.12	ENSG00000228097	670	-0.36418	0.004862	0.044458
RP11-635N19.2	ENSG00000267430	1153	-0.36382	0.000104	0.005013
CBX5P1	ENSG00000241535	561	-0.36361	0.000431	0.010606
RP11-158H5.2	ENSG00000279989	473	-0.3635	0.000701	0.013971
MTND4P15	ENSG00000227321	1033	-0.36323	0.002435	0.029154
HMGA1P2	ENSG00000248641	324	-0.3632	0.000127	0.005505
MXRA8	ENSG00000162576	3622	-0.3632	0.001925	0.02527
AL132772.1	ENSG00000265893	90	-0.36314	0.003668	0.037619
TSSK3	ENSG00000162526	2191	-0.36306	0.000322	0.009053
RASGRF2-AS1	ENSG00000251450	1166	-0.36282	0.004973	0.045169
RP1-86D1.3	ENSG00000279776	1904	-0.36268	0.000358	0.009626
RP11-449G16.1	ENSG00000273035	477	-0.36259	0.000814	0.01523
FAM83H	ENSG00000180921	5654	-0.36214	0.000742	0.014446
RHOBTB2	ENSG00000008853	6580	-0.3621	0.00554	0.048173
LINC00504	ENSG00000248360	5095	-0.36201	8.25E-05	0.004483
RN7SL834P	ENSG00000243650	286	-0.36185	0.001539	0.022097
RP11-1079K10.3	ENSG00000248714	881	-0.36163	0.002409	0.028944
AC007050.18	ENSG00000250261	1123	-0.36121	0.001256	0.019567
RP11-165N19.2	ENSG00000225513	450	-0.36119	0.001103	0.018281
EPN1	ENSG00000063245	17616	-0.36103	0.000553	0.012276
CACNA1H	ENSG00000196557	8484	-0.36089	0.005523	0.048089
KIAA1522	ENSG00000162522	5965	-0.36062	0.000277	0.008378
INF2	ENSG00000203485	9488	-0.35992	0.003202	0.034537
IFITM10	ENSG00000244242	4784	-0.35975	0.005019	0.045441
AP003068.23	ENSG00000254614	1791	-0.3597	0.003116	0.034021
HMGB3P4	ENSG00000228808	514	-0.35953	0.002051	0.026467
RP11-231E4.5	ENSG00000276934	483	-0.35921	0.004754	0.043913
MRGPRX3	ENSG00000179826	1741	-0.359	0.004532	0.042609
AC019070.1	ENSG00000224007	1024	-0.35865	0.002727	0.031391
AC091132.1	ENSG00000236234	769	-0.35862	0.004776	0.043997
AC011330.13	ENSG00000275601	635	-0.3581	0.001801	0.024312
RECQL5	ENSG00000108469	8766	-0.35798	0.002623	0.030539
ADCY9	ENSG00000162104	10358	-0.35736	0.001723	0.023704

C1orf220	ENSG00000213057	2775	-0.35725	0.000527	0.012001
RP11-881L2.1	ENSG00000266541	534	-0.35697	0.001193	0.019048
ATN1	ENSG00000111676	4762	-0.35684	0.000537	0.012129
ZNF579	ENSG00000218891	3321	-0.35677	0.001272	0.019699
ZNF296	ENSG00000170684	1744	-0.35667	0.000353	0.009555
CTD-2373N4.5	ENSG00000253112	407	-0.35665	0.002203	0.027516
BCYRN1	ENSG00000236824	13458	-0.35595	7.57E-05	0.004295
EEF1A1P25	ENSG00000241429	1267	-0.3553	0.005188	0.046555
DOT1L	ENSG00000104885	11272	-0.35528	0.000627	0.013207
CCDC162P	ENSG00000203799	5378	-0.35477	0.000669	0.013662
RNF43	ENSG00000108375	7505	-0.35476	0.00083	0.015324
RNF125	ENSG00000101695	6508	-0.35473	3.23E-05	0.00276
RP11-39K24.13	ENSG00000237711	774	-0.35379	0.003876	0.03878
CTC-277H1.6	ENSG00000280163	1675	-0.35361	0.002624	0.030539
CTC-260E6.6	ENSG00000267383	3684	-0.3531	0.001112	0.018381
ZMIZ1	ENSG00000108175	11048	-0.35302	7.83E-05	0.00438
ZBTB7A	ENSG00000178951	5725	-0.35276	5.68E-07	0.000235
AC004987.9	ENSG00000233225	1148	-0.35219	0.004905	0.044734
CTC-436K13.3	ENSG00000253424	548	-0.35218	0.000878	0.015873
HNRNPA1P14	ENSG00000227638	935	-0.35195	0.001409	0.020976
EPPK1	ENSG00000261150	16002	-0.35125	0.002846	0.032169
RP5-849H19.2	ENSG00000213703	546	-0.35097	0.002305	0.028169
RP1-102K2.8	ENSG00000268812	794	-0.35043	0.000466	0.011131
RP4-734G22.3	ENSG00000272078	7923	-0.35004	0.002319	0.028271
RN7SL517P	ENSG00000241939	305	-0.3499	0.004497	0.042497
SP2	ENSG00000167182	3184	-0.3497	0.000218	0.007397
RP11-17A4.2	ENSG00000254254	3078	-0.34954	0.000663	0.013646
RP11-665C16.1	ENSG00000239686	348	-0.3492	0.004306	0.041419
YJEFN3	ENSG00000250067	2959	-0.34918	9.22E-05	0.004764
RP11-521B24.3	ENSG00000251602	2279	-0.34904	0.004256	0.04119
SH3GL1P1	ENSG00000266777	2855	-0.34894	0.002811	0.031988
RP3-323A16.1	ENSG00000279184	54079	-0.34884	0.000122	0.005346
PPP1R13L	ENSG00000104881	5032	-0.34851	0.0006	0.012876
TYRO3P	ENSG00000259581	2705	-0.34848	0.001797	0.024274
RP11-34A14.3	ENSG00000230928	1168	-0.34833	0.001232	0.019407
ABC12-49244600F4.4	ENSG00000276144	751	-0.34826	0.002229	0.027667
RP11-504P24.8	ENSG00000272645	2052	-0.34815	0.000547	0.012204
AC003976.2	ENSG00000274808	3014	-0.3479	0.004509	0.042507
AC093620.5	ENSG00000241269	583	-0.34772	0.005633	0.048677
AC004471.9	ENSG00000223461	582	-0.34766	0.004159	0.040586

ZDHHC14	ENSG00000175048	11121	-0.34765	5.43E-06	0.000971
NNAT	ENSG00000053438	1297	-0.34756	0.001563	0.022273
TCF3P1	ENSG00000236567	1937	-0.34746	0.005576	0.048363
RP11-392O18.2	ENSG00000271711	616	-0.34739	0.001133	0.018518
CEP170B	ENSG00000099814	7199	-0.34736	0.001234	0.019408
RP11-302F12.10	ENSG00000250541	483	-0.34726	0.002388	0.028791
RP11-293A21.2	ENSG00000248340	743	-0.34709	0.0032	0.034527
AC079807.3	ENSG00000236549	455	-0.3469	0.002025	0.026258
CTD-2583A14.8	ENSG00000269867	679	-0.34654	0.000479	0.011314
RP11-894P9.1	ENSG00000246451	1404	-0.34618	2.23E-05	0.002133
ARHGAP39	ENSG00000147799	4888	-0.34596	0.000463	0.011105
LTB4R	ENSG00000213903	4465	-0.34595	0.000238	0.007776
RGS17P1	ENSG00000229473	553	-0.3454	0.000183	0.006688
AC096574.4	ENSG00000225057	3890	-0.34534	0.000577	0.012663
RP11-396K3.1	ENSG00000233369	3609	-0.3453	0.004813	0.044162
RLIMP1	ENSG00000229456	1680	-0.34528	0.000357	0.009607
AC138035.2	ENSG00000238035	1838	-0.34504	0.00091	0.016287
FAM35CP	ENSG00000259096	2470	-0.34472	0.000433	0.010645
RP11-843P14.1	ENSG00000251414	528	-0.34468	0.002882	0.032371
SEMA4G	ENSG00000095539	5253	-0.3445	3.64E-05	0.002906
KRT18P4	ENSG00000229222	1288	-0.34413	0.001942	0.025399
RP11-329N15.3	ENSG00000279320	1991	-0.344	0.004175	0.040678
RP11-775J23.2	ENSG00000243508	713	-0.34399	0.000143	0.005758
SRRM2	ENSG00000167978	13266	-0.34389	0.00016	0.006121
SYNPO	ENSG00000171992	10258	-0.34386	0.000767	0.014715
RP11-461A8.1	ENSG00000261938	329	-0.34363	0.002739	0.031391
SOX12	ENSG00000177732	4824	-0.34338	9.31E-05	0.004774
ARHGAP32	ENSG00000134909	12841	-0.34313	1.59E-06	0.000483
AL442127.2	ENSG00000278371	62	-0.34297	0.00123	0.019395
RP11-473O4.4	ENSG00000254263	467	-0.34209	0.00446	0.042322
FLJ22447	ENSG00000232774	2653	-0.34208	6.02E-05	0.003803
MT-CO1	ENSG00000198804	1542	-0.34158	0.000149	0.005876
MTND4P23	ENSG00000225796	1363	-0.34062	0.00525	0.046859
AC008993.2	ENSG00000279385	744	-0.34039	6.09E-05	0.003803
RP11-411B10.7	ENSG00000279352	864	-0.3402	0.000516	0.01187
SNORA70G	ENSG00000206650	142	-0.34006	0.003224	0.034694
RN7SL16P	ENSG00000243005	269	-0.34001	0.001175	0.018886
MIR181A2	ENSG00000207595	110	-0.33981	0.005192	0.046564
RP3-405J10.3	ENSG00000257298	4713	-0.33948	0.000761	0.014613
FAT1	ENSG00000083857	16177	-0.33913	0.000106	0.005029
RP11-421E14.2	ENSG00000264895	2170	-0.33892	0.004083	0.040098

MIR4743	ENSG00000266276	112	-0.33873	0.002068	0.026558
LENG8	ENSG00000167615	6469	-0.33812	0.000699	0.013945
HNRNPA3P9	ENSG00000270903	979	-0.33759	0.00575	0.04927
MEX3D	ENSG00000181588	2850	-0.33717	3.84E-05	0.002967
HMGB3P14	ENSG00000231845	599	-0.33685	0.000588	0.01278
TPM3P1	ENSG00000213050	747	-0.33666	0.004892	0.044625
RP11-562A8.1	ENSG00000242737	359	-0.33616	0.001223	0.019333
TTYH1	ENSG00000167614	7068	-0.33598	0.003054	0.033645
RP11-423E7.2	ENSG00000279862	1399	-0.33592	0.005212	0.04663
ATXN2L	ENSG00000168488	6508	-0.33566	3.91E-05	0.002999
DOCK5	ENSG00000147459	15960	-0.33559	2.9E-05	0.002574
RP11-780K2.1	ENSG00000257359	570	-0.33558	3.51E-05	0.002905
AC004754.3	ENSG00000269881	2242	-0.33556	0.000723	0.014245
HNF1A-AS1	ENSG00000241388	3859	-0.33553	0.001903	0.025119
RP11-823P9.4	ENSG00000279107	1161	-0.33546	0.001393	0.02077
RP11-39K24.4	ENSG00000224083	1528	-0.33493	0.005202	0.046594
MAF	ENSG00000178573	6976	-0.33489	0.001346	0.020366
CMIP	ENSG00000153815	12715	-0.33486	7.52E-06	0.001161
ZBTB20-AS4	ENSG00000242767	675	-0.3343	0.004314	0.041449
Z95152.1	ENSG00000221743	91	-0.33381	0.000268	0.00824
RP11-166B2.3	ENSG00000261560	1495	-0.33364	0.00068	0.013767
PLEKHM1P	ENSG00000214176	8469	-0.33359	0.000184	0.006694
RP11-731D1.1	ENSG00000249012	486	-0.33346	0.004476	0.042394
NDUFA6-AS1	ENSG00000237037	5206	-0.33346	0.000529	0.012008
ZNF483	ENSG00000173258	5738	-0.33338	0.004828	0.044254
MIR6723	ENSG00000237973	1543	-0.33331	0.003194	0.034488
RP11-284F21.7	ENSG00000229953	758	-0.33269	0.001768	0.024008
RP5-901A4.1	ENSG00000255306	739	-0.33216	0.005445	0.047752
RP4-591N18.2	ENSG00000232564	2483	-0.33201	0.000766	0.01471
NATD1	ENSG00000274180	4931	-0.33201	0.00145	0.021343
NCOR2	ENSG00000196498	13975	-0.33179	0.000121	0.005346
AIRN	ENSG00000268257	4374	-0.33177	0.000828	0.015324
RP11-862L9.3	ENSG00000266844	2478	-0.33117	0.000142	0.005755
CTD-2576D5.4	ENSG00000261448	641	-0.33065	5.92E-06	0.001037
RP11-54H7.4	ENSG00000275216	4205	-0.3306	0.0052	0.046594
RP11-574K11.29	ENSG00000272140	1192	-0.33058	0.000846	0.0155
AC004490.1	ENSG00000267122	2738	-0.33044	0.005569	0.04835
AC004837.5	ENSG00000228554	731	-0.33043	0.000289	0.0086
ITFG3	ENSG00000167930	4699	-0.32996	0.001448	0.021314
MT-CO2	ENSG00000198712	684	-0.32994	0.001513	0.021862
GOLGA2P5	ENSG00000238105	10957	-0.32982	0.001178	0.018908

AC073130.3	ENSG00000243243	917	-0.32934	0.004275	0.04124
GS1-124K5.11	ENSG00000229180	7092	-0.32917	0.000698	0.013945
RP11-1046B16.3	ENSG00000273797	527	-0.32915	0.002326	0.028345
ITSN1	ENSG00000205726	21748	-0.32909	4.5E-06	0.00086
RP4-569M23.5	ENSG00000267882	2253	-0.32869	0.001265	0.019597
SPTB	ENSG00000070182	14772	-0.32863	0.000394	0.010124
NRBF2P5	ENSG00000270427	788	-0.32857	0.002653	0.030787
AC005519.4	ENSG00000258559	1367	-0.32833	0.004353	0.04166
AC124914.3	ENSG00000231395	518	-0.32828	0.00262	0.030524
CIC	ENSG00000079432	9494	-0.32825	0.000987	0.016984
RP1-86D1.2	ENSG00000228972	1828	-0.3281	0.003346	0.035524
RP11-325L12.5	ENSG00000270218	589	-0.32783	0.003598	0.037179
RP11-946L20.4	ENSG00000253103	900	-0.32778	0.00036	0.009633
RP11-120K19.3	ENSG00000279236	1303	-0.32732	0.002745	0.031391
FKSG61	ENSG00000280308	375	-0.32686	0.00128	0.019765
AC007192.6	ENSG00000269145	463	-0.32675	0.001079	0.018056
RP11-304L19.11	ENSG00000261663	788	-0.32634	0.002422	0.029045
RP1-86D1.4	ENSG00000227112	339	-0.32579	0.00462	0.043079
RP11-415F23.4	ENSG00000272529	551	-0.32553	0.000972	0.016885
AHNAK	ENSG00000124942	19465	-0.32549	0.000414	0.010372
TMEM51-AS1	ENSG00000175147	7100	-0.32545	0.000285	0.008535
NOTCH4	ENSG00000204301	9748	-0.32484	0.00171	0.02356
C4orf32	ENSG00000174749	8901	-0.32467	7.06E-05	0.004117
NPM1P29	ENSG00000239351	803	-0.32385	0.001087	0.018114
KIAA1217	ENSG00000120549	10392	-0.32346	6.18E-06	0.001068
FOXP2	ENSG00000128573	15782	-0.32336	0.00327	0.034935
CASZ1	ENSG00000130940	11336	-0.32313	0.001369	0.020516
RP11-97O12.6	ENSG00000271997	3844	-0.32287	0.002496	0.029643
ATP11A	ENSG00000068650	13733	-0.32188	0.000299	0.008696
RP11-167N5.5	ENSG00000267834	497	-0.32164	0.00442	0.042073
LINC00954	ENSG00000228784	4683	-0.32154	0.005688	0.04895
TRIM17	ENSG00000162931	3548	-0.32141	0.001002	0.017189
SLC25A37	ENSG00000147454	8147	-0.32104	0.000309	0.008834
RP11-57H12.3	ENSG00000226026	2505	-0.321	0.005424	0.047709
TCF7	ENSG00000081059	10063	-0.3201	6.78E-06	0.001124
NRARP	ENSG00000198435	1770	-0.3198	0.000647	0.013478
BNIP3P5	ENSG00000260571	498	-0.31936	0.00154	0.022097
RP11-384K6.6	ENSG00000260404	4395	-0.31904	0.003589	0.03713
RP11-322E11.2	ENSG00000275512	2533	-0.31879	0.000556	0.012313
PKD1P6	ENSG00000250251	6015	-0.31878	0.002879	0.032351
AC016831.7	ENSG00000233559	1616	-0.31866	0.000221	0.007424

RPLP1P11	ENSG00000243686	341	-0.31837	0.004762	0.043941
RP11-326N17.2	ENSG00000274281	2127	-0.31789	0.000514	0.011834
RP11-157K17.5	ENSG00000179038	1599	-0.3177	0.002171	0.027278
RP11-306O13.1	ENSG00000213121	1212	-0.31763	0.001623	0.022793
VMAC	ENSG00000187650	2254	-0.31758	0.000299	0.008696
FAM84A	ENSG00000162981	8384	-0.31758	0.001479	0.021614
MIR1281	ENSG00000221160	54	-0.31711	0.001485	0.021643
UVSSA	ENSG00000163945	7830	-0.31705	0.002893	0.032406
WDR88	ENSG00000166359	1841	-0.31702	0.001166	0.018836
PRR12	ENSG00000126464	7093	-0.31667	0.002582	0.030206
AC007191.4	ENSG00000279407	2995	-0.31637	0.002096	0.026732
BCAN	ENSG00000132692	4931	-0.3162	0.001366	0.020482
RP11-335G20.7	ENSG00000259984	459	-0.31616	0.003874	0.038774
NOL4L	ENSG00000197183	9448	-0.31599	2.14E-05	0.00211
RP4-569M23.2	ENSG00000231119	1809	-0.31598	0.002109	0.026836
ADAMTSL4	ENSG00000143382	6432	-0.31591	0.004809	0.044152
VIPR1-AS1	ENSG00000232354	2743	-0.31587	0.001781	0.024153
CTB-39G8.3	ENSG00000267344	457	-0.3157	0.001	0.017155
FOXP4	ENSG00000137166	6170	-0.3156	0.000754	0.014552
MGAT4C	ENSG00000182050	27763	-0.31539	0.004957	0.045075
RP11-419C5.2	ENSG00000226232	1728	-0.31523	0.001307	0.020039
PAICSP1	ENSG00000226055	1217	-0.315	0.001318	0.020148
HNRNPMP1	ENSG00000259335	1768	-0.31499	0.003774	0.038204
TET2-AS1	ENSG00000251586	537	-0.31483	0.003423	0.036104
SPIRE2	ENSG00000204991	7975	-0.31467	0.001621	0.022793
YBX2	ENSG00000006047	4618	-0.31445	0.004981	0.045208
CFAP52	ENSG00000166596	3078	-0.31444	0.00194	0.025399
RP11-144I2.1	ENSG00000279029	1855	-0.31437	0.002829	0.032062
RP11-138I18.1	ENSG00000271360	923	-0.31424	0.003059	0.033666
CAMSAP3	ENSG00000076826	4849	-0.31415	0.001876	0.024905
MAML3	ENSG00000196782	7012	-0.31412	5.57E-05	0.00368
RP11-10O22.1	ENSG00000241168	1834	-0.31341	0.004514	0.042518
AGAP1-IT1	ENSG00000235529	664	-0.31278	0.002236	0.027677
RP11-75C10.6	ENSG00000279801	2464	-0.31267	0.003722	0.037892
HNRNPA1P49	ENSG00000233231	904	-0.31217	0.000977	0.016916
RP11-321L2.2	ENSG00000237414	687	-0.31172	0.001525	0.021957
KMT2B	ENSG00000272333	10279	-0.31151	0.000387	0.010037
TLE3	ENSG00000140332	12617	-0.31144	3.22E-05	0.00276
CTD-3099C6.11	ENSG00000268970	644	-0.31137	0.003508	0.036679
SEMA3F	ENSG00000001617	4826	-0.31136	0.003564	0.037022
RP4-724E16.2	ENSG00000197670	1947	-0.31119	0.002219	0.027658

LRFN3	ENSG00000126243	4044	-0.31108	0.003276	0.034992
SATB2	ENSG00000119042	10958	-0.31016	0.003122	0.034056
RASA4	ENSG00000105808	8077	-0.31002	0.005553	0.048249
BNIP3	ENSG00000176171	4847	-0.30954	0.002257	0.027831
CTD-2574D22.3	ENSG00000279789	2194	-0.30908	0.000527	0.012001
RPL3P1	ENSG00000228149	1211	-0.30893	0.00587	0.049922
AC006128.2	ENSG00000279716	1661	-0.30887	0.000135	0.005613
FZD7	ENSG00000155760	3859	-0.30823	0.00558	0.04837
KIAA1671	ENSG00000197077	15818	-0.30775	0.000387	0.010037
GLUD1P3	ENSG00000250959	1028	-0.3077	0.000138	0.005673
NUMBL	ENSG00000105245	5823	-0.30768	0.001252	0.019535
AC073046.25	ENSG00000235499	390	-0.3062	0.002592	0.030288
ANO6	ENSG00000177119	8235	-0.30573	0.001618	0.022793
PRR5	ENSG00000186654	5247	-0.30542	0.002824	0.03202
AC114730.11	ENSG00000235351	584	-0.30529	0.000361	0.009649
RP11-25C19.3	ENSG00000233340	358	-0.30528	0.002396	0.028857
RP3-453C12.14	ENSG00000275894	709	-0.30511	0.00303	0.033442
CTB-52I2.7	ENSG00000279172	442	-0.30507	0.001739	0.023815
MAGI1	ENSG00000151276	11649	-0.30488	5.35E-07	0.000231
AC006548.28	ENSG00000280156	84332	-0.30474	0.000349	0.009506
AC008746.5	ENSG00000235681	218	-0.3041	0.004233	0.041038
XYLT1	ENSG00000103489	11004	-0.30407	8.36E-07	0.000318
ASB14	ENSG00000239388	3232	-0.30356	4.67E-05	0.003332
NAP1L4P1	ENSG00000177173	1157	-0.30266	0.001258	0.019567
AC015849.19	ENSG00000270871	488	-0.3023	0.003138	0.034177
RPS6KA5	ENSG00000100784	27726	-0.30229	0.000372	0.009842
AF131215.3	ENSG00000254936	1741	-0.30223	0.004757	0.043928
EPS8L3	ENSG00000198758	3477	-0.30175	0.00029	0.008607
PACS1	ENSG00000175115	9037	-0.30164	0.000164	0.006222
ARR3	ENSG00000120500	1729	-0.30103	0.004343	0.041581
TMEM45A	ENSG00000181458	6005	-0.30092	0.000184	0.006691
TCTE3	ENSG00000184786	769	-0.30077	0.002513	0.029798
TMEM198B	ENSG00000182796	3564	-0.30045	0.000108	0.00508
CDK18	ENSG00000117266	6831	-0.30037	0.002375	0.028733
Z83851.4	ENSG00000233903	545	-0.29952	0.002541	0.029912
RNF44	ENSG00000146083	5660	-0.2995	0.000172	0.006425
RP11-89H19.1	ENSG00000205537	1287	-0.29942	0.002283	0.027981
AC092669.3	ENSG00000232164	1679	-0.299	0.000249	0.007947
LA16c-60D12.1	ENSG00000280341	6114	-0.29884	0.000489	0.011511
CTD-2630F21.1	ENSG00000267309	1392	-0.29851	0.000479	0.011314
IL22RA1	ENSG00000142677	2798	-0.29839	0.003681	0.037651

ICOSLG	ENSG00000160223	8110	-0.29788	0.003241	0.034763
AGAP6	ENSG00000204149	3160	-0.29762	6.32E-05	0.003815
KLF9	ENSG00000119138	5175	-0.29757	6.92E-05	0.004092
TLN2	ENSG00000171914	16202	-0.29718	0.003884	0.038817
MNX1	ENSG00000130675	4202	-0.29696	0.001724	0.023704
RP11-815J21.1	ENSG00000259453	746	-0.29693	0.003811	0.038429
CICP27	ENSG00000233750	3812	-0.29655	0.005803	0.049518
AC010243.1	ENSG00000279188	369	-0.2959	0.005331	0.047234
ACVR2B-AS1	ENSG00000229589	2517	-0.29583	0.004742	0.043861
NBPF15	ENSG00000266338	5306	-0.29533	0.002102	0.026774
ANKRD11	ENSG00000167522	16949	-0.2953	3.57E-06	0.000768
RP13-1056D16.2	ENSG00000244730	330	-0.29508	0.003727	0.037892
AC078883.3	ENSG00000232788	1077	-0.29506	0.003871	0.038751
RP11-53I6.2	ENSG00000263917	565	-0.29505	0.000915	0.016338
FGFRL1	ENSG00000127418	4145	-0.29482	0.000328	0.009191
RP5-1160K1.6	ENSG00000228703	688	-0.29452	0.000269	0.00824
TCF7L2	ENSG00000148737	4935	-0.29441	0.001024	0.017381
RPL29P14	ENSG00000241112	428	-0.29428	0.003976	0.039444
ANGPTL2	ENSG00000136859	3889	-0.29409	0.00155	0.022148
ARRDC2	ENSG00000105643	4637	-0.29339	0.00023	0.007647
CTD-2583A14.11	ENSG00000270804	936	-0.29263	0.000538	0.012135
SCAF1	ENSG00000126461	4548	-0.29229	0.00148	0.021614
RP11-337C18.8	ENSG00000237188	3618	-0.29208	0.000687	0.013855
RP11-365N19.2	ENSG00000259515	828	-0.29206	0.000268	0.00824
RP11-848P1.4	ENSG00000266340	668	-0.29188	0.005224	0.046721
BAIAP2L2	ENSG00000128298	2146	-0.2918	0.003853	0.038632
RP11-257O5.2	ENSG00000279059	1667	-0.29115	0.00209	0.026712
SH3BP2	ENSG00000087266	13968	-0.29099	0.000655	0.013567
GPR126	ENSG00000112414	8473	-0.29088	0.00183	0.024574
MAPK8IP3	ENSG00000138834	10285	-0.29021	0.002153	0.027159
LRRTM2	ENSG00000146006	6095	-0.28993	0.001336	0.020271
SKI	ENSG00000157933	6080	-0.28986	0.000243	0.007878
TMEM14E	ENSG00000221962	378	-0.28958	0.001848	0.024717
PPP1R26-AS1	ENSG00000225361	3750	-0.28941	0.001901	0.025119
CDCA4P4	ENSG00000234219	680	-0.28934	0.00075	0.014494
PCBP2-OT1	ENSG00000273658	182	-0.28928	0.000468	0.011158
C8orf44	ENSG00000213865	2505	-0.28924	0.000699	0.013945
PABPC1P1	ENSG00000231707	591	-0.28921	0.001308	0.020039
DYRK1B	ENSG00000105204	3192	-0.28916	0.003363	0.035639
AC007383.3	ENSG00000227946	1034	-0.28906	9.17E-06	0.001298
AC114498.1	ENSG00000276171	63	-0.28882	0.003233	0.034723

RP11-108L7.4	ENSG00000236662	342	-0.28875	0.000502	0.011705
SMAD3	ENSG00000166949	9113	-0.28838	0.000206	0.007171
C6orf132	ENSG00000188112	6852	-0.28822	0.001336	0.020271
RP4-584D14.5	ENSG00000240449	577	-0.28821	0.005331	0.047234
WASH4P	ENSG00000234769	5322	-0.28802	0.000495	0.011611
SH2D3A	ENSG00000125731	5716	-0.28748	0.005421	0.047709
AMER1	ENSG00000184675	8443	-0.28696	0.001222	0.019323
RP11-1376P16.2	ENSG00000265678	555	-0.28687	0.002267	0.027865
RP11-549J18.1	ENSG00000251023	3157	-0.2864	0.004795	0.044081
AC011747.4	ENSG00000236008	8956	-0.28629	0.00141	0.020981
RP11-425M5.7	ENSG00000276603	419	-0.28587	0.001755	0.023967
CTD-2619J13.14	ENSG00000232098	3417	-0.28586	0.0003	0.008696
MICAL3	ENSG00000243156	22354	-0.285	2.34E-05	0.002189
ZFP41	ENSG00000264668	2897	-0.28497	0.002513	0.029798
PIK3R2	ENSG00000105647	4524	-0.28489	0.000133	0.005604
RP4-534N18.2	ENSG00000228634	517	-0.2848	0.005063	0.045731
MIR194-2	ENSG00000229719	3647	-0.28436	0.00286	0.032288
PTGES2-AS1	ENSG00000232850	1938	-0.28435	0.003398	0.035932
CSAD	ENSG00000139631	4390	-0.28402	0.000663	0.013646
RP11-77H9.5	ENSG00000259939	409	-0.28393	0.000889	0.015995
SOX13	ENSG00000143842	7420	-0.28309	0.000706	0.014023
RP11-739N20.2	ENSG00000226330	486	-0.28279	0.005603	0.048489
ODCP	ENSG00000244556	1639	-0.2826	0.001803	0.024323
RP11-400N9.1	ENSG00000259793	2285	-0.28257	0.003095	0.033909
LINC00887	ENSG00000214145	4153	-0.28243	0.003504	0.036668
RP11-175P13.3	ENSG00000257489	1405	-0.28195	0.001913	0.025178
XXbac-B461K10.4	ENSG00000093100	4328	-0.28176	0.00062	0.013137
MAGI2	ENSG00000187391	8946	-0.28175	0.003141	0.034177
AC096772.6	ENSG00000244567	1262	-0.28147	0.00123	0.019395
RP11-624A21.1	ENSG00000279912	3925	-0.28135	0.000582	0.012714
WASH6P	ENSG00000182484	4618	-0.28133	0.003181	0.034431
DGCR11	ENSG00000273311	2214	-0.27977	9.68E-05	0.004873
RP11-245J9.5	ENSG00000271843	611	-0.27959	0.002417	0.028993
RP11-981P6.1	ENSG00000258302	1711	-0.27957	0.004804	0.044122
RP11-333E13.2	ENSG00000250568	315	-0.27955	0.001065	0.017886
CTD-2024I7.13	ENSG00000246422	2480	-0.2795	0.004361	0.041716
SLX4	ENSG00000188827	8591	-0.27938	0.000711	0.014085
TPM3P9	ENSG00000241015	2920	-0.27929	8.58E-05	0.004574
GP1BA	ENSG00000185245	2501	-0.27911	0.00585	0.049804
CREBBP	ENSG00000005339	15068	-0.27909	1.8E-06	0.000504
LINC00894	ENSG00000235703	6323	-0.27907	0.002642	0.030704

RP11-848P1.5	ENSG00000264107	492	-0.27839	0.004556	0.042762
KB-1247B1.1	ENSG00000249510	833	-0.27836	0.000873	0.015849
TRANK1	ENSG00000168016	11177	-0.27803	0.00375	0.038089
CCDC157	ENSG00000187860	6586	-0.27779	0.001692	0.023377
RP11-403H13.1	ENSG00000224972	553	-0.27754	0.003176	0.034409
MGRN1	ENSG00000102858	10219	-0.27749	0.001788	0.024188
RP3-477O4.14	ENSG00000230155	839	-0.27721	0.001194	0.019055
IMPDH1P10	ENSG00000232133	1542	-0.277	0.00148	0.021614
MTA1	ENSG00000182979	6966	-0.27695	0.000359	0.009626
RP11-174G6.5	ENSG00000261324	5428	-0.27676	0.00052	0.011906
LUC7L	ENSG00000007392	6176	-0.27604	8.39E-08	7.64E-05
MAST2	ENSG00000086015	7877	-0.27572	0.001118	0.018442
TJP3	ENSG00000105289	4217	-0.2755	0.003431	0.036144
CTD-2117L12.1	ENSG00000250127	1184	-0.27545	0.004204	0.040847
AL078471.5	ENSG00000273840	3336	-0.27534	0.000588	0.01278
RP11-382B18.1	ENSG00000279417	3930	-0.2751	0.000596	0.012854
SLC23A2	ENSG00000089057	7515	-0.27455	3.62E-05	0.002905
KMT2D	ENSG00000167548	20476	-0.27448	0.000693	0.013899
RP11-153M7.3	ENSG00000250771	3277	-0.27446	0.001025	0.017381
LENG8-AS1	ENSG00000226696	1248	-0.27434	0.001187	0.018997
KIAA1161	ENSG00000164976	6398	-0.27391	0.002051	0.026467
PLEKHA7	ENSG00000166689	9575	-0.27379	0.001032	0.017454
RP11-463O12.5	ENSG00000280138	18662	-0.27353	0.000502	0.011705
SETD1B	ENSG00000139718	8314	-0.27348	0.00066	0.013603
PSMD6-AS2	ENSG00000239653	2456	-0.27259	0.001183	0.018947
PTPN23	ENSG00000076201	5981	-0.27241	0.002679	0.031022
KIZ-AS1	ENSG00000232712	5451	-0.27184	0.003912	0.039051
AC087294.2	ENSG00000235530	1519	-0.2718	0.002635	0.030635
RP11-206L10.2	ENSG00000228327	1317	-0.27137	0.002276	0.02792
CTC-444N24.6	ENSG00000267871	738	-0.27084	0.001564	0.022278
RP4-758J18.13	ENSG00000272455	1523	-0.27082	0.000275	0.008351
RP3-395M20.8	ENSG00000238164	6137	-0.27074	0.001017	0.0173
WASH5P	ENSG00000225373	6599	-0.27067	0.000841	0.015449
RP11-317N8.5	ENSG00000258938	2867	-0.27064	0.002452	0.029247
ABHD16B	ENSG00000183260	2094	-0.27007	0.003521	0.036745
RP11-700A24.1	ENSG00000279742	2387	-0.26985	0.001949	0.025431
AC092415.1	ENSG00000235493	850	-0.26965	0.002944	0.032789
RP11-767N6.2	ENSG00000234329	788	-0.26938	0.002706	0.031232
PER2	ENSG00000132326	6877	-0.26925	0.002095	0.026732
RP13-516M14.8	ENSG00000280407	2778	-0.26894	0.001311	0.020085
ARHGEF5	ENSG00000050327	5818	-0.26875	0.001927	0.025275

SGK494	ENSG00000167524	4663	-0.26755	0.000618	0.01312
RBPMS	ENSG00000157110	8931	-0.26728	0.000101	0.004971
AC093110.3	ENSG00000238018	602	-0.26715	0.002005	0.026026
PLEKHG2	ENSG00000090924	9200	-0.26674	0.005653	0.048804
ZBTB20-AS1	ENSG00000241560	2035	-0.26672	0.000883	0.01593
PTPRJ	ENSG00000149177	9620	-0.26647	0.000266	0.008226
RP4-591C20.9	ENSG00000268858	3082	-0.26638	0.000595	0.012854
AC005785.2	ENSG00000268189	1911	-0.26629	0.000142	0.005755
RP11-449P15.2	ENSG00000273151	6758	-0.26621	0.003232	0.034723
ADNP-AS1	ENSG00000259456	927	-0.26563	0.00238	0.028733
RP11-474B12.1	ENSG00000261369	870	-0.26557	0.004949	0.04503
RP11-334A14.2	ENSG00000236360	502	-0.26555	0.003544	0.036859
TNRC6C	ENSG00000078687	11058	-0.26552	0.000161	0.006139
TRAK1	ENSG00000182606	10992	-0.26519	0.001689	0.02336
F2RL2	ENSG00000164220	3429	-0.26449	0.003926	0.039114
RP11-24N18.1	ENSG00000260570	295	-0.26425	0.00326	0.03489
AC007256.5	ENSG00000213090	2765	-0.264	0.002312	0.028228
CYP4F12	ENSG00000186204	5351	-0.26384	0.002056	0.026499
RP11-455O6.8	ENSG00000276101	610	-0.26322	0.00387	0.038751
GATS	ENSG00000239521	4860	-0.26276	0.001136	0.018537
FAM21A	ENSG00000099290	5162	-0.26249	0.000137	0.005622
ZSWIM5	ENSG00000162415	6046	-0.26239	0.004094	0.040173
RP11-395B7.7	ENSG00000260336	3921	-0.26239	0.000369	0.00978
RP11-707P17.1	ENSG00000259678	560	-0.262	0.00028	0.008422
RP11-1035H13.2	ENSG00000260017	361	-0.26159	1.05E-05	0.001391
LY6G5B	ENSG00000240053	3031	-0.26072	0.001293	0.019886
AC093818.1	ENSG00000225205	2447	-0.26067	0.000472	0.011228
AC002543.2	ENSG00000235945	1954	-0.25995	0.003538	0.036851
RP11-346D14.1	ENSG00000259380	2982	-0.25991	0.001563	0.022273
XXbac-BPG154L12.4	ENSG00000225914	3458	-0.25945	0.001709	0.02356
DSC2	ENSG00000134755	12371	-0.25902	0.004791	0.044059
PCNT	ENSG00000160299	11660	-0.25873	1.43E-05	0.001653
TPT1-AS1	ENSG00000170919	10402	-0.25844	0.000411	0.010339
RP11-294J22.7	ENSG00000278963	2151	-0.25838	0.001912	0.025178
DNHD1	ENSG00000179532	19629	-0.25832	0.00527	0.046938
TRIM73	ENSG00000178809	5544	-0.25809	0.004099	0.040202
ZNRF3	ENSG00000183579	6968	-0.25775	0.001725	0.023704
BRD4	ENSG00000141867	12093	-0.25772	0.000102	0.004972
MEF2D	ENSG00000116604	6400	-0.25751	0.000277	0.008365
RP11-229D13.3	ENSG00000280388	1987	-0.25706	0.003146	0.034209

HIC1	ENSG00000177374	8108	-0.25665	0.00489	0.044625
KIAA1328	ENSG00000150477	11127	-0.2563	0.000524	0.01196
SLC7A8	ENSG00000092068	5991	-0.25627	0.00076	0.014609
SRCAP	ENSG00000080603	15255	-0.25621	0.000573	0.012604
EHBP1L1	ENSG00000173442	6705	-0.25602	0.003582	0.03713
EGLN1P1	ENSG00000254779	762	-0.25556	0.00499	0.045247
TEX22	ENSG00000226174	2837	-0.25554	0.001029	0.017443
RP3-521E19.2	ENSG00000257494	751	-0.25531	0.001894	0.025074
C1RL-AS1	ENSG00000205885	6094	-0.25524	0.000594	0.012849
TTC21A	ENSG00000168026	7677	-0.2552	0.004293	0.04137
RP11-163E9.2	ENSG00000239969	740	-0.25491	0.00013	0.005521
FAM193B	ENSG00000146067	5804	-0.25477	0.002904	0.032472
CTD-2260A17.3	ENSG00000272109	569	-0.2544	0.004332	0.041534
PIK3C2B	ENSG00000133056	9414	-0.25408	0.001074	0.018018
RP11-627G18.1	ENSG00000266283	770	-0.25397	0.004171	0.040656
LINC00674	ENSG00000237854	734	-0.25394	9.17E-05	0.00476
PXN	ENSG00000089159	7376	-0.25356	0.004504	0.042503
RP11-15I20.1	ENSG00000272814	274	-0.25355	0.003589	0.03713
HIVEP1	ENSG00000095951	10979	-0.25311	3.3E-06	0.000739
WASF2	ENSG00000158195	5676	-0.253	6.13E-06	0.001067
FOXK1	ENSG00000164916	13093	-0.25255	0.000737	0.014375
CTD-2369P2.12	ENSG00000267303	1850	-0.25251	0.002259	0.027841
RP11-295I5.4	ENSG00000275197	563	-0.25245	0.005577	0.048363
IQSEC2	ENSG00000124313	6962	-0.25198	0.00488	0.044594
ATP2A1	ENSG00000196296	4587	-0.25196	0.002837	0.032114
RP11-307E17.8	ENSG00000232063	2162	-0.25169	0.001509	0.021834
MAOB	ENSG00000069535	2891	-0.25168	0.003583	0.03713
EP300	ENSG00000100393	9585	-0.25137	3.41E-06	0.000753
DLGAP4-AS1	ENSG00000232907	2437	-0.25048	0.000142	0.005755
RP13-487P22.1	ENSG00000261529	1603	-0.25043	0.002869	0.032351
DSP	ENSG00000096696	10093	-0.25025	0.000171	0.006394
RP11-126O1.4	ENSG00000267476	670	-0.24994	0.003344	0.035524
CCDC88C	ENSG00000015133	12426	-0.24959	0.000152	0.005969
AL157871.2	ENSG00000259052	206	-0.24939	1.39E-05	0.001625
LTBP3	ENSG00000168056	10543	-0.24919	0.002095	0.026732
FBRS	ENSG00000156860	7218	-0.24908	0.00077	0.014749
PLXDC2	ENSG00000120594	12567	-0.24896	0.004366	0.041736
C12orf50	ENSG00000165805	5609	-0.24879	0.002575	0.030148
RP11-259O2.3	ENSG00000249731	560	-0.2484	0.004563	0.042762
ECHDC2	ENSG00000121310	11572	-0.24773	0.005442	0.047752
RP11-499P20.2	ENSG00000240291	1575	-0.2468	0.002438	0.029176

RP11-156P1.3	ENSG00000262879	3348	-0.24658	0.001147	0.018661
FOXJ2	ENSG00000065970	6296	-0.24631	3.19E-06	0.000721
TAF4	ENSG00000130699	9808	-0.246	8.01E-05	0.004442
AC007743.1	ENSG00000233251	3713	-0.24581	0.005082	0.045871
COBL	ENSG00000106078	14304	-0.24532	0.004146	0.040499
CTB-58E17.9	ENSG00000275665	563	-0.24529	0.000268	0.00824
AMPD2	ENSG00000116337	6823	-0.24498	0.003147	0.034212
WASH2P	ENSG00000146556	2958	-0.24478	0.005533	0.048151
AGER	ENSG00000204305	2077	-0.24464	0.002482	0.029505
PROSER3	ENSG00000167595	6327	-0.24459	0.005097	0.045939
TTC7A	ENSG00000068724	6949	-0.24449	0.003594	0.037149
RGL4	ENSG00000159496	4715	-0.24336	0.00113	0.018506
CALCOCO1	ENSG00000012822	8898	-0.24327	0.000294	0.008664
RP11-242D8.1	ENSG00000267002	1191	-0.24314	0.002463	0.029312
PLEKHG3	ENSG00000126822	11910	-0.24287	0.005489	0.047933
NR6A1	ENSG00000148200	7124	-0.24258	0.003994	0.039584
SYT2	ENSG00000143858	7774	-0.24226	0.004975	0.045177
NFATC2	ENSG00000101096	8286	-0.24188	0.001682	0.023335
ZYX	ENSG00000159840	3482	-0.24179	0.003941	0.039195
POM121	ENSG00000196313	9821	-0.24141	0.000231	0.007647
EXT1	ENSG00000182197	8325	-0.24126	1.88E-05	0.001953
GAB2	ENSG00000033327	6771	-0.23968	8.06E-05	0.004442
MLANA	ENSG00000120215	3083	-0.23941	0.004191	0.040763
FNBP4	ENSG00000109920	7596	-0.23902	6.72E-06	0.001124
UGDH-AS1	ENSG00000249348	2787	-0.23837	0.002571	0.030123
TACC2	ENSG00000138162	18835	-0.23831	0.001873	0.024881
FCAMR	ENSG00000162897	3541	-0.23831	0.005877	0.049963
ANKMY1	ENSG00000144504	11477	-0.23791	0.001229	0.019394
RAPGEF1	ENSG00000107263	7127	-0.23782	3.5E-05	0.002905
TOX3	ENSG00000103460	5031	-0.23782	0.000717	0.014158
LPAR5	ENSG00000184574	3258	-0.23761	0.000279	0.008399
STAG3	ENSG00000066923	8583	-0.23737	0.003787	0.038272
SAMD4B	ENSG00000179134	8057	-0.23731	0.000122	0.005346
KIZ	ENSG00000088970	5207	-0.23724	0.001455	0.021349
ELMSAN1	ENSG00000156030	13484	-0.23723	0.000622	0.013167
CDC42BPB	ENSG00000198752	7958	-0.23665	0.000294	0.008664
CD99P1	ENSG00000223773	5921	-0.23649	0.000543	0.012171
AP000347.2	ENSG00000272578	7012	-0.23636	0.001696	0.02342
VDR	ENSG00000111424	5674	-0.23595	9.39E-05	0.004783
ZNF608	ENSG00000168916	6922	-0.23593	0.002147	0.027118
PDZD2	ENSG00000133401	15642	-0.23575	0.002497	0.029647

BRD1	ENSG00000100425	5850	-0.23571	3.73E-05	0.002942
AC022384.1	ENSG00000273665	87	-0.23461	0.002147	0.027118
RRN3P3	ENSG00000257122	3366	-0.23429	0.001259	0.019567
EEF2K	ENSG00000103319	8020	-0.23373	0.001957	0.025483
CTB-152G17.6	ENSG00000272918	2646	-0.23369	0.002081	0.026663
TRIM56	ENSG00000169871	3933	-0.23313	0.000971	0.016885
CTB-191K22.5	ENSG00000267815	457	-0.23276	0.004797	0.044081
PIP5K1C	ENSG00000186111	6694	-0.23261	0.004534	0.042617
BEND7	ENSG00000165626	9815	-0.23191	0.005801	0.049518
FAM193A	ENSG00000125386	6174	-0.23104	6.91E-06	0.001124
WIZ	ENSG00000011451	7965	-0.23079	0.004883	0.044594
TBC1D17	ENSG00000104946	4745	-0.2307	0.002319	0.028271
C10orf12	ENSG00000155640	4929	-0.22985	0.000101	0.004971
PHLDB1	ENSG00000019144	21722	-0.22931	0.001622	0.022793
PCBP2	ENSG00000197111	7926	-0.22861	0.000172	0.006425
AC024560.3	ENSG00000214135	3502	-0.22843	0.000293	0.008664
UTS2B	ENSG00000188958	3850	-0.22835	0.001553	0.022162
SPEN	ENSG00000065526	13294	-0.22769	1.69E-05	0.001858
RP4-635E18.8	ENSG00000271895	870	-0.22743	0.005659	0.048813
DIP2A	ENSG00000160305	13557	-0.22684	0.000561	0.01241
WDPCP	ENSG00000143951	11776	-0.22676	0.002807	0.031955
POM121C	ENSG00000272391	8767	-0.22674	0.000589	0.012781
MMRN2	ENSG00000173269	5334	-0.22671	0.005577	0.048363
RP11-493P1.2	ENSG00000258034	501	-0.22655	0.00112	0.018442
RP11-568K15.1	ENSG00000242193	5564	-0.22618	0.0012	0.019099
PDXDC2P	ENSG00000196696	6114	-0.22586	0.001434	0.021212
PLEKHH1	ENSG00000054690	10817	-0.2258	0.001568	0.022314
KIAA1211L	ENSG00000196872	4705	-0.22574	0.003384	0.035834
RP11-373L24.1	ENSG00000267520	5702	-0.22557	0.000811	0.015195
AGFG2	ENSG00000106351	5478	-0.22538	0.002068	0.026558
RAD51-AS1	ENSG00000245849	1696	-0.22518	0.003966	0.039389
RP4-613B23.1	ENSG00000230084	785	-0.22509	0.002968	0.032931
DAPK1	ENSG00000196730	8907	-0.22455	0.000821	0.015254
DLGAP4	ENSG00000080845	8789	-0.22411	0.000439	0.010737
RP11-927P21.5	ENSG00000263647	492	-0.22344	0.003141	0.034177
RALGDS	ENSG00000160271	7624	-0.22333	0.003601	0.037183
RPL32P3	ENSG00000251474	4504	-0.2232	0.001473	0.021565
ZC3H4	ENSG00000130749	6502	-0.22319	0.000959	0.016775
TRIM8	ENSG00000171206	2916	-0.22258	0.000148	0.005876
NRBP2	ENSG00000185189	4924	-0.22251	0.004154	0.040567
TET3	ENSG00000187605	12271	-0.22241	0.000127	0.005505

NRIP2	ENSG00000053702	2803	-0.22238	0.004431	0.042152
RP9P	ENSG00000205763	1433	-0.22195	0.004409	0.042036
AAK1	ENSG00000115977	24845	-0.22175	0.002335	0.028399
FAM222B	ENSG00000173065	5514	-0.22166	0.00073	0.014283
CHERP	ENSG00000085872	6113	-0.22152	0.002304	0.028169
MAP3K12	ENSG00000139625	7971	-0.22123	0.00112	0.018442
RP1-41C23.2	ENSG00000278977	768	-0.2212	0.003916	0.03906
GUSBP11	ENSG00000228315	6550	-0.22068	0.001766	0.024008
TJAP1	ENSG00000137221	6741	-0.22066	0.005265	0.046916
PAXBP1-AS1	ENSG00000238197	4046	-0.22062	0.002745	0.031391
TNS3	ENSG00000136205	11797	-0.21968	0.002521	0.029831
KLF5	ENSG00000102554	3893	-0.21958	0.000747	0.014464
NCOA6	ENSG00000198646	10564	-0.21928	0.000587	0.012771
TGFB1	ENSG00000105329	3238	-0.21919	0.000117	0.005271
DIAPH2	ENSG00000147202	9550	-0.21897	0.000194	0.006868
KIAA0226	ENSG00000145016	8613	-0.21695	0.0008	0.015119
PRR26	ENSG00000180525	4835	-0.21692	0.000712	0.014085
FOXP1	ENSG00000114861	13628	-0.2154	0.000246	0.007896
RP11-1007O24.3	ENSG00000261423	2713	-0.21524	0.001186	0.018988
MAML2	ENSG00000184384	7109	-0.21521	0.004982	0.045208
IGF2BP2	ENSG00000073792	4735	-0.21466	0.000134	0.005613
TCF3	ENSG00000071564	6080	-0.21465	0.003485	0.036524
SLC22A23	ENSG00000137266	8898	-0.21439	0.001861	0.024805
SMG6	ENSG00000070366	10554	-0.21432	5.83E-05	0.003767
PRKACA	ENSG00000072062	6144	-0.21386	0.005456	0.047798
BOD1L1	ENSG00000038219	15860	-0.2127	1.63E-05	0.001809
BAZ2A	ENSG00000076108	10918	-0.21255	3.3E-05	0.002786
MINK1	ENSG00000141503	7817	-0.21227	0.002878	0.032351
RP11-603J24.7	ENSG00000237493	604	-0.21226	0.002888	0.032374
AGAP1	ENSG00000157985	13898	-0.21223	0.001284	0.019798
CFLAR-AS1	ENSG00000226312	3166	-0.212	0.000367	0.009751
MIR6080	ENSG00000215769	3038	-0.21139	0.001256	0.019567
BAGE2	ENSG00000187172	4080	-0.21138	0.002535	0.029867
RP11-849H4.4	ENSG00000251143	4233	-0.21133	0.001913	0.025178
CYTH1	ENSG00000108669	7754	-0.21118	0.000417	0.010423
SGSM2	ENSG00000141258	9771	-0.21076	0.005693	0.048975
ZNF516	ENSG00000101493	8217	-0.21035	0.001752	0.023933
ZBTB20	ENSG00000181722	8279	-0.21029	0.002409	0.028944
PPP1R3E	ENSG00000235194	5548	-0.20993	0.003134	0.034147
GATA6	ENSG00000141448	3945	-0.2098	0.00203	0.026279
PSMG4	ENSG00000180822	10418	-0.208	0.000313	0.008901

APPL1	ENSG00000157500	7033	-0.20744	0.000361	0.009649
EP400	ENSG00000183495	18705	-0.20728	0.00168	0.023323
NEO1	ENSG00000067141	8117	-0.20552	0.004425	0.042107
CLASRP	ENSG00000104859	4931	-0.20547	0.001353	0.020408
HECTD4	ENSG00000173064	21572	-0.20539	0.000584	0.012728
DMXL2	ENSG00000104093	13807	-0.20521	0.000892	0.016033
SLC12A6	ENSG00000140199	10056	-0.20499	0.00012	0.005334
STK35	ENSG00000125834	7055	-0.20466	0.000258	0.008078
MIR1302-2	ENSG00000227518	2923	-0.20437	0.004295	0.041377
AKAP17A	ENSG00000197976	4075	-0.20403	0.00215	0.027142
KMT2A	ENSG00000118058	21961	-0.20381	0.000167	0.006285
AC007283.5	ENSG00000234431	445	-0.20226	0.000821	0.015253
HIVEP2	ENSG00000010818	9774	-0.2009	0.001146	0.018661
MLLT4	ENSG00000130396	13720	-0.20047	0.000626	0.013207
ING5	ENSG00000168395	8200	-0.20017	0.003719	0.037883
RP11-977G19.5	ENSG00000258199	403	-0.20011	0.004221	0.040967
MLLT1	ENSG00000130382	4740	-0.2001	0.001217	0.019274
NBPF9	ENSG00000269713	9046	-0.19963	0.002681	0.03103
DNMBP	ENSG00000107554	7072	-0.19948	0.001145	0.018652
CUX1	ENSG00000257923	17788	-0.19938	0.000133	0.005604
ATAT1	ENSG00000137343	4035	-0.19918	0.004144	0.040491
SNRNP70	ENSG00000104852	4684	-0.19891	0.002749	0.031408
RP4-781K5.2	ENSG00000228830	693	-0.19859	0.000436	0.010688
RP11-73E17.2	ENSG00000258738	2117	-0.1984	0.001348	0.020377
ATXN7L1	ENSG00000146776	8295	-0.19836	0.001129	0.018506
RERE	ENSG00000142599	11503	-0.19766	0.00575	0.04927
FAM21C	ENSG00000172661	5732	-0.19719	0.000378	0.009926
CHD2	ENSG00000279765	1506	-0.19668	0.000102	0.004972
CTDNEP1	ENSG00000175826	2584	-0.19662	0.003702	0.037765
SCAF4	ENSG00000156304	5901	-0.19631	0.000399	0.010193
SH3RF2	ENSG00000156463	7232	-0.19543	0.004055	0.039938
OPHN1	ENSG00000079482	8224	-0.19439	0.001628	0.022839
ARHGAP17	ENSG00000140750	9879	-0.19404	0.002335	0.028399
BRPF3	ENSG00000096070	6613	-0.19382	0.003541	0.036851
RREB1	ENSG00000124782	17254	-0.19373	0.000469	0.011181
RIN2	ENSG00000132669	5053	-0.19348	0.001655	0.023098
TET2	ENSG00000168769	16474	-0.19312	0.002203	0.027516
RP11-793H13.8	ENSG00000257379	1571	-0.19293	9.16E-06	0.001298
EEA1	ENSG00000102189	10150	-0.19285	0.004738	0.043836
CFLAR	ENSG00000003402	22529	-0.19244	1.44E-05	0.001659
KDM3A	ENSG00000115548	13423	-0.19243	0.003002	0.033226

TJP2	ENSG00000119139	7306	-0.19173	0.004685	0.043512
DNMBP-AS1	ENSG00000227695	2336	-0.19093	0.002114	0.02687
MAGI3	ENSG00000081026	8180	-0.19088	0.002136	0.027063
USP42	ENSG00000106346	7072	-0.19045	0.000204	0.00715
AC139887.4	ENSG00000233799	286	-0.18989	0.005432	0.047734
ZXDC	ENSG00000070476	7357	-0.18881	0.002235	0.027677
PPP1R13B	ENSG00000088808	9031	-0.18849	0.002066	0.026558
ZNF587	ENSG00000198466	7350	-0.18776	0.000635	0.013325
CTBP1-AS2	ENSG00000196810	7820	-0.18657	0.003849	0.03861
STIM2	ENSG00000109689	6909	-0.1856	0.004341	0.041581
ZNF142	ENSG00000115568	6994	-0.18539	0.005131	0.046143
ZNF814	ENSG00000204514	12600	-0.18462	0.003301	0.035168
SYNJ2	ENSG00000078269	9921	-0.18413	0.000554	0.012276
ZNF785	ENSG00000197162	4952	-0.18356	0.002883	0.032371
MPRIP	ENSG00000133030	18105	-0.18321	0.003806	0.038409
REL	ENSG00000162924	11255	-0.18318	0.003671	0.037619
AFF1	ENSG00000172493	10733	-0.18286	0.001651	0.023078
TBC1D2B	ENSG00000167202	9763	-0.1826	0.003114	0.034012
R3HDM2	ENSG00000179912	6039	-0.18163	0.001463	0.021449
GPATCH8	ENSG00000186566	9442	-0.18143	0.003551	0.036912
NFAT5	ENSG00000102908	16401	-0.18141	0.005688	0.04895
TJP1	ENSG00000104067	11340	-0.181	0.00346	0.036342
ZNF621	ENSG00000172888	8354	-0.18043	0.000893	0.016045
SMARCC2	ENSG00000139613	8555	-0.18012	3.74E-05	0.002942
DAZAP1	ENSG00000071626	9201	-0.17912	0.002083	0.026676
RP11-158M2.2	ENSG00000259544	569	-0.17893	0.002339	0.028434
SETD5	ENSG00000168137	10808	-0.17883	0.000369	0.00978
BCOR	ENSG00000183337	8544	-0.17876	0.005475	0.047906
SMG1	ENSG00000157106	25988	-0.17861	0.00011	0.005088
ZNF169	ENSG00000175787	5286	-0.17847	0.002104	0.026787
ERICH1	ENSG00000104714	9726	-0.17733	0.004688	0.043524
WNK1	ENSG00000060237	14370	-0.17649	0.000509	0.011785
STX16-NPEPL1	ENSG00000254995	3859	-0.17637	0.003321	0.035327
ATXN2	ENSG00000204842	13933	-0.17631	0.001383	0.020644
SMG7	ENSG00000116698	8317	-0.17622	0.002023	0.026236
GSE1	ENSG00000131149	10173	-0.17586	0.001618	0.022793
AHSA2	ENSG00000173209	8382	-0.1757	0.000214	0.007294
AKAP8L	ENSG00000011243	5081	-0.17436	0.001619	0.022793
C11orf49	ENSG00000149179	6693	-0.17282	0.003964	0.039383
SSBP3	ENSG00000157216	5227	-0.17247	0.002585	0.030224
LARP1	ENSG00000155506	8492	-0.17246	0.000311	0.008861

SETD8	ENSG00000183955	4579	-0.17194	0.00214	0.027075
FAM63B	ENSG00000128923	9454	-0.1717	0.001122	0.01845
SVIL	ENSG00000197321	9937	-0.17142	0.00086	0.015677
KIAA2026	ENSG00000183354	7930	-0.17074	0.000819	0.015231
CTD-2647L4.5	ENSG00000259607	585	-0.17016	0.003692	0.037725
AC004893.11	ENSG00000242687	4194	-0.16991	0.00303	0.033442
KLC1	ENSG00000126214	19251	-0.16939	0.003077	0.033748
AGO2	ENSG00000123908	4824	-0.16889	0.000746	0.014464
CREB3L2	ENSG00000182158	8788	-0.16842	0.002709	0.031252
ZNF767P	ENSG00000133624	4012	-0.16813	0.004641	0.043218
ATXN7	ENSG00000163635	9172	-0.16801	0.001339	0.020299
SAFB2	ENSG00000130254	6222	-0.16768	8.49E-05	0.004544
ARID1B	ENSG00000049618	16334	-0.16757	0.002865	0.032315
SF1	ENSG00000168066	5972	-0.16743	0.000417	0.010418
CEP350	ENSG00000135837	14301	-0.16648	0.003983	0.039503
CRIM1	ENSG00000150938	7122	-0.16647	0.004495	0.042497
ZBTB4	ENSG00000174282	6026	-0.16564	0.001671	0.023235
HMBOX1	ENSG00000147421	6634	-0.16522	0.000321	0.009043
AC083884.8	ENSG00000232729	2847	-0.16484	0.002767	0.031581
GLG1	ENSG00000260539	2466	-0.16478	0.003353	0.035557
PRRC2C	ENSG00000117523	13921	-0.16458	0.00166	0.023132
MAML1	ENSG00000161021	10690	-0.16454	0.000791	0.015041
MACF1	ENSG00000127603	43102	-0.16451	0.000656	0.013567
NKTR	ENSG00000114857	17356	-0.16449	0.001175	0.018886
SYMPK	ENSG00000125755	10628	-0.16337	0.004264	0.041215
DUSP28	ENSG00000188542	3709	-0.16259	0.003543	0.036858
LRCH1	ENSG00000136141	7307	-0.16248	0.004498	0.042497
NFATC2IP	ENSG00000176953	5738	-0.16108	0.000712	0.014085
PRRC2B	ENSG00000130723	12293	-0.16086	0.005462	0.047818
AGO4	ENSG00000134698	7267	-0.16057	0.000803	0.015146
KMT2E	ENSG00000005483	9233	-0.16038	0.003124	0.034071
C17orf85	ENSG00000074356	5360	-0.16033	0.000101	0.004971
KMT2C	ENSG00000055609	20114	-0.15746	0.000787	0.014988
PHLPP1	ENSG00000081913	9142	-0.1571	0.005795	0.0495
RP11-247I13.11	ENSG00000240591	788	-0.15662	0.00354	0.036851
PATL1	ENSG00000166889	4278	-0.15623	0.005749	0.04927
FYCO1	ENSG00000163820	8895	-0.15394	0.005169	0.046399
SFSWAP	ENSG00000061936	10157	-0.15267	0.000406	0.010306
KIF1B	ENSG00000054523	17693	-0.15181	0.00109	0.018145
TNRC6A	ENSG00000090905	12522	-0.15114	0.001955	0.025466
TRIO	ENSG00000038382	17500	-0.15096	0.005501	0.047994

RBM6	ENSG00000004534	7302	-0.15029	0.001222	0.019323
GTF2I	ENSG00000263001	10438	-0.15013	0.001473	0.021565
SP1	ENSG00000185591	8083	-0.14908	0.000571	0.012577
SUGP2	ENSG00000064607	7744	-0.14895	0.005423	0.047709
UBE2H	ENSG00000186591	6392	-0.14771	0.000811	0.015195
RBM33	ENSG00000184863	13132	-0.14755	0.003587	0.03713
TRRAP	ENSG00000196367	13218	-0.1475	0.004593	0.042883
STRN	ENSG00000115808	8345	-0.14713	0.004266	0.041215
ORAOV1	ENSG00000149716	7872	-0.14684	0.002966	0.032931
RALGAPB	ENSG00000170471	12624	-0.14676	0.001128	0.0185
AKAP1	ENSG00000121057	5399	-0.14609	0.005161	0.046354
EZH1	ENSG00000108799	6988	-0.14545	0.000913	0.016328
PARD3B	ENSG00000116117	10382	-0.14343	0.00465	0.04327
SAP130	ENSG00000136715	4373	-0.14256	0.002166	0.027253
MAP4	ENSG00000047849	14896	-0.14251	0.000617	0.013099
RC3H1	ENSG00000135870	11508	-0.14191	0.000993	0.017057
UBN1	ENSG00000118900	8551	-0.1403	0.002206	0.027538
NPEPPS	ENSG00000141279	11514	-0.14026	0.002414	0.028975
ARHGEF7	ENSG00000102606	11768	-0.13973	0.002922	0.032614
CSNK2A2	ENSG00000070770	6905	-0.13968	0.000245	0.007896
IKBKB	ENSG00000104365	10239	-0.13878	0.000785	0.014959
KDM2A	ENSG00000173120	10380	-0.13516	0.000338	0.009337
ZBTB40	ENSG00000184677	8992	-0.13482	0.001881	0.02496
IRF2BP2	ENSG00000168264	4724	-0.13438	0.004325	0.041522
RP4-761J14.8	ENSG00000219410	2399	-0.1339	0.003185	0.034436
SRPK2	ENSG00000135250	6341	-0.13158	0.00284	0.032141
ANKZF1	ENSG00000163516	6119	-0.13127	0.005571	0.048353
CTTNBP2NL	ENSG00000143079	7404	-0.13086	0.003324	0.035339
DIDO1	ENSG00000101191	13281	-0.12896	0.002347	0.028525
HNRNPUL1	ENSG00000105323	6543	-0.12865	0.000382	0.009964
TCF20	ENSG00000100207	7548	-0.12605	0.000798	0.015084
PPP2R2D	ENSG00000175470	7756	-0.12533	0.001442	0.021273
ZNF384	ENSG00000126746	5181	-0.1249	0.003389	0.035873
ARHGEF12	ENSG00000196914	24404	-0.12416	0.002533	0.029855
MECP2	ENSG00000169057	12556	-0.12318	0.003428	0.036143
ETV6	ENSG00000139083	7075	-0.123	0.004301	0.041395
ZSCAN29	ENSG00000140265	6634	-0.12123	0.001792	0.024225
YY1	ENSG00000100811	8720	-0.12006	0.003788	0.038272
SF3A1	ENSG00000099995	6329	-0.11863	0.002168	0.027261
ACIN1	ENSG00000100813	9110	-0.11651	0.000877	0.015873
TMEM131	ENSG00000075568	12985	-0.11602	0.003432	0.036144

CPSF7	ENSG00000149532	6036	-0.11261	0.003951	0.039282
ADD1	ENSG00000087274	13106	-0.11128	0.003638	0.037431
CDK13	ENSG00000065883	8921	-0.11102	0.000285	0.008535
CDK12	ENSG00000167258	12773	-0.1065	0.001012	0.017266
MARK3	ENSG00000075413	8891	-0.10309	0.000947	0.016648
KDM3B	ENSG00000120733	7961	-0.09509	0.002224	0.027667
HERC3	ENSG00000138641	8634	0.108552	0.001441	0.021273
UBR1	ENSG00000159459	9928	0.119334	0.005116	0.046071
IKBKAP	ENSG00000070061	6048	0.120166	0.005459	0.047809
ATP2C1	ENSG00000017260	8533	0.120612	0.00087	0.01583
RHBDD1	ENSG00000144468	6926	0.121893	0.004413	0.042036
SF3B3	ENSG00000189091	12419	0.124496	0.002139	0.027075
ZNF780A	ENSG00000197782	7972	0.126435	0.005259	0.046915
SMU1	ENSG00000122692	7122	0.128415	0.004435	0.042152
C11orf58	ENSG00000110696	8675	0.130141	0.004854	0.044398
TASP1	ENSG00000089123	2905	0.132446	0.002819	0.03202
SPOP	ENSG00000121067	6380	0.134158	0.002307	0.028178
SACM1L	ENSG00000211456	7352	0.134219	0.004842	0.044354
HELQ	ENSG00000163312	3713	0.138989	0.003736	0.037967
STAG1	ENSG00000118007	7846	0.139059	0.001415	0.021029
ZNHIT3	ENSG00000273611	6687	0.139739	0.00309	0.033878
CHMP3	ENSG00000115561	7183	0.140256	0.004101	0.040204
CHURC1-FNTB	ENSG00000125954	2076	0.143626	0.005357	0.047356
COPS5	ENSG00000121022	7779	0.143851	0.001948	0.025431
RPA1	ENSG00000132383	5492	0.144161	0.000431	0.010606
KLHL12	ENSG00000117153	3552	0.144849	0.003413	0.036043
DHX15	ENSG00000109606	8162	0.144906	0.004818	0.044191
ERVK3-1	ENSG00000142396	5023	0.145982	0.003153	0.034261
NDUFAF5	ENSG00000101247	7217	0.146024	0.001587	0.022488
AL136419.6	ENSG00000260669	3045	0.146337	0.003582	0.03713
ALG9	ENSG00000086848	8013	0.146675	0.001639	0.022974
SKP1	ENSG00000113558	12771	0.146937	0.004706	0.043626
EED	ENSG00000074266	7016	0.147147	0.000849	0.015523
RAD1	ENSG00000113456	6763	0.147175	0.002097	0.026732
ZNF200	ENSG00000010539	7498	0.147372	0.004208	0.040873
UBA6-AS1	ENSG00000248049	5575	0.148179	0.005121	0.046083
RBM23	ENSG00000100461	6583	0.148203	0.001004	0.017189
SMIM4	ENSG00000168273	2482	0.148286	0.003816	0.038438
PPIP5K2	ENSG00000145725	17270	0.148711	0.001975	0.025674
TMEM183A	ENSG00000163444	4026	0.148914	0.003997	0.039592
SRRD	ENSG00000100104	4319	0.14915	0.003788	0.038272

RP11-176H8.1	ENSG00000203546	4422	0.149297	0.00464	0.043218
TM2D3	ENSG00000184277	11894	0.150327	0.002747	0.0314
ASCC3	ENSG00000112249	11731	0.150452	0.005375	0.047464
RNGTT	ENSG00000111880	4797	0.1508	0.002836	0.032114
MFAP3	ENSG00000037749	4792	0.151239	0.004594	0.042883
CCNT1	ENSG00000129315	7371	0.153646	0.000958	0.016763
PDHA1	ENSG00000131828	4493	0.154233	0.005767	0.049322
YARS2	ENSG00000139131	2152	0.15509	0.00424	0.041081
DDX1	ENSG00000079785	3833	0.155606	0.002646	0.030718
MKKS	ENSG00000125863	6953	0.155772	0.003675	0.037619
CNIH1	ENSG00000100528	5060	0.156367	0.005298	0.047087
RNF146	ENSG00000118518	3508	0.156488	0.001015	0.017294
PSMC1	ENSG00000100764	6161	0.157242	0.001763	0.024003
ENOX2	ENSG00000165675	4279	0.157643	0.002443	0.029193
PGM2	ENSG00000169299	3704	0.15821	0.004921	0.044845
RPE	ENSG00000197713	3522	0.158813	0.002456	0.029279
DERL1	ENSG00000136986	7081	0.159503	0.00135	0.020377
DARS2	ENSG00000117593	3689	0.160673	0.002896	0.032426
SDHC	ENSG00000143252	14160	0.160758	0.00274	0.031391
NUP107	ENSG00000111581	8320	0.161748	0.005033	0.045525
RP11-512M8.5	ENSG00000256861	2748	0.161757	0.003701	0.037765
UBR7	ENSG00000012963	3694	0.162199	0.002565	0.030063
ERP29	ENSG00000089248	3083	0.162327	0.005526	0.048101
LARP4	ENSG00000161813	8112	0.163071	0.002235	0.027677
MTPN	ENSG00000105887	3775	0.163227	0.002909	0.032499
UNC50	ENSG00000115446	2230	0.163523	0.001911	0.025178
KLHL9	ENSG00000198642	5710	0.163569	0.000457	0.011008
NUP205	ENSG00000155561	7619	0.16357	0.001455	0.021349
CAND1	ENSG00000111530	13655	0.163798	0.000625	0.013191
UXS1	ENSG00000115652	7023	0.164198	0.004054	0.039938
AIMP1	ENSG00000164022	3328	0.164309	0.004479	0.042402
LARS	ENSG00000133706	8118	0.16433	0.004453	0.042276
DPY19L1P1	ENSG00000229358	2594	0.164349	0.001939	0.025399
SLC37A3	ENSG00000157800	10588	0.165009	0.002266	0.027865
DIABLO	ENSG00000184047	5967	0.165225	0.005646	0.048755
PEX12	ENSG00000108733	2861	0.165251	0.004948	0.04503
ZCCHC4	ENSG00000168228	4400	0.165419	0.002501	0.029681
SLC18B1	ENSG00000146409	2444	0.16555	0.001859	0.024805
INTS7	ENSG00000143493	5311	0.165779	0.001261	0.019567
PIK3R4	ENSG00000196455	5376	0.165796	0.000342	0.009389
C14orf119	ENSG00000179933	3676	0.1658	0.005602	0.048489

NUP54	ENSG00000138750	4062	0.166083	0.004503	0.042503
RBM45	ENSG00000155636	4784	0.166288	0.000305	0.008781
YME1L1	ENSG00000136758	7335	0.166397	0.004588	0.042883
SCO1	ENSG00000133028	10230	0.166451	0.003222	0.034691
RAB28	ENSG00000157869	2221	0.167124	0.001334	0.020271
WDR92	ENSG00000243667	4079	0.167228	0.005134	0.046148
KANSL2	ENSG00000139620	3386	0.167991	0.000675	0.013717
DDX18	ENSG00000088205	7851	0.168073	0.00381	0.038429
UBA5	ENSG00000081307	6827	0.168247	0.001079	0.018056
ARFIP1	ENSG00000164144	4290	0.168415	0.005413	0.047676
UROS	ENSG00000188690	6643	0.168642	0.004434	0.042152
CTC-454I21.3	ENSG00000267360	1654	0.169259	0.005551	0.048242
TMEM39A	ENSG00000176142	5333	0.16952	0.003419	0.036088
NUDT15	ENSG00000136159	2098	0.16956	0.004263	0.041215
ZNF230	ENSG00000159882	6298	0.169708	0.004044	0.039884
PARL	ENSG00000175193	2034	0.169786	0.002091	0.026712
TBC1D31	ENSG00000156787	8611	0.170278	0.001035	0.017479
POLB	ENSG00000070501	4106	0.170593	0.003442	0.03619
ZW10	ENSG00000086827	2992	0.170704	0.003111	0.034012
GTF2F2	ENSG00000188342	3619	0.170893	0.005719	0.049118
TMEM242	ENSG00000215712	4644	0.171181	0.00124	0.019434
FUNDC2	ENSG00000165775	10252	0.17121	0.001499	0.021728
DAP3	ENSG00000132676	4588	0.171288	0.002262	0.027841
MLH1	ENSG00000076242	3532	0.171314	0.001199	0.019091
COIL	ENSG00000121058	3030	0.171696	0.000939	0.016565
PEX19	ENSG00000162735	4461	0.171881	0.001359	0.020425
RP11-894J14.5	ENSG00000272305	1760	0.172022	0.005244	0.046852
ENSA	ENSG00000143420	5493	0.172327	0.000208	0.007222
DHX9	ENSG00000135829	5645	0.172368	0.000977	0.016916
GDAP1	ENSG00000104381	4052	0.172431	0.002886	0.032374
HSPA14	ENSG00000187522	4818	0.172437	0.000227	0.007589
RBM18	ENSG00000119446	5193	0.172461	0.003232	0.034723
SPTLC1	ENSG00000090054	3658	0.172523	0.004504	0.042503
TIMM10B	ENSG00000132286	3041	0.172719	0.002784	0.031721
NUPL1	ENSG00000139496	10422	0.173233	0.00317	0.034387
CMC2	ENSG00000103121	12781	0.173343	0.000525	0.01198
RP11-762I7.5	ENSG00000257390	2534	0.173691	0.002332	0.028384
PRICKLE4	ENSG00000124593	4479	0.173958	0.001017	0.017296
SYPL1	ENSG00000008282	2794	0.174358	0.004216	0.040934
MPV17	ENSG00000115204	5625	0.174976	0.004519	0.042544
RBM8A	ENSG00000265241	5241	0.175004	0.001904	0.02512

TDG	ENSG00000139372	4903	0.175038	0.003641	0.037431
RABGGTB	ENSG00000137955	5913	0.175733	0.000918	0.016367
CMTR2	ENSG00000180917	7582	0.175941	0.004693	0.043546
TMEM223	ENSG00000168569	2280	0.176134	0.003098	0.033913
SRPK1	ENSG00000096063	6692	0.176184	0.001834	0.024589
TMEM30A	ENSG00000112697	5123	0.176386	0.002928	0.032662
ANAPC7	ENSG00000196510	6009	0.176648	0.0003	0.008696
SDHAF2	ENSG00000167985	2492	0.176753	0.000577	0.012663
RP5-1180E21.5	ENSG00000273221	647	0.176822	0.003106	0.033964
ORC5	ENSG00000164815	3724	0.177275	0.001208	0.019167
TMEM50B	ENSG00000142188	4495	0.177853	0.001216	0.019269
CHUK	ENSG00000213341	6928	0.177998	0.003508	0.036679
EEF1D	ENSG00000104529	9390	0.178086	0.004561	0.042762
COMMD9	ENSG00000110442	6592	0.178495	0.002402	0.028888
RTFDC1	ENSG00000022277	4063	0.179053	0.000829	0.015324
SLC25A17	ENSG00000100372	3989	0.17913	0.001165	0.018836
TMEM167B	ENSG00000215717	4574	0.179148	0.003789	0.038272
APOLD1	ENSG00000178878	7243	0.179354	0.002968	0.032931
BRK1	ENSG00000254999	1233	0.179419	0.003072	0.033706
ANP32A	ENSG00000140350	7544	0.179704	0.002879	0.032351
ZC3HC1	ENSG00000091732	3011	0.179769	0.004524	0.04256
SNRPD3	ENSG00000100028	2544	0.180487	0.001361	0.020437
ATP10D	ENSG00000145246	7965	0.180916	0.001488	0.021654
TMED10	ENSG00000170348	4974	0.180945	0.00337	0.035698
MALSU1	ENSG00000156928	3851	0.1812	0.000878	0.015873
KARS	ENSG00000065427	4065	0.181243	0.001785	0.024171
USP46	ENSG00000109189	8917	0.181326	0.000401	0.010237
ACTR6	ENSG00000075089	3813	0.181405	0.005003	0.045352
MTO1	ENSG00000135297	12071	0.181517	0.00585	0.049804
UAP1	ENSG00000117143	3437	0.181844	0.002263	0.027841
RFT1	ENSG00000163933	5488	0.182015	0.000475	0.011267
ADSS	ENSG00000035687	3666	0.182125	0.005748	0.04927
MFSD1	ENSG00000118855	5288	0.182173	0.00021	0.007234
PPA2	ENSG00000138777	4654	0.182279	0.003773	0.038204
RUVBL1	ENSG00000175792	6860	0.182399	0.005522	0.048089
APTX	ENSG00000137074	3789	0.182478	0.00024	0.007839
C8orf33	ENSG00000182307	3378	0.182571	0.005344	0.047319
COMMD10	ENSG00000145781	2464	0.183336	0.001124	0.018464
MRFAP1L1	ENSG00000178988	2180	0.183598	0.000153	0.005998
TMEM179B	ENSG00000185475	1496	0.183707	0.002559	0.030015
PTRH2	ENSG00000141378	4740	0.183753	0.000167	0.006285

MRPS18C	ENSG00000163319	4866	0.184028	0.004507	0.042503
AASDHPPT	ENSG00000149313	6183	0.18428	0.000946	0.01664
MOB4	ENSG00000115540	4296	0.184344	0.004685	0.043512
PRKAG1	ENSG00000181929	4556	0.184366	0.002441	0.029183
GPN1	ENSG00000198522	3746	0.184411	8.1E-05	0.004446
NIPSNAP3A	ENSG00000136783	2585	0.184773	0.003824	0.038482
APITD1	ENSG00000175279	1739	0.184817	0.003208	0.034581
CLPTM1L	ENSG00000049656	6557	0.185657	0.00087	0.01583
CTC-498J12.3	ENSG00000248664	946	0.185864	0.001803	0.024323
ITGB3BP	ENSG00000142856	5459	0.185956	0.000564	0.012466
UFC1	ENSG00000143222	2274	0.18641	0.002434	0.029152
MIR3654	ENSG00000255508	5197	0.186691	0.003283	0.035049
PTGES3	ENSG00000110958	3399	0.187093	5.74E-05	0.00374
TM9SF1	ENSG00000254692	2740	0.187115	0.002188	0.027401
RAD17	ENSG00000152942	4159	0.187188	0.001678	0.023306
EEF1G	ENSG00000254772	2716	0.187482	0.003347	0.035524
NPRL2	ENSG00000114388	3677	0.187555	0.003985	0.039503
NOL11	ENSG00000130935	4713	0.187792	0.002735	0.031391
ARL14EP	ENSG00000152219	5465	0.187943	0.005757	0.049296
MRPS22	ENSG00000175110	5382	0.187956	0.002782	0.031705
MED18	ENSG00000130772	2019	0.188092	0.003609	0.037219
SRP54	ENSG00000100883	3899	0.188199	0.005433	0.047734
PDHX	ENSG00000110435	3760	0.188282	0.004514	0.042518
NECAP1	ENSG00000089818	4155	0.188324	0.005844	0.049804
TMEM87A	ENSG00000103978	5668	0.188684	4.15E-05	0.003099
RP4-769N13.7	ENSG00000270050	387	0.188706	0.002449	0.029242
PSMC5	ENSG00000087191	4490	0.188799	0.003773	0.038204
PPP1R8	ENSG00000117751	2819	0.188911	0.000741	0.014437
SNRNP40	ENSG00000060688	3881	0.188955	0.001014	0.017293
C14orf166	ENSG00000087302	8323	0.189238	0.002141	0.027076
SUPT4H1	ENSG00000213246	2079	0.189289	0.000679	0.013759
RNF139	ENSG00000170881	2700	0.189323	0.001518	0.021908
ERI3	ENSG00000117419	2642	0.189599	0.000534	0.012074
NGRN	ENSG00000182768	7694	0.189606	0.001273	0.019703
OSTF1	ENSG00000134996	1319	0.189657	0.004059	0.03995
ZMAT2	ENSG00000146007	1812	0.189731	0.000963	0.016814
HNRNPA1	ENSG00000135486	5093	0.189738	0.000301	0.008715
RP11-26J3.4	ENSG00000276418	1593	0.189814	0.003563	0.037022
RP11-315I20.1	ENSG00000234222	3175	0.18992	0.004586	0.042883
REEP5	ENSG00000129625	4230	0.189992	0.00051	0.011785
DERL2	ENSG00000072849	6386	0.190431	0.002177	0.027344

SNUPN	ENSG00000169371	3516	0.190442	0.002116	0.026871
TSG101	ENSG00000074319	3839	0.191114	0.00286	0.032288
RIOK2	ENSG00000058729	5502	0.191128	0.002137	0.027063
HSDL1	ENSG00000103160	4507	0.191243	0.005323	0.047204
SAMM50	ENSG00000100347	6435	0.191326	0.002223	0.027667
ARMC1	ENSG00000104442	3421	0.191506	0.000854	0.015591
BRE	ENSG00000158019	3392	0.191527	0.005757	0.049296
BCCIP	ENSG00000107949	4498	0.19166	0.002941	0.032764
RPP30	ENSG00000148688	4653	0.191878	0.002923	0.032614
POP4	ENSG00000105171	6574	0.191959	0.000522	0.011931
SAE1	ENSG00000142230	3396	0.192482	0.004165	0.040615
PPIE	ENSG00000084072	8071	0.192534	0.002364	0.028674
TMEM165	ENSG00000134851	7045	0.192718	0.001166	0.018836
AC010642.1	ENSG00000267216	4227	0.192925	0.000116	0.005253
MAPRE1	ENSG00000101367	2623	0.193156	0.001663	0.023161
UBE2L3	ENSG00000185651	3838	0.19353	0.00295	0.032817
MGAT2	ENSG00000168282	2687	0.193748	0.001744	0.023879
PIGU	ENSG00000101464	2609	0.193755	0.005329	0.047234
RPL22	ENSG00000116251	4900	0.193898	0.004707	0.043626
RP11-21J18.1	ENSG00000265257	1050	0.194283	0.004027	0.03979
FAM114A2	ENSG00000055147	6543	0.194552	0.000749	0.014487
MRPS14	ENSG00000120333	2594	0.194617	0.000566	0.012509
RP11-649E7.5	ENSG00000258377	2469	0.1947	0.001653	0.02309
VWA9	ENSG00000138614	4210	0.194824	7.21E-05	0.004182
COQ5	ENSG00000110871	2523	0.195057	0.004296	0.041377
RP11-336A10.2	ENSG00000226647	1412	0.195151	0.001178	0.018908
PPP2R3C	ENSG00000092020	3863	0.195343	0.002778	0.03169
NGDN	ENSG00000129460	4654	0.195532	0.00022	0.007424
LHFPL5	ENSG00000197753	2961	0.19588	0.000877	0.015873
TFAM	ENSG00000108064	5606	0.195915	0.000936	0.016547
KDELR2	ENSG00000136240	3990	0.196009	0.001756	0.023967
IFT52	ENSG00000101052	2542	0.196092	0.005569	0.04835
LBHD1	ENSG00000162194	3362	0.196281	0.001086	0.018105
EXOSC1	ENSG00000171311	3345	0.196356	0.0014	0.020855
CANX	ENSG00000127022	6235	0.196704	0.005818	0.049601
ATP6V1A	ENSG00000114573	5130	0.196763	0.000755	0.014559
CTD-2545G14.6	ENSG00000279641	1615	0.197359	0.001204	0.019123
HNRNPC	ENSG00000092199	7309	0.197588	0.001357	0.020422
MRPL48	ENSG00000175581	4386	0.197619	0.003185	0.034436
ALG6	ENSG00000088035	4950	0.197628	0.001487	0.021654
TEFM	ENSG00000172171	3224	0.197724	0.000271	0.008275

FOCAD	ENSG00000188352	10036	0.197732	0.001429	0.021175
RNF5	ENSG00000204308	1178	0.198041	0.004923	0.044854
FTSJ2	ENSG00000122687	2843	0.19822	0.000567	0.012511
NDUFV2	ENSG00000178127	2711	0.199284	0.003451	0.036257
CCDC84	ENSG00000186166	5105	0.199437	0.000156	0.006081
ZCCHC17	ENSG00000121766	1843	0.19949	0.00094	0.016574
WDR41	ENSG00000164253	7230	0.199967	0.000456	0.011003
RPL5	ENSG00000122406	2890	0.20032	0.004813	0.044162
DDX50	ENSG00000107625	2692	0.200348	0.000464	0.011105
CUTA	ENSG00000112514	1876	0.200471	0.001576	0.022395
ATXN10	ENSG00000130638	5098	0.200608	0.000344	0.009424
CLN5	ENSG00000102805	4226	0.200733	0.001357	0.020422
ELP6	ENSG00000163832	3313	0.200854	0.000603	0.012899
MRPS17	ENSG00000249773	1732	0.200933	0.004883	0.044594
RNMTL1	ENSG00000171861	2065	0.201375	0.00586	0.049868
DLD	ENSG00000091140	5299	0.201377	0.001137	0.018552
NFU1	ENSG00000169599	2197	0.201402	0.005145	0.046228
MIF4GD	ENSG00000125457	2283	0.201465	0.004412	0.042036
WDR47	ENSG00000085433	4423	0.20156	0.000388	0.010037
TSN	ENSG00000211460	4407	0.201816	0.000134	0.005606
ARV1	ENSG00000173409	1987	0.201849	0.004579	0.042832
RP11-697E2.6	ENSG00000261147	3838	0.202113	0.001264	0.019589
SLC39A9	ENSG00000029364	7136	0.202193	0.002458	0.029283
WDR61	ENSG00000140395	7279	0.202207	3.79E-05	0.00296
BTBD3	ENSG00000132640	6228	0.20227	0.005412	0.047676
COMMD2	ENSG00000114744	4901	0.202371	0.003753	0.038105
RP11-546B15.1	ENSG00000261002	988	0.202656	0.000543	0.012171
ZNF627	ENSG00000198551	3827	0.202765	0.000107	0.005039
RP11-770J1.5	ENSG00000254873	2395	0.203153	0.000668	0.013662
SPCS1	ENSG00000114902	5324	0.203443	0.002184	0.027382
PFDN6	ENSG00000204220	2953	0.203598	0.001427	0.021165
COX4I1	ENSG00000131143	4792	0.203778	0.004189	0.040762
CTNNB1	ENSG00000168036	6614	0.203875	0.004066	0.039999
MAGT1	ENSG00000102158	4862	0.204018	0.003927	0.039114
TMEM251	ENSG00000153485	1365	0.20446	0.003683	0.037655
VKORC1L1	ENSG00000196715	5899	0.204629	0.004272	0.041239
POLR2C	ENSG00000102978	3940	0.204648	0.000757	0.014574
EEF1A1P19	ENSG00000249855	1382	0.204677	0.001864	0.024805
MIPEP	ENSG00000027001	3407	0.204803	0.000694	0.0139
RP5-1014D13.2	ENSG00000279738	19416	0.204868	0.00322	0.034688
GAS2	ENSG00000148935	3529	0.204946	0.004113	0.040272

TTC1	ENSG00000113312	1938	0.204961	0.000685	0.013817
FDXACB1	ENSG00000255561	3551	0.205017	0.005728	0.049169
COPS6	ENSG00000168090	2807	0.205201	0.000179	0.006587
COPZ1	ENSG00000111481	3901	0.205263	0.001053	0.017736
NAGA	ENSG00000198951	3706	0.205584	0.003648	0.037479
APMAP	ENSG00000101474	2454	0.205728	0.001607	0.022724
RWDD1	ENSG00000111832	5909	0.205784	0.004025	0.039789
COPS3	ENSG00000141030	2788	0.205847	0.001633	0.02289
PSMB7	ENSG00000136930	1787	0.206228	0.003519	0.036733
VAMP8	ENSG00000118640	990	0.206356	0.005359	0.047356
AC144449.1	ENSG00000231969	1022	0.206728	0.001769	0.024017
UTP6	ENSG00000108651	6450	0.206786	2.74E-05	0.002487
AC091133.1	ENSG00000230532	483	0.206886	0.001261	0.019567
CYB5B	ENSG00000103018	6903	0.207038	0.001845	0.024697
METTL5	ENSG00000138382	2865	0.207477	0.003585	0.03713
ANKRA2	ENSG00000164331	2659	0.207697	0.002414	0.028975
SYNJ2BP-COX16	ENSG00000258644	1404	0.20771	0.000675	0.013717
TXNDC12	ENSG00000117862	3630	0.207795	0.000309	0.008834
LYRM2	ENSG00000083099	8679	0.208032	0.00071	0.01408
MTMR6	ENSG00000139505	5779	0.208411	0.003446	0.036227
AC009120.6	ENSG00000259972	3297	0.208541	0.000494	0.0116
JAGN1	ENSG00000171135	1799	0.208551	0.004307	0.041419
ARHGAP19	ENSG00000213390	6167	0.208558	0.005382	0.04747
KBTBD7	ENSG00000120696	4734	0.208698	0.000336	0.009311
L3MBTL2	ENSG00000100395	6451	0.208799	0.000325	0.00912
ATRAID	ENSG00000138085	2019	0.209112	0.000979	0.016923
HEXB	ENSG00000049860	5067	0.209153	0.003812	0.038429
TIMM21	ENSG00000075336	5208	0.209567	0.003324	0.035339
PPCS	ENSG00000127125	2590	0.209586	0.001672	0.023241
PRUNE	ENSG00000143363	3063	0.210016	0.000227	0.007589
UMPS	ENSG00000114491	3499	0.210372	8E-05	0.004442
CTD-2540L5.5	ENSG00000259115	611	0.210633	0.000115	0.00522
CAPZA1	ENSG00000116489	4130	0.210684	0.004781	0.044011
LINC00339	ENSG00000218510	1424	0.210728	0.005037	0.045544
HMGN2	ENSG00000198830	2509	0.210831	0.000544	0.012171
LZIC	ENSG00000162441	2794	0.210999	1.96E-05	0.002005
AARSD1	ENSG00000266967	3704	0.21112	0.004014	0.039715
CTC-487M23.8	ENSG00000272869	2174	0.211245	0.003162	0.034337
ANXA7	ENSG00000138279	2862	0.211385	0.00072	0.014202
CCDC115	ENSG00000136710	3782	0.211487	0.001199	0.019091
LSM4	ENSG00000130520	3029	0.21156	0.002615	0.030504

CSTF1	ENSG00000101138	4800	0.211675	4.91E-05	0.003429
SFT2D1	ENSG00000198818	3104	0.211744	3.95E-05	0.003008
NAA20	ENSG00000173418	2376	0.211808	0.00013	0.005521
TWISTNB	ENSG00000105849	3997	0.211943	0.005745	0.04927
EI24	ENSG00000149547	3558	0.211956	0.000114	0.00519
NELFE	ENSG00000204356	2719	0.212001	0.00393	0.039131
M6PR	ENSG00000003056	3777	0.212245	0.000129	0.005521
EIF3H	ENSG00000147677	6434	0.212363	0.000351	0.009525
C11orf73	ENSG00000149196	3421	0.212434	0.000214	0.007294
UGP2	ENSG00000169764	6495	0.21257	0.001864	0.024805
MED31	ENSG00000108590	2103	0.212941	0.005705	0.049053
COA7	ENSG00000162377	4165	0.213044	0.004495	0.042497
MNAT1	ENSG00000020426	3865	0.213056	0.002273	0.02792
TIMMDC1	ENSG00000113845	2730	0.213329	6.26E-05	0.003815
PPIH	ENSG00000171960	1444	0.213532	0.002132	0.027036
RAP1GDS1	ENSG00000138698	5424	0.214047	0.000107	0.005039
URM1	ENSG00000167118	4410	0.214566	0.005508	0.047998
XRCC5	ENSG00000079246	5463	0.214625	0.000379	0.00993
C4orf46	ENSG00000205208	3730	0.214965	0.004413	0.042036
G3BP2	ENSG00000138757	7183	0.215103	0.003717	0.037875
FASTKD1	ENSG00000138399	4497	0.215112	0.000158	0.006106
MRPS21	ENSG00000266472	1641	0.215154	0.000613	0.013033
CCT5	ENSG00000150753	5839	0.215228	0.001759	0.023979
ATP6AP2	ENSG00000182220	2573	0.215573	0.000145	0.005806
SAR1A	ENSG00000079332	6914	0.215829	0.000957	0.016763
TMEM59	ENSG00000116209	4167	0.215835	0.001585	0.022482
RNF25	ENSG00000163481	2323	0.215841	0.001333	0.020271
NUDCD1	ENSG00000120526	4439	0.215886	0.00358	0.03713
CGRRF1	ENSG00000100532	4564	0.216049	0.001435	0.021212
ADH5	ENSG00000197894	4866	0.216433	0.00205	0.026467
ESD	ENSG00000139684	3401	0.216445	0.001329	0.02026
STRAP	ENSG00000023734	2211	0.216484	0.000937	0.016547
MRPL57	ENSG00000173141	2246	0.216528	0.000919	0.016372
RP11-661A12.7	ENSG00000254741	431	0.216541	0.001149	0.018671
RP11-677I18.3	ENSG00000254433	1247	0.216789	0.001052	0.017728
ILF2	ENSG00000143621	2281	0.216813	0.004694	0.043546
TIMM23	ENSG00000265354	1222	0.216929	0.002233	0.027677
MRPL33	ENSG00000243147	1539	0.217065	0.001915	0.025191
MTX2	ENSG00000128654	1717	0.217102	1.85E-05	0.001937
DCLRE1A	ENSG00000198924	4600	0.217203	0.000466	0.011131
NUDT5	ENSG00000165609	4969	0.217223	1.54E-05	0.00175

RP11-15L13.4	ENSG00000272910	501	0.217223	0.001893	0.025074
RPS6	ENSG00000137154	2714	0.217246	0.002261	0.027841
PSMA1	ENSG00000129084	3301	0.21734	0.001767	0.024008
PTGES3L-AARSD1	ENSG00000108825	2383	0.217376	0.002706	0.031232
GOT2	ENSG00000125166	3766	0.217445	0.000578	0.012681
SLC17A5	ENSG00000119899	3604	0.217503	0.004452	0.042275
ACTL6A	ENSG00000136518	3675	0.21782	0.000426	0.010548
DTD1	ENSG00000125821	3329	0.217836	0.003069	0.033698
SSBP1	ENSG00000106028	4988	0.217859	0.000685	0.013817
MED8	ENSG00000159479	2704	0.217885	0.002992	0.033137
RPS8	ENSG00000142937	2847	0.21805	0.004543	0.042686
MRPL35	ENSG00000132313	3829	0.218113	0.00093	0.016503
PPP1R42	ENSG00000178125	2250	0.218302	0.001242	0.019464
HCCS	ENSG00000004961	2335	0.218398	0.00105	0.017697
CREG1	ENSG00000143162	2688	0.219005	0.001169	0.01884
CCT4	ENSG00000115484	2830	0.219309	0.0001	0.004953
SNX11	ENSG00000002919	3606	0.219859	0.001176	0.018886
DDX3X	ENSG00000215301	8881	0.219938	0.005267	0.046925
RPL18	ENSG00000063177	4209	0.220075	0.004492	0.042497
GRPEL2	ENSG00000164284	4419	0.220347	0.003072	0.033706
COQ2	ENSG00000173085	2774	0.220443	0.001492	0.021692
SAP18	ENSG00000150459	3396	0.220744	0.000706	0.014023
RP11-644F5.10	ENSG00000258311	3510	0.22075	0.003924	0.039112
LMAN2L	ENSG00000114988	2709	0.220755	0.001898	0.025099
CDC123	ENSG00000151465	2404	0.220869	0.000212	0.007266
OMA1	ENSG00000162600	3718	0.220977	0.005351	0.047351
GMFB	ENSG00000197045	7966	0.221045	0.003141	0.034177
SRP9P1	ENSG00000180581	261	0.221246	0.002822	0.03202
PSMC6	ENSG00000100519	7482	0.221306	0.000382	0.009964
ABT1	ENSG00000146109	2949	0.221517	0.000424	0.01053
XRCC6	ENSG00000196419	3288	0.221822	0.000342	0.009378
SUMO2P17	ENSG00000248278	897	0.222029	0.001854	0.024756
CTSL	ENSG00000135047	2366	0.222162	0.002888	0.032374
ISCA2	ENSG00000165898	3061	0.222193	0.000107	0.005039
RP11-69L16.4	ENSG00000238221	500	0.222204	0.001261	0.019567
LSM2	ENSG00000204392	1077	0.222301	0.005748	0.04927
PRPS1	ENSG00000147224	2280	0.222568	0.004405	0.042036
ATP5F1	ENSG00000116459	3383	0.222581	0.004098	0.0402
PSMD7	ENSG00000103035	2740	0.222663	0.001593	0.02256
EIF1AXP1	ENSG00000236698	435	0.222705	0.003318	0.035311

CISD1	ENSG00000122873	2857	0.2229	0.005313	0.047152
PTPMT1	ENSG00000110536	2740	0.222904	0.000484	0.011407
HNRNPH2	ENSG00000126945	2223	0.222937	0.000835	0.015364
ANAPC16	ENSG00000166295	4324	0.223002	0.000424	0.010531
TMEM98	ENSG00000006042	5187	0.223045	0.000457	0.011009
NABP2	ENSG00000139579	2218	0.223166	0.003188	0.034456
LRRC75A-AS1	ENSG00000175061	3759	0.223185	0.002554	0.030001
EIF2B3	ENSG00000070785	3411	0.22331	0.001586	0.022488
FASTKD2	ENSG00000118246	6079	0.22354	0.000273	0.008331
BET1	ENSG00000105829	5247	0.223581	0.005074	0.045816
AAGAB	ENSG00000103591	3833	0.223689	0.00105	0.017697
TMEM205	ENSG00000105518	1606	0.223806	0.004609	0.042991
DHRS7B	ENSG00000109016	5544	0.223822	0.003295	0.035126
MRPS23	ENSG00000181610	6379	0.223887	0.001102	0.018281
CMTM7	ENSG00000153551	3751	0.224621	0.001106	0.01831
TEX30	ENSG00000151287	2154	0.22505	0.003525	0.036762
RASL11A	ENSG00000122035	1624	0.225075	0.00572	0.049118
SRSF3	ENSG00000112081	7118	0.22526	0.000343	0.009401
RP11-486O12.2	ENSG00000247373	8279	0.225458	0.00207	0.026558
RP11-831H9.11	ENSG00000255432	577	0.22576	0.001131	0.018509
PSMD13	ENSG00000185627	4767	0.225906	0.00039	0.010071
LAMTOR1	ENSG00000149357	2778	0.226369	0.001531	0.022017
POLR3B	ENSG00000013503	4858	0.22638	0.001058	0.017798
MDP1	ENSG00000213920	1072	0.226425	0.000315	0.008954
SMDT1	ENSG00000183172	1731	0.22644	0.001917	0.025202
MRPS16	ENSG00000182180	2794	0.226633	0.000464	0.011105
PPP1R7	ENSG00000115685	3781	0.226695	0.000157	0.006092
PRELID2	ENSG00000186314	5375	0.227035	0.002294	0.028086
FBXL5	ENSG00000118564	4573	0.227325	0.000895	0.016068
C1GALT1C1	ENSG00000171155	1785	0.227363	0.000702	0.013974
MSANTD4	ENSG00000170903	5105	0.227441	0.000475	0.011267
RP4-734P14.4	ENSG00000256566	823	0.227619	0.001312	0.020085
SSR2	ENSG00000163479	6651	0.22773	0.001082	0.018096
CFDP1	ENSG00000153774	3317	0.227857	0.001462	0.021442
CAP1	ENSG00000131236	3814	0.227885	0.003486	0.036524
ASAH1	ENSG00000104763	5051	0.22798	0.000381	0.009956
PSAP	ENSG00000197746	3224	0.2283	0.002075	0.026592
TSSC1	ENSG00000032389	5775	0.228401	0.004433	0.042152
SCMH1	ENSG00000010803	4447	0.228415	0.003183	0.034436
TCEB1	ENSG00000154582	4934	0.228476	0.005204	0.046594
ISOC1	ENSG00000066583	1940	0.228487	0.000298	0.008696

RANGRF	ENSG00000108961	1577	0.228572	0.002228	0.027667
PTRHD1	ENSG00000184924	1917	0.228686	0.002137	0.027063
FARSA	ENSG00000179115	2435	0.229191	0.001547	0.022148
SEC61A1	ENSG00000058262	4697	0.229304	0.001246	0.019494
ATP5A1	ENSG00000152234	10908	0.22946	0.003726	0.037892
CTSC	ENSG00000109861	11421	0.229493	0.004364	0.04173
YWHAE	ENSG00000108953	3847	0.229656	0.000141	0.005755
TOMM6	ENSG00000214736	637	0.229816	0.000292	0.008642
C17orf49	ENSG00000258315	2628	0.22982	0.002181	0.02738
WDR83	ENSG00000123154	2360	0.229902	0.000971	0.016885
MIR1282	ENSG00000221792	101	0.230417	0.002857	0.032273
SRP9	ENSG00000143742	1728	0.230427	0.000796	0.015076
UQCRFS1	ENSG00000169021	3475	0.230628	0.002246	0.027751
CFAP20	ENSG00000070761	2375	0.230992	0.002331	0.028384
SNAPC5	ENSG00000174446	2509	0.231155	3.6E-05	0.002905
RNASEK	ENSG00000219200	1789	0.231286	0.002229	0.027667
THNSL1	ENSG00000185875	3788	0.231845	0.002874	0.032351
POLR2I	ENSG00000105258	1269	0.232025	0.005374	0.047464
DHRS12	ENSG00000102796	3525	0.232214	0.004709	0.043633
ZBTB6	ENSG00000186130	4106	0.232306	0.000275	0.008346
ERGIC3	ENSG00000125991	6012	0.232345	0.000254	0.008018
CDK5	ENSG00000164885	2939	0.232566	0.003588	0.03713
LRR1	ENSG00000165501	2359	0.23309	0.001098	0.018239
ORMDL2	ENSG00000123353	2810	0.233204	0.003903	0.038988
CHCHD3	ENSG00000106554	3718	0.233207	0.001646	0.023017
WDR25	ENSG00000176473	4218	0.233264	0.000402	0.01025
LAPTM4A	ENSG00000068697	1946	0.233323	2.33E-05	0.002188
AD001527.4	ENSG00000279504	785	0.233373	0.00485	0.044398
RP11-258F1.2	ENSG00000279428	3130	0.233455	0.004244	0.041087
OXSM	ENSG00000151093	2486	0.233463	0.00023	0.007647
ADSL	ENSG00000239900	5632	0.233472	0.000643	0.013429
CSE1L	ENSG00000124207	3800	0.233623	0.001873	0.024881
RPL11	ENSG00000142676	1987	0.233641	0.002427	0.029091
POLE4	ENSG00000115350	2151	0.233916	0.001687	0.023357
C11orf31	ENSG00000211450	2305	0.234066	0.003517	0.036726
UXT-AS1	ENSG00000267064	1545	0.234174	0.00414	0.040469
SEC11A	ENSG00000140612	5748	0.234706	0.000105	0.00502
GFM1	ENSG00000168827	9032	0.23475	0.000119	0.005312
RP11-661A12.8	ENSG00000279605	4947	0.23505	0.001006	0.017216
CLP1	ENSG00000172409	2793	0.235199	0.000406	0.010306
TRAPPC13	ENSG00000113597	7049	0.235377	5.32E-05	0.00359

EIF1AX	ENSG00000173674	4427	0.235439	0.001171	0.018861
COPS8	ENSG00000198612	4743	0.235682	0.002732	0.031391
MPC2	ENSG00000143158	2552	0.235772	0.000667	0.013662
COMMD3	ENSG00000148444	3317	0.236038	0.000206	0.007163
TMED2	ENSG00000086598	3830	0.236069	0.001685	0.023357
COPB2	ENSG00000184432	8177	0.236148	0.000678	0.013755
SEP15	ENSG00000183291	2103	0.236184	9.08E-05	0.004731
MIR3655	ENSG00000264052	83	0.236231	0.000328	0.009194
C11orf98	ENSG00000278615	887	0.236527	0.001482	0.021615
PFDN5	ENSG00000123349	3380	0.236686	0.000544	0.012171
SDF2	ENSG00000132581	3332	0.236725	0.000377	0.009926
FAM209A	ENSG00000124103	1137	0.236749	0.000445	0.010835
AP2S1	ENSG00000042753	1704	0.236778	0.005307	0.047152
MYL12A	ENSG00000101608	3245	0.236797	0.003061	0.033666
RP11-411B6.6	ENSG00000255339	2139	0.237034	0.003501	0.03665
HIST1H2AC	ENSG00000180573	1668	0.237353	0.002872	0.032351
JKAMP	ENSG00000050130	4350	0.237613	2.07E-05	0.002082
PSMB2	ENSG00000126067	4843	0.237679	0.000206	0.007163
FAM220A	ENSG00000178397	2395	0.237744	0.002789	0.031764
NSF	ENSG00000073969	5005	0.237818	1.04E-05	0.001378
RPL3	ENSG00000100316	4787	0.238001	0.001841	0.024662
PCMT1	ENSG00000120265	2832	0.23802	0.000185	0.006709
RP11-802O23.3	ENSG00000272182	561	0.238039	0.000209	0.007222
ZNF576	ENSG00000124444	3481	0.238236	0.000119	0.005321
DDOST	ENSG00000244038	2721	0.238275	0.001113	0.018381
RP11-10L12.4	ENSG00000246560	883	0.238318	0.003772	0.038204
DCTN3	ENSG00000137100	3740	0.238344	0.000796	0.015076
TSPAN15	ENSG00000099282	2646	0.238402	0.002781	0.031702
DRG1	ENSG00000185721	2113	0.238527	5.45E-05	0.003663
AAR2	ENSG00000131043	3101	0.238608	0.000347	0.00947
SNRPB2	ENSG00000125870	2607	0.239578	0.000971	0.016885
AC073610.5	ENSG00000255863	406	0.239593	0.000727	0.014282
RPL14	ENSG00000188846	2409	0.239707	0.002457	0.029283
RNASEK-C17orf49	ENSG00000161939	1947	0.239723	0.000653	0.01355
SRP14	ENSG00000140319	2005	0.239927	0.000177	0.006546
ST13P19	ENSG00000228110	1124	0.239931	0.005424	0.047709
HSPA9	ENSG00000113013	4751	0.239958	0.002086	0.026693
ACTR10	ENSG00000131966	4039	0.239969	0.000804	0.015148
TMEM199	ENSG00000244045	4620	0.239989	0.000255	0.008025
SLC5A6	ENSG00000138074	5606	0.240126	0.004338	0.041573
YY1AP1	ENSG00000163374	5001	0.240284	0.001481	0.021614

TPI1	ENSG00000111669	2700	0.240579	0.002377	0.028733
ATG4A	ENSG00000101844	2927	0.240598	0.005487	0.047931
CTD-2256P15.4	ENSG00000271980	2550	0.240744	0.000744	0.01445
MFSD5	ENSG00000182544	2581	0.240985	0.002381	0.028733
PPIA	ENSG00000196262	4432	0.241	0.000292	0.008642
ATP5C1	ENSG00000165629	2326	0.241421	0.00087	0.01583
UXT	ENSG00000126756	1243	0.241607	0.000345	0.009432
NACA	ENSG00000196531	10008	0.241805	0.001357	0.020422
EIF3E	ENSG00000104408	6005	0.241855	0.00559	0.048439
ATP5J2-PTCD1	ENSG00000248919	2859	0.24201	0.002489	0.029577
EMC3	ENSG00000125037	4270	0.242012	0.000171	0.006394
NARS	ENSG00000134440	4089	0.242307	0.001606	0.022724
NSL1	ENSG00000117697	4006	0.242529	0.002074	0.026592
VDAC3	ENSG00000078668	3141	0.242552	0.000243	0.007859
ZMAT5	ENSG00000100319	3200	0.242578	0.005205	0.046594
RP11-446E9.1	ENSG00000236814	1170	0.242578	0.00326	0.03489
LINC00493	ENSG00000232388	1098	0.242695	0.002977	0.03299
RPS9	ENSG00000170889	4371	0.242759	0.005382	0.04747
SLC25A3	ENSG00000075415	8896	0.242856	0.00126	0.019567
PDHB	ENSG00000168291	4717	0.242869	0.000177	0.006546
PDZD11	ENSG00000120509	1393	0.243117	0.000803	0.015146
MARS2	ENSG00000247626	3019	0.243136	0.002381	0.028733
USO1	ENSG00000138768	4588	0.243281	0.005509	0.047998
CHID1	ENSG00000177830	5984	0.243355	0.002042	0.026397
CHMP2A	ENSG00000130724	2129	0.243473	0.004311	0.041448
ATP5I	ENSG00000169020	940	0.243664	0.003487	0.036524
NDUFB11	ENSG00000147123	1418	0.2438	0.002538	0.029879
TMA7	ENSG00000232112	1139	0.243991	0.002688	0.031105
CCT6A	ENSG00000146731	3901	0.244005	0.000136	0.005622
NDUFS3	ENSG00000213619	3670	0.244056	0.002066	0.026558
ARMCX6	ENSG00000198960	2489	0.244205	0.000883	0.01593
CTD-2545G14.7	ENSG00000262526	776	0.244393	1.73E-05	0.001869
RPS15	ENSG00000115268	2137	0.24452	0.002949	0.032817
GLT8D1	ENSG00000016864	3916	0.244798	0.001936	0.025387
PGK1	ENSG00000102144	5415	0.245175	0.000253	0.008014
AC002398.9	ENSG00000188223	1037	0.245245	0.003066	0.033692
GMNN	ENSG00000112312	2476	0.24528	0.000366	0.009751
NIPA2	ENSG00000140157	3505	0.245425	0.000627	0.013207
RRM1	ENSG00000167325	4177	0.245453	0.000602	0.012895
PSMA3	ENSG00000100567	3119	0.245682	0.000483	0.011392
GEMIN2	ENSG00000092208	2532	0.24572	0.00069	0.013872

MRPL18	ENSG00000112110	1375	0.245949	0.001363	0.020454
ATP5B	ENSG00000110955	2322	0.245981	0.001606	0.022724
LINC01420	ENSG00000204272	1529	0.246548	0.001305	0.020029
MRPL22	ENSG00000082515	5267	0.246549	9.2E-05	0.004764
RPL15	ENSG00000174748	6528	0.246569	0.000301	0.008715
KBTBD4	ENSG00000123444	3302	0.246641	0.000188	0.006754
RP11-193F5.1	ENSG00000258892	200	0.247023	0.000818	0.015231
TMEM9B	ENSG00000175348	3064	0.247095	0.000173	0.006458
RPL23	ENSG00000125691	5979	0.247166	0.002734	0.031391
RP11-977G19.10	ENSG00000144785	1053	0.247517	3.2E-05	0.002759
ATP6V1F	ENSG00000128524	789	0.24753	0.00045	0.010918
YIPF6	ENSG00000181704	8850	0.247713	0.000409	0.010337
ATG4C	ENSG00000125703	3110	0.247972	0.001659	0.023132
GNB2L1	ENSG00000204628	5990	0.247982	0.000816	0.015231
ARL6IP5	ENSG00000144746	2522	0.24826	0.001115	0.018407
HSPD1	ENSG00000144381	4245	0.248303	0.00069	0.013872
GTF3C6	ENSG00000155115	1067	0.24839	0.002729	0.031391
RP11-863K10.7	ENSG00000183154	2086	0.248623	0.001094	0.018197
BPGM	ENSG00000172331	2539	0.24898	0.000473	0.011246
PHF5A	ENSG00000100410	1235	0.249234	0.000547	0.012199
NEIL2	ENSG00000154328	3367	0.249252	0.005485	0.047931
RP3-337H4.9	ENSG00000271754	545	0.249378	0.00236	0.028645
GFM2	ENSG00000164347	4197	0.249545	1.12E-06	0.000392
RP1-101K10.6	ENSG00000227627	663	0.249547	0.000744	0.01445
RPL29	ENSG00000162244	1912	0.249632	0.00545	0.047775
NDUFB5	ENSG00000136521	5871	0.249672	0.001249	0.019531
ARPC5	ENSG00000162704	10624	0.249732	0.00067	0.013679
DYNLL1	ENSG00000088986	2957	0.249764	0.00244	0.029183
CKLF-CMTM1	ENSG00000254788	1437	0.249826	0.002377	0.028733
TRMT12	ENSG00000183665	2331	0.249909	0.00013	0.005521
LINC00998	ENSG00000214194	1896	0.249923	0.002029	0.026273
CKLF	ENSG00000217555	1161	0.25006	0.001973	0.025667
HAT1	ENSG00000128708	6790	0.250205	0.000915	0.016338
NDUFB8	ENSG00000166136	1999	0.25026	0.001817	0.024449
RP11-886H22.1	ENSG00000266997	936	0.250391	0.002447	0.029226
CYB561D2	ENSG00000114395	2086	0.250556	0.004319	0.041479
GRPEL1	ENSG00000109519	4177	0.250697	0.00081	0.015195
RP11-76I23.7	ENSG00000231880	553	0.250911	0.000427	0.010567
MXD3	ENSG00000213347	5154	0.251023	0.004666	0.043381
BANF1	ENSG00000175334	1645	0.251195	0.000417	0.010418
RPN2	ENSG00000118705	2819	0.251306	0.00102	0.017327

PPIAP22	ENSG00000198618	498	0.251363	0.000886	0.015949
EEF1B2P3	ENSG00000232472	678	0.251408	0.002158	0.027203
NDUFB4	ENSG00000065518	1889	0.251758	0.001832	0.024574
TRAPPC3	ENSG00000054116	3527	0.251882	9.06E-06	0.001298
U47924.29	ENSG00000271969	434	0.252052	0.001432	0.021196
XPOT	ENSG00000184575	7364	0.252193	0.003475	0.036468
SPCS2	ENSG00000118363	2982	0.252351	2.23E-05	0.002133
RARS	ENSG00000113643	3421	0.252413	0.000735	0.014355
PRDX3	ENSG00000165672	3417	0.252434	0.000257	0.008059
ARL1	ENSG00000120805	4979	0.252559	0.003243	0.034763
VPS25	ENSG00000131475	2404	0.252765	7.82E-05	0.00438
ST13	ENSG00000100380	4147	0.252971	0.000393	0.010124
RNF113A	ENSG00000125352	1353	0.25314	0.001913	0.025178
AK6	ENSG00000273841	2577	0.253678	0.0007	0.013956
NEDD8-MDP1	ENSG00000255526	1051	0.253697	8.24E-05	0.004483
EMC7	ENSG00000134153	1538	0.253923	0.000956	0.016763
MRPL39	ENSG00000154719	1199	0.254063	0.000553	0.012276
HSPE1-MOB4	ENSG00000270757	890	0.254232	0.001021	0.01734
TCP1	ENSG00000120438	4929	0.254243	0.000209	0.007222
GTF2H5	ENSG00000272047	7481	0.25442	0.003674	0.037619
MRPL27	ENSG00000108826	3027	0.254495	0.000191	0.006802
RPL7A	ENSG00000148303	2242	0.254575	0.000489	0.011512
RP5-827C21.4	ENSG00000231663	575	0.254788	0.004641	0.043218
AKR1A1	ENSG00000117448	3121	0.255086	0.002967	0.032931
EMC4	ENSG00000128463	4459	0.255183	9.34E-05	0.004774
SELT	ENSG00000198843	4135	0.255213	0.000205	0.007163
RPL27	ENSG00000131469	2026	0.255869	0.001381	0.020627
RANBP1	ENSG00000099901	4622	0.256047	0.004573	0.042815
RPL6P27	ENSG00000235552	872	0.256109	0.001442	0.021273
JPX_1	ENSG00000274430	135	0.256121	0.001301	0.019986
ATP5G2	ENSG00000135390	3991	0.25618	0.001427	0.021162
TMEM14C	ENSG00000111843	1321	0.256299	0.000145	0.005806
RPA2	ENSG00000117748	2019	0.25633	0.00014	0.005743
MRPL20	ENSG00000242485	2788	0.256391	0.000213	0.007271
RP11-138I1.4	ENSG00000265401	461	0.256396	0.00051	0.011785
SWI5	ENSG00000175854	1649	0.256638	0.004071	0.040034
ATG101	ENSG00000123395	3199	0.256909	0.005763	0.049315
EIF3I	ENSG00000084623	1725	0.256992	0.000727	0.014282
BNIP1	ENSG00000113734	2440	0.257065	0.000837	0.015391
IDH1	ENSG00000138413	4721	0.25708	0.001454	0.021349
EIF3L	ENSG00000100129	4296	0.25715	0.000407	0.010313

GHITM	ENSG00000165678	2449	0.257193	0.002589	0.030267
RP11-421L21.2	ENSG00000235795	671	0.257262	0.001609	0.022733
AC004549.6	ENSG00000229893	859	0.257405	0.000757	0.014574
EDF1	ENSG00000107223	1200	0.257531	0.001116	0.018408
FH	ENSG00000091483	2142	0.257711	0.00035	0.009516
C6orf48	ENSG00000204387	1801	0.257949	0.001831	0.024574
PDRG1	ENSG00000088356	1998	0.258464	0.00118	0.018924
POLR2F	ENSG00000100142	7042	0.258549	9.49E-05	0.004815
NDUFA7	ENSG00000267855	1413	0.25864	0.005005	0.045355
MRPL3	ENSG00000114686	2715	0.258642	0.000385	0.010015
STT3A	ENSG00000134910	7345	0.2587	0.000299	0.008696
MRPL47	ENSG00000136522	1370	0.258767	0.002532	0.029855
GABARAP	ENSG00000170296	2266	0.258788	1.19E-05	0.001491
TOMM22	ENSG00000100216	2191	0.258962	0.000425	0.01054
RP11-371E8.4	ENSG00000259066	920	0.258972	3.9E-06	0.000804
COX16	ENSG00000133983	1863	0.259115	0.000549	0.012232
C17orf58	ENSG00000186665	1752	0.259421	0.004559	0.042762
TRAPPC2L	ENSG00000167515	4463	0.2596	0.000973	0.016885
NHP2	ENSG00000145912	1510	0.259691	0.001372	0.020548
NDUFS4	ENSG00000164258	1176	0.259741	0.000412	0.010356
DOLK	ENSG00000175283	2090	0.259858	0.000255	0.008022
CCT8	ENSG00000156261	3341	0.259889	5.67E-05	0.0037
PSMC4	ENSG00000013275	2387	0.259898	0.005322	0.047204
POLR2L	ENSG00000177700	1142	0.259913	0.005543	0.048191
C2orf47	ENSG00000162972	1624	0.260513	0.000584	0.012728
RP11-603J24.14	ENSG00000257809	540	0.260617	0.002237	0.027677
YRDC	ENSG00000196449	1826	0.260632	0.002285	0.027993
PRMT1	ENSG00000126457	4796	0.26068	0.000982	0.016953
SARS	ENSG00000031698	3836	0.260762	0.002471	0.029405
DPH3	ENSG00000154813	3562	0.260968	0.001807	0.024341
MRPL50	ENSG00000136897	3332	0.261532	0.000805	0.015153
SPCS3	ENSG00000129128	6831	0.261875	0.000396	0.010156
RP11-97O12.5	ENSG00000279945	517	0.261918	0.003113	0.034012
RPL35	ENSG00000136942	1535	0.26192	0.001549	0.022148
TP53TG1	ENSG00000182165	1023	0.262218	0.002692	0.031139
MRPL36	ENSG00000171421	1394	0.262317	0.002733	0.031391
CCT7	ENSG00000135624	3585	0.262324	4.83E-05	0.003409
FAM103A2P	ENSG00000235272	354	0.26236	0.004802	0.04412
GPN3	ENSG00000111231	2595	0.262456	0.001103	0.018281
TRIAP1	ENSG00000170855	1442	0.262535	1.85E-05	0.001937
TAF9	ENSG00000085231	2162	0.262797	0.00472	0.043683

FLAD1	ENSG00000160688	4019	0.26287	0.000608	0.01298
GOT1	ENSG00000120053	2424	0.263131	0.001175	0.018886
PACSIN2	ENSG00000100266	4548	0.263165	0.004547	0.042706
PSMA2	ENSG00000256646	3068	0.263212	0.000196	0.006911
CHCHD4	ENSG00000163528	1772	0.263463	0.000256	0.008025
PGD	ENSG00000142657	2966	0.2635	0.003227	0.034712
RP11-403P17.6	ENSG00000277978	382	0.263708	0.00192	0.02522
RPA3	ENSG00000106399	3232	0.263793	0.000336	0.009306
ARL6IP1	ENSG00000170540	3666	0.263824	0.001721	0.023695
FBXO10	ENSG00000147912	5997	0.26385	0.001687	0.023357
PRMT5	ENSG00000100462	3467	0.263906	0.000792	0.015052
PHB	ENSG00000167085	3838	0.264032	0.001085	0.018105
PSMC2	ENSG00000161057	3538	0.264703	0.000251	0.007968
IGBP1-AS2	ENSG00000220925	513	0.265413	0.002031	0.026279
C1orf43	ENSG00000143612	2582	0.265566	4.46E-05	0.003216
PLA2G4A	ENSG00000116711	2875	0.265954	0.000538	0.012135
LSM5	ENSG00000106355	4701	0.26622	0.000324	0.009107
CENPN	ENSG00000166451	3568	0.266251	0.000925	0.016461
PRMT5-AS1	ENSG00000237054	2685	0.266278	0.001251	0.019535
RP11-1012A1.4	ENSG00000258466	804	0.266285	0.001098	0.018239
UCHL3	ENSG00000118939	1905	0.26655	0.000268	0.00824
RP11-644F5.11	ENSG00000258056	1443	0.266869	0.004332	0.041534
PNO1	ENSG00000115946	2674	0.266944	0.001107	0.018325
SNRPC	ENSG00000124562	1277	0.267116	0.000181	0.006619
C10orf32-ASMT	ENSG00000270316	2670	0.267172	0.000673	0.013708
MRPS33	ENSG00000090263	1818	0.26738	0.000833	0.015345
FAM162A	ENSG00000114023	3793	0.267977	0.000279	0.008399
CHAC2	ENSG00000143942	1321	0.268107	0.003639	0.037431
MROH8	ENSG00000101353	3878	0.268157	0.001622	0.022793
KIAA0391	ENSG00000258790	3857	0.268252	0.000109	0.005083
AC113189.5	ENSG00000233223	1058	0.268338	0.000191	0.006802
NDUFC1	ENSG00000109390	1941	0.268607	0.000276	0.008351
RPL36A-HNRNPH2	ENSG00000257529	695	0.268686	0.000598	0.012867
MRPL11	ENSG00000174547	2539	0.26872	0.000583	0.012726
NUP37	ENSG00000075188	3492	0.268857	0.00029	0.008612
TMX2	ENSG00000213593	2246	0.26902	0.000231	0.007647
ANAPC13	ENSG00000129055	2359	0.269216	0.000682	0.01379
TIGD4	ENSG00000169989	2472	0.269323	0.002398	0.028865
VRK1	ENSG00000100749	2744	0.269327	0.002819	0.03202
SENP3-EIF4A1	ENSG00000277957	3519	0.269404	0.001293	0.019886

ATP5C1P1	ENSG00000224004	895	0.269468	0.004158	0.040586
CHCHD2	ENSG00000106153	1132	0.269922	0.001235	0.019421
TMEM116	ENSG00000198270	7989	0.269925	0.005436	0.047734
RPL36A	ENSG00000241343	3964	0.270113	0.00295	0.032817
RP11-518L10.5	ENSG00000240963	642	0.270391	0.002656	0.030797
SLC25A11	ENSG00000108528	2669	0.27055	0.000975	0.016906
TXNDC9	ENSG00000115514	2816	0.270635	0.000544	0.012171
CCT2	ENSG00000166226	4322	0.27077	0.000298	0.008696
APOO	ENSG00000184831	1146	0.270799	0.001341	0.020311
PDCL3	ENSG00000115539	1831	0.270933	0.000261	0.008127
NDUFA2	ENSG00000131495	1488	0.271502	0.002127	0.026998
AC010468.1	ENSG00000214784	817	0.271807	0.002738	0.031391
COA3	ENSG00000183978	1922	0.271809	2.98E-05	0.002623
XPNPEP1	ENSG00000108039	6691	0.271903	0.002956	0.032873
STS	ENSG00000101846	6521	0.272103	0.000987	0.016984
snoU2-30	ENSG00000273885	70	0.272308	0.000292	0.008637
FAM195B	ENSG00000225663	3726	0.272981	0.005477	0.047906
NME1-NME2	ENSG00000243678	2638	0.273203	0.001367	0.020498
TUBA1A	ENSG00000167552	3421	0.273279	0.001962	0.025535
CACYBPP2	ENSG00000177855	678	0.274237	0.001243	0.019468
ASCC1	ENSG00000138303	5586	0.274672	9.2E-06	0.001298
HIST1H1C	ENSG00000187837	642	0.274674	0.000681	0.013776
ATG10	ENSG00000152348	5449	0.274805	0.002432	0.029139
CCT3	ENSG00000163468	4018	0.274825	2.2E-05	0.002133
RP11-234B24.6	ENSG00000255639	1186	0.27494	0.00069	0.013872
NXT1	ENSG00000132661	1123	0.275144	0.002189	0.027408
RPL18AP3	ENSG00000213442	528	0.275271	0.00494	0.044995
RP11-1060J15.4	ENSG00000256377	1519	0.275787	0.001534	0.022056
PSMD8	ENSG00000099341	3105	0.275977	0.000597	0.012861
SNAPIN	ENSG00000143553	1544	0.27603	0.000114	0.005202
ARMCX3	ENSG00000102401	4239	0.276097	2.72E-05	0.002485
COX7B	ENSG00000131174	3024	0.276597	0.001863	0.024805
RPS28	ENSG00000233927	2119	0.276675	0.000405	0.010293
RP4-758J18.2	ENSG00000224870	2525	0.276679	0.000148	0.005859
POLR2G	ENSG00000168002	1923	0.277029	1.02E-05	0.001374
HCRTR1	ENSG00000121764	2558	0.277043	0.002697	0.031166
ZDHHC4	ENSG00000136247	3557	0.277077	2.02E-05	0.002044
YARS	ENSG00000134684	8212	0.277219	0.003642	0.037431
CHCHD5	ENSG00000125611	3759	0.277658	0.003043	0.033549
TIMM17B	ENSG00000126768	2562	0.277751	0.001925	0.02527
RPS10-NUDT3	ENSG00000270800	876	0.277959	0.000593	0.012846

AL162151.3	ENSG00000234648	158	0.278026	0.001749	0.023914
HSBP1	ENSG00000230989	9525	0.278253	1.22E-05	0.001509
RPN1	ENSG00000163902	3992	0.278336	0.000265	0.008194
EEF1A1	ENSG00000156508	5948	0.278409	0.000208	0.007205
SHFM1	ENSG00000127922	11490	0.278461	3.99E-06	0.000808
CTB-96E2.3	ENSG00000258924	1050	0.278894	0.000505	0.011758
SNRPD1	ENSG00000167088	1841	0.278916	0.000497	0.011616
SMCO4	ENSG00000166002	1223	0.279222	0.002057	0.026499
GYG1	ENSG00000163754	3475	0.280098	7.74E-06	0.001169
RP11-307L3.2	ENSG00000233846	487	0.280109	0.001127	0.018488
ALG8	ENSG00000159063	3448	0.280268	0.00018	0.006608
METTL7B	ENSG00000170439	1506	0.280319	0.004414	0.042036
RPL21	ENSG00000122026	2249	0.280637	0.002283	0.027981
ST6GALNAC1	ENSG00000070526	3448	0.280738	0.002246	0.027751
MAD2L2	ENSG00000116670	3324	0.280746	0.002556	0.030004
LAMTOR2	ENSG00000116586	1438	0.280852	0.000467	0.011143
CTC-297N7.10	ENSG00000263388	171	0.280971	0.003066	0.033692
HSPD1P1	ENSG00000213430	1726	0.280989	0.000602	0.012896
MORF4L2	ENSG00000123562	2648	0.280996	0.000657	0.01357
C4orf3	ENSG00000279402	201	0.281372	0.000454	0.010981
H3F3B	ENSG00000132475	6134	0.281592	0.000387	0.010037
AC016708.2	ENSG00000230076	318	0.281775	0.002378	0.028733
SNRPB	ENSG00000125835	1278	0.281895	0.003004	0.033226
RP11-1100L3.7	ENSG00000257663	647	0.282146	0.002188	0.027401
FAU	ENSG00000149806	1846	0.282258	0.000378	0.009926
RPL4	ENSG00000174444	5637	0.282541	0.000421	0.010495
N6AMT2	ENSG00000150456	1339	0.282649	0.001487	0.021654
RPL26	ENSG00000161970	2472	0.282849	0.004836	0.044309
YBX1P10	ENSG00000213866	975	0.28287	0.003998	0.039592
SSR3	ENSG00000114850	4627	0.283052	5.95E-05	0.0038
SERF2	ENSG00000140264	7347	0.283333	0.000393	0.010124
WDR89	ENSG00000140006	3209	0.283403	0.000121	0.005346
XXcos-LUCA11.5	ENSG00000272104	408	0.283726	0.00116	0.018795
UBB	ENSG00000170315	1621	0.283734	0.000134	0.005606
MMACHC	ENSG00000132763	5296	0.28383	0.000846	0.0155
MIR194-1	ENSG00000207624	85	0.284016	0.001886	0.025006
ATP5J2	ENSG00000241468	2275	0.284026	0.001747	0.023912
TTI2	ENSG00000129696	4222	0.28403	2.12E-06	0.000545
REP15	ENSG00000174236	1078	0.284185	0.000983	0.016953
MMAB	ENSG00000139428	6290	0.284195	0.002216	0.027645
GPR180	ENSG00000152749	8822	0.284252	0.005501	0.047994

PEF1	ENSG00000162517	2227	0.284332	0.000374	0.00987
AC079742.4	ENSG00000232581	357	0.284343	0.000341	0.009378
MRFAP1	ENSG00000179010	2655	0.284562	4.31E-06	0.000847
TXNDC17	ENSG00000129235	3784	0.284991	0.001133	0.018518
PNPLA3	ENSG00000100344	3232	0.284997	0.000387	0.010037
SPINT2	ENSG00000167642	5199	0.285069	0.001826	0.02453
RBKS	ENSG00000171174	3625	0.285308	0.000987	0.016984
RPS11	ENSG00000142534	1390	0.285366	0.000365	0.009719
NDUFAF1	ENSG00000137806	1790	0.285666	0.000133	0.005604
SLC2A3P1	ENSG00000253861	1451	0.285701	0.00561	0.04851
EIF4A3	ENSG00000141543	4967	0.285828	0.000118	0.0053
KRTCAP2	ENSG00000163463	3826	0.285845	1.21E-05	0.001504
LGALS4	ENSG00000171747	2645	0.285853	0.004762	0.043941
MYL6	ENSG00000092841	4609	0.28596	0.000254	0.008018
CNPY2	ENSG00000257727	2956	0.286373	3.68E-06	0.000781
COA6	ENSG00000168275	1257	0.286396	0.000456	0.011003
MYEOV2	ENSG00000172428	2039	0.286407	6.21E-05	0.003815
RPL24	ENSG00000114391	2682	0.286616	0.000354	0.009558
NDUFAF4	ENSG00000123545	2540	0.286743	0.00452	0.042544
RPL37	ENSG00000145592	8153	0.286761	0.001084	0.018105
RPL15P3	ENSG00000212802	615	0.286881	0.000329	0.009198
RP11-380N8.7	ENSG00000277368	464	0.287017	0.003097	0.033909
CIAPIN1	ENSG00000005194	3509	0.287021	0.000308	0.008834
NEDD8	ENSG00000129559	3879	0.28707	1.82E-05	0.001926
MRPL51	ENSG00000111639	1551	0.287213	2.96E-05	0.002614
ALG5	ENSG00000120697	2194	0.287301	4.65E-05	0.00333
HSP90AA2P	ENSG00000224411	2196	0.287301	0.005022	0.045451
HIST1H3E	ENSG00000274750	411	0.287367	0.000812	0.015212
RBX1	ENSG00000100387	3092	0.288147	2.84E-05	0.002553
LAMTOR5	ENSG00000134248	2388	0.288271	6.09E-05	0.003803
MRPL17	ENSG00000158042	2276	0.288317	0.000245	0.007896
MCTS1	ENSG00000232119	2837	0.288332	0.000133	0.005604
RPS10	ENSG00000124614	1642	0.288467	0.000507	0.011769
ATP5J	ENSG00000154723	2043	0.288619	0.000355	0.00956
NDUFS5	ENSG00000168653	548	0.288904	0.001004	0.017189
MPDU1	ENSG00000129255	3285	0.288935	7.39E-05	0.004224
PRPSAP2	ENSG00000141127	4734	0.289019	1.1E-05	0.001418
HSD17B10	ENSG00000072506	1115	0.289065	0.001782	0.024153
RP11-500M8.7	ENSG00000272921	818	0.2892	0.000428	0.010591
MRPS7	ENSG00000125445	3324	0.289249	0.000738	0.014389
ACAA2	ENSG00000167315	5072	0.28929	0.000896	0.016068

AIMP2	ENSG00000106305	1838	0.289295	0.00018	0.006608
EEF1B2	ENSG00000114942	1915	0.289345	0.000262	0.00816
ATP6V1E1	ENSG00000131100	3212	0.289404	6.12E-05	0.003803
CTB-102L5.4	ENSG00000267748	546	0.28953	0.002114	0.02687
RP11-761B3.1	ENSG00000273269	983	0.289663	0.000648	0.013481
RTN3P1	ENSG00000251333	711	0.289688	0.001009	0.017244
PET100	ENSG00000229833	2220	0.289842	0.000539	0.012138
RAN	ENSG00000132341	4825	0.290122	0.000279	0.008399
CTD-2026K11.2	ENSG00000260206	572	0.290293	0.002752	0.03143
ZFAS1	ENSG00000177410	2243	0.290377	0.00099	0.017009
MAGOH	ENSG00000162385	1463	0.290659	5.57E-06	0.000986
RPS27	ENSG00000177954	946	0.29086	0.00298	0.033011
PSMC3	ENSG00000165916	2276	0.291189	8.03E-05	0.004442
RP4-605O3.4	ENSG00000272368	1117	0.291348	0.00042	0.010464
SNRPF	ENSG00000139343	3043	0.291351	0.000831	0.015331
SELK	ENSG00000113811	2165	0.291432	0.002384	0.028747
STOML2	ENSG00000165283	1806	0.291933	0.000117	0.005271
DPM3	ENSG00000179085	622	0.291939	0.003938	0.03918
GPX1	ENSG00000233276	1425	0.292174	0.001413	0.021012
PSMB4	ENSG00000159377	2158	0.292498	0.00013	0.005521
PRDX5	ENSG00000126432	893	0.293243	0.002217	0.027645
NME2	ENSG00000011052	1241	0.293267	0.000918	0.016367
NDC1	ENSG00000058804	5135	0.293526	0.001784	0.024167
NCEH1	ENSG00000144959	4808	0.293833	0.000236	0.007735
ARL3	ENSG00000138175	3844	0.294103	0.00021	0.007234
PSMD10	ENSG00000101843	2459	0.294292	9.98E-05	0.004942
COX5B	ENSG00000135940	1441	0.294456	0.000665	0.013659
LSM1	ENSG00000175324	1690	0.294875	1.32E-05	0.00159
RN7SK	ENSG00000202198	331	0.295182	0.002532	0.029855
COX7C	ENSG00000127184	1993	0.295684	9.33E-05	0.004774
NDUFA12	ENSG00000184752	1815	0.295705	0.001569	0.022314
ERH	ENSG00000100632	1446	0.296056	0.000131	0.005551
LEPROTL1	ENSG00000104660	5421	0.296058	0.000112	0.005147
COX8A	ENSG00000176340	507	0.296135	0.004594	0.042883
NUDT1	ENSG00000106268	2093	0.296732	0.002816	0.032012
RPL10A	ENSG00000198755	1501	0.297034	0.000679	0.013759
FAM3D	ENSG00000198643	1640	0.297256	0.002702	0.031214
NDUFA9	ENSG00000139180	10391	0.297425	0.000188	0.006754
EIF3K	ENSG00000178982	2798	0.297866	0.00021	0.007234
RPL13AP5	ENSG00000236552	612	0.297952	0.000952	0.016723
TMEM263	ENSG00000151135	4768	0.298065	0.000884	0.015941

YAE1D1	ENSG00000241127	2569	0.298104	0.004594	0.042883
SNORD58B	ENSG00000271982	66	0.298123	0.000545	0.012177
UBA52	ENSG00000221983	4306	0.298246	7E-05	0.004117
ATP6V0E1	ENSG00000113732	1623	0.29881	2.89E-06	0.000669
NDUFC2	ENSG00000151366	2652	0.298841	9.67E-05	0.004873
PRDX1	ENSG00000117450	1522	0.298867	0.00052	0.011906
RP11-123K3.9	ENSG00000276727	701	0.298921	0.003462	0.036346
PSMB1	ENSG00000008018	2256	0.299062	2.83E-05	0.002553
COX14	ENSG00000178449	1073	0.299484	8.63E-05	0.004578
ATP5G1	ENSG00000159199	2615	0.299798	0.001011	0.017262
AC107081.5	ENSG00000236498	802	0.300078	2.28E-06	0.000566
MMADHC	ENSG00000168288	2191	0.300335	7.24E-05	0.004182
NDUFS6	ENSG00000145494	1451	0.300598	0.000796	0.015076
GJB2	ENSG00000165474	2427	0.300699	0.000813	0.015212
TMEM126B	ENSG00000171204	2416	0.300814	3.6E-05	0.002905
C19orf12	ENSG00000131943	5378	0.300855	0.000909	0.016272
SLC25A5	ENSG00000005022	1500	0.300951	0.000274	0.008346
ENO1	ENSG00000074800	3958	0.301366	0.000274	0.008346
EFNA4	ENSG00000243364	1263	0.301438	0.002768	0.031581
NDUFA6	ENSG00000184983	1416	0.301482	0.00035	0.009521
MIR632	ENSG00000207928	94	0.301874	0.000116	0.005253
CROT	ENSG00000005469	4979	0.301908	0.002595	0.030303
SLC50A1	ENSG00000169241	2340	0.301921	0.004767	0.043956
BLOC1S1	ENSG00000135441	1656	0.302262	0.000163	0.006205
MOGAT2	ENSG00000166391	4139	0.302348	0.004716	0.043661
COMMD8	ENSG00000169019	1766	0.302774	0.000276	0.008351
COPS4	ENSG00000138663	3566	0.303488	3.84E-05	0.002967
PSMB6	ENSG00000142507	1448	0.303695	0.001066	0.01789
COX6A1	ENSG00000111775	1344	0.303882	0.00103	0.01745
RPL26L1	ENSG00000037241	1164	0.303902	0.001111	0.01838
SDHD	ENSG00000204370	2509	0.304232	0.000201	0.007056
TMBIM4	ENSG00000155957	4137	0.304285	1.04E-05	0.001378
DNAJB9	ENSG00000128590	2837	0.30482	0.001499	0.021728
DYNLT1	ENSG00000146425	2949	0.304943	0.00026	0.008111
CCL15-CCL14	ENSG00000275688	1047	0.304944	0.000353	0.009555
MSRB1	ENSG00000198736	2157	0.305597	0.000311	0.008861
RPS23	ENSG00000186468	4204	0.305694	0.001338	0.020293
GLA	ENSG00000102393	2065	0.305826	0.000191	0.006802
ATP1B3	ENSG00000069849	2584	0.306122	0.000942	0.016589
HSPA13	ENSG00000155304	4194	0.306187	0.002969	0.032931
PCNA	ENSG00000132646	1471	0.306418	4.05E-05	0.003037

ATP6V1C2	ENSG00000143882	3324	0.306466	0.00058	0.012691
TSC22D3	ENSG00000157514	4766	0.306779	0.003673	0.037619
DBI	ENSG00000155368	2444	0.306857	0.000832	0.015331
STEAP1	ENSG00000164647	1625	0.306963	0.002733	0.031391
CTA-292E10.9	ENSG00000280111	24928	0.307148	0.001444	0.021278
ZNFX1-AS1_2	ENSG00000274760	93	0.307172	0.00195	0.025435
PSMA6	ENSG00000100902	6506	0.307208	6.3E-05	0.003815
ELOVL6	ENSG00000170522	7297	0.307335	0.00449	0.042489
PSMA4	ENSG00000041357	6996	0.307404	7.03E-05	0.004117
TMEM38B	ENSG00000095209	4511	0.307502	0.003478	0.036493
RPL30	ENSG00000156482	4027	0.307699	0.000637	0.013347
SNORA5A	ENSG00000206838	134	0.307707	0.005606	0.048489
CCL15	ENSG00000275718	1560	0.307887	0.00037	0.009813
NHP2L1	ENSG00000100138	2780	0.307953	4.11E-06	0.00082
VBP1	ENSG00000155959	2018	0.308249	7.34E-05	0.004222
TMEM101	ENSG00000091947	3029	0.308517	0.000191	0.006802
GAS5	ENSG00000234741	3631	0.308887	0.00031	0.008835
AMD1	ENSG00000123505	7222	0.30892	0.000455	0.010999
ATL2	ENSG00000119787	4885	0.308974	0.002875	0.032351
PYURF	ENSG00000145337	1361	0.309223	2.64E-07	0.000139
ECE2	ENSG00000145194	6552	0.309288	0.005194	0.046564
SNORA71A	ENSG00000225091	134	0.309363	0.005507	0.047998
KLHL13	ENSG00000003096	6983	0.309752	0.003603	0.037183
PDIA6	ENSG00000143870	4101	0.309762	0.000427	0.01057
TRMT10C	ENSG00000174173	1888	0.310061	8.3E-05	0.004484
DLEU2_3	ENSG00000274878	73	0.310227	0.004916	0.044814
CTB-13L3.1	ENSG00000273299	371	0.310347	0.002318	0.028271
TOMM7	ENSG00000196683	1751	0.310709	9.74E-05	0.004884
KIF3B	ENSG00000101350	6103	0.310827	0.001247	0.019503
RNF181	ENSG00000168894	1220	0.311646	4.05E-05	0.003037
RP11-338I21.1	ENSG00000271933	2163	0.311792	0.001574	0.02238
C16orf91	ENSG00000174109	1057	0.311982	0.001758	0.023974
C7orf55	ENSG00000164898	1524	0.311984	0.000988	0.016987
TATDN1	ENSG00000147687	4778	0.312085	5.92E-05	0.003788
CTSH	ENSG00000103811	5892	0.31227	9.1E-05	0.004731
CDC26	ENSG00000176386	1175	0.312752	1.39E-05	0.001625
RPL37A	ENSG00000197756	6841	0.313288	0.000153	0.005998
GPR128	ENSG00000144820	4091	0.313507	0.000354	0.009558
FEN1	ENSG00000168496	2478	0.313555	0.004188	0.040762
TMEM14B	ENSG00000137210	4815	0.313591	1.25E-07	9.8E-05
RP11-3P17.4	ENSG00000240567	2845	0.314004	0.000332	0.009245

NDUFB1	ENSG00000183648	2263	0.314509	0.00032	0.009035
KRBA2	ENSG00000263809	3087	0.314519	0.001079	0.018056
CREB3	ENSG00000107175	1921	0.314912	0.004891	0.044625
HINT1	ENSG00000169567	1936	0.315383	4.16E-06	0.000823
SFXN4	ENSG00000183605	1868	0.315905	9.96E-05	0.004942
TMEM53	ENSG00000126106	3635	0.315914	0.000402	0.01025
RP11-1017G21.5	ENSG00000271780	1079	0.315973	0.00077	0.014749
RPS18	ENSG00000231500	1898	0.316044	0.000832	0.015331
PPIB	ENSG00000166794	1911	0.316129	0.000379	0.00993
TMEM261	ENSG00000137038	3000	0.316258	6.06E-05	0.003803
RP11-90P5.2	ENSG00000253356	562	0.316328	6.94E-05	0.004092
RNF7	ENSG00000114125	3147	0.316522	4.39E-05	0.003193
C19orf10	ENSG00000074842	1678	0.316812	0.003016	0.033331
TMEM147	ENSG00000105677	1932	0.317489	0.000251	0.007968
C14orf2	ENSG00000156411	3415	0.317491	0.000182	0.006639
TARS	ENSG00000113407	5718	0.317559	0.002533	0.029855
SNX22	ENSG00000157734	5767	0.317598	0.000483	0.011392
WDR83OS	ENSG00000105583	2656	0.317742	0.000221	0.007424
COMMD6	ENSG00000188243	2897	0.317796	6.01E-05	0.003803
SNORD6	ENSG00000202314	73	0.31787	0.001377	0.020611
KCNK3	ENSG00000171303	6223	0.317982	0.000508	0.011777
TAF1D	ENSG00000166012	8420	0.318142	0.000105	0.005013
PSMA7	ENSG00000101182	1750	0.318469	0.000241	0.007839
TMEM14A	ENSG00000096092	992	0.31865	0.00038	0.009936
AC079922.2	ENSG00000231747	359	0.318665	0.005866	0.049897
MARS	ENSG00000166986	7028	0.31896	0.000608	0.01298
COQ3	ENSG00000132423	1458	0.318994	0.000142	0.005755
H2AFJ	ENSG00000246705	3356	0.319258	0.000528	0.012001
RP11-864N7.2	ENSG00000227615	399	0.319328	0.000159	0.006121
SRSF7	ENSG00000115875	4045	0.319485	1.14E-08	1.64E-05
RPL13A	ENSG00000142541	2369	0.319677	0.000274	0.008344
RP13-753N3.3	ENSG00000275185	542	0.319983	4.04E-05	0.003037
ASNA1	ENSG00000198356	1652	0.320007	0.000265	0.008194
RPS23P8	ENSG00000230629	330	0.320343	0.000533	0.01207
RPS19	ENSG00000105372	3805	0.320519	0.000201	0.007056
NDUFAB1	ENSG00000004779	1600	0.320801	0.000155	0.006076
RP11-201K10.3	ENSG00000273088	753	0.320944	6.21E-06	0.001068
HAX1	ENSG00000143575	2155	0.321269	9.26E-06	0.001298
CCDC175	ENSG00000151838	3245	0.32175	0.000698	0.013945
SNRPE	ENSG00000182004	1474	0.321906	0.000288	0.0086
HIST1H4I	ENSG00000276180	1294	0.322027	0.003537	0.036851

TMEM135	ENSG00000166575	5030	0.322035	0.000233	0.007689
CLRN3	ENSG00000180745	1152	0.322603	0.000213	0.007271
PANK1	ENSG00000152782	7638	0.322623	0.001914	0.025178
HNRNPCP2	ENSG00000204253	881	0.323266	1.81E-05	0.001926
NSMCE1	ENSG00000169189	3770	0.323398	1.32E-05	0.00159
BTD	ENSG00000169814	3704	0.323425	0.000772	0.014765
ATP5E	ENSG00000124172	5126	0.324041	1.18E-05	0.001484
MIR4680	ENSG00000265827	66	0.324046	0.000318	0.009012
TMEM106C	ENSG00000134291	3475	0.324196	9.78E-05	0.004896
CTSB	ENSG00000164733	7428	0.32457	0.000122	0.005346
RP11-573D15.9	ENSG00000263826	2400	0.324626	4.65E-05	0.00333
ACSL5	ENSG00000197142	4236	0.325781	0.003399	0.035932
ATP6V1D	ENSG00000100554	4251	0.325896	0.000109	0.00508
RPSAP15	ENSG00000237506	861	0.325904	0.002696	0.031166
C14orf142	ENSG00000170270	1154	0.327225	2.29E-05	0.00217
AGR3	ENSG00000173467	1160	0.327306	0.001351	0.020379
ANAPC11	ENSG00000141552	4027	0.32742	0.000698	0.013945
PSMA5	ENSG00000143106	5724	0.327534	1.32E-05	0.00159
ST13P6	ENSG00000225259	1102	0.327554	0.000107	0.005039
KCTD14	ENSG00000151364	2702	0.32825	9.01E-05	0.004712
RPLP0	ENSG00000089157	4001	0.328633	0.000114	0.005197
SNHG1	ENSG00000255717	3756	0.329721	0.000286	0.008537
XX-FW83563B9.5	ENSG00000280195	1939	0.329833	0.00055	0.012245
RPS15A	ENSG00000134419	6679	0.330458	0.000212	0.00727
RPL29P11	ENSG00000224858	492	0.330917	0.004529	0.042595
SSR4	ENSG00000180879	4590	0.331032	0.00071	0.01408
WDR38	ENSG00000136918	1387	0.331162	0.000303	0.008738
NDUFA11	ENSG00000174886	4839	0.331769	0.000269	0.00824
SLC20A2	ENSG00000168575	6641	0.331791	0.001864	0.024805
RP11-87F15.2	ENSG00000248980	610	0.332111	7.23E-05	0.004182
RP11-637O19.3	ENSG00000272162	1589	0.332574	0.001328	0.020251
PNPLA4	ENSG00000006757	3441	0.33259	0.002318	0.028271
MSL3P1	ENSG00000224287	2278	0.333476	0.004122	0.040341
NDUFC2-KCTD14	ENSG00000259112	731	0.333788	7.73E-05	0.004357
AL132780.1	ENSG00000207765	109	0.334184	0.000873	0.015849
FAM103A1	ENSG00000169612	1568	0.334538	1.16E-05	0.001471
FAM50B	ENSG00000145945	1932	0.33478	0.001509	0.021834
PEX11A	ENSG00000166821	3171	0.334782	0.001335	0.020271
ATP5L	ENSG00000167283	3382	0.334852	2.71E-05	0.002483
BOLA3	ENSG00000163170	3156	0.335113	0.001851	0.024726
RP11-613H2.2	ENSG00000253899	1283	0.335476	0.000405	0.010294

SF3B5	ENSG00000169976	693	0.33572	7.05E-05	0.004117
SLIRP	ENSG00000119705	2631	0.335856	2.32E-05	0.002185
NDUFB9	ENSG00000147684	2629	0.336532	0.000145	0.005806
AC005538.3	ENSG00000279809	1864	0.336671	0.004036	0.039851
NUTF2	ENSG00000102898	3895	0.336769	2.73E-05	0.002487
UQCRH	ENSG00000173660	1408	0.336904	0.000131	0.005535
C17orf100	ENSG00000256806	1756	0.336917	0.003973	0.039428
RP11-1035H13.3	ENSG00000260342	764	0.336919	7.61E-06	0.001163
TMSB4X	ENSG00000205542	1705	0.337027	6.1E-05	0.003803
GALE	ENSG00000117308	3978	0.337226	0.003484	0.036524
WRB	ENSG00000182093	5558	0.337386	0.000102	0.004972
RPL10	ENSG00000147403	4825	0.337622	5.81E-05	0.003767
RPS12	ENSG00000112306	767	0.337861	0.000101	0.004971
SNORA72	ENSG00000245970	1989	0.337988	0.000459	0.011016
NDUFB7	ENSG00000099795	531	0.338142	0.001085	0.018105
MIR3656	ENSG00000266398	69	0.338195	0.005312	0.047152
PHB2	ENSG00000215021	2499	0.338204	0.000227	0.007585
PSENEN	ENSG00000205155	1845	0.338869	0.000105	0.005013
SLC9B1	ENSG00000164037	2977	0.338901	5.07E-05	0.00351
RPS4X	ENSG00000198034	2982	0.339108	0.00033	0.009198
USMG5	ENSG00000173915	898	0.339175	0.000647	0.013478
MRPL15	ENSG00000137547	1191	0.339192	6.55E-06	0.001113
TMEM216	ENSG00000187049	2364	0.339478	8.38E-07	0.000318
TMEM141	ENSG00000244187	1443	0.3396	0.003765	0.038179
COX6C	ENSG00000164919	3066	0.340342	7.83E-05	0.00438
DCTPP1	ENSG00000179958	1581	0.340642	3.71E-05	0.002942
CTD-2540B15.11	ENSG00000267580	659	0.34073	0.003181	0.034431
SPCS2P4	ENSG00000228589	681	0.341599	1.47E-06	0.000468
RPS3	ENSG00000149273	5894	0.341829	2.13E-05	0.00211
C12orf5	ENSG00000078237	3023	0.341954	0.000818	0.015231
NAA38	ENSG00000183011	1943	0.342377	4.91E-05	0.003429
AL021546.6	ENSG00000111780	552	0.342423	0.000843	0.015477
SYP	ENSG00000102003	5215	0.34265	0.001615	0.022774
NDUFA8	ENSG00000119421	844	0.343126	5.43E-06	0.000971
RPL7	ENSG00000147604	2907	0.343346	4.24E-05	0.003141
TMEM208	ENSG00000168701	1787	0.343666	0.000493	0.011582
MRPL13	ENSG00000172172	4209	0.34436	1.73E-06	0.000494
RP11-540D14.8	ENSG00000251246	1042	0.344479	0.00081	0.015195
GARS	ENSG00000106105	4152	0.344874	0.003828	0.038493
RP11-104J23.1	ENSG00000275944	1919	0.344878	0.00013	0.005521
RTN4IP1	ENSG00000130347	1974	0.345083	4.48E-06	0.00086

ATP5O	ENSG00000241837	3336	0.346036	6.23E-05	0.003815
B3GALNT1	ENSG00000169255	4183	0.346044	0.003684	0.037655
H1F0	ENSG00000189060	2344	0.34607	0.004079	0.040069
RP11-745O10.4	ENSG00000228144	984	0.346089	3.04E-06	0.000698
TCEB2	ENSG00000103363	1128	0.346182	0.000267	0.00824
UQCR11	ENSG00000267059	1002	0.346575	6.09E-05	0.003803
CFAP57	ENSG00000243710	5764	0.346806	0.002619	0.030524
TMED9	ENSG00000184840	2440	0.347619	0.001281	0.019765
SLC51B	ENSG00000186198	940	0.347851	0.000355	0.00956
RPL27A	ENSG00000166441	7193	0.347854	7.98E-06	0.001192
ATP6V0B	ENSG00000117410	3535	0.348055	0.000933	0.016541
FARSA-AS1	ENSG00000266975	241	0.348078	0.000945	0.01664
CRYBA2	ENSG00000163499	1454	0.348323	0.00539	0.047525
PPIL1	ENSG00000137168	1810	0.34884	0.000979	0.016923
MRPL34	ENSG00000130312	1686	0.349343	5.84E-05	0.003767
SNORA12	ENSG00000212464	147	0.349388	0.00535	0.047351
RBP1	ENSG00000114115	3905	0.349663	0.00366	0.037568
SNORD18C	ENSG00000199574	69	0.349686	0.003362	0.035639
SNRPD2	ENSG00000125743	1608	0.349845	3.93E-05	0.002999
CTD-2215E18.1	ENSG00000251513	1793	0.34993	0.001902	0.025119
CETN2	ENSG00000147400	1928	0.350041	0.000729	0.014283
CNGA3	ENSG00000144191	4378	0.350717	0.005245	0.046852
SMC2-AS1	ENSG00000270332	7619	0.351146	0.004506	0.042503
SLC25A20	ENSG00000178537	1982	0.351593	3.18E-05	0.002755
MPC1	ENSG00000060762	1546	0.351671	0.000726	0.014282
RP11-110J1.2	ENSG00000237842	1531	0.351812	3.32E-05	0.002792
FLRT3	ENSG00000125848	5190	0.351821	0.003029	0.033442
RP11-303E16.8	ENSG00000260643	773	0.352306	0.002736	0.031391
CKS1B	ENSG00000173207	2338	0.353232	8.75E-07	0.000323
AC024592.12	ENSG00000267740	2193	0.353426	0.000104	0.005013
XBP1	ENSG00000100219	3028	0.353738	0.000973	0.016885
RSPH10B	ENSG00000155026	4993	0.353917	0.003737	0.037967
RP11-127I20.5	ENSG00000267077	720	0.354135	0.000924	0.016451
KCTD16	ENSG00000183775	14294	0.35415	0.005456	0.047798
SNORD12C	ENSG00000209042	89	0.354553	0.003242	0.034763
TMEM187	ENSG00000177854	1924	0.354603	0.000361	0.009649
LINC00649	ENSG00000237945	14130	0.355132	0.000219	0.007419
TUBA1B	ENSG00000123416	3574	0.355395	0.001895	0.025074
MINOS1	ENSG00000173436	5227	0.355902	1.78E-07	0.000118
COPB1	ENSG00000129083	5131	0.355907	0.000213	0.007275
RPS13	ENSG00000110700	2186	0.355953	6.75E-05	0.004015

IARS	ENSG00000196305	7361	0.355977	0.001358	0.020422
EDARADD	ENSG00000186197	3726	0.356168	0.002254	0.027817
SNRPG	ENSG00000143977	1795	0.356489	0.000105	0.005013
PSMB8	ENSG00000204264	2006	0.356851	0.000502	0.011705
HSPE1	ENSG00000115541	1977	0.357823	3.82E-05	0.002964
RTCB	ENSG00000100220	2948	0.35843	1.15E-05	0.001467
SNORD49A	ENSG00000277370	71	0.358485	6.91E-06	0.001124
POMP	ENSG00000132963	1428	0.358582	4.03E-05	0.003037
ZNRD1	ENSG00000066379	3672	0.359045	4.63E-06	0.000878
HSPA4L	ENSG00000164070	11621	0.359258	0.001258	0.019567
RP11-324O2.3	ENSG00000232934	1120	0.36032	0.001667	0.023196
SMIM6	ENSG00000259120	1086	0.360582	0.001251	0.019535
MAD2L1	ENSG00000164109	5748	0.360704	0.005166	0.046389
LMO7-AS1	ENSG00000261105	593	0.361491	1.72E-05	0.001862
KPNA2	ENSG00000182481	3140	0.361635	0.003633	0.037412
CTC-260F20.3	ENSG00000258674	1846	0.361656	6.16E-05	0.003815
SSTR2	ENSG00000180616	7852	0.361741	0.000232	0.007671
CTD-3148I10.15	ENSG00000273189	1421	0.361813	0.001086	0.018105
RPL35A	ENSG00000182899	2977	0.361888	3.71E-05	0.002942
SEC11C	ENSG00000166562	4511	0.362001	0.000251	0.007973
AC108479.3	ENSG00000275955	127	0.362123	0.000245	0.007896
OSTC	ENSG00000198856	1617	0.362225	1.97E-05	0.002008
TOMM5	ENSG00000175768	3644	0.362514	7.19E-06	0.001136
NDUFA13	ENSG00000186010	2708	0.362807	5.52E-05	0.00368
NPM3	ENSG00000107833	928	0.363619	0.003823	0.038482
SLC39A8	ENSG00000138821	6480	0.364395	0.005681	0.048919
NDUFA4	ENSG00000189043	3556	0.364583	7.59E-05	0.004295
HIST1H4E	ENSG00000276966	312	0.364612	0.000528	0.012001
HIST1H2AM	ENSG00000278677	393	0.365156	0.00263	0.030582
HIST4H4	ENSG00000197837	2578	0.368477	6.51E-05	0.003911
SNORA58	ENSG00000201129	136	0.368644	0.003817	0.038438
MRPL54	ENSG00000183617	1003	0.368783	6.32E-05	0.003815
FUNDC1	ENSG00000069509	1399	0.369064	0.000963	0.016814
RPS7	ENSG00000171863	5583	0.369619	3.94E-06	0.000804
RP11-269F19.2	ENSG00000225721	765	0.369848	9.28E-05	0.004774
RRAGA	ENSG00000155876	1589	0.370138	1.36E-05	0.001625
RP11-663P9.2	ENSG00000124399	390	0.371143	0.002463	0.029312
CTD-2287O16.1	ENSG00000185641	373	0.371715	4.21E-05	0.003129
PIGY	ENSG00000255072	217	0.372604	1.04E-07	8.34E-05
SCG2	ENSG00000171951	2719	0.372938	0.003936	0.039173
SRPRB	ENSG00000144867	4180	0.373154	4.84E-05	0.00341

AP000304.12	ENSG00000249209	1078	0.37438	3.74E-05	0.002942
SMIM22	ENSG00000267795	1762	0.374456	0.000588	0.01278
PARK7	ENSG00000116288	2397	0.375141	2.29E-07	0.000133
CHRNA5	ENSG00000169684	3623	0.375526	0.003755	0.038108
MTFP1	ENSG00000242114	1696	0.37582	0.000191	0.006802
SNORA28	ENSG00000272533	126	0.376237	0.000609	0.012984
RP11-172H24.4	ENSG00000278291	4412	0.37675	0.001687	0.023357
TMEM70	ENSG00000175606	2982	0.376857	5.36E-07	0.000231
ZDHHC2	ENSG00000104219	6735	0.377189	0.005274	0.046955
RP11-386G11.10	ENSG00000258017	1632	0.37724	0.001689	0.02336
FBXO6	ENSG00000116663	1811	0.377357	0.00045	0.010918
RPL24P4	ENSG00000181524	421	0.377616	0.00012	0.005334
HIST1H4K	ENSG00000273542	312	0.377729	0.001033	0.017454
RPS27A	ENSG00000143947	3130	0.377911	2.56E-06	0.000625
MDH1	ENSG00000014641	4990	0.377972	2.27E-07	0.000133
COX11P1	ENSG00000231162	835	0.378562	0.002724	0.031384
AARS	ENSG00000090861	4498	0.379196	0.000159	0.006121
RPS3AP6	ENSG00000234797	795	0.379392	2.09E-05	0.002086
ATP5G3	ENSG00000154518	6780	0.379451	0.000104	0.005013
IFT20	ENSG00000109083	3227	0.379502	7.58E-05	0.004295
RPL22L1	ENSG00000163584	2326	0.379539	0.000234	0.007713
RPS21	ENSG00000171858	1023	0.379566	5.1E-05	0.003514
PSMB3	ENSG00000277791	1179	0.379608	1.71E-06	0.000494
CTC-518P12.6	ENSG00000267484	511	0.379873	0.002062	0.026538
RPS3A	ENSG00000145425	2341	0.381102	2.05E-06	0.000536
CLCN4	ENSG00000073464	6789	0.381179	0.002069	0.026558
NDUFA1	ENSG00000125356	766	0.38156	9.57E-06	0.001316
NDUFB6	ENSG00000165264	1388	0.381917	1.61E-05	0.001799
RPL26P30	ENSG00000236264	645	0.382128	0.000246	0.007896
RPL32	ENSG00000144713	3720	0.382463	3.24E-05	0.00276
SRGN	ENSG00000122862	1220	0.382798	0.003968	0.039397
NDUFB2	ENSG00000090266	4447	0.382878	3.13E-07	0.000158
TMEM126A	ENSG00000171202	971	0.382922	1.7E-06	0.000494
DNAJC17	ENSG00000104129	5633	0.383129	0.003915	0.03906
TIMM17A	ENSG00000134375	3513	0.384155	8.76E-05	0.004621
ATP6V1G1	ENSG00000136888	1305	0.384482	0.00012	0.005334
HIST2H2AB	ENSG00000184270	393	0.38454	0.003223	0.034693
PFDN2	ENSG00000143256	677	0.384604	8.87E-05	0.004674
SCARNA21	ENSG00000252835	139	0.385362	0.000508	0.011777
RPSAP6	ENSG00000214629	882	0.385555	0.001641	0.022974
RNU6-398P	ENSG00000206870	109	0.385847	0.000196	0.006911

RP11-650L12.2	ENSG00000261762	2154	0.386034	0.002941	0.032764
DBNDD2	ENSG00000244274	2764	0.386158	0.000111	0.00512
RPS14	ENSG00000164587	4544	0.386415	6.94E-05	0.004092
QPCT	ENSG00000115828	2599	0.386822	0.000242	0.007852
RPL39L	ENSG00000163923	1060	0.38778	0.003788	0.038272
TIMM8B	ENSG00000150779	1166	0.388106	2.06E-06	0.000536
SYP-AS1	ENSG00000237341	542	0.388799	0.000726	0.014282
CDK1	ENSG00000170312	3931	0.389727	0.004514	0.042518
TAS2R46	ENSG00000226761	930	0.389867	0.004196	0.040784
SLC25A5-AS1	ENSG00000224281	3065	0.390255	0.001349	0.020377
RP11-613M10.8	ENSG00000256966	3217	0.390283	1.14E-06	0.000396
COX7A2L	ENSG00000115944	5605	0.390951	1.37E-06	0.000446
C12orf57	ENSG00000111678	1806	0.390951	5.6E-05	0.00368
CD63	ENSG00000135404	2633	0.390995	1.01E-05	0.001367
RPS5	ENSG00000083845	3617	0.391293	8.07E-05	0.004442
TSPAN6	ENSG00000000003	4535	0.391781	0.000145	0.005803
DAD1	ENSG00000129562	808	0.392121	8.49E-05	0.004544
SNORD97	ENSG00000255125	441	0.392922	0.004571	0.042815
RNase_MRP	ENSG00000277027	264	0.39313	0.000411	0.010339
RMRP	ENSG00000269900	268	0.393137	0.00041	0.010339
IFI30	ENSG00000216490	2481	0.394024	8.66E-05	0.004578
HIST1H3J	ENSG00000197153	775	0.395104	0.004265	0.041215
RP11-793I11.1	ENSG00000254780	285	0.395222	0.000176	0.006536
HLTF-AS1	ENSG00000239718	415	0.39552	0.004089	0.04014
RPL9	ENSG00000163682	3259	0.395603	0.000124	0.005421
U4	ENSG00000272160	135	0.395985	0.000236	0.007735
SERPINI1	ENSG00000163536	2418	0.39611	0.004705	0.043626
IL17D	ENSG00000172458	3327	0.396379	0.001169	0.01884
RNU11	ENSG00000270103	131	0.396985	0.000902	0.016153
PPP1R14D	ENSG00000166143	873	0.397021	3.4E-05	0.002844
RPL31	ENSG00000071082	5791	0.397765	1.37E-05	0.001625
UQCR10	ENSG00000184076	975	0.398111	1.51E-05	0.001724
U11	ENSG00000274978	134	0.398393	0.000876	0.015873
CDKN1C	ENSG00000129757	2586	0.398756	0.001543	0.022125
RDH11	ENSG00000072042	5518	0.399653	0.005015	0.045419
PSMB5	ENSG00000100804	1765	0.399674	2.09E-05	0.002086
SLC25A42	ENSG00000181035	4519	0.4001	0.003602	0.037183
C19orf70	ENSG00000174917	1854	0.400511	7.55E-05	0.004295
SNX6P1	ENSG00000267920	1224	0.400704	0.001012	0.017266
CHCHD1	ENSG00000172586	1483	0.40216	1.43E-07	0.000103
AC021037.1	ENSG00000273577	87	0.402555	0.00015	0.005911

FDFT1	ENSG00000079459	5202	0.403156	0.000143	0.005757
PLS3	ENSG00000102024	4145	0.404992	0.000612	0.013028
LEAP2	ENSG00000164406	2405	0.405308	0.002229	0.027667
SYS1-DBNDD2	ENSG00000254806	1402	0.40534	5.08E-05	0.00351
PMVK	ENSG00000163344	1280	0.405619	0.000507	0.011777
SLC7A5	ENSG00000103257	4745	0.405763	0.00252	0.029831
RPL41P2	ENSG00000256338	409	0.406612	0.000112	0.005147
SNORD17	ENSG00000212232	237	0.40784	0.001221	0.019323
RPL34	ENSG00000109475	3393	0.408586	3.86E-06	0.000804
CDKN3	ENSG00000100526	1836	0.408602	0.001988	0.025837
LRRC31	ENSG00000114248	3113	0.408863	0.000306	0.008785
CYCS	ENSG00000172115	4457	0.409884	0.000399	0.010193
AC005387.3	ENSG00000268938	352	0.411309	0.004015	0.039715
CCL28	ENSG00000151882	4524	0.411401	0.000302	0.008715
WBP5	ENSG00000185222	1096	0.412549	0.00344	0.036185
GPD1	ENSG00000167588	3991	0.413337	0.002171	0.027278
RNU7-1	ENSG00000272173	1097	0.413683	3.3E-05	0.002786
MRPL14	ENSG00000180992	959	0.414076	1.03E-05	0.001378
RP1-5O6.5	ENSG00000235246	297	0.414225	0.000496	0.011614
TMEM258	ENSG00000134825	3068	0.414789	2.04E-06	0.000536
STYK1	ENSG00000060140	3424	0.414936	0.003127	0.034089
SEC61B	ENSG00000106803	1190	0.415543	2.81E-06	0.000664
UQCRHL	ENSG00000233954	2180	0.416733	5.05E-05	0.003505
GLRX5	ENSG00000182512	2845	0.417019	6.12E-05	0.003803
TDO2	ENSG00000151790	5264	0.417434	0.002356	0.028613
HIST1H4C	ENSG00000197061	435	0.41785	0.000383	0.009964
ST6GALNAC4	ENSG00000136840	2630	0.418741	0.003813	0.038429
PBK	ENSG00000168078	2165	0.418774	0.001009	0.017245
APLP1	ENSG00000105290	3656	0.41993	0.002552	0.029982
RP11-351I24.3	ENSG00000254719	208	0.420455	0.001452	0.021346
BST2	ENSG00000130303	1119	0.421324	0.005621	0.048585
SNORA20	ENSG00000207392	132	0.421474	0.004301	0.041395
RP3-325F22.5	ENSG00000272189	1894	0.421508	0.000294	0.008664
ACTRT3	ENSG00000184378	2005	0.422502	0.000254	0.008018
UBL5	ENSG00000198258	1931	0.42317	2.64E-07	0.000139
RPS7P11	ENSG00000213326	586	0.423683	1.26E-06	0.00042
RPL39	ENSG00000198918	2141	0.424413	1.63E-05	0.001807
PPA1	ENSG00000180817	5164	0.425096	2.48E-06	0.00061
RP11-554E23.4	ENSG00000257222	374	0.425692	4.92E-05	0.003429
C1QB	ENSG00000173369	1601	0.425937	0.003345	0.035524
HAUS1P3	ENSG00000253226	662	0.42679	0.004852	0.044398

TMEM97	ENSG00000109084	3034	0.427052	3.97E-05	0.003008
SS18L2	ENSG00000008324	1301	0.427093	2.76E-06	0.000664
ROMO1	ENSG00000125995	702	0.427232	0.000395	0.010139
UBE2T	ENSG00000077152	1065	0.428398	0.000598	0.012867
RPS20P21	ENSG00000244295	343	0.429432	0.000146	0.005819
SNORD3A	ENSG00000263934	699	0.429555	0.00579	0.049489
ACEA_U3	ENSG00000277813	218	0.429563	0.00579	0.049489
UQCRQ	ENSG00000164405	1472	0.431414	2.23E-05	0.002133
RPL23A	ENSG00000198242	2824	0.432167	1.72E-07	0.000117
GCH1	ENSG00000131979	2951	0.432989	0.000444	0.010822
NPC2	ENSG00000119655	2749	0.43383	2.7E-07	0.000139
MARCH4	ENSG00000144583	4447	0.433886	0.001782	0.024153
SCARNA10	ENSG00000239002	330	0.434225	9.6E-05	0.00485
HIST1H4B	ENSG00000278705	438	0.435084	0.001281	0.019765
CKS2	ENSG00000123975	613	0.437684	0.000107	0.005039
CTD-2636A23.2	ENSG00000261604	2497	0.438028	0.005094	0.045934
RHOBTB1	ENSG00000072422	4841	0.438327	0.005808	0.049545
SNORA74	ENSG00000223111	201	0.438827	0.004043	0.039884
ENO1P1	ENSG00000244457	1304	0.439017	0.000213	0.007271
SC5D	ENSG00000109929	5566	0.440888	0.003579	0.03713
HMGCR	ENSG00000113161	6215	0.440959	0.00025	0.007959
GDF9	ENSG00000164404	3279	0.443207	9.52E-05	0.004818
RP11-11N7.4	ENSG00000273175	535	0.443645	0.000971	0.016885
RP11-338K17.8	ENSG00000255839	321	0.443898	0.004238	0.041073
HIST1H4A	ENSG00000278637	312	0.443925	0.005795	0.0495
SNORD28	ENSG00000274544	75	0.446045	5.66E-05	0.0037
SNORD4A	ENSG00000264577	899	0.447143	1.87E-07	0.000119
RPS3AP5	ENSG00000178429	821	0.447359	0.001237	0.019426
SNORD116-14	ENSG00000206621	92	0.448636	0.004188	0.040762
HIST1H3F	ENSG00000277775	411	0.44937	0.004649	0.04327
RUSC1-AS1	ENSG00000225855	3516	0.449644	0.000642	0.013414
CTC-209H22.3	ENSG00000253172	496	0.4498	0.000826	0.015317
HIST1H4H	ENSG00000158406	397	0.449964	0.000213	0.007271
GHRL	ENSG00000157017	2064	0.450181	0.001335	0.020271
FAM160B1	ENSG00000151553	6216	0.450543	0.000279	0.008399
SNORA80E	ENSG00000207475	137	0.452444	0.004043	0.039884
SCOC	ENSG00000153130	6294	0.453388	2.27E-05	0.002158
PSMB9	ENSG00000240065	3072	0.453596	0.000306	0.008785
CLEC10A	ENSG00000132514	2469	0.455533	0.002742	0.031391
PRDX4	ENSG00000123131	1904	0.455762	3.97E-05	0.003008
FDPS	ENSG00000160752	3718	0.456082	0.000872	0.015849

GRHL1	ENSG00000134317	7952	0.456615	0.004653	0.043287
SNORA74B	ENSG00000212402	201	0.457583	0.004562	0.042762
ASGR1	ENSG00000141505	3748	0.458783	0.00016	0.006121
POFUT1	ENSG00000101346	6426	0.459422	7.42E-06	0.001153
LYPD6	ENSG00000187123	5033	0.459532	0.001357	0.020422
SNORA24	ENSG00000206903	131	0.460107	0.00312	0.034055
COX7A2	ENSG00000112695	1922	0.460174	5.17E-06	0.000947
SCARNA13	ENSG00000252481	275	0.460798	3.51E-05	0.002905
CD14	ENSG00000170458	1886	0.461376	0.001451	0.021346
RNVU1-14	ENSG00000207501	164	0.461552	0.000337	0.009337
ARRDC4	ENSG00000140450	4072	0.463782	0.001738	0.023815
NDUFB2-AS1	ENSG00000240889	1648	0.464317	7.62E-05	0.004306
EIF4EBP1	ENSG00000187840	1073	0.464341	0.001378	0.020614
RRM2	ENSG00000171848	4955	0.464636	0.004104	0.040214
SNORA84	ENSG00000239183	133	0.464856	0.000672	0.013697
RP11-196G11.1	ENSG00000255439	2716	0.465666	0.004178	0.040694
HIST1H2AH	ENSG00000274997	387	0.46679	0.003526	0.036762
TXNIP	ENSG00000265972	3625	0.467536	8.61E-05	0.004578
H3F3C	ENSG00000188375	1057	0.467771	0.001205	0.019138
SUCO	ENSG00000094975	7894	0.46925	0.000352	0.009555
SNORD100	ENSG00000221500	76	0.470065	0.000231	0.007661
DNAJC12	ENSG00000108176	2121	0.470785	2.24E-05	0.002135
FLJ22763	ENSG00000241224	2406	0.471049	0.000159	0.006121
MS4A4A	ENSG00000110079	2508	0.471699	0.00064	0.013406
GCHFR	ENSG00000137880	1531	0.471805	0.004853	0.044398
RP1-93H18.6	ENSG00000244158	530	0.472614	0.003162	0.034337
SNHG3	ENSG00000242125	4088	0.47384	0.000156	0.006081
MIR611	ENSG00000207601	67	0.473878	3.4E-06	0.000753
SNORD13	ENSG00000239039	104	0.474297	0.004053	0.039938
SNORD46	ENSG00000200913	104	0.47623	0.002241	0.027711
RP11-367G18.2	ENSG00000218208	447	0.477163	6.3E-05	0.003815
RPL7P1	ENSG00000214485	747	0.477402	1.91E-05	0.001966
GOLT1A	ENSG00000174567	1136	0.477416	0.000136	0.005622
SPAM1	ENSG00000106304	3032	0.477456	0.00017	0.006394
SNORA73A	ENSG00000274266	206	0.477869	0.00016	0.006121
LAPTM4B	ENSG00000104341	3197	0.478065	0.000166	0.006272
CTD-2571L23.6	ENSG00000269656	540	0.478278	0.000397	0.010174
TSPAN13	ENSG00000106537	2224	0.479044	5.13E-05	0.003526
CLCA1	ENSG00000016490	3340	0.479684	9.05E-05	0.004722
ACAT2	ENSG00000120437	4299	0.482067	5.26E-05	0.003579
SEC61G	ENSG00000132432	1842	0.482831	9.6E-08	7.95E-05

STX1A	ENSG00000106089	3693	0.483668	0.003101	0.033929
SCARNA12	ENSG00000238795	270	0.483985	7.9E-05	0.004407
IL20RA	ENSG00000016402	4730	0.48841	0.000335	0.009304
ADORA3	ENSG00000121933	4486	0.48918	0.00185	0.024726
KB-1107E3.1	ENSG00000253629	361	0.489242	0.004243	0.041087
SNORA1	ENSG00000206834	130	0.490387	7.61E-06	0.001163
NME1	ENSG00000239672	3438	0.490623	0.000543	0.012171
DIRAS3	ENSG00000162595	1973	0.490748	0.002559	0.030015
CTD-2531D15.5	ENSG00000255126	590	0.492077	0.005194	0.046564
MIR3609	ENSG00000266019	80	0.492952	5.55E-05	0.00368
CCDC167	ENSG00000198937	619	0.493118	8.13E-05	0.004448
RN7SL5P	ENSG00000265735	321	0.496137	0.001726	0.023704
SCARNA5	ENSG00000252010	276	0.497922	1.38E-05	0.001625
NSDHL	ENSG00000147383	1996	0.500372	3.92E-05	0.002999
AC079250.1	ENSG00000230979	531	0.501127	0.00017	0.006388
HIST2H3D	ENSG00000183598	411	0.5019	0.005264	0.046916
AC147651.4	ENSG00000237181	731	0.502347	0.003294	0.035126
SLC35B1	ENSG00000121073	5919	0.504213	1.07E-06	0.000381
U12	ENSG00000276027	150	0.504749	3.4E-05	0.002844
HMGCS1	ENSG00000112972	4702	0.508271	0.000398	0.010193
ARL4A	ENSG00000122644	3561	0.508396	7.05E-05	0.004117
VKORC1	ENSG00000167397	2191	0.509605	0.002581	0.030203
FFAR4	ENSG00000186188	4060	0.510518	0.003697	0.037762
SNORA55	ENSG00000201457	135	0.510545	0.001485	0.021643
RNU12	ENSG00000270022	664	0.510917	2.16E-05	0.002118
CIDEC	ENSG00000187288	1926	0.512037	0.000651	0.01352
C6orf99	ENSG00000203711	859	0.512203	0.000129	0.005521
RNU5A-1	ENSG00000199568	116	0.513386	0.001538	0.022097
LDHB	ENSG00000111716	2983	0.51542	8.15E-05	0.004448
MCM6	ENSG00000076003	4118	0.51631	0.000116	0.005253
ST13P4	ENSG00000232150	1093	0.516874	1.57E-06	0.000483
ATP5EP2	ENSG00000180389	385	0.518568	1.91E-06	0.000526
ABHD6	ENSG00000163686	3589	0.519256	0.001199	0.019091
RPL24P2	ENSG00000235065	475	0.519425	0.001608	0.022727
TTR	ENSG00000118271	2173	0.519815	0.001149	0.018671
CTD-3014M21.1	ENSG00000279602	1321	0.520386	0.002561	0.030027
EIF4A1	ENSG00000161960	5316	0.520582	0.000238	0.007788
HSD17B14	ENSG00000087076	1281	0.5215	0.004232	0.041038
RPSA	ENSG00000168028	3936	0.522735	1.58E-06	0.000483
MIR19A	ENSG00000277328	82	0.52422	0.000326	0.009157
SNORA45B	ENSG00000212607	131	0.524849	0.000136	0.005622

RN7SL2	ENSG00000280102	374	0.525003	0.000194	0.006855
RN7SL1	ENSG00000258486	300	0.525029	0.002737	0.031391
SNORA10	ENSG00000206811	133	0.526427	0.004076	0.040054
SCGN	ENSG00000079689	1482	0.526764	2.63E-07	0.000139
SCG5	ENSG00000166922	2196	0.5277	0.002741	0.031391
FABP2	ENSG00000145384	2478	0.527706	0.001512	0.021859
SUCLA2P1	ENSG00000224936	1370	0.529218	7.26E-06	0.001141
RNaseP_nuc	ENSG00000277209	333	0.531022	2.45E-05	0.00228
RPPH1	ENSG00000259001	638	0.531025	2.45E-05	0.00228
H2AFZ	ENSG00000164032	2303	0.531678	8.35E-08	7.64E-05
SNORA8	ENSG00000207304	139	0.531879	0.005053	0.045667
SNORA70	ENSG00000207165	135	0.532168	0.000217	0.007367
UBE2Q2P6	ENSG00000237550	717	0.53237	0.002262	0.027841
RP1-99E18.2	ENSG00000218996	599	0.533735	3.62E-05	0.002905
REG1A	ENSG00000115386	1725	0.53438	0.001017	0.017296
SCARNA6	ENSG00000251791	265	0.534393	6.21E-05	0.003815
SNORA74A	ENSG00000200959	198	0.53574	9.01E-05	0.004712
RP11-963H4.3	ENSG00000263508	774	0.536553	0.000232	0.007661
RMDN3	ENSG00000137824	5114	0.53719	6.98E-06	0.001124
SEC22C	ENSG00000093183	8159	0.539351	0.000136	0.005622
MIR4709	ENSG00000265649	72	0.539409	5.24E-08	5.52E-05
SNORA44	ENSG00000273544	132	0.542175	0.001292	0.019886
SLC16A14	ENSG00000163053	4946	0.544727	0.00237	0.0287
SNORD22	ENSG00000277194	126	0.545308	0.001313	0.020094
FBXO8	ENSG00000164117	2810	0.545772	8.69E-07	0.000323
SVOP	ENSG00000166111	7270	0.54589	0.000106	0.005029
RP11-671J11.5	ENSG00000279423	671	0.546337	0.00252	0.029831
U1	ENSG00000206828	164	0.546658	0.001033	0.017454
SNORA38B	ENSG00000200394	131	0.546788	0.00154	0.022097
SNHG15	ENSG00000232956	3647	0.547499	0.000149	0.005876
DHCR7	ENSG00000172893	4160	0.550605	0.003004	0.033226
GGH	ENSG00000137563	3130	0.552977	0.000147	0.005854
SNHG8	ENSG00000269893	923	0.553561	4.25E-07	0.000198
SNORA26	ENSG00000212588	122	0.555685	0.003581	0.03713
IDI1	ENSG00000067064	3589	0.556941	0.002816	0.032012
RNY1	ENSG00000201098	113	0.558624	8.96E-05	0.004712
SNORA64	ENSG00000207187	133	0.560998	0.003825	0.038482
RP11-15B17.1	ENSG00000245322	1714	0.561294	3.51E-08	3.99E-05
SNORA21	ENSG00000199293	132	0.562292	0.001514	0.021862
SNORA76A	ENSG00000206952	134	0.563268	0.001549	0.022148
RGS4	ENSG00000117152	4836	0.565029	0.000193	0.006848

RPL18A	ENSG00000105640	3527	0.566233	1.89E-05	0.001956
APOH	ENSG00000091583	1332	0.569069	0.004462	0.042328
RP11-215A21.2	ENSG00000236058	549	0.576885	0.000141	0.005753
RP1-292B18.1	ENSG00000219747	405	0.579095	0.000551	0.012245
SCARNA23	ENSG00000251869	130	0.584415	0.000109	0.005083
HIST1H4D	ENSG00000277157	312	0.587839	4.54E-05	0.003264
FABP4	ENSG00000170323	1396	0.590702	0.005714	0.0491
RP11-36C20.1	ENSG00000240376	629	0.590729	3.44E-06	0.000753
RPS29	ENSG00000213741	9783	0.59543	5.49E-07	0.000231
RNU4ATAC	ENSG00000264229	127	0.597782	1.39E-05	0.001625
RAB30	ENSG00000137502	11832	0.597949	0.00223	0.027667
SNORA23	ENSG00000201998	182	0.602321	1.5E-05	0.001714
SNHG12	ENSG00000197989	3377	0.604214	0.000246	0.007896
MSMO1	ENSG00000052802	2892	0.605869	0.001861	0.024805
SNORA48	ENSG00000209582	135	0.607335	0.000204	0.007154
CCK	ENSG00000187094	2085	0.607922	2.69E-05	0.002475
TCP1P1	ENSG00000229161	1610	0.608338	0.005503	0.047997
SNORA25	ENSG00000207112	134	0.60895	1.73E-06	0.000494
RBP4	ENSG00000138207	1449	0.610162	0.00426	0.041211
SNORD105B	ENSG00000238531	79	0.611803	0.00044	0.01077
CCR2	ENSG00000121807	3733	0.615006	0.000643	0.01342
C11orf86	ENSG00000173237	1187	0.617873	0.001947	0.025428
MMD	ENSG00000108960	3177	0.619234	0.00026	0.008123
RP11-216N14.7	ENSG00000223599	1309	0.63175	9.82E-05	0.004908
RNU1-122P	ENSG00000202408	156	0.634254	0.005247	0.046854
RP11-288G3.4	ENSG00000234115	1241	0.641052	0.000738	0.014385
TFF3	ENSG00000160180	1168	0.645573	7.04E-07	0.000279
GZMH	ENSG00000100450	951	0.649135	0.002035	0.026326
HPS5	ENSG00000110756	6433	0.649662	1.11E-05	0.00143
SNORA16A	ENSG00000274582	137	0.650491	0.005679	0.048919
HIST2H3DP1	ENSG00000213244	416	0.653476	0.000744	0.01445
RIMBP3C	ENSG00000183246	6105	0.655261	0.00042	0.010464
RNVU1-6	ENSG00000201558	164	0.657187	0.000506	0.011758
SNORA75	ENSG00000206885	137	0.658265	0.000338	0.009337
MIR4426	ENSG00000231767	466	0.666055	5.44E-05	0.003661
SNORA33	ENSG00000200534	130	0.671416	6.22E-08	6.08E-05
RP11-535A19.1	ENSG00000254814	758	0.671574	0.004124	0.040342
ABHD5	ENSG00000011198	3172	0.673221	2.07E-05	0.002082
SNORA77	ENSG00000221643	125	0.679472	0.004273	0.041239
RPS20P35	ENSG00000244086	357	0.683493	0.002628	0.030573
ASNS	ENSG00000070669	4455	0.688607	0.001554	0.022162

LIPG	ENSG00000101670	13112	0.688702	0.000177	0.006546
GZMB	ENSG00000100453	1411	0.692338	0.0022	0.027516
METTL12	ENSG00000214756	3185	0.694206	4.96E-05	0.003447
SCARNA3	ENSG00000252906	143	0.694757	1.81E-05	0.001926
RN7SL674P	ENSG00000239899	275	0.695824	0.000675	0.013717
LRG1	ENSG00000171236	3133	0.696794	0.000845	0.0155
KDM8	ENSG00000155666	4039	0.697179	5.84E-05	0.003767
SHBG	ENSG00000129214	1972	0.69881	0.000815	0.015231
DGAT2	ENSG00000062282	7905	0.70647	0.002086	0.026693
IGHV3-11	ENSG00000211941	473	0.707914	0.002275	0.02792
CXCL2	ENSG00000081041	1500	0.708454	0.002523	0.029834
CHRDL2	ENSG00000054938	3135	0.70869	0.003706	0.037778
SNORA57	ENSG00000206597	149	0.711271	5.18E-05	0.003541
RNU2-63P	ENSG00000222724	194	0.711628	0.003269	0.034935
RPL9P7	ENSG00000238103	580	0.716395	0.004676	0.043455
CTD-3203P2.1	ENSG00000259940	1686	0.720191	3.56E-05	0.002905
ACSL1	ENSG00000151726	6284	0.721577	0.000221	0.007424
SNHG9	ENSG00000255198	551	0.722228	0.000478	0.011314
KRT222	ENSG00000213424	3169	0.724544	0.001497	0.021728
KLK12	ENSG00000186474	1769	0.725047	0.003759	0.038135
AC002480.5	ENSG00000225541	1282	0.735062	0.003841	0.038553
RENBP	ENSG00000102032	2057	0.735661	0.002973	0.032964
SCARNA18	ENSG00000238835	134	0.740064	9.92E-06	0.001356
CTD-2028E8.2	ENSG00000279701	967	0.744134	0.000728	0.014283
SNORA54	ENSG00000207008	123	0.749566	0.000417	0.010418
SLC25A5P6	ENSG00000213332	886	0.750055	0.000305	0.008785
SNORA9	ENSG00000277184	133	0.755418	0.000745	0.01445
SNORA7A	ENSG00000207496	139	0.75623	0.000628	0.013214
SCARNA4	ENSG00000252808	129	0.769269	3.02E-05	0.002645
SNORA71D	ENSG00000200354	134	0.775874	0.002969	0.032931
SNORA76C	ENSG00000266402	278	0.776983	0.001508	0.021833
SNORA6	ENSG00000206760	151	0.78131	6.48E-07	0.000261
AC002480.4	ENSG00000232949	557	0.783338	0.003293	0.035126
SNORA68	ENSG00000207166	133	0.784972	0.000188	0.006761
LINC00888	ENSG00000240024	2131	0.786561	2.79E-06	0.000664
RP11-357D18.1	ENSG00000250978	837	0.793386	0.002128	0.026998
SNORA79	ENSG00000221303	140	0.804865	3.52E-05	0.002905
SNORA31	ENSG00000199477	130	0.815465	2.94E-09	5.03E-06
SNORA78	ENSG00000273587	127	0.816962	0.002406	0.028928
AJUBA	ENSG00000129474	5979	0.819161	6.92E-05	0.004092
RN7SL4P	ENSG00000263740	295	0.831754	7.36E-07	0.000287

AC005077.7	ENSG00000224273	631	0.832459	0.002672	0.030956
G0S2	ENSG00000123689	866	0.832919	0.000823	0.015262
G6PC2	ENSG00000152254	3125	0.836332	0.003774	0.038204
TMEM107	ENSG00000179029	3163	0.849829	2.19E-06	0.000553
RNU6ATAC	ENSG00000221676	126	0.85025	0.000187	0.006739
RP3-340B19.2	ENSG00000219023	392	0.853899	4.7E-09	7.13E-06
SNORA14A	ENSG00000201643	135	0.856351	3.18E-05	0.002755
AC139452.2	ENSG00000227337	471	0.862467	7.42E-05	0.004237
SNORA45A	ENSG00000200983	130	0.864164	0.001707	0.023559
RNU5F-1	ENSG00000199377	117	0.866461	0.000662	0.01364
RNVU1-19	ENSG00000275538	167	0.873971	0.000538	0.012135
CTC-518B2.9	ENSG00000268906	485	0.87434	0.000301	0.008715
SNORA14B	ENSG00000207181	135	0.875228	1.35E-06	0.000443
SNORA2A	ENSG00000206612	135	0.877303	0.001615	0.022774
RNU1-120P	ENSG00000199879	157	0.889292	0.000937	0.016547
RP11-293M10.2	ENSG00000258820	874	0.898994	0.003408	0.036005
LPL	ENSG00000175445	4382	0.914276	0.005655	0.048805
SNORD118	ENSG00000200463	134	0.923872	3.89E-06	0.000804
RP11-356M20.1	ENSG00000259674	802	0.935465	0.003659	0.037563
PSAT1	ENSG00000135069	2188	0.965304	0.000818	0.015231
RP11-96A15.1	ENSG00000278464	670	0.968346	0.005437	0.047734
SNORA17	ENSG00000274998	133	0.976415	0.001807	0.024341
PAPPA2	ENSG00000116183	10930	0.977559	5.42E-06	0.000971
NTS	ENSG00000133636	1555	1.010749	0.00085	0.015538
SNORA62	ENSG00000202363	153	1.015419	6.86E-06	0.001124
SNORD15A	ENSG00000206941	148	1.020583	8.41E-06	0.001248
DUOX2	ENSG00000140279	9834	1.02766	0.000958	0.016763
SCARNA14	ENSG00000252712	136	1.120089	1.92E-06	0.000526
PLIN2	ENSG00000147872	5086	1.123463	2.26E-06	0.000566
SNORA63	ENSG00000199363	131	1.149263	0.000104	0.005013
MFSD2A	ENSG00000168389	4007	1.156549	1.28E-05	0.001566
SNORA61	ENSG00000278274	130	1.216831	8.99E-06	0.001298
IGHV2-70	ENSG00000211974	434	1.238835	0.000681	0.013776
INHBE	ENSG00000139269	3015	1.254452	0.000713	0.014094
RP11-587D21.1	ENSG00000240036	376	1.256618	0.00043	0.010606
LCN2	ENSG00000148346	1598	1.276131	0.000157	0.006082
CHAC1	ENSG00000128965	2058	1.283768	0.000156	0.006076
PNLIPRP1	ENSG00000187021	4986	1.335258	0.000541	0.012167
CPXM2	ENSG00000121898	4859	1.366175	0.001191	0.019039
CELA2B	ENSG00000215704	1457	1.410338	0.001233	0.019408
TRBV6-1	ENSG00000211706	401	1.443923	0.001078	0.018056

DUOXA2	ENSG00000140274	2554	1.501411	0.004332	0.041534
TREML2	ENSG00000112195	3302	1.526715	0.000435	0.010655
RN7SL3	ENSG00000279868	297	1.526718	1.26E-05	0.00155
NLRP2	ENSG00000022556	6313	1.573741	1.12E-05	0.00143
RNU1-1	ENSG00000206652	164	1.599571	0.003539	0.036851
RN7SL272P	ENSG00000242360	289	1.60585	0.002551	0.029982
CEL	ENSG00000170835	2681	1.605983	0.000147	0.005857
SNORD3B-2	ENSG00000262074	688	1.636701	5.31E-05	0.00359
PLA2G1B	ENSG00000170890	638	1.764413	0.000931	0.016514
TRIB3	ENSG00000101255	3434	1.824858	5.59E-05	0.00368
UBD	ENSG00000213886	1087	1.911491	0.000388	0.010037
CELA3B	ENSG00000219073	1541	1.975439	0.000221	0.007424
SNORD3B-1	ENSG00000265185	583	2.019492	5.59E-06	0.000986
PRSS1	ENSG00000204983	2081	2.166156	0.000136	0.005622
AC091154.1	ENSG00000276078	89	2.464243	0.000137	0.005622
RP11-689K5.3	ENSG00000251331	699	2.481136	4.78E-06	0.000901
SNORD3C	ENSG00000264940	581	3.528362	1.32E-08	1.71E-05
CTRC	ENSG00000162438	3486	3.5289	3.05E-05	0.002664
CPB1	ENSG00000153002	5703	3.622689	2.79E-05	0.002526
CELA3A	ENSG00000142789	1224	4.215595	1.17E-06	0.0004
CTRB2	ENSG00000168928	2346	4.977091	0.000312	0.008868
U8	ENSG00000206987	133	5.028652	0.005498	0.047993
CELA2A	ENSG00000142615	1519	5.030251	7.73E-06	0.001169
CTRB1	ENSG00000168925	1016	5.185435	3.54E-05	0.002905
CLPS	ENSG00000137392	601	6.432123	0.000596	0.012854
CPA1	ENSG00000091704	2921	6.526719	3.19E-06	0.000721

Abbreviations: FC, Fold change; FDR, false discovery rate

Supplementary Table 2. List of genes differentially expressed in DM that were also common to MitoCarta 2.0

Symbol	log2FC	Fold change	PValue	FDR
NDUFAF5	0.146024248	1.10651595	0.001586599	0.022487924
PDHA1	0.154233049	1.112829869	0.005767201	0.049321566
YARS2	0.155089554	1.113490735	0.004240152	0.041080793
DARS2	0.160672892	1.117808377	0.002896167	0.032425924
SDHC	0.160758398	1.117874629	0.002740253	0.031391004
DIABLO	0.165224858	1.121340836	0.005645685	0.048755105
YME1L1	0.166396827	1.122252124	0.004588117	0.042883086
SCO1	0.166450774	1.12229409	0.003221505	0.034690501
PARL	0.169786387	1.124891915	0.002090781	0.026712174
FUNDC2	0.171209541	1.126002118	0.001498524	0.021727703
DAP3	0.171288373	1.126063647	0.00226234	0.027840925
MLH1	0.171313565	1.12608331	0.001198746	0.019091342
GDAP1	0.172431287	1.126956076	0.002885975	0.032373712
TIMM10B	0.172718734	1.127180637	0.002784446	0.031720638
CMC2	0.173343057	1.127668527	0.000525378	0.011980286
MPV17	0.174976394	1.128945933	0.004518963	0.042543806
SDHAF2	0.176753347	1.130337301	0.000576641	0.012663411
SLC25A17	0.179130106	1.132201003	0.001165231	0.018835704
ATP10D	0.180915952	1.13360337	0.001488177	0.02165421
MALSU1	0.181200342	1.133826853	0.000878384	0.015873034
KARS	0.181243078	1.13386044	0.001784943	0.024171441
MTO1	0.18151697	1.13407572	0.00585046	0.049804088
PPA2	0.182278783	1.134674726	0.003773379	0.038203547
PTRH2	0.183753131	1.135834888	0.000166576	0.006284792
MRPS18C	0.184028067	1.136051366	0.004506643	0.042502979
NIPSNAP3A	0.184773247	1.13663831	0.003824486	0.03848151
MRPS22	0.187955824	1.139148495	0.002781898	0.031704827
PDHX	0.188281644	1.139405791	0.00451399	0.042517752
NGRN	0.189606223	1.140452392	0.00127338	0.019703154
HSDL1	0.191243121	1.141747096	0.005322716	0.0472035
SAMM50	0.191325589	1.141812364	0.002222827	0.027666901
MRPS14	0.19461654	1.14441994	0.000566398	0.01250879
COQ5	0.195057087	1.144769458	0.004296481	0.04137728
TFAM	0.195915105	1.145450492	0.000935984	0.01654696
MRPL48	0.197619225	1.146804305	0.003185261	0.034435831
TEFM	0.197723816	1.146887447	0.000270566	0.008274659
FTSJ2	0.198219946	1.14728192	0.000566952	0.012510919
NDUFV2	0.199284086	1.148128474	0.003450513	0.036256906

MRPS17	0.20093314	1.149441577	0.00488336	0.0445945
RNMTL1	0.201374553	1.149793319	0.005860328	0.049868422
DLD	0.201376788	1.1497951	0.001137214	0.018551655
NFU1	0.201401865	1.149815086	0.005145048	0.046227653
COX4I1	0.203777704	1.151710169	0.004189396	0.040762378
MIPEP	0.204803002	1.152528959	0.000693934	0.013899522
CYB5B	0.207038312	1.154316071	0.001845455	0.024697306
METTL5	0.207477403	1.154667446	0.003585365	0.037129614
TXNDC12	0.207795406	1.154921989	0.000309208	0.008833926
LYRM2	0.208031884	1.155111313	0.000710149	0.014079904
TIMM21	0.209566866	1.156340969	0.003324427	0.035339482
COA7	0.213044402	1.159131626	0.004495496	0.042497101
TIMMDC1	0.213328695	1.159360063	6.26363E-05	0.003814758
FASTKD1	0.215111864	1.160793917	0.000157884	0.006105684
MRPS21	0.215153691	1.160827571	0.000613032	0.013033361
MRPL57	0.216527531	1.161933523	0.000919181	0.016372206
TIMM23	0.216928965	1.162256879	0.002233182	0.027676648
MRPL33	0.217065049	1.162366516	0.00191548	0.025190549
MTX2	0.217101929	1.16239623	1.84876E-05	0.001936668
NUDT5	0.217222971	1.162493759	1.54295E-05	0.001750447
GOT2	0.217445011	1.162672688	0.000578348	0.012680518
SSBP1	0.217859181	1.163006517	0.000684741	0.013816612
MRPL35	0.218113292	1.163211382	0.000930157	0.016503197
HCCS	0.218397563	1.163440606	0.001049728	0.01769686
GRPEL2	0.220347018	1.165013778	0.003071603	0.033706266
COQ2	0.220442598	1.165090965	0.001491536	0.021691531
OMA1	0.22097676	1.165522423	0.005351442	0.047350735
ISCA2	0.222193419	1.16650575	0.000107259	0.005038797
ATP5F1	0.222581228	1.166819359	0.0040978	0.040200196
CISD1	0.222900475	1.167077588	0.00531344	0.047151818
PTPMT1	0.22290409	1.167080512	0.00048397	0.011407084
FASTKD2	0.223540308	1.1675953	0.000272727	0.008331434
TMEM205	0.223805619	1.167810039	0.004608652	0.042990501
DHRS7B	0.223821783	1.167823124	0.003295369	0.035126241
MRPS23	0.223886806	1.167875759	0.001102261	0.01828147
SMDT1	0.22644021	1.169944595	0.001917254	0.025201746
MRPS16	0.226632506	1.170100547	0.000463612	0.011105115
PRELID2	0.227035472	1.170427419	0.002293813	0.028085597
ATP5A1	0.22946036	1.172396333	0.003726331	0.037892223
TOMM6	0.229815969	1.172685352	0.000292248	0.008641564
UQCRFS1	0.230628233	1.173345781	0.002246045	0.027751266

THNSL1	0.231845203	1.174335961	0.002874194	0.032351364
CHCHD3	0.233207001	1.175444971	0.001645806	0.023016876
OXSM	0.233462944	1.175653521	0.000230262	0.007646623
GFM1	0.234749748	1.176702607	0.000118511	0.00531184
MPC2	0.235772144	1.177536798	0.000667192	0.013662094
HSPA9	0.23995777	1.180958092	0.002086382	0.026693391
TPI1	0.240579325	1.181466993	0.002377274	0.028732582
ATP5C1	0.241421049	1.182156508	0.00087019	0.015829588
ATP5J2-PTCD1	0.242009721	1.182638969	0.002489142	0.029576546
VDAC3	0.242551676	1.183083316	0.000242592	0.007858649
SLC25A3	0.242856344	1.183333186	0.001259503	0.019566816
PDHB	0.242868759	1.183343369	0.000177174	0.006546084
MARS2	0.243136365	1.183562888	0.002381333	0.028732582
ATP5I	0.2436636	1.183995501	0.003486616	0.036523973
NDUFB11	0.243800121	1.184107547	0.002537556	0.029879118
NDUFS3	0.244055668	1.184317309	0.00206581	0.026557875
MRPL18	0.245948587	1.185872238	0.001363079	0.020454417
ATP5B	0.245980593	1.185898547	0.001606023	0.022723509
MRPL22	0.246548817	1.18636572	9.20315E-05	0.004763662
HSPD1	0.248303466	1.187809492	0.00068968	0.013871915
GFM2	0.249545386	1.188832438	1.11845E-06	0.000392044
NDUFB5	0.249672107	1.188936865	0.001249365	0.019530524
NDUFB8	0.250260249	1.189421656	0.001816958	0.024449225
GRPEL1	0.25069684	1.189781656	0.000809866	0.015194917
NDUFB4	0.251758394	1.190657435	0.001831721	0.02457364
RARS	0.252412813	1.191197651	0.000735051	0.014355013
PRDX3	0.252434186	1.191215297	0.00025703	0.008059015
MRPL39	0.254062798	1.192560781	0.000553252	0.012276486
MRPL27	0.25449519	1.192918259	0.000190957	0.00680228
ATP5G2	0.256179993	1.194312182	0.001426519	0.021162488
TMEM14C	0.256299275	1.194410932	0.000145256	0.005806186
MRPL20	0.256391142	1.194486991	0.000212915	0.007271412
IDH1	0.257079901	1.195057389	0.001454128	0.021349275
GHITM	0.257193049	1.195151119	0.002589348	0.030267369
FH	0.257710565	1.195579915	0.00034973	0.009516104
NDUFA7	0.258640308	1.196350653	0.005004816	0.045355216
MRPL3	0.258642142	1.196352173	0.000384977	0.010014887
MRPL47	0.258767356	1.196456012	0.002532184	0.029855204
TOMM22	0.258962039	1.196617477	0.000424821	0.010539968
COX16	0.259114697	1.196744103	0.000549125	0.012232454

NDUFS4	0.259741227	1.197263935	0.000411858	0.01035564
C2orf47	0.260513225	1.197904772	0.000584228	0.012727786
MRPL50	0.261531864	1.198750872	0.000804722	0.015152831
MRPL36	0.262316925	1.199403364	0.002733475	0.031391004
CCT7	0.262323902	1.199409165	4.8252E-05	0.003408934
TRIAP1	0.262535113	1.199584771	1.84865E-05	0.001936668
FLAD1	0.262870457	1.199863639	0.000608131	0.012979642
PACSIN2	0.263165468	1.200109019	0.004546957	0.042706406
CHCHD4	0.263462778	1.200356363	0.000255649	0.008024925
PHB	0.264032177	1.200830209	0.001084741	0.018105209
MRPS33	0.267379576	1.203619659	0.000833429	0.015344632
FAM162A	0.267976526	1.204117789	0.000279248	0.00839925
KIAA0391	0.268252094	1.204347809	0.000108949	0.005082511
NDUFC1	0.268606874	1.204644011	0.000275826	0.008351451
MRPL11	0.268719615	1.204738154	0.00058322	0.012726107
CHCHD2	0.269921723	1.205742405	0.001235337	0.019421385
SLC25A11	0.270550189	1.206267764	0.000975139	0.016906317
APOO	0.270798958	1.206475784	0.001340897	0.020311068
NDUFA2	0.271501594	1.207063517	0.002127127	0.026998067
COA3	0.271808979	1.207320725	2.98349E-05	0.002622884
COX7B	0.276597047	1.21133428	0.001863492	0.024805015
CHCHD5	0.277658448	1.212225796	0.003043064	0.033548549
TIMM17B	0.277751447	1.21230394	0.001925033	0.025270235
MMACHC	0.283829602	1.217422219	0.000846198	0.015500393
ATP5J2	0.284025598	1.217587623	0.001747423	0.023912059
MMAB	0.284195155	1.217730731	0.002216462	0.027644922
NDUFAF1	0.285666472	1.218973254	0.000133369	0.00560438
COA6	0.286396127	1.219589916	0.000456046	0.011002896
NDUFAF4	0.286743181	1.219883335	0.004520309	0.042543806
MRPL51	0.287213378	1.220280979	2.96369E-05	0.00261388
MRPL17	0.288317474	1.221215219	0.000245127	0.007895962
ATP5J	0.288619228	1.221470675	0.000354902	0.009560361
NDUFS5	0.288904016	1.221711817	0.001003796	0.01718877
HSD17B10	0.28906531	1.221848413	0.001781838	0.024153315
MRPS7	0.289248913	1.22200392	0.000738353	0.014388678
ACAA2	0.289289576	1.222038363	0.00089563	0.016067857
PET100	0.289842287	1.222506628	0.000539396	0.012137956
STOML2	0.291933454	1.22427992	0.000117071	0.005270659
GPX1	0.292173968	1.224484039	0.001413322	0.021012309
PRDX5	0.293243129	1.225391823	0.002217377	0.027644922
NCEH1	0.293832671	1.225892668	0.000235657	0.007734803

COX5B	0.294455866	1.226422326	0.000665428	0.013658767
COX7C	0.295684249	1.227467008	9.33213E-05	0.004774114
NDUFA12	0.295705164	1.227484804	0.001568643	0.022314394
COX8A	0.296134957	1.227850538	0.004593566	0.042883086
RPL10A	0.297034411	1.228616285	0.000678847	0.013758613
NDUFA9	0.297424987	1.228948949	0.000187998	0.006754357
NDUFC2	0.298841478	1.230156169	9.66836E-05	0.004873057
COX14	0.299483519	1.230703746	8.63393E-05	0.004578287
ATP5G1	0.299798366	1.230972358	0.001010778	0.01726152
MMADHC	0.300334802	1.231430154	7.23504E-05	0.004182101
NDUFS6	0.30059816	1.231654968	0.000795842	0.01507622
TMEM126B	0.300814308	1.231839511	3.60454E-05	0.002905301
SLC25A5	0.300951364	1.231956542	0.000274369	0.008346487
NDUFA6	0.301482349	1.232410047	0.000350334	0.009521342
CROT	0.301907816	1.232773552	0.002594597	0.030302804
BLOC1S1	0.302262299	1.233076493	0.000163184	0.006205308
COX6A1	0.303881734	1.234461407	0.001030088	0.01744959
SDHD	0.304232489	1.234761571	0.000200726	0.007056272
TMBIM4	0.304284897	1.234806427	1.03834E-05	0.001378118
DBI	0.306856627	1.23700954	0.000831566	0.015330983
PSMA6	0.307207729	1.237310623	6.29756E-05	0.003814758
PYURF	0.309223484	1.239040619	2.64145E-07	0.000138973
TRMT10C	0.310061215	1.239760303	8.30464E-05	0.004483651
TOMM7	0.310708514	1.240316675	9.73601E-05	0.004884264
C16orf91	0.311981519	1.241411589	0.001758091	0.023974053
C7orf55	0.311983937	1.24141367	0.000987884	0.016987251
NDUFB1	0.314509151	1.243588475	0.000319545	0.009034841
HINT1	0.315382656	1.244341655	4.15527E-06	0.000823256
SFXN4	0.315905123	1.244792371	9.96074E-05	0.004941817
RPS18	0.31604448	1.244912617	0.000832144	0.015331307
C14orf2	0.317490559	1.246161075	0.000181541	0.006639242
TARS	0.317559233	1.246220395	0.002533341	0.029855204
COQ3	0.318993675	1.247460103	0.000142281	0.005754582
NDUFAB1	0.320801263	1.249024056	0.000155428	0.00607632
ATP5E	0.324041258	1.251832258	1.18354E-05	0.00148437
RPS15A	0.330457601	1.257412143	0.000211931	0.007270268
NDUFA11	0.331769105	1.258555733	0.000268813	0.008240475
ATP5L	0.334851699	1.261247753	2.70656E-05	0.002483227
BOLA3	0.33511339	1.261476552	0.001851204	0.024725828
SLIRP	0.335855895	1.262125957	2.31806E-05	0.002185453
NDUFB9	0.336531653	1.262717275	0.000144898	0.005806186

UQCRH	0.33690374	1.263042986	0.000130566	0.005534595
NDUFB7	0.338142108	1.264127611	0.001084565	0.018105209
PHB2	0.338203724	1.264181602	0.000226665	0.007585373
USMG5	0.339174968	1.265032955	0.000646766	0.013478073
MRPL15	0.339191576	1.265047518	6.55266E-06	0.001112771
COX6C	0.340341624	1.266056355	7.82721E-05	0.004379729
NDUFA8	0.343126174	1.268502334	5.43307E-06	0.000970886
MRPL13	0.344360075	1.269587716	1.73462E-06	0.000494023
GARS	0.344874082	1.270040129	0.003828002	0.038492604
RTN4IP1	0.34508348	1.27022448	4.48052E-06	0.000859693
ATP5O	0.346035641	1.271063089	6.22579E-05	0.003814758
UQCR11	0.346574964	1.271538341	6.0904E-05	0.003803187
MRPL34	0.3493433	1.273980592	5.84264E-05	0.00376693
SLC25A20	0.351592572	1.275968375	3.18371E-05	0.002754615
MPC1	0.351671355	1.276038055	0.000726139	0.014282375
MINOS1	0.355901536	1.279785066	1.77565E-07	0.00011841
HSPE1	0.357823049	1.281490736	3.81603E-05	0.002964035
RPL35A	0.361887689	1.285106291	3.71127E-05	0.002941993
TOMM5	0.362514453	1.285664713	7.1891E-06	0.001136168
NDUFA13	0.362806927	1.285925379	5.52009E-05	0.003680404
NDUFA4	0.364582868	1.287509314	7.58695E-05	0.004294716
MRPL54	0.368783333	1.291263411	6.31718E-05	0.003814758
FUNDC1	0.369063884	1.291514539	0.000963034	0.016814303
PARK7	0.375140691	1.296966028	2.28905E-07	0.00013316
MTFP1	0.375820303	1.297577135	0.000190591	0.00680228
TMEM70	0.376857023	1.298509909	5.36163E-07	0.000231135
MDH1	0.377971707	1.299513578	2.27008E-07	0.00013316
ATP5G3	0.379451229	1.300846946	0.000104047	0.005013008
NDUFA1	0.381559748	1.302749543	9.575E-06	0.001315528
NDUFB6	0.381917463	1.303072598	1.61199E-05	0.001798912
NDUFB2	0.382877813	1.303940295	3.12837E-07	0.000158394
TMEM126A	0.382921702	1.303979964	1.70483E-06	0.000494023
TIMM17A	0.384154505	1.305094709	8.75541E-05	0.004621265
RPS14	0.386415208	1.307141396	6.94484E-05	0.004092217
TIMM8B	0.388106395	1.308674579	2.05771E-06	0.000535807
COX7A2L	0.390950759	1.311257258	1.36995E-06	0.000445903
UQCR10	0.398110521	1.3177809	1.51334E-05	0.001724004
RDH11	0.399653487	1.319191023	0.00501523	0.045419477
SLC25A42	0.400099933	1.319599314	0.003601677	0.037182806
C19orf70	0.40051144	1.319975764	7.54586E-05	0.004294716
CHCHD1	0.402159798	1.32148477	1.42793E-07	0.00010274

RPL34	0.408586159	1.32738434	3.86E-06	0.000804157
CYCS	0.40988373	1.328578736	0.00039863	0.010192935
MRPL14	0.414075969	1.332444987	1.03291E-05	0.001378118
GLRX5	0.417018991	1.335165879	6.11766E-05	0.003803187
ROMO1	0.427231705	1.344650936	0.000394938	0.010138979
UQCRQ	0.431414371	1.348555008	2.22598E-05	0.002132932
PRDX4	0.455762045	1.371507057	3.96667E-05	0.003008214
FDPS	0.456081535	1.371810816	0.000871909	0.015849058
COX7A2	0.460174165	1.375707886	5.17312E-06	0.000946517
LDHB	0.515420374	1.429410582	8.14967E-05	0.00444751
RMDN3	0.537190161	1.45114347	6.97994E-06	0.001123729
IDI1	0.556940963	1.471146544	0.002815851	0.032011721
ACSL1	0.721576781	1.648983292	0.000221039	0.007424364

Abbreviations: FC, fold change; FDR, false discovery rate

Supplementary Table 3. Differentially expressed (FDR < 0.05) mitochondrial protein genes grouped into functional categories

Gene	Description	LogFC
**Nuclear-DNA OXPHOS genes**		
NDUFA1	"NADH dehydrogenase (ubiquinone) 1 alpha subcomplex, 1, 7.5kDa"	0.38156
NDUFA11	"NADH dehydrogenase (ubiquinone) 1 alpha subcomplex, 11, 14.7kDa"	0.331769
NDUFA12	"NADH dehydrogenase (ubiquinone) 1 alpha subcomplex, 12"	0.295705
NDUFA13	"NADH dehydrogenase (ubiquinone) 1 alpha subcomplex, 13"	0.362807
NDUFA2	"NADH dehydrogenase (ubiquinone) 1 alpha subcomplex, 2, 8kDa"	0.271502
NDUFA4	"NADH dehydrogenase (ubiquinone) 1 alpha subcomplex, 4, 9kDa"	0.364583
NDUFA6	"NADH dehydrogenase (ubiquinone) 1 alpha subcomplex, 6, 14kDa"	0.301482
NDUFA7	"NADH dehydrogenase (ubiquinone) 1 alpha subcomplex, 7, 14.5kDa"	0.25864
NDUFA8	"NADH dehydrogenase (ubiquinone) 1 alpha subcomplex, 8, 19kDa"	0.343126
NDUFA9	"NADH dehydrogenase (ubiquinone) 1 alpha subcomplex, 9, 39kDa"	0.297425
NDUFAB1	"NADH dehydrogenase (ubiquinone) 1, alpha/beta subcomplex, 1, 8kDa"	0.320801
NDUFB1	"NADH dehydrogenase (ubiquinone) 1 beta subcomplex, 1, 7kDa"	0.314509
NDUFB11	"NADH dehydrogenase (ubiquinone) 1 beta subcomplex, 11, 17.3kDa"	0.2438
NDUFB2	"NADH dehydrogenase (ubiquinone) 1 beta subcomplex, 2, 8kDa"	0.382878
NDUFB4	"NADH dehydrogenase (ubiquinone) 1 beta subcomplex, 4, 15kDa"	0.251758
NDUFB5	"NADH dehydrogenase (ubiquinone) 1 beta subcomplex, 5, 16kDa"	0.249672
NDUFB6	"NADH dehydrogenase (ubiquinone) 1 beta subcomplex, 6, 17kDa"	0.381917
NDUFB7	"NADH dehydrogenase (ubiquinone) 1 beta subcomplex, 7, 18kDa"	0.338142
NDUFB8	"NADH dehydrogenase (ubiquinone) 1 beta subcomplex, 8, 19kDa"	0.25026
NDUFB9	"NADH dehydrogenase (ubiquinone) 1 beta subcomplex, 9, 22kDa"	0.336532
NDUFC1	"NADH dehydrogenase (ubiquinone) 1, subcomplex	0.268607

	unknown, 1, 6kDa"	
NDUFC2	"NADH dehydrogenase (ubiquinone) 1, subcomplex unknown, 2, 14.5kDa"	0.298841
NDUFC2-KCTD14	NDUFC2-KCTD14 Readthrough	0.333788
NDUFS3	"NADH dehydrogenase (ubiquinone) Fe-S protein 3, 30kDa (NADH-coenzyme Q reductase)"	0.244056
NDUFS4	"NADH dehydrogenase (ubiquinone) Fe-S protein 4, 18kDa (NADH-coenzyme Q reductase)"	0.259741
NDUFS5	"NADH dehydrogenase (ubiquinone) Fe-S protein 5, 15kDa (NADH-coenzyme Q reductase)"	0.288904
NDUFS6	"NADH dehydrogenase (ubiquinone) Fe-S protein 6, 13kDa (NADH-coenzyme Q reductase)"	0.300598
NDUFV2	"NADH dehydrogenase (ubiquinone) flavoprotein 2, 24kDa"	0.199284
SDHC	"succinate dehydrogenase complex, subunit C, integral membrane protein, 15kDa"	0.160758
SDHD	"succinate dehydrogenase complex, subunit D, integral membrane protein"	0.304232
UQCR10	"ubiquinol-cytochrome c reductase, complex III subunit X"	0.398111
UQCR11	"ubiquinol-cytochrome c reductase, complex III subunit XI"	0.346575
UQCRFS1	"ubiquinol-cytochrome c reductase, Rieske iron-sulfur polypeptide 1"	0.230628
UQCRH	ubiquinol-cytochrome c reductase hinge protein	0.336904
UQCRHL	Ubiquinol-Cytochrome C Reductase Hinge Protein Like	0.416733
UQCRQ	"ubiquinol-cytochrome c reductase, complex III subunit VII, 9.5kDa"	0.431414
COX4I1	cytochrome c oxidase subunit IV isoform 1	0.203778
COX5B	cytochrome c oxidase subunit Vb	0.294456
COX6A1	cytochrome c oxidase subunit VIa polypeptide 1	0.303882
COX6C	cytochrome c oxidase subunit VIc	0.340342
COX7A2	cytochrome c oxidase subunit VIIa polypeptide 2 (liver)	0.460174
COX7A2L	cytochrome c oxidase subunit VIIa polypeptide 2 like	0.390951
COX7B	cytochrome c oxidase subunit VIIb	0.276597
COX7C	cytochrome c oxidase subunit VIIc	0.295684
COX8A	cytochrome c oxidase subunit VIIIA (ubiquitous)	0.296135
atp5a1	"ATP synthase, H+ transporting, mitochondrial F1 complex, alpha subunit 1, cardiac muscle"	0.22946
atp5b	"ATP synthase, H+ transporting, mitochondrial F1 complex, beta polypeptide"	0.245981
atp5c1	"ATP synthase, H+ transporting, mitochondrial F1	0.241421

	complex, gamma polypeptide 1"	
atp5e	"ATP synthase, H+ transporting, mitochondrial F1 complex, epsilon subunit"	0.324041
atp5f1	"ATP synthase, H+ transporting, mitochondrial Fo complex, subunit B1"	0.222581
atp5g1	"ATP synthase, H+ transporting, mitochondrial Fo complex, subunit C1 (subunit 9)"	0.299798
atp5g2	"ATP synthase, H+ transporting, mitochondrial Fo complex, subunit C2 (subunit 9)"	0.25618
atp5g3	"ATP synthase, H+ transporting, mitochondrial Fo complex, subunit C3 (subunit 9)"	0.379451
atp5i	"ATP synthase, H+ transporting, mitochondrial Fo complex, subunit E"	0.243664
atp5j	"ATP synthase, H+ transporting, mitochondrial Fo complex, subunit F6"	0.288619
atp5j2	"ATP synthase, H+ transporting, mitochondrial Fo complex, subunit F2"	0.284026
atp5l	"ATP synthase, H+ transporting, mitochondrial Fo complex, subunit G"	0.334852
atp5o	"ATP synthase, H+ transporting, mitochondrial F1 complex, O subunit"	0.346036
**Other genes related to ETC**		
COA6	cytochrome c oxidase assembly factor 6 homolog (S. cerevisiae)	0.286396
COA3	cytochrome c oxidase assembly factor 3	0.271809
COA7	cytochrome c oxidase assembly factor 7 (putative)	0.213044
COQ5	"coenzyme Q5 homolog, methyltransferase (S. cerevisiae)"	0.195057
COQ3	coenzyme Q3 methyltransferase	0.318994
COQ2	coenzyme Q2 4-hydroxybenzoate polyprenyltransferase	0.220443
CMC2	C-x(9)-C motif containing 2	0.173343
PET100	PET100 homolog (S. cerevisiae)	0.289842
HCCS	holocytochrome c synthase	0.218398
MIPEP	mitochondrial intermediate peptidase	0.204803
CYCS	"cytochrome c, somatic"	0.409884
USMG5	up-regulated during skeletal muscle growth 5 homolog (mouse)	0.339175
**Carbohydrate metabolism**		
DLD	dihydrolipoamide dehydrogenase	0.201377
FH	fumarate hydratase	0.257711
IDH1	"isocitrate dehydrogenase 1 (NADP+), soluble"	0.25708
MDH1	"malate dehydrogenase 1, NAD (soluble)"	0.377972
PDHA1	pyruvate dehydrogenase (lipoamide) alpha 1	0.154233

PDHB	pyruvate dehydrogenase (lipoamide) beta	0.242869
SDHC	"succinate dehydrogenase complex, subunit C, integral membrane protein, 15kDa"	0.160758
SDHD	"succinate dehydrogenase complex, subunit D, integral membrane protein"	0.304232
MPC2	mitochondrial pyruvate carrier 2	0.235772
MPC1	mitochondrial pyruvate carrier 1	0.351671
ADH5	Alcohol Dehydrogenase 5 (Class III), Chi Polypeptide	0.216433
AKR1A1	Aldo-keto reductase family 1, member A1	0.255086
BPGM	Bisphosphoglycerate Mutase	0.24898
DLD	dihydrolipoamide dehydrogenase	0.201377
ENO1	Enolase 1	0.301366
G6PC2	Glucose-6-Phosphatase Catalytic Subunit 2	0.836332
LDHB	lactate dehydrogenase B	0.51542
PDHA1	pyruvate dehydrogenase (lipoamide) alpha 1	0.154233
PDHB	pyruvate dehydrogenase (lipoamide) beta	0.242869
PGK1	Phosphoglycerate Kinase 1	0.245175
TPI1	triosephosphate isomerase 1	0.240579
**Mitochondrial quality control**		
HSPA9	heat shock 70kDa protein 9 (mortalin)	0.239958
HSPD1	heat shock 60kDa protein 1 (chaperonin)	0.248303
HSPE1	heat shock 10kDa protein 1	0.357823
YME1L1	YME1-like 1 ATPase	0.166397
OMA1	OMA1 zinc metallopeptidase	0.220977
TFAM	Transcription factor A, mitochondrial	0.195915
**Mitophagy**		
FUNDC1	FUN14 domain containing 1	0.369064
PHB2	prohibitin 2	0.338204
PARK7	parkinson protein 7	0.375141
PARL	"presenilin associated, rhomboid-like"	0.169786
**Protein translocation into mitochondria**		
TIMM8B	translocase of inner mitochondrial membrane 8 homolog B (yeast)	0.388106
TIMM17A	translocase of inner mitochondrial membrane 17 homolog A (yeast)	0.384155
TIMM21	translocase of inner mitochondrial membrane 21 homolog (yeast)	0.209567
TIMM17B	translocase of inner mitochondrial membrane 17 homolog B (yeast)	0.277751
TIMM23	translocase of inner mitochondrial membrane 23 homolog (yeast)	0.216929
TIMM10B	translocase of inner mitochondrial membrane 10	0.172719

	homolog B (yeast)	
TIMMDC1	translocase of inner mitochondrial membrane domain containing 1	0.213329
MPV17	MpV17 mitochondrial inner membrane protein	0.174976
CHCHD5	coiled-coil-helix-coiled-coil-helix domain containing 5	0.277658
SMDT1	single-pass membrane protein with aspartate-rich tail 1	0.22644
MINOS1	mitochondrial inner membrane organizing system 1	0.355902
CHCHD3	coiled-coil-helix-coiled-coil-helix domain containing 3	0.233207
CHCHD4	coiled-coil-helix-coiled-coil-helix domain containing 4	0.263463
TMEM126A	transmembrane protein 126A	0.382922
TMEM70	transmembrane protein 70	0.376857
TMEM14C	transmembrane protein 14C	0.256299
TMEM205	transmembrane protein 205	0.223806
TMEM126B	transmembrane protein 126B	0.300814
TOMM7	translocase of outer mitochondrial membrane 7 homolog (yeast)	0.310709
TOMM22	translocase of outer mitochondrial membrane 22 homolog (yeast)	0.258962
TOMM6	translocase of outer mitochondrial membrane 6 homolog (yeast)	0.229816
TOMM5	translocase of outer mitochondrial membrane 5 homolog (yeast)	0.362514
SAMM50	SAMM50 sorting and assembly machinery component	0.191326
MTX2	metaxin 2	0.217102
VDAC3	voltage-dependent anion channel 3	0.242552
GRPEL1	"GrpE-like 1, mitochondrial (E. coli)"	0.250697
GRPEL2	"GrpE-like 2, mitochondrial (E. coli)"	0.220347
**Mitochondrial translation**		
MRPS23	mitochondrial ribosomal protein S23	0.223887
MRPL34	mitochondrial ribosomal protein L34	0.349343
MRPS14	mitochondrial ribosomal protein S14	0.194617
MRPS17	mitochondrial ribosomal protein S17	0.200933
MRPL17	mitochondrial ribosomal protein L17	0.288317
MRPL11	mitochondrial ribosomal protein L11	0.26872
MRPL13	mitochondrial ribosomal protein L13	0.34436
MRPL33	mitochondrial ribosomal protein L33	0.217065
MRPL15	mitochondrial ribosomal protein L15	0.339192
MRPS7	mitochondrial ribosomal protein S7	0.289249

MRPL3	mitochondrial ribosomal protein L3	0.258642
MRPL36	mitochondrial ribosomal protein L36	0.262317
MRPS18C	mitochondrial ribosomal protein S18C	0.184028
MRPL20	mitochondrial ribosomal protein L20	0.256391
MRPS21	mitochondrial ribosomal protein S21	0.215154
MRPL22	mitochondrial ribosomal protein L22	0.246549
MRPL47	mitochondrial ribosomal protein L47	0.258767
MRPS16	mitochondrial ribosomal protein S16	0.226633
MRPL27	mitochondrial ribosomal protein L27	0.254495
MRPL51	mitochondrial ribosomal protein L51	0.287213
MRPL18	mitochondrial ribosomal protein L18	0.245949
MRPL54	mitochondrial ribosomal protein L54	0.368783
MRPL35	mitochondrial ribosomal protein L35	0.218113
MRPL57	mitochondrial ribosomal protein L57	0.216528
MRPL50	mitochondrial ribosomal protein L50	0.261532
MRPL14	mitochondrial ribosomal protein L14	0.414076
MRPS22	mitochondrial ribosomal protein S22	0.187956
MRPS33	mitochondrial ribosomal protein S33	0.26738
MRPL39	mitochondrial ribosomal protein L39	0.254063
MRPL48	mitochondrial ribosomal protein L48	0.197619
YARS2	"tyrosyl-tRNA synthetase 2, mitochondrial"	0.15509
MARS2	"methionyl-tRNA synthetase 2, mitochondrial"	0.243136
DARS2	"aspartyl-tRNA synthetase 2, mitochondrial"	0.160673
GARS	glycyl-tRNA synthetase	0.344874
KARS	lysyl-tRNA synthetase	0.181243
TARS	threonyl-tRNA synthetase	0.317559
RARS	arginyl-tRNA synthetase	0.252413
GFM1	"G elongation factor, mitochondrial 1"	0.23475
GFM2	"G elongation factor, mitochondrial 2"	0.249545
TRMT10C	tRNA methyltransferase 10 homolog C (S. cerevisiae)	0.310061
RNMTL1	RNA methyltransferase like 1	0.201375
MTO1	mitochondrial tRNA translation optimization 1	0.181517
CHCHD1	coiled-coil-helix-coiled-coil-helix domain containing 1	0.40216
DAP3	death associated protein 3	0.171288
FTSJ2	FtsJ RNA methyltransferase homolog 2 (E. coli)	0.19822
MALSU1	mitochondrial assembly of ribosomal large subunit 1	0.1812
**Fe-S cluster assembly**		
ISCA2	iron-sulfur cluster assembly 2	0.222193
UQCRFS1	"ubiquinol-cytochrome c reductase, Rieske iron-sulfur polypeptide 1"	0.230628

NFU1	NFU1 iron-sulfur cluster scaffold homolog (S. cerevisiae)	0.201402
CISD1	CDGSH iron sulfur domain 1	0.2229
GLRX5	glutaredoxin 5	0.417019
BOLA3	bolA family member 3	0.335113
**Mitochondrial transporters**		
SLC25A3	"solute carrier family 25 (mitochondrial carrier; phosphate carrier), member 3"	0.242856
SLC25A20	"solute carrier family 25 (carnitine/acylcarnitine translocase), member 20"	0.351593
SLC25A11	"solute carrier family 25 (mitochondrial carrier; oxoglutarate carrier), member 11"	0.27055
SLC25A42	"solute carrier family 25, member 42"	0.4001
SLC25A5	"solute carrier family 25 (mitochondrial carrier; adenine nucleotide translocator), member 5"	0.300951
SLC25A17	"solute carrier family 25 (mitochondrial carrier; peroxisomal membrane protein, 34kDa), member 17"	0.17913
**Antioxidant genes**		
GPX1	glutathione peroxidase 1	0.292174
PRDX3	peroxiredoxin 3	0.252434
PRDX4	peroxiredoxin 4	0.455762
PRDX5	peroxiredoxin 5	0.293243
ROMO1	reactive oxygen species modulator 1	0.427232

Abbreviations: FDR, false discovery rate; FC, fold change

Supplementary Table 4. Differentially expressed genes in type 1 and type 2 diabetes mellitus

		T1DM vs Control		T2DM vs Control	
Symbol	Ensembl ID	log2FC	FDR	log2FC	FDR
LUC7L	ENSG00000007392	-0.2561717	0.020322	-0.29107	0.018995
RP11-96C23.13	ENSG00000151303	-0.5599426	0.008917	-0.60363	0.003833
MAB21L3	ENSG00000173212	-0.6765525	0.001103	-0.55437	0.04632
IRS2	ENSG00000185950	-0.6308078	0.027633	-0.56182	0.042392
KRT5	ENSG00000186081	-2.818464	0.048197	-3.65685	0.035675
MT-ND6	ENSG00000198695	-1.2263923	0.002505	-1.00379	0.03824
MT-ND1	ENSG00000198888	-1.2722522	0.000821	-1.10323	0.009463
MT-ATP6	ENSG00000198899	-0.9963554	0.000341	-0.91969	0.000698
MT-RNR2	ENSG00000210082	-1.3736696	0.002505	-1.39282	0.00826
MT-RNR1	ENSG00000211459	-2.3614739	4.17E-06	-2.34382	4.71E-05
MT-ND4L	ENSG00000212907	-1.2071991	0.000487	-0.96116	0.019481
RNA45S5	ENSG00000225840	-3.9922617	1.21E-07	-4.21218	2.39E-08
RNA28S5	ENSG00000226958	-5.2331639	2.55E-10	-5.46535	3.85E-09
MT-ATP8	ENSG00000228253	-1.2365491	3.32E-05	-1.14857	0.000337
KLF3-AS1	ENSG00000231160	-0.4400777	0.013127	-0.48887	0.008452
MTRNR2L4	ENSG00000232196	-1.1071813	0.008202	-1.11995	0.015286
RP1-122P22.2	ENSG00000233895	-0.3429591	0.026814	-0.44681	0.02349
RP11-69I8.2	ENSG00000236673	-0.820645	0.019855	-0.83647	0.040175
BMS1P3	ENSG00000240089	-0.5909965	0.02043	-0.62614	0.015012
MTATP8P1	ENSG00000240409	-1.5322122	2.55E-07	-1.27309	0.000351
RN7SL438P	ENSG00000241774	-3.8703949	0.004105	-3.96436	0.003965
RN7SL434P	ENSG00000241868	-0.9946898	0.015878	-1.04527	0.020193
MTATP6P1	ENSG00000248527	-1.1331421	3.32E-05	-1.01559	0.000351
LINC00939	ENSG00000249267	-2.4070137	0.007563	-2.91245	0.000664
MTRNR2L1	ENSG00000256618	-2.9663933	1.16E-05	-2.02297	0.026507
RP3-405J10.4	ENSG00000257256	-0.6482887	0.002906	-0.5701	0.028493
RP11-680F8.4	ENSG00000259644	-0.527206	0.016268	-0.51019	0.019186
RP11-96C23.11	ENSG00000261011	-0.5735926	0.026583	-0.74305	0.003965
RP11-846F4.9	ENSG00000264316	-2.9056012	0.009506	-3.35126	0.021211
CTD-2104P17.2	ENSG00000264932	-0.5303005	0.048958	-0.63282	0.029243
MTRNR2L12	ENSG00000269028	-1.8422783	0.001119	-1.57001	0.033921
RP11-345P4.6	ENSG00000269227	-0.6354254	0.022949	-0.77677	0.020137
BMS1P7	ENSG00000270025	-0.7154225	0.003144	-0.63607	0.038272
AC002128.5	ENSG00000271366	-0.4137993	0.049487	-0.47657	0.034305
AGAP11	ENSG00000271880	-0.5696366	0.01632	-0.67523	0.002472

RP11-96C23.14	ENSG00000272508	-0.5965901	0.012522	-0.64205	0.005691
MIR6087	ENSG00000275110	-3.19458	0.004627	-3.26729	0.012189
AL353644.10	ENSG00000277048	-2.3748232	1.04E-06	-2.46317	2.13E-06
NLRP2	ENSG00000022556	1.60170078	0.012404	1.573323	0.015422
SCGN	ENSG00000079689	0.51997213	0.008965	0.536767	0.009463
CPA1	ENSG00000091704	3.7161611	0.027074	7.46787	0.000236
LIPG	ENSG00000101670	0.7327793	0.043684	0.647614	0.029527
SRSF7	ENSG00000115875	0.33951751	0.004627	0.307107	0.013895
COX7A2L	ENSG00000115944	0.38854805	0.012673	0.402745	0.030606
NPC2	ENSG00000119655	0.40396009	0.007775	0.464385	0.019186
SEC61G	ENSG00000132432	0.54567099	0.001119	0.414254	0.041663
PLIN2	ENSG00000147872	1.11806111	0.000399	1.1374	0.020137
H2AFZ	ENSG00000164032	0.58692544	0.00043	0.472161	0.043262
PPA1	ENSG00000180817	0.44415857	0.008186	0.416929	0.041685
RPL23A	ENSG00000198242	0.48583429	0.001807	0.376411	0.020505
SNORA31	ENSG00000199477	0.98448513	4.17E-06	0.599536	0.034065
SNORA33	ENSG00000200534	0.72250844	0.002354	0.60487	0.008419
RN7SKP189	ENSG00000201912	1.01072383	0.008254	0.883733	0.038272
SNORA25	ENSG00000207112	0.59032345	0.010031	0.623441	0.024314
RPL7P1	ENSG00000214485	0.51950558	0.030828	0.430633	0.041685
RP3-340B19.2	ENSG00000219023	1.00951977	3.21E-05	0.666707	0.034065
SPCS2P4	ENSG00000228589	0.34586195	0.022053	0.340118	0.037321
ST13P4	ENSG00000232150	0.48819334	0.015135	0.553027	0.036012
FLJ43681	ENSG00000232938	0.8746482	0.035448	1.382582	0.035371
RP11-15B17.1	ENSG00000245322	0.62914863	7.18E-05	0.487368	0.046926
RP11-689K5.3	ENSG00000251331	2.74856282	0.001103	2.046488	0.028493
SNORD4A	ENSG00000264577	0.50233601	0.002505	0.387108	0.037321
SNORD3C	ENSG00000264940	4.31089344	2.55E-07	1.232051	0.003648
SNORD3B-1	ENSG00000265185	2.10395409	0.001397	1.937477	0.019481
MIR4709	ENSG00000265649	0.51872046	0.006016	0.559012	0.015098
RP11-728B21.3	ENSG00000271266	0.89639777	0.031739	0.925359	0.041685
ACEA_U3	ENSG00000275303	4.31956809	2.55E-07	1.238813	0.003343
AC091154.1	ENSG00000276078	1.99406735	0.037134	2.878737	0.01261
ACEA_U3	ENSG00000276271	2.34952543	0.002673	2.174284	0.030606
Metazoa_SRP	ENSG00000278771	1.60979221	0.008208	1.442101	0.01527
RN7SL3	ENSG00000279868	1.60979171	0.008208	1.442101	0.01527

Abbreviation: DM, diabetes mellitus; FC, fold change; FDR, false discovery rate

Supplementary Table 5. Spearman's correlation between Principal Components (PC1, 2 and 3) and loadings (Bolded values are statistically significant)

Genes	PC1	PC2	PC3
MT-ND1	**-0.5711**[c]	**-0.5769**[c]	**0.3383**[a]
MT-ND2	**-0.4735**[b]	**-0.6753**[c]	**0.3449**[a]
MT-ND3	**-0.3603**[a]	**-0.3591**[a]	-0.2036
MT-ND4	**-0.4358**[b]	**-0.7211**[c]	**0.4555**[b]
MT-ND4L	**-0.4751**[b]	**-0.7055**[c]	**0.5257**[c]
MT-ND5	**-0.4848**[b]	**-0.6506**[c]	**0.5271**[c]
MT-ND6	**-0.4059**[a]	**-0.7771**[c]	**0.4304**[b]
NDUFA1	**0.8791**[c]	-0.2998	-0.1291
NDUFA11	**0.8227**[c]	-0.0401	0.186
NDUFA12	**0.9144**[c]	**-0.4279**[b]	0.0366
NDUFA13	**0.8852**[c]	-0.0038	0.0194
NDUFA2	**0.8723**[c]	-0.1269	0.0706
NDUFA4	**0.9144**[c]	-0.1585	-0.0445
NDUFA6	**0.9399**[c]	-0.2899	0.0018
NDUFA7	**0.9032**[c]	-0.0935	0.0941
NDUFA8	**0.8858**[c]	**-0.3294**[a]	0.2158
NDUFA9	**0.9028**[c]	**-0.3257**[a]	0.152
NDUFAB1	**0.9093**[c]	-0.1842	0.1229
NDUFB1	**0.8154**[c]	**-0.3826**[a]	-0.2502
NDUFB11	**0.7087**[c]	0.1453	0.0449
NDUFB2	**0.8609**[c]	-0.1136	0.0202
NDUFB4	**0.8316**[c]	**-0.4298**[b]	-0.0474
NDUFB5	**0.8263**[c]	**-0.4115**[b]	-0.2095
NDUFB6	**0.9302**[c]	-0.2794	-0.0761
NDUFB7	**0.6735**[c]	0.1308	0.1739
NDUFB8	**0.9549**[c]	-0.2498	0.0654
NDUFB9	**0.8974**[c]	**-0.4364**[b]	0.0794
NDUFC1	**0.8895**[c]	-0.2464	0.0245
NDUFC2	**0.7909**[c]	**-0.4144**[b]	0.0482
NDUFC2-KCTD14	**0.8455**[c]	**-0.3547**[a]	0.1421
NDUFS3	**0.952**[c]	-0.2213	0.1338
NDUFS4	**0.8889**[c]	**-0.3445**[a]	-0.0109
NDUFS5	**0.8615**[c]	-0.2383	0.1466
NDUFS6	**0.8451**[c]	**-0.3387**[a]	0.1387
NDUFV2	**0.8927**[c]	-0.3034	-0.0415
SDHC	**0.6937**[c]	**-0.6362**[c]	0.3073
SDHD	**0.8611**[c]	-0.2381	0.0567
MT-CYB	**-0.4771**[b]	**-0.7047**[c]	**0.4119**[b]

UQCR10	**0.9656**[c]	-0.2593	0.0579
UQCR11	**0.9306**[c]	-0.0682	-0.0182
UQCRFS1	**0.8628**[c]	**-0.3265**[a]	0.2158
UQCRH	**0.8565**[c]	**-0.3532**[a]	-0.0287
UQCRHL	**0.7196**[c]	-0.2909	0.1314
UQCRQ	**0.915**[c]	-0.0911	0.0016
MT-CO1	-0.1433	**-0.7818**[c]	0.2326
MT-CO2	0.0243	**-0.8524**[c]	0.2895
COX4I1	**0.9002**[c]	-0.23	0.0848
COX5B	**0.9071**[c]	-0.0423	0.1087
COX6A1	**0.9385**[c]	-0.1075	0.0818
COX6C	**0.9091**[c]	-0.2551	-0.1567
COX7A2	**0.9211**[c]	-0.1344	0.07
COX7A2L	**0.8233**[c]	**-0.3534**[a]	0.0285
COX7B	**0.9215**[c]	-0.2939	-0.104
COX7C	**0.9158**[c]	-0.2275	-0.2123
COX8A	**0.9071**[c]	0.0277	0.1441
MT-ATP6	**-0.434**[b]	**-0.7393**[c]	**0.4385**[b]
MT-ATP8	**-0.3652**[a]	**-0.732**[c]	**0.3553**[a]
atp5a1	**0.867**[c]	**-0.3429**[a]	0.1239
atp5b	**0.8777**[c]	-0.2927	0.2686
atp5c1	**0.9172**[c]	**-0.3609**[a]	-0.081
atp5e	**0.7575**[c]	-0.1194	**-0.3457**[a]
atp5f1	**0.9071**[c]	**-0.3872**[a]	0.0915
atp5g1	**0.8407**[c]	-0.2383	0.266
atp5g2	**0.781**[c]	-0.0132	-0.13
atp5g3	**0.8955**[c]	-0.2733	0.2261
atp5i	**0.919**[c]	-0.2498	-0.0364
atp5j	**0.881**[c]	**-0.436**[b]	-0.0006
atp5j2	**0.8447**[c]	**-0.4176**[b]	0.1164
atp5l	**0.9543**[c]	-0.2077	-0.1211
atp5o	**0.9109**[c]	**-0.3551**[a]	0.0271

Abbreviations: PC, principal component
a: 0.01< P < 0.05, b: 0.001 < P < 0.01, c : P < 0.001

Supplementary Table 6. Differentially expressed miRNAs in DGE compared to Controls

ID	Symbol	Expr Log Ratio	Expr p-value	Confidence
hsa-miR-101-3p	miR-101-3p (and other miRNAs w/seed ACAGUAC)	-0.689	1.12E-11	High (predicted)
hsa-miR-127-5p	miR-127-5p (miRNAs w/seed UGAAGCU)	-0.651	3.45E-08	High (predicted)
hsa-miR-1296-5p	miR-1296-5p (miRNAs w/seed UAGGGCC)	-0.537	0.00000402	High (predicted)
hsa-miR-1307-5p	miR-1307-5p (miRNAs w/seed CGACCGG)	-0.555	0.0000421	High (predicted)
hsa-miR-130a-3p	miR-130a-3p (and other miRNAs w/seed AGUGCAA)	-0.521	0.00000131	High (predicted)
hsa-miR-136-3p	miR-136-3p (miRNAs w/seed AUCAUCG)	-0.561	0.00000606	High (predicted)
hsa-miR-138-5p	miR-138-5p (miRNAs w/seed GCUGGUG)	-0.55	0.000735	High (predicted)
hsa-miR-1468-5p	miR-1468-5p (miRNAs w/seed UCCGUUU)	0.602	0.0000122	High (predicted)
hsa-miR-15a-5p	miR-16-5p (and other miRNAs w/seed AGCAGCA)	-0.662	0.00000193	Experimentally Observed
hsa-miR-106b-5p	miR-17-5p (and other miRNAs w/seed AAAGUGC)	-0.62	0.00000132	High (predicted)
hsa-miR-190a-5p	miR-190a-5p (and other miRNAs w/seed GAUAUGU)	-0.573	0.00156	High (predicted)
hsa-miR-192-3p	miR-192-3p (miRNAs w/seed UGCCAAU)	-0.503	0.00468	High (predicted)
hsa-miR-193b-3p	miR-193a-3p (and other miRNAs w/seed ACUGGCC)	-0.537	0.000864	High (predicted)
hsa-miR-199b-5p	miR-199a-5p (and other miRNAs w/seed CCAGUGU)	-0.588	0.000000031	High (predicted)
hsa-miR-204-3p	miR-204-3p (and other miRNAs w/seed CUGGGAA)	0.627	0.0244	High (predicted)
hsa-miR-204-5p	miR-204-5p (and other miRNAs w/seed UCCCUUU)	0.7	0.00191	High (predicted)
hsa-miR-21-3p	miR-21-3p (and other miRNAs w/seed	-0.735	0.000000918	High (predicted)

	AACACCA)			
hsa-miR-29c-3p	miR-29b-3p (and other miRNAs w/seed AGCACCA)	-0.527	0.0000457	High (predicted)
hsa-miR-30b-5p	miR-30c-5p (and other miRNAs w/seed GUAAACA)	-0.738	0.00000012	High (predicted)
hsa-miR-31-3p	miR-31-3p (and other miRNAs w/seed GCUAUGC)	-0.726	0.0000824	High (predicted)
hsa-miR-324-5p	miR-324-5p (miRNAs w/seed GCAUCCC)	-0.523	0.0000763	High (predicted)
hsa-miR-34c-5p	miR-34a-5p (and other miRNAs w/seed GGCAGUG)	-0.539	0.00000758	High (predicted)
hsa-miR-3614-5p	miR-3614-5p (miRNAs w/seed CACUUGG)	0.689	0.0000601	High (predicted)
hsa-miR-369-3p	miR-369-3p (miRNAs w/seed AUAAUAC)	-0.556	0.0000032	High (predicted)
hsa-miR-374b-3p	miR-374b-3p (miRNAs w/seed UUAGCAG)	-0.529	0.0000026	High (predicted)
hsa-miR-378e	miR-378a-3p (and other miRNAs w/seed CUGGACU)	-0.599	0.00166	High (predicted)
hsa-miR-451a	miR-451a (and other miRNAs w/seed AACCGUU)	-0.619	0.00298	High (predicted)
hsa-miR-582-5p	miR-582-5p (miRNAs w/seed UACAGUU)	-0.728	0.00000792	High (predicted)
hsa-miR-671-3p	miR-671-3p (and other miRNAs w/seed CCGGUUC)	0.515	0.0000175	High (predicted)
hsa-miR-7974	miR-7974 (miRNAs w/seed GGCUGUG)	0.708	0.00104	High (predicted)

Supplementary Table 7. Anti-correlated miRNA-mRNA pairs

miRNA	Log_2FC miRNA	Target mRNA	Log2FC mRNA	Source	Confidence
hsa-miR-101-3p	-0.689	CHAC2	0.268	TargetScan Human	High (predicted)
hsa-miR-101-3p	-0.689	CYB561D2	0.251	TargetScan Human	High (predicted)
hsa-miR-101-3p	-0.689	EED	0.147	TargetScan Human	High (predicted)
hsa-miR-101-3p	-0.689	FLRT3	0.352	TargetScan Human	High (predicted)
hsa-miR-101-3p	-0.689	HSPE1-MOB4	0.254	TargetScan Human	High (predicted)
hsa-miR-101-3p	-0.689	MOB4	0.184	TargetScan Human	High (predicted)
hsa-miR-101-3p	-0.689	NACA	0.242	TargetScan Human	High (predicted)
hsa-miR-101-3p	-0.689	NDUFB5	0.25	TargetScan Human	High (predicted)
hsa-miR-101-3p	-0.689	POMP	0.359	TargetScan Human	High (predicted)
hsa-miR-101-3p	-0.689	RRM1	0.245	TargetScan Human	High (predicted)
hsa-miR-21-3p	-0.735	SNRPB2	0.24	TargetScan Human	High (predicted)
hsa-miR-127-5p	-0.651	CCT6A	0.244	TargetScan Human	High (predicted)
hsa-miR-127-5p	-0.651	KDELR2	0.196	TargetScan Human	High (predicted)
hsa-miR-127-5p	-0.651	MCRIP1	0.273	TargetScan Human	High (predicted)

hsa-miR-127-5p	-0.651	PEF1	0.284	TargetScan Human	High (predicted)
hsa-miR-127-5p	-0.651	PIGY	0.373	TargetScan Human	High (predicted)
hsa-miR-127-5p	-0.651	PYURF	0.309	TargetScan Human	High (predicted)
hsa-miR-127-5p	-0.651	YY1AP1	0.24	TargetScan Human	High (predicted)
hsa-miR-1296-5p	-0.537	ENOX2	0.158	TargetScan Human	High (predicted)
hsa-miR-1296-5p	-0.537	KANSL2	0.168	TargetScan Human	High (predicted)
hsa-miR-1296-5p	-0.537	TMEM107	0.85	TargetScan Human	High (predicted)
hsa-miR-1307-5p	-0.555	TEX30	0.225	TargetScan Human	High (predicted)
hsa-miR-130a-3p	-0.521	CCT6A	0.244	TargetScan Human	High (predicted)
hsa-miR-130a-3p	-0.521	CHMP3	0.14	TargetScan Human	High (predicted)
hsa-miR-130a-3p	-0.521	G0S2	0.833	TargetScan Human	High (predicted)
hsa-miR-130a-3p	-0.521	G3BP2	0.215	TargetScan Human	High (predicted)
hsa-miR-130a-3p	-0.521	MTMR6	0.208	TargetScan Human	High (predicted)
hsa-miR-130a-3p	-0.521	PDZD11	0.243	TargetScan Human	High (predicted)
hsa-miR-130a-3p	-0.521	RAB30	0.598	TargetScan Human	High (predicted)
hsa-	-0.521	WDR47	0.202	TargetScan	High (predicted)

miR-130a-3p				Human	
hsa-miR-136-3p	-0.561	COMMD2	0.202	TargetScan Human	High (predicted)
hsa-miR-136-3p	-0.561	FABP2	0.528	TargetScan Human	High (predicted)
hsa-miR-136-3p	-0.561	TRIAP1	0.263	TargetScan Human	High (predicted)
hsa-miR-138-5p	-0.55	ARL6IP5	0.248	TargetScan Human	High (predicted)
hsa-miR-138-5p	-0.55	CLN5	0.201	TargetScan Human	High (predicted)
hsa-miR-138-5p	-0.55	CTSH	0.312	TargetScan Human	High (predicted)
hsa-miR-138-5p	-0.55	EIF4EBP1	0.464	TargetScan Human	High (predicted)
hsa-miR-138-5p	-0.55	GPD1	0.413	TargetScan Human	High (predicted)
hsa-miR-138-5p	-0.55	H3F3A/H3F3B	0.282	TargetScan Human	High (predicted)
hsa-miR-138-5p	-0.55	KBTBD4	0.247	TargetScan Human	High (predicted)
hsa-miR-138-5p	-0.55	KDELR2	0.196	TargetScan Human	High (predicted)
hsa-miR-138-5p	-0.55	LSM1	0.295	TargetScan Human	High (predicted)
hsa-miR-138-5p	-0.55	MCRIP1	0.273	TargetScan Human	High (predicted)
hsa-miR-138-5p	-0.55	MIF4GD	0.201	TargetScan Human	High (predicted)
hsa-miR-	-0.55	MRM3	0.201	TargetScan Human	High (predicted)

138-5p					
hsa-miR-138-5p	-0.55	PACSIN2	0.263	TargetScan Human	High (predicted)
hsa-miR-138-5p	-0.55	PHB	0.264	TargetScan Human	High (predicted)
hsa-miR-138-5p	-0.55	RHBDD1	0.122	TargetScan Human	High (predicted)
hsa-miR-138-5p	-0.55	RIMBP3 (includes others)	0.655	TargetScan Human	High (predicted)
hsa-miR-138-5p	-0.55	RPS15A	0.33	TargetScan Human	High (predicted)
hsa-miR-138-5p	-0.55	RSPH10B/RSPH10B2	0.354	TargetScan Human	High (predicted),Moderate (predicted)
hsa-miR-138-5p	-0.55	ST6GALNAC4	0.419	TargetScan Human	High (predicted)
hsa-miR-138-5p	-0.55	TMEM199	0.24	TargetScan Human	High (predicted)
hsa-miR-15a-5p	-0.662	ADSS	0.182	TarBase	Experimentally Observed
hsa-miR-15a-5p	-0.662	ANAPC16	0.223	miRecords	Experimentally Observed
hsa-miR-15a-5p	-0.662	ARL3	0.294	TargetScan Human	High (predicted)
hsa-miR-15a-5p	-0.662	CHAC1	1.284	TargetScan Human	High (predicted)
hsa-miR-15a-5p	-0.662	DTD1	0.218	TarBase	Experimentally Observed
hsa-miR-15a-5p	-0.662	EMC4	0.255	TargetScan Human	High (predicted)
hsa-miR-15a-5p	-0.662	G0S2	0.833	TargetScan Human	High (predicted)

hsa-miR-15a-5p	-0.662	GFM1	0.235	TarBase	Experimentally Observed
hsa-miR-15a-5p	-0.662	H3F3A/H3F3B	0.282	miRecords	Experimentally Observed
hsa-miR-15a-5p	-0.662	HSPA4L	0.359	TargetScan Human	High (predicted)
hsa-miR-15a-5p	-0.662	HSPE1-MOB4	0.254	TargetScan Human	High (predicted)
hsa-miR-15a-5p	-0.662	LAMTOR5	0.288	TarBase	Experimentally Observed
hsa-miR-15a-5p	-0.662	LMAN2L	0.221	TargetScan Human	High (predicted)
hsa-miR-15a-5p	-0.662	MOB4	0.184	TargetScan Human	High (predicted)
hsa-miR-15a-5p	-0.662	MRPL20	0.256	TarBase	Experimentally Observed
hsa-miR-15a-5p	-0.662	OMA1	0.221	miRecords	Experimentally Observed
hsa-miR-15a-5p	-0.662	PCMT1	0.238	TargetScan Human	High (predicted)
hsa-miR-15a-5p	-0.662	PEX12	0.165	TargetScan Human	High (predicted)
hsa-miR-15a-5p	-0.662	PSAT1	0.965	TarBase,TargetScan Human	Experimentally Observed,Moderate (predicted)
hsa-miR-15a-5p	-0.662	PSMD7	0.223	TargetScan Human	High (predicted)
hsa-miR-15a-5p	-0.662	RAB30	0.598	TarBase,TargetScan Human	Experimentally Observed,High (predicted)
hsa-miR-15a-5p	-0.662	RARS	0.252	TarBase	Experimentally Observed
hsa-	-0.662	RFT1	0.182	TarBase	Experimentally

miR-15a-5p					Observed
hsa-miR-15a-5p	-0.662	RPL14	0.24	TargetScan Human	High (predicted)
hsa-miR-15a-5p	-0.662	SCOC	0.453	TargetScan Human	High (predicted)
hsa-miR-15a-5p	-0.662	SPTLC1	0.173	TarBase,TargetScan Human	Experimentally Observed,Moderate (predicted)
hsa-miR-15a-5p	-0.662	SRPRB	0.373	TarBase	Experimentally Observed
hsa-miR-15a-5p	-0.662	TMEM183A	0.149	TargetScan Human	High (predicted)
hsa-miR-15a-5p	-0.662	TMEM251	0.204	miRecords	Experimentally Observed
hsa-miR-15a-5p	-0.662	TMEM258	0.415	TargetScan Human	High (predicted)
hsa-miR-15a-5p	-0.662	TPI1	0.241	miRecords	Experimentally Observed
hsa-miR-15a-5p	-0.662	UGP2	0.213	miRecords	Experimentally Observed
hsa-miR-15a-5p	-0.662	WDR83OS	0.318	TargetScan Human	High (predicted)
hsa-miR-106b-5p	-0.62	CMTR2	0.176	TargetScan Human	High (predicted)
hsa-miR-106b-5p	-0.62	COMMD6	0.318	TargetScan Human	High (predicted)
hsa-miR-106b-5p	-0.62	CROT	0.302	TargetScan Human	High (predicted)
hsa-miR-106b-5p	-0.62	DERL2	0.19	TargetScan Human	High (predicted)
hsa-miR-	-0.62	FBXL5	0.227	TargetScan Human	High (predicted)

106b-5p					
hsa-miR-106b-5p	-0.62	43528	0.434	TargetScan Human	High (predicted)
hsa-miR-106b-5p	-0.62	RPS15A	0.33	TargetScan Human	High (predicted)
hsa-miR-106b-5p	-0.62	TIMM17A	0.384	TargetScan Human	High (predicted)
hsa-miR-190a-5p	-0.573	ARPC5	0.25	TargetScan Human	High (predicted)
hsa-miR-190a-5p	-0.573	GYG1	0.28	TargetScan Human	High (predicted)
hsa-miR-190a-5p	-0.573	TOMM5	0.363	TargetScan Human	High (predicted)
hsa-miR-192-3p	-0.503	ALG8	0.28	TargetScan Human	High (predicted)
hsa-miR-192-3p	-0.503	MS4A4A	0.472	TargetScan Human	High (predicted)
hsa-miR-192-3p	-0.503	RANGRF	0.229	TargetScan Human	High (predicted)
hsa-miR-192-3p	-0.503	TMED2	0.236	TargetScan Human	High (predicted)
hsa-miR-192-3p	-0.503	TMEM187	0.355	TargetScan Human	High (predicted)
hsa-miR-192-3p	-0.503	ZNF627	0.203	TargetScan Human	High (predicted)
hsa-miR-193b-3p	-0.537	AIMP2	0.289	TargetScan Human	High (predicted)
hsa-miR-193b-3p	-0.537	ARFIP1	0.168	TargetScan Human	High (predicted)
hsa-miR-193b-3p	-0.537	ARPC5	0.25	TargetScan Human	High (predicted)

hsa-miR-193b-3p	-0.537	COX7C	0.296	TargetScan Human	High (predicted)
hsa-miR-193b-3p	-0.537	HIST1H4I	0.322	TargetScan Human	High (predicted)
hsa-miR-193b-3p	-0.537	LYRM2	0.208	TargetScan Human	High (predicted)
hsa-miR-193b-3p	-0.537	NME1	0.491	TargetScan Human	High (predicted)
hsa-miR-193b-3p	-0.537	NSF	0.238	TargetScan Human	High (predicted)
hsa-miR-193b-3p	-0.537	RNF146	0.156	TargetScan Human	High (predicted)
hsa-miR-193b-3p	-0.537	RPS21	0.38	TargetScan Human	High (predicted)
hsa-miR-193b-3p	-0.537	RTN4IP1	0.345	TargetScan Human	High (predicted)
hsa-miR-193b-3p	-0.537	TWISTNB	0.212	TargetScan Human	High (predicted)
hsa-miR-193b-3p	-0.537	ZBTB6	0.232	TargetScan Human	High (predicted)
hsa-miR-199b-5p	-0.588	FABP2	0.528	TargetScan Human	High (predicted)
hsa-miR-199b-5p	-0.588	FLRT3	0.352	TargetScan Human	High (predicted)
hsa-miR-199b-5p	-0.588	JKAMP	0.238	TargetScan Human	High (predicted)
hsa-miR-199b-5p	-0.588	MAGT1	0.204	TargetScan Human	High (predicted)
hsa-miR-199b-5p	-0.588	NUDT5	0.217	TargetScan Human	High (predicted)
hsa-	-0.588	SCGN	0.527	TargetScan	High (predicted)

miR-199b-5p				Human	
hsa-miR-199b-5p	-0.588	TSPAN6	0.392	TargetScan Human	High (predicted)
hsa-miR-199b-5p	-0.588	TXNDC12	0.208	TargetScan Human	High (predicted)
hsa-miR-199b-5p	-0.588	ZNF627	0.203	TargetScan Human	High (predicted)
hsa-miR-204-3p	0.627	GPATCH8	-0.181	TargetScan Human	High (predicted)
hsa-miR-204-3p	0.627	ICOSLG/ LOC102723996	-0.298	TargetScan Human	High (predicted)
hsa-miR-204-3p	0.627	LRRN4	-0.437	TargetScan Human	High (predicted)
hsa-miR-204-3p	0.627	RBM33	-0.148	TargetScan Human	High (predicted)
hsa-miR-204-3p	0.627	SLX4	-0.279	TargetScan Human	High (predicted)
hsa-miR-204-3p	0.627	SYNJ2	-0.184	TargetScan Human	High (predicted)
hsa-miR-204-5p	0.7	CASTOR3	-0.263	TargetScan Human	High (predicted)
hsa-miR-204-5p	0.7	ITGB4	-0.383	miRecords	Experimentally Observed
hsa-miR-204-5p	0.7	TLX2	-0.497	TargetScan Human	High (predicted)
hsa-miR-204-5p	0.7	TTYH1	-0.336	TargetScan Human	High (predicted)
hsa-miR-29c-3p	-0.527	AIMP2	0.289	TargetScan Human	High (predicted)
hsa-miR-	-0.527	COMMD2	0.202	TargetScan Human	High (predicted)

29c-3p					
hsa-miR-29c-3p	-0.527	CREG1	0.219	TargetScan Human	High (predicted)
hsa-miR-29c-3p	-0.527	CYCS	0.41	TargetScan Human	High (predicted)
hsa-miR-29c-3p	-0.527	GEMIN2	0.246	TargetScan Human	High (predicted)
hsa-miR-29c-3p	-0.527	GMFB	0.221	Ingenuity Expert Findings	Experimentally Observed
hsa-miR-29c-3p	-0.527	IFI30	0.394	TargetScan Human	High (predicted)
hsa-miR-29c-3p	-0.527	MFAP3	0.151	TargetScan Human	High (predicted)
hsa-miR-29c-3p	-0.527	NIPSNAP3A	0.185	TargetScan Human	High (predicted)
hsa-miR-29c-3p	-0.527	NPM3	0.364	TargetScan Human	High (predicted)
hsa-miR-29c-3p	-0.527	OSTC	0.362	TargetScan Human	High (predicted)
hsa-miR-29c-3p	-0.527	RAB30	0.598	TargetScan Human	High (predicted)
hsa-miR-29c-3p	-0.527	SLC16A14	0.545	TargetScan Human	High (predicted)
hsa-miR-29c-3p	-0.527	TDG	0.175	TarBase,TargetScan Human,miRecords	Experimentally Observed,High (predicted)
hsa-miR-29c-3p	-0.527	TIMM8B	0.388	TargetScan Human	High (predicted)
hsa-miR-29c-3p	-0.527	TMEM141	0.34	TargetScan Human	High (predicted)
hsa-miR-29c-3p	-0.527	TMEM183A	0.149	TargetScan Human	High (predicted)

hsa-miR-29c-3p	-0.527	UQCR11	0.347	TargetScan Human	High (predicted)
hsa-miR-30b-5p	-0.738	ANKRA2	0.208	TargetScan Human	High (predicted)
hsa-miR-30b-5p	-0.738	ARL4A	0.508	TargetScan Human	High (predicted)
hsa-miR-30b-5p	-0.738	CFDP1	0.228	TargetScan Human	High (predicted)
hsa-miR-30b-5p	-0.738	EED	0.147	TargetScan Human	High (predicted)
hsa-miR-30b-5p	-0.738	IDH1	0.257	TarBase,TargetScan Human	Experimentally Observed,High (predicted)
hsa-miR-30b-5p	-0.738	MPDU1	0.289	TarBase	Experimentally Observed
hsa-miR-30b-5p	-0.738	NCEH1	0.294	TarBase,TargetScan Human	Experimentally Observed,Moderate (predicted)
hsa-miR-30b-5p	-0.738	NECAP1	0.188	TargetScan Human	High (predicted)
hsa-miR-30b-5p	-0.738	SRSF7	0.319	TargetScan Human	High (predicted)
hsa-miR-30b-5p	-0.738	STX1A	0.484	miRecords	Experimentally Observed
hsa-miR-30b-5p	-0.738	SYPL1	0.174	TarBase	Experimentally Observed
hsa-miR-30b-5p	-0.738	TMED10	0.181	TarBase,TargetScan Human	Experimentally Observed,Moderate (predicted)
hsa-miR-30b-5p	-0.738	TMED2	0.236	TarBase,TargetScan Human	Experimentally Observed,Moderate (predicted)
hsa-miR-30b-5p	-0.738	TMEM59	0.216	TarBase	Experimentally Observed
hsa-	-0.738	TMEM87A	0.189	TarBase,TargetSca	Experimentally

miR-30b-5p				n Human	Observed,Moderate (predicted)
hsa-miR-30b-5p	-0.738	UAP1	0.182	TarBase	Experimentally Observed
hsa-miR-31-3p	-0.726	C19orf12	0.301	TargetScan Human	High (predicted)
hsa-miR-31-3p	-0.726	DIRAS3	0.491	TargetScan Human	High (predicted)
hsa-miR-31-3p	-0.726	ELOC	0.228	TargetScan Human	High (predicted)
hsa-miR-31-3p	-0.726	MRPS16	0.227	TargetScan Human	High (predicted)
hsa-miR-31-3p	-0.726	MTPN	0.163	TargetScan Human	High (predicted)
hsa-miR-31-3p	-0.726	RBM8A	0.175	TargetScan Human	High (predicted)
hsa-miR-31-3p	-0.726	TMEM107	0.85	TargetScan Human	High (predicted)
hsa-miR-324-5p	-0.523	ACTRT3	0.423	TargetScan Human	High (predicted)
hsa-miR-324-5p	-0.523	IL17D	0.396	TargetScan Human	High (predicted)
hsa-miR-324-5p	-0.523	PANK1	0.323	TargetScan Human	High (predicted)
hsa-miR-324-5p	-0.523	POLR2C	0.205	TargetScan Human	High (predicted)
hsa-miR-324-5p	-0.523	RPA2	0.256	TargetScan Human	High (predicted)
hsa-miR-324-5p	-0.523	SRP9	0.23	TargetScan Human	High (predicted)
hsa-miR-	-0.523	TMEM135	0.322	TargetScan Human	High (predicted)

324-5p					
hsa-miR-324-5p	-0.523	TMEM141	0.34	TargetScan Human	High (predicted)
hsa-miR-34c-5p	-0.539	C12orf57	0.391	TargetScan Human	High (predicted)
hsa-miR-34c-5p	-0.539	COMMD9	0.178	TargetScan Human	High (predicted)
hsa-miR-34c-5p	-0.539	GCH1	0.433	TargetScan Human	High (predicted)
hsa-miR-34c-5p	-0.539	GLRX5	0.417	TargetScan Human	High (predicted)
hsa-miR-34c-5p	-0.539	HIST1H4H	0.45	TargetScan Human	High (predicted)
hsa-miR-34c-5p	-0.539	HNRNPA1	0.19	TargetScan Human	High (predicted)
hsa-miR-34c-5p	-0.539	KCNK3	0.318	TargetScan Human	High (predicted)
hsa-miR-34c-5p	-0.539	NCEH1	0.294	TargetScan Human	High (predicted)
hsa-miR-34c-5p	-0.539	PPP1R14D	0.397	TargetScan Human	High (predicted)
hsa-miR-34c-5p	-0.539	RAP1GDS1	0.214	TargetScan Human	High (predicted)
hsa-miR-34c-5p	-0.539	RDH11	0.4	TargetScan Human	High (predicted)
hsa-miR-34c-5p	-0.539	RPL36A-HNRNPH2	0.269	TargetScan Human	High (predicted)
hsa-miR-34c-5p	-0.539	SMIM30	0.25	TargetScan Human	High (predicted)
hsa-miR-34c-5p	-0.539	STX1A	0.484	TargetScan Human	High (predicted)

hsa-miR-34c-5p	-0.539	TMEM126B	0.301	TargetScan Human	High (predicted)
hsa-miR-34c-5p	-0.539	TMEM141	0.34	TargetScan Human	High (predicted)
hsa-miR-34c-5p	-0.539	TSN	0.202	TargetScan Human	High (predicted)
hsa-miR-34c-5p	-0.539	XBP1	0.354	TargetScan Human	High (predicted)
hsa-miR-34c-5p	-0.539	ZCCHC17	0.199	TargetScan Human	High (predicted)
hsa-miR-369-3p	-0.556	GPN3	0.262	TargetScan Human	High (predicted)
hsa-miR-374b-3p	-0.529	EMC4	0.255	TargetScan Human	High (predicted)
hsa-miR-374b-3p	-0.529	HSPE1-MOB4	0.254	TargetScan Human	High (predicted)
hsa-miR-374b-3p	-0.529	LCN2	1.276	TargetScan Human	High (predicted)
hsa-miR-374b-3p	-0.529	MOB4	0.184	TargetScan Human	High (predicted)
hsa-miR-374b-3p	-0.529	TMEM70	0.377	TargetScan Human	High (predicted)
hsa-miR-374b-3p	-0.529	TSG101	0.191	TargetScan Human	High (predicted)
hsa-miR-378e	-0.599	GOLT1A	0.477	TargetScan Human	High (predicted)
hsa-miR-378e	-0.599	GPN1	0.184	TargetScan Human	High (predicted)
hsa-miR-378e	-0.599	GZMB	0.692	TargetScan Human	High (predicted)
hsa-	-0.599	HNRNPA1	0.19	TargetScan	High (predicted)

miR-378e				Human	
hsa-miR-378e	-0.599	KBTBD7	0.209	TargetScan Human	High (predicted)
hsa-miR-378e	-0.599	MROH8	0.268	TargetScan Human	High (predicted)
hsa-miR-378e	-0.599	PSMA1	0.217	TargetScan Human	High (predicted)
hsa-miR-378e	-0.599	PTGES3	0.187	TargetScan Human	High (predicted)
hsa-miR-378e	-0.599	RBX1	0.288	TargetScan Human	High (predicted)
hsa-miR-378e	-0.599	RPN2	0.251	TargetScan Human	High (predicted)
hsa-miR-378e	-0.599	TRIAP1	0.263	TargetScan Human	High (predicted)
hsa-miR-451a	-0.619	ATP5F1E	0.324	TargetScan Human	High (predicted)
hsa-miR-451a	-0.619	PSMB8	0.357	TargetScan Human	High (predicted)
hsa-miR-582-5p	-0.728	CXCL2	0.708	TargetScan Human	High (predicted)
hsa-miR-582-5p	-0.728	HNRNPA1	0.19	TargetScan Human	High (predicted)
hsa-miR-582-5p	-0.728	PBK	0.419	TargetScan Human	High (predicted)
hsa-miR-582-5p	-0.728	PPIH	0.214	TargetScan Human	High (predicted)
hsa-miR-582-5p	-0.728	TIMM23	0.217	TargetScan Human	High (predicted)
hsa-miR-	-0.728	UQCRH	0.337	TargetScan Human	High (predicted)

582-5p					
hsa-miR-582-5p	-0.728	ZNF627	0.203	TargetScan Human	High (predicted)
hsa-miR-671-3p	0.515	FOXP2	-0.323	TargetScan Human	High (predicted)
hsa-miR-671-3p	0.515	IGF2BP2-AS1	-0.414	TargetScan Human	High (predicted)
hsa-miR-671-3p	0.515	PRRC2B	-0.161	TargetScan Human	High (predicted)
hsa-miR-671-3p	0.515	SLC7A8	-0.256	TargetScan Human	High (predicted)
hsa-miR-671-3p	0.515	VMAC	-0.318	TargetScan Human	High (predicted)
hsa-miR-7974	0.708	ASB14	-0.304	TargetScan Human	High (predicted)
hsa-miR-7974	0.708	ATXN2	-0.176	TargetScan Human	High (predicted)
hsa-miR-7974	0.708	C11orf49	-0.173	TargetScan Human	High (predicted)
hsa-miR-7974	0.708	CYP4F3	-0.472	TargetScan Human	High (predicted)
hsa-miR-7974	0.708	FAM222B	-0.222	TargetScan Human	High (predicted)
hsa-miR-7974	0.708	IGFBP4	-0.427	TargetScan Human	High (predicted)
hsa-miR-7974	0.708	KIAA1211L	-0.226	TargetScan Human	High (predicted)
hsa-miR-7974	0.708	MT-ND4L	-1.086	TargetScan Human	High (predicted)
hsa-miR-7974	0.708	PARD3B	-0.143	TargetScan Human	High (predicted)

hsa-miR-7974	0.708	PLEC	-0.456	TargetScan Human	High (predicted)
hsa-miR-7974	0.708	PPP5D1	-0.366	TargetScan Human	High (predicted)
hsa-miR-7974	0.708	SEMA4G	-0.344	TargetScan Human	High (predicted)
hsa-miR-7974	0.708	SOX13	-0.283	TargetScan Human	High (predicted)

Abbreviations: FC, fold change

Supplementary Table 8. AME analysis – 118 motifs enriched in promoters of differentially expressed genes

Rank	motif_ID	motif_alt_ID	consensus	p-value	adj_p-value	E-value	tests	FASTA max	pos	neg	PWM min	TP	%TP	FP	%FP
1	MA0516.1	SP2	GYCCCGCCYCYBSSS	1.63E-67	3.12E-64	5.65E-61	1915	3139	3139	3139	3.35	976	31.09	412	13.13
2	MA0079.3	SP1	GCCCCKCCCCC	1.92E-66	3.86E-63	6.97E-60	2005	3139	3139	3139	6.92	862	27.46	334	10.64
3	MA0599.1	KLF5	GCCCCDCCCH	1.53E-49	2.80E-46	5.06E-43	1832	3139	3139	3139	1.38	1113	35.46	595	18.96
4	MA0746.1	SP3	VCCACGCCCMC	2.04E-41	4.14E-38	7.48E-35	2026	3139	3139	3139	3.24	689	21.95	304	9.68
5	MA0528.1	ZNF263	GGAGGAGGRRGRGGRGGRRGR	1.17E-34	1.58E-31	2.86E-28	1351	3139	3139	3139	2.98	691	22.01	335	10.67
6	MA0136.2	ELF5	AMSMGGAAGTD	1.20E-33	1.82E-30	3.29E-27	1516	3139	3139	3139	2.1	678	21.6	330	10.51
7	MA0076.2	ELK4	BCRCTTCCGGB	1.04E-32	1.08E-29	1.96E-26	1038	3139	3139	3139	11.8	274	8.73	67	2.13
8	MA0685.1	SP4	BWRGCCACGCCCMCTYH	3.67E-31	1.10E-28	1.98E-25	299	3139	3139	3139	1.24	244	7.77	55	1.75
9	MA0095.2	YY1	CAARATGGCBGC	6.58E-31	3.79E-28	6.85E-25	576	3139	3139	3139	3.6	302	9.62	87	2.77
10	MA0028.2	ELK1	ACCGGAAGTR	2.90E-30	1.36E-27	2.47E-24	471	3139	3139	3139	1.3	333	10.61	107	3.41
11	MA0741.1	KLF16	GMCACGCCCCC	1.75E-30	2.45E-27	4.43E-24	1398	3139	3139	3139	17	284	9.05	78	2.48
12	MA0062.2	Gabpa	CCGGAAGTGVV	8.92E-30	7.98E-27	1.44E-23	895	3139	3139	3139	9.95	268	8.54	71	2.26
13	MA0474.2	ERG	ACCGGAARTV	1.01E-29	8.66E-27	1.57E-23	854	3139	3139	3139	4.81	275	8.76	75	2.39
14	MA0475.2	FLI1	ACCGGAARTR	2.41E-29	1.50E-26	2.72E-23	624	3139	3139	3139	2.4	320	10.19	102	3.25
15	MA0759.1	ELK3	ACCGGAAGTD	2.33E-29	1.89E-26	3.41E-23	810	3139	3139	3139	4.99	255	8.12	65	2.07
16	MA0098.3	ETS1	ACCGGAARTR	2.38E-29	2.15E-26	3.89E-23	902	3139	3139	3139	3.24	348	11.09	119	3.79
17	MA0764.1	ETV4	ACCGGAAGTR	3.74E-29	3.92E-26	7.09E-23	1049	3139	3139	3139	3.89	314	10	99	3.15
18	MA0765.1	ETV5	ACCGGAWGYN	3.79E-29	4.54E-26	8.20E-23	1198	3139	3139	3139	5.76	237	7.55	56	1.78
19	MA0750.2	ZBTB7A	NVCCGGAAGTGSV	6.88E-29	8.91E-26	1.61E-22	1295	3139	3139	3139	6.23	326	10.39	107	3.41
20	MA0761.1	ETV1	ACCGGAAGTD	1.47E-28	1.42E-25	2.56E-22	963	3139	3139	3139	3.57	321	10.23	105	3.35
21	MA0760.1	ERF	ACCGGAAGTR	3.17E-28	1.99E-25	3.59E-22	627	3139	3139	3139	3.09	282	8.98	83	2.64
22	MA0156.2	FEV	ACCGGAAGTN	7.18E-28	6.10E-25	1.10E-21	849	3139	3139	3139	3	337	10.74	117	3.73

23	MA0641.1	ELF4	AACCCGGAAGTR	1.38E-26	4.76E-24	8.61E-21	346	3139	3139	3139	1.13	267	8.51	79	2.52
24	MA0645.1	ETV6	MSCGGAAGTR	8.31E-27	8.33E-24	1.51E-20	1002	3139	3139	3139	3.69	372	11.85	143	4.56
25	MA1125.1	ZNF384	DNWMAAAAAAAA	1.20E-26	1.13E-23	2.04E-20	938	3139	3139	3139	1810	123	3.92	10	0.32
26	MA0763.1	ETV3	ACCGGAAGTR	1.67E-26	1.26E-23	2.27E-20	753	3139	3139	3139	6.13	225	7.17	56	1.78
27	MA0740.1	KLF14	KRCCACGCCCMCYT	4.52E-26	2.47E-23	4.46E-20	546	3139	3139	3139	1.15	383	12.2	153	4.87
28	MA0598.2	EHF	WACCCGGAAGTA	1.46E-25	7.34E-23	1.33E-19	502	3139	3139	3139	1.21	345	10.99	130	4.14
29	MA0473.2	ELF1	AACCCGGAAGTR	2.10E-25	7.81E-23	1.41E-19	371	3139	3139	3139	1.29	268	8.54	83	2.64
30	MA0747.1	SP8	RCCACGCCCMCY	6.82E-26	1.02E-22	1.84E-19	1493	3139	3139	3139	1.79	721	22.97	404	12.87
31	MA0506.1	NRF1	GCGCVTGCGCR	3.52E-25	3.23E-22	5.83E-19	917	3139	3139	3139	75.7	134	4.27	16	0.51
32	MA0640.1	ELF3	WACCCGGAAGTAR	1.78E-23	4.52E-21	8.17E-18	254	3139	3139	3139	1.26	194	6.18	47	1.5
33	MA0041.1	Foxd3	GWWTGTTTDTTT	3.07E-23	3.13E-20	5.66E-17	1021	3139	3139	3139	7.6	397	12.65	174	5.54
34	MA0050.2	IRF1	NTTYASTTTCACTTTCDBTTT	2.23E-20	1.30E-17	2.34E-14	580	3139	3139	3139	4.52	303	9.65	122	3.89
35	MA1107.1	KLF9	VSCCACACCCACH	2.02E-20	1.84E-17	3.32E-14	908	3139	3139	3139	1.21	547	17.43	299	9.53
36	MA0748.1	YY2	RWCCGCCATTW	2.25E-19	1.42E-16	2.57E-13	632	3139	3139	3139	2.54	254	8.09	94	2.99
37	MA0018.3	CREB1	NVTGACGTCABN	1.46E-18	7.12E-16	1.29E-12	488	3139	3139	3139	1.66	271	8.63	108	3.44
38	MA0493.1	Klf1	RRCCACACCCW	1.01E-18	9.26E-16	1.67E-12	920	3139	3139	3139	1.07	577	18.38	333	10.61
39	MA0604.1	Atf1	RTGACGTA	5.58E-18	2.67E-15	4.83E-12	479	3139	3139	3139	3.97	168	5.35	47	1.5
40	MA1133.1	JUN::JUNB (var.2)	KRTGACGTCATN	7.57E-18	4.05E-15	7.33E-12	535	3139	3139	3139	5.88	125	3.98	25	0.8
41	MA0497.1	MEF2C	DDDCYAAAAATAGMW	1.45E-17	1.24E-14	2.25E-11	859	3139	3139	3139	6.81	315	10.04	142	4.52
42	MA0686.1	SPDEF	AMCCGGATGTR	5.35E-17	1.59E-14	2.87E-11	297	3139	3139	3139	1.12	212	6.75	76	2.42
43	MA0834.1	ATF7	NDATGACGTCATMV	3.23E-16	2.84E-14	5.13E-11	88	3139	3139	3139	1.22	76	2.42	7	0.22
44	MA0502.1	NFYB	VMMYBRRCCAATCAG	6.87E-17	3.09E-14	5.59E-11	450	3139	3139	3139	4.51	213	6.79	77	2.45
45	MA0032.2	FOXC1	WAWGTAAAYAW	3.94E-17	4.30E-14	7.77E-11	1090	3139	3139	3139	1.08	669	21.31	419	13.35
46	MA0108.2	TBP	STATAAAWRSVVVBN	6.06E-17	6.80E-14	1.23E-10	1122	3139	3139	3139	2.19	497	15.83	281	8.95

47	MA0846.1	FOXC2	WAWGTAAACAWW	1.46E-16	1.75E-13	3.16E-10	1199	3139	3139	3139	1.81	601	19.15	367	11.69
48	MA1129.1	FOSL1::JUN (var.2)	ATGACGTCAT	6.18E-16	3.49E-13	6.31E-10	565	3139	3139	3139	1.07	373	11.88	192	6.12
49	MA1136.1	FOSB::JUNB (var.2)	RTGACGTCAT	8.99E-16	4.24E-13	7.67E-10	472	3139	3139	3139	1.76	233	7.42	94	2.99
50	MA1122.1	TFDP1	VSGCGGGAAVN	3.41E-16	4.70E-13	8.49E-10	1377	3139	3139	3139	1.3	701	22.33	453	14.43
51	MA0847.1	FOXD2	GTAAACA	5.48E-16	9.23E-13	1.67E-09	1685	3139	3139	3139	4.09	349	11.12	174	5.54
52	MA0762.1	ETV2	AACCGGAAATR	2.71E-15	1.11E-12	2.00E-09	408	3139	3139	3139	1.08	280	8.92	128	4.08
53	MA1145.1	FOSL2::JUND (var.2)	NNRTGACGTCAYHSN	4.36E-15	1.53E-12	2.76E-09	350	3139	3139	3139	4.53	132	4.21	35	1.12
54	MA1131.1	FOSL2::JUN (var.2)	GRTGACGTMAT	1.04E-14	3.84E-12	6.94E-09	368	3139	3139	3139	11	90	2.87	15	0.48
55	MA0850.1	FOXP3	RTAAACA	2.86E-15	4.63E-12	8.37E-09	1619	3139	3139	3139	5.03	264	8.41	117	3.73
56	MA1139.1	FOSL2::JUNB (var.2)	DATGACGTCATH	1.96E-14	5.73E-12	1.04E-08	293	3139	3139	3139	3.35	127	4.05	34	1.08
57	MA1140.1	JUNB(var.2)	RTGACGTCAY	9.34E-15	6.62E-12	1.20E-08	709	3139	3139	3139	1.07 E+00	441	14.05	251	8
58	MA1127.1	FOSB::JUN	GATGACGTCAT	1.73E-14	6.74E-12	1.22E-08	389	3139	3139	3139	5.82	114	3.63	27	0.86
59	MA0656.1	JDP2(var.2)	GATGACGTCAYC	1.03E-13	8.10E-12	1.47E-08	79	3139	3139	3139	2.35	61	1.94	5	0.16
60	MA0033.2	FOXL1	RTAAACA	1.45E-14	1.39E-11	2.51E-08	957	3139	3139	3139	9.1	268	8.54	123	3.92
61	MA0845.1	FOXB1	WATGTAAATAT	4.34E-14	3.88E-11	7.01E-08	894	3139	3139	3139	3.88	308	9.81	154	4.91
62	MA0593.1	FOXP2	RWGTAAACAVR	9.15E-14	4.76E-11	8.60E-08	520	3139	3139	3139	4.94	244	7.77	110	3.5
63	MA0840.1	Creb5	NATGACGTCAYH	6.91E-13	7.47E-11	1.35E-07	108	3139	3139	3139	9	55	1.75	4	0.13
64	MA0848.1	FOXO4	GTAAACA	8.01E-14	7.68E-11	1.39E-07	959	3139	3139	3139	3.52	321	10.23	165	5.26
65	MA0081.1	SPIB	WGMGGAA	5.78E-14	1.25E-10	2.25E-07	2156	3139	3139	3139	2	882	28.1	630	20.07
66	MA0899.1	HOXA10	DGYMATAAAAH	1.34E-13	2.09E-10	3.78E-07	1563	3139	3139	3139	2.29	643	20.48	425	13.54
67	MA0609.1	Crem	NATGACGTMA	9.88E-13	2.35E-10	4.25E-07	238	3139	3139	3139	13	75	2.39	12	0.38
68	MA0152.1	NFATC2	TTTTCCA	2.45E-13	4.74E-10	8.56E-07	1936	3139	3139	3139	1.04	1098	34.98	833	26.54
69	MA0851.1	Foxj3	NDAADGTAAACAAANNM	1.16E-12	8.35E-10	1.51E-06	720	3139	3139	3139	1.33	427	13.6	254	8.09
70	MA0638.1	CREB3	VTGCCACGTCAYCR	5.65E-12	8.59E-10	1.55E-06	152	3139	3139	3139	1.18	113	3.6	33	1.05

71	MA0852.2	FOXK1	NRDGTAAACAAGNN	9.42E-13	9.90E-10	1.79E-06	1051	3139	3139	3139	1.51	525	16.73	333	10.61
72	MA0073.1	RREB1	CCCCMAAMCAMCCMCMMMCV	2.99E-11	4.01E-09	7.24E-06	134	3139	3139	3139	1.4	104	3.31	30	0.96
73	MA1103.1	FOXK2	WDGTAAACANV	4.59E-12	4.84E-09	8.75E-06	1055	3139	3139	3139	3.27	326	10.39	179	5.7
74	MA0614.1	Foxj2	RTAAACAA	9.52E-12	8.05E-09	1.45E-05	845	3139	3139	3139	4.88	258	8.22	130	4.14
75	MA1143.1	FOSL1::JUND (var.2)	RTGACGTMAY	1.29E-11	1.35E-08	2.44E-05	1048	3139	3139	3139	2.94	208	6.63	95	3.03
76	MA0481.2	FOXP1	NDGTAAACAGNN	5.46E-11	5.39E-08	9.74E-05	986	3139	3139	3139	1.74	451	14.37	286	9.11
77	MA0517.1	STAT1::STAT2	THAGTTTCRKTTTCY	9.58E-11	5.94E-08	1.07E-04	620	3139	3139	3139	1.27	376	11.98	227	7.23
78	MA0605.1	Atf3	GATGACGT	1.06E-10	6.12E-08	1.11E-04	577	3139	3139	3139	1.45	316	10.07	180	5.73
79	MA0862.1	GMEB2	TTACGTAA	4.02E-10	1.26E-07	2.28E-04	314	3139	3139	3139	2.35	120	3.82	43	1.37
80	MA0471.1	E2F6	RGGCGGGARRV	1.46E-10	1.45E-07	2.62E-04	992	3139	3139	3139	17.1	212	6.75	103	3.28
81	MA0742.1	Klf12	GRCCACGCCCWHHWH	4.83E-10	1.56E-07	2.83E-04	324	3139	3139	3139	1.18	211	6.72	105	3.35
82	MA0060.3	NFYA	RRCCAATCAGM	2.28E-10	1.67E-07	3.03E-04	735	3139	3139	3139	11.3	142	4.52	56	1.78
83	MA0913.1	Hoxd9	GYAATWAAAW	1.15E-10	1.87E-07	3.39E-04	1632	3139	3139	3139	1.82	741	23.61	538	17.14
84	MA0910.1	Hoxd8	TAADTAATTAATRGCTN	2.73E-10	2.50E-07	4.51E-04	916	3139	3139	3139	1.42	524	16.69	353	11.25
85	MA0606.1	NFAT5	NTTTTCCAYN	1.45E-10	2.75E-07	4.98E-04	1900	3139	3139	3139	1.12	1032	32.88	804	25.61
86	MA0849.1	FOXO6	GTAAACA	4.75E-10	2.91E-07	5.25E-04	612	3139	3139	3139	1.9	282	8.98	158	5.03
87	MA1126.1	FOS::JUN(var.2)	DRTGACGTCATHNDTN	4.29E-10	2.96E-07	5.35E-04	689	3139	3139	3139	1.17	413	13.16	262	8.35
88	MA0042.2	FOXI1	GTAAACA	5.00E-10	3.21E-07	5.81E-04	643	3139	3139	3139	1.22	378	12.04	234	7.45
89	MA1116.1	RBPJ	BVTGGGAANN	4.83E-10	3.64E-07	6.58E-04	753	3139	3139	3139	12.1	211	6.72	105	3.35
90	MA0030.1	FOXF2	BNAASGTAAACAAD	1.45E-09	3.86E-07	6.98E-04	266	3139	3139	3139	1.46	171	5.45	79	2.52
91	MA0052.3	MEF2A	KCTAWAAATAGM	2.25E-09	4.70E-07	8.50E-04	209	3139	3139	3139	10.6	82	2.61	23	0.73
92	MA0687.1	SPIC	HAAAAGVGGAAGTA	7.19E-10	5.32E-07	9.62E-04	740	3139	3139	3139	1.25	435	13.86	282	8.98
93	MA0526.2	USF2	NNNGGTCACGTGRYYN	2.45E-09	5.49E-07	9.92E-04	224	3139	3139	3139	5.37	80	2.55	22	0.7
94	MA0829.1	Srebf1(var.2)	ATCACGTGAY	5.05E-09	8.44E-07	1.53E-03	167	3139	3139	3139	2.21	89	2.84	28	0.89

95	MA0047.2	Foxa2	TGTTTACWYWGB	1.57E-09	9.47E-07	1.71E-03	602	3139	3139	3139	1.3	346	11.02	212	6.75
96	MA0603.1	Arntl	NGTCACGTGH	2.60E-09	9.91E-07	1.79E-03	381	3139	3139	3139	16.2	54	1.72	9	0.29
97	MA0622.1	Mlxip	BCACGTGK	6.05E-09	1.16E-06	2.09E-03	191	3139	3139	3139	13	110	3.5	41	1.31
98	MA1100.1	ASCL1	VSAGCAGCTGSNN	1.38E-09	1.25E-06	2.25E-03	904	3139	3139	3139	1.05	535	17.04	369	11.76
99	MA0601.1	Arid3b	ATATTAATWAN	1.90E-09	2.20E-06	3.97E-03	1154	3139	3139	3139	3.13	470	14.97	315	10.04
100	MA0692.1	TFEB	RYCACGTGAC	4.76E-09	2.22E-06	4.02E-03	467	3139	3139	3139	36.6	38	1.21	3	0.1
101	MA0828.1	SREBF2(var.2)	ATCACGTGAY	1.74E-08	2.35E-06	4.24E-03	135	3139	3139	3139	2.12	81	2.58	25	0.8
102	MA0093.2	USF1	VYCAYGTGACC	7.04E-09	2.61E-06	4.72E-03	371	3139	3139	3139	92.2	27	0.86	0	0
103	MA0835.1	BATF3	TGATGACGTCATCA	2.30E-07	5.06E-06	9.14E-03	22	3139	3139	3139	1.61	22	0.7	0	0
104	MA0488.1	JUN	DDRATGATGTCAT	2.22E-08	5.70E-06	1.03E-02	257	3139	3139	3139	1.66	157	5	75	2.39
105	MA0901.1	HOXB13	CCAATAAAAH	6.75E-09	5.99E-06	1.08E-02	888	3139	3139	3139	1.18	498	15.86	344	10.96
106	MA0663.1	MLX	RTCACGTGAT	7.12E-08	8.69E-06	1.57E-02	122	3139	3139	3139	6.31	50	1.59	10	0.32
107	MA0146.2	Zfx	SSSGCCBVGGCCTS	8.12E-09	8.90E-06	1.61E-02	1096	3139	3139	3139	16.6	170	5.42	82	2.61
108	MA0620.2	MITF	NNDRGTCACGTGACYHNN	5.28E-08	1.08E-05	1.96E-02	205	3139	3139	3139	40.3	28	0.89	1	0.03
109	MA0139.1	CTCF	YGRCCASYAGRKGGCRSYR	3.08E-08	1.10E-05	1.99E-02	357	3139	3139	3139	77.9	35	1.12	3	0.1
110	MA0650.1	HOXA13	CYAATAAAAM	1.10E-08	1.13E-05	2.03E-02	1021	3139	3139	3139	1.21	566	18.03	405	12.9
111	MA0790.1	POU4F1	ATGMATAATTAATG	1.35E-08	1.13E-05	2.05E-02	839	3139	3139	3139	1.53	450	14.34	306	9.75
112	MA0148.3	FOXA1	YNBNTGTTTACWYWD	2.44E-08	1.23E-05	2.23E-02	506	3139	3139	3139	4.7	206	6.56	111	3.54
113	MA0664.1	MLXIPL	RTCACGTGAT	7.05E-08	1.63E-05	2.95E-02	231	3139	3139	3139	9.73	58	1.85	14	0.45
114	MA0617.1	Id2	RCACGTGR	9.05E-08	1.73E-05	3.13E-02	191	3139	3139	3139	9.1	124	3.95	55	1.75
115	MA0004.1	Arnt	CACGTG	4.90E-08	1.77E-05	3.21E-02	362	3139	3139	3139	4.81	136	4.33	62	1.98
116	MA0773.1	MEF2D	DCTAWAAATAGM	2.30E-07	2.30E-05	4.16E-02	100	3139	3139	3139	1.19	75	2.39	25	0.8
117	MA0831.2	TFE3	CACGTGAY	4.43E-08	2.31E-05	4.18E-02	521	3139	3139	3139	13.9	55	1.75	12	0.38
118	MA0791.1	POU4F3	RTGMATWATTAATGAR	3.10E-08	2.34E-05	4.23E-02	755	3139	3139	3139	2.06	371	11.82	243	7.74

Supplementary Table 9. FIMO motif scanning using JASPAR motif matrices using default p-value cutoff 1e-4 (GABPA)

GABPA motif	Alt ID	Sequence Name	Strand	Start	End	p-value	q-value	Matched Sequence
1	ACCGGAAGNS	NUDT5	-	4	13	7.46E-07	0.0772	ACCGGAAGAG
1	ACCGGAAGNS	RPL29P11	-	81	90	7.46E-07	0.0772	ACCGGAAGAG
1	ACCGGAAGNS	RPL29	-	148	157	7.46E-07	0.0772	ACCGGAAGAG
1	ACCGGAAGNS	MED31	+	97	106	7.46E-07	0.0772	ACCGGAAGAG
1	ACCGGAAGNS	SLC37A3	-	5	14	1.49E-06	0.0772	ACCGGAAGTG
1	ACCGGAAGNS	NUDT1	-	17	26	1.49E-06	0.0772	ACCGGAAGTG
1	ACCGGAAGNS	TNRC6A	-	56	65	1.49E-06	0.0772	ACCGGAAGTG
1	ACCGGAAGNS	RBM8A	-	73	82	1.49E-06	0.0772	ACCGGAAGTG
1	ACCGGAAGNS	ZNF230	-	100	109	1.49E-06	0.0772	ACCGGAAGTG
1	ACCGGAAGNS	CHUK	+	25	34	1.49E-06	0.0772	ACCGGAAGTG
1	ACCGGAAGNS	MRPL27	+	27	36	1.49E-06	0.0772	ACCGGAAGTG
1	ACCGGAAGNS	ERVK3-1	+	41	50	1.49E-06	0.0772	ACCGGAAGTG
1	ACCGGAAGNS	NUP54	+	47	56	1.49E-06	0.0772	ACCGGAAGTG
1	ACCGGAAGNS	MED8	+	79	88	1.49E-06	0.0772	ACCGGAAGTG
1	ACCGGAAGNS	MRPL3	+	80	89	1.49E-06	0.0772	ACCGGAAGTG
1	ACCGGAAGNS	MTPN	+	90	99	1.49E-06	0.0772	ACCGGAAGTG
1	ACCGGAAGNS	MED18	+	117	126	1.49E-06	0.0772	ACCGGAAGTG
1	ACCGGAAGNS	SNORA21	-	32	41	2.24E-06	0.0781	ACCGGAAGAC
1	ACCGGAAGNS	RPL18A	-	96	105	2.24E-06	0.0781	ACCGGAAGAC
1	ACCGGAAGNS	ARV1	-	156	165	2.24E-06	0.0781	ACCGGAAGAC
1	ACCGGAAGNS	RPS4X	+	166	175	2.24E-06	0.0781	ACCGGAAGAC
1	ACCGGAAGNS	SLX4	+	5	14	3.46E-06	0.0781	ACCGGAAGCG
1	ACCGGAAGNS	MINOS1	+	15	24	3.46E-06	0.0781	ACCGGAAGGG
1	ACCGGAAGNS	PSMC6	+	21	30	3.46E-06	0.0781	ACCGGAAGGG
1	ACCGGAAGNS	TEX22	+	29	38	3.46E-06	0.0781	ACCGGAAGCG
1	ACCGGAAGNS	ADH5	-	23	32	3.46E-06	0.0781	ACCGGAAGGG
1	ACCGGAAGNS	SARS	-	26	35	3.46E-06	0.0781	ACCGGAAGCG
1	ACCGGAAGNS	UBA52	+	56	65	3.46E-06	0.0781	ACCGGAAGCG

1	ACCGGAAGNS	WDR25	-	71	80	3.46E-06	0.0781	ACCGGAAGCG
1	ACCGGAAGNS	EPN1	-	78	87	3.46E-06	0.0781	ACCGGAAGCG
1	ACCGGAAGNS	UBL5	-	82	91	3.46E-06	0.0781	ACCGGAAGCG
1	ACCGGAAGNS	HSBP1	-	85	94	3.46E-06	0.0781	ACCGGAAGGG
1	ACCGGAAGNS	SNRPE	-	86	95	3.46E-06	0.0781	ACCGGAAGCG
1	ACCGGAAGNS	NGDN	+	104	113	3.46E-06	0.0781	ACCGGAAGGG
1	ACCGGAAGNS	MRPS23	-	97	106	3.46E-06	0.0781	ACCGGAAGCG
1	ACCGGAAGNS	YAE1D1	-	106	115	3.46E-06	0.0781	ACCGGAAGCG
1	ACCGGAAGNS	TOMM22	-	109	118	3.46E-06	0.0781	ACCGGAAGCG
1	ACCGGAAGNS	CLASRP	-	116	125	3.46E-06	0.0781	ACCGGAAGCG
1	ACCGGAAGNS	MGRN1	-	121	130	3.46E-06	0.0781	ACCGGAAGCG
1	ACCGGAAGNS	POM121C	-	2	11	4.21E-06	0.0805	ACCGGAAGTC
1	ACCGGAAGNS	POM121	-	8	17	4.21E-06	0.0805	ACCGGAAGTC
1	ACCGGAAGNS	SDHD	-	22	31	4.21E-06	0.0805	ACCGGAAGTC
1	ACCGGAAGNS	TIMMDC1	+	39	48	4.21E-06	0.0805	ACCGGAAGTC
1	ACCGGAAGNS	KMT5A	+	89	98	4.21E-06	0.0805	ACCGGAAGTC
1	ACCGGAAGNS	MAPK8IP3	-	86	95	4.21E-06	0.0805	ACCGGAAGTC
1	ACCGGAAGNS	LINC00493	+	174	183	4.21E-06	0.0805	ACCGGAAGTC
1	ACCGGAAGNS	RP11-57H14.5	+	58	67	5.43E-06	0.0828	GCCGGAAGAG
1	ACCGGAAGNS	SNRPD1	+	80	89	5.43E-06	0.0828	GCCGGAAGAG
1	ACCGGAAGNS	ERGIC3	+	119	128	5.43E-06	0.0828	GCCGGAAGAG
1	ACCGGAAGNS	RPL34	-	112	121	5.43E-06	0.0828	CCCGGAAGAG
1	ACCGGAAGNS	RPS10	+	124	133	5.43E-06	0.0828	CCCGGAAGAG
1	ACCGGAAGNS	COMMD8	+	138	147	5.43E-06	0.0828	GCCGGAAGAG
1	ACCGGAAGNS	AGO4	+	149	158	5.43E-06	0.0828	GCCGGAAGAG
1	ACCGGAAGNS	CHCHD2	-	185	194	6.34E-06	0.0828	ACCGGAAGAT
1	ACCGGAAGNS	LAMTOR1	+	91	100	7.57E-06	0.0828	ACCGGAAGCC
1	ACCGGAAGNS	AC021037.1	-	45	54	8.31E-06	0.0828	AGCGGAAGAG
1	ACCGGAAGNS	HSPA9	+	74	83	8.31E-06	0.0828	AGCGGAAGAG
1	ACCGGAAGNS	ARPC5	+	92	101	8.31E-06	0.0828	AGCGGAAGAG

1	ACCGGAAGNS	AUTS2	-	119	128	8.31E-06	0.0828	AGCGGAAGAG
1	ACCGGAAGNS	ATP5E	-	125	134	8.31E-06	0.0828	AGCGGAAGAG
1	ACCGGAAGNS	FYCO1	-	147	156	8.31E-06	0.0828	AGCGGAAGAG
1	ACCGGAAGNS	BPGM	+	7	16	9.22E-06	0.0828	AACGGAAGAG
1	ACCGGAAGNS	CPSF7	+	3	12	1.04E-05	0.0828	GCCGGAAGTG
1	ACCGGAAGNS	SF3A1	+	4	13	1.04E-05	0.0828	CCCGGAAGTG
1	ACCGGAAGNS	MFAP3	+	8	17	1.04E-05	0.0828	CCCGGAAGTG
1	ACCGGAAGNS	EEF1B2	+	10	19	1.04E-05	0.0828	GCCGGAAGTG
1	ACCGGAAGNS	MRPL47	+	12	21	1.04E-05	0.0828	GCCGGAAGTG
1	ACCGGAAGNS	MAGOH	-	3	12	1.04E-05	0.0828	CCCGGAAGTG
1	ACCGGAAGNS	RRAGA	+	21	30	1.04E-05	0.0828	GCCGGAAGTG
1	ACCGGAAGNS	GRPEL2	+	22	31	1.04E-05	0.0828	GCCGGAAGTG
1	ACCGGAAGNS	RPS19	-	14	23	1.04E-05	0.0828	GCCGGAAGTG
1	ACCGGAAGNS	NDUFB5	-	15	24	1.04E-05	0.0828	GCCGGAAGTG
1	ACCGGAAGNS	FAM114A2	-	15	24	1.04E-05	0.0828	CCCGGAAGTG
1	ACCGGAAGNS	FTSJ2	-	16	25	1.04E-05	0.0828	GCCGGAAGTG
1	ACCGGAAGNS	ATG4C	+	29	38	1.04E-05	0.0828	CCCGGAAGTG
1	ACCGGAAGNS	CSTF1	+	39	48	1.04E-05	0.0828	CCCGGAAGTG
1	ACCGGAAGNS	SSBP1	+	42	51	1.04E-05	0.0828	GCCGGAAGTG
1	ACCGGAAGNS	UQCRH	-	42	51	1.04E-05	0.0828	GCCGGAAGTG
1	ACCGGAAGNS	TOMM6	+	52	61	1.04E-05	0.0828	GCCGGAAGTG
1	ACCGGAAGNS	PSMB7	+	56	65	1.04E-05	0.0828	GCCGGAAGTG
1	ACCGGAAGNS	RPL32	+	72	81	1.04E-05	0.0828	GCCGGAAGTG
1	ACCGGAAGNS	SDHAF2	+	72	81	1.04E-05	0.0828	CCCGGAAGTG
1	ACCGGAAGNS	ROMO1	+	73	82	1.04E-05	0.0828	CCCGGAAGTG
1	ACCGGAAGNS	TMEM208	+	79	88	1.04E-05	0.0828	GCCGGAAGTG
1	ACCGGAAGNS	ZNF384	+	83	92	1.04E-05	0.0828	GCCGGAAGTG
1	ACCGGAAGNS	MARK3	-	75	84	1.04E-05	0.0828	GCCGGAAGTG
1	ACCGGAAGNS	DDX50	+	89	98	1.04E-05	0.0828	CCCGGAAGTG
1	ACCGGAAGNS	POMP	-	81	90	1.04E-05	0.0828	GCCGGAAGTG

1	ACCGGAAGNS	AKAP17A	+	95	104	1.04E-05	0.0828	GCCGGAAGTG
1	ACCGGAAGNS	MBD6	+	96	105	1.04E-05	0.0828	CCCGGAAGTG
1	ACCGGAAGNS	PNPLA4	-	87	96	1.04E-05	0.0828	CCCGGAAGTG
1	ACCGGAAGNS	HAT1	-	87	96	1.04E-05	0.0828	GCCGGAAGTG
1	ACCGGAAGNS	COMMD2	+	99	108	1.04E-05	0.0828	GCCGGAAGTG
1	ACCGGAAGNS	PARK7	+	100	109	1.04E-05	0.0828	GCCGGAAGTG
1	ACCGGAAGNS	ZNF384	+	104	113	1.04E-05	0.0828	CCCGGAAGTG
1	ACCGGAAGNS	SLC25A17	+	105	114	1.04E-05	0.0828	CCCGGAAGTG
1	ACCGGAAGNS	TM2D3	+	105	114	1.04E-05	0.0828	CCCGGAAGTG
1	ACCGGAAGNS	BOD1L1	-	98	107	1.04E-05	0.0828	CCCGGAAGTG
1	ACCGGAAGNS	CLPTM1L	-	100	109	1.04E-05	0.0828	GCCGGAAGTG
1	ACCGGAAGNS	EMC4	+	109	118	1.04E-05	0.0828	GCCGGAAGTG
1	ACCGGAAGNS	ELOVL6	-	102	111	1.04E-05	0.0828	GCCGGAAGTG
1	ACCGGAAGNS	PSMB1	+	118	127	1.04E-05	0.0828	GCCGGAAGTG
1	ACCGGAAGNS	FAM195B	-	111	120	1.04E-05	0.0828	GCCGGAAGTG
1	ACCGGAAGNS	LEPROTL1	-	112	121	1.04E-05	0.0828	CCCGGAAGTG
1	ACCGGAAGNS	NUP58	-	119	128	1.04E-05	0.0828	GCCGGAAGTG
1	ACCGGAAGNS	DDOST	-	120	129	1.04E-05	0.0828	GCCGGAAGTG
1	ACCGGAAGNS	PNPLA4	-	120	129	1.04E-05	0.0828	CCCGGAAGTG
1	ACCGGAAGNS	RTFDC1	+	130	139	1.04E-05	0.0828	GCCGGAAGTG
1	ACCGGAAGNS	RTFDC1	+	161	170	1.04E-05	0.0828	CCCGGAAGTG
1	ACCGGAAGNS	CHERP	-	176	185	1.04E-05	0.0828	GCCGGAAGTG
1	ACCGGAAGNS	APMAP	-	181	190	1.04E-05	0.0828	CCCGGAAGTG
1	ACCGGAAGNS	NELFE	-	11	20	1.23E-05	0.0901	ACCGGAAGTT
1	ACCGGAAGNS	TMEM199	-	64	73	1.23E-05	0.0901	ACCGGAAGTT
1	ACCGGAAGNS	AAR2	-	102	111	1.23E-05	0.0901	ACCGGAAGTT
1	ACCGGAAGNS	PSMD8	-	117	126	1.23E-05	0.0901	ACCGGAAGTT
1	ACCGGAAGNS	SSR2	-	127	136	1.23E-05	0.0901	ACAGGAAGAG
1	ACCGGAAGNS	LZIC	+	14	23	1.30E-05	0.0901	AGCGGAAGTG
1	ACCGGAAGNS	RPS6	-	39	48	1.30E-05	0.0901	AGCGGAAGTG

1	ACCGGAAGNS	SMU1	+	54	63	1.30E-05	0.0901	AGCGGAAGTG
1	ACCGGAAGNS	RPS18	-	56	65	1.30E-05	0.0901	AGCGGAAGTG
1	ACCGGAAGNS	PIK3R4	-	77	86	1.30E-05	0.0901	AGCGGAAGTG
1	ACCGGAAGNS	COPS3	+	88	97	1.30E-05	0.0901	AGCGGAAGTG
1	ACCGGAAGNS	SNRPE	-	80	89	1.30E-05	0.0901	AGCGGAAGTG
1	ACCGGAAGNS	LYRM2	-	92	101	1.30E-05	0.0901	AGCGGAAGTG
1	ACCGGAAGNS	COX6A1	-	100	109	1.30E-05	0.0901	AGCGGAAGTG
1	ACCGGAAGNS	PSMB4	-	100	109	1.30E-05	0.0901	AGCGGAAGTG
1	ACCGGAAGNS	TIMM23	+	117	126	1.30E-05	0.0901	AGCGGAAGTG
1	ACCGGAAGNS	TMEM116	-	101	110	1.42E-05	0.094	GCCGGAAGAC
1	ACCGGAAGNS	TIMM10B	+	36	45	1.51E-05	0.094	AACGGAAGTG
1	ACCGGAAGNS	KBTBD4	+	96	105	1.51E-05	0.094	AACGGAAGTG
1	ACCGGAAGNS	POMP	+	114	123	1.51E-05	0.094	AACGGAAGTG
1	ACCGGAAGNS	MYEOV2	+	9	18	1.79E-05	0.094	CCCGGAAGGG
1	ACCGGAAGNS	JKAMP	+	18	27	1.79E-05	0.094	CCCGGAAGCG
1	ACCGGAAGNS	RP11-46F15.2	+	19	28	1.79E-05	0.094	GCCGGAAGCG
1	ACCGGAAGNS	RPS21	+	24	33	1.79E-05	0.094	CCCGGAAGCG
1	ACCGGAAGNS	UVSSA	+	26	35	1.79E-05	0.094	GCCGGAAGGG
1	ACCGGAAGNS	TNRC6A	+	28	37	1.79E-05	0.094	CCCGGAAGGG
1	ACCGGAAGNS	FAM234A	+	33	42	1.79E-05	0.094	CCCGGAAGCG
1	ACCGGAAGNS	TMEM251	-	26	35	1.79E-05	0.094	GCCGGAAGCG
1	ACCGGAAGNS	SNAPC5	+	37	46	1.79E-05	0.094	GCCGGAAGGG
1	ACCGGAAGNS	NUDT1	+	40	49	1.79E-05	0.094	CCCGGAAGCG
1	ACCGGAAGNS	FAM103A1	+	41	50	1.79E-05	0.094	GCCGGAAGCG
1	ACCGGAAGNS	TRMT10C	+	58	67	1.79E-05	0.094	GCCGGAAGGG
1	ACCGGAAGNS	DPH3	+	68	77	1.79E-05	0.094	GCCGGAAGGG
1	ACCGGAAGNS	XPOT	+	69	78	1.79E-05	0.094	CCCGGAAGCG
1	ACCGGAAGNS	OXSM	-	65	74	1.79E-05	0.094	CCCGGAAGCG
1	ACCGGAAGNS	DIDO1	+	75	84	1.79E-05	0.094	CCCGGAAGCG
1	ACCGGAAGNS	PDIA3P2	-	68	77	1.79E-05	0.094	GCCGGAAGGG

1	ACCGGAAGNS	EP400	-	86	95	1.79E-05	0.094	GCCGGAAGGG
1	ACCGGAAGNS	MRPL20	+	99	108	1.79E-05	0.094	CCCGGAAGCG
1	ACCGGAAGNS	RARS	-	90	99	1.79E-05	0.094	CCCGGAAGCG
1	ACCGGAAGNS	LINC00493	+	104	113	1.79E-05	0.094	GCCGGAAGCG
1	ACCGGAAGNS	PROSER3	+	111	120	1.79E-05	0.094	GCCGGAAGCG
1	ACCGGAAGNS	COX6A1	-	106	115	1.79E-05	0.094	GCCGGAAGCG
1	ACCGGAAGNS	ADGRG6	+	115	124	1.79E-05	0.094	GCCGGAAGCG
1	ACCGGAAGNS	RBM45	+	116	125	1.79E-05	0.094	CCCGGAAGCG
1	ACCGGAAGNS	CLPTM1L	+	121	130	1.79E-05	0.094	CCCGGAAGCG
1	ACCGGAAGNS	AC114730.8	-	123	132	1.79E-05	0.094	CCCGGAAGCG
1	ACCGGAAGNS	METTL5	-	126	135	1.79E-05	0.094	GCCGGAAGCG
1	ACCGGAAGNS	KBTBD4	+	137	146	1.79E-05	0.094	GCCGGAAGCG
1	ACCGGAAGNS	RP11-274B21.4	-	134	143	1.79E-05	0.094	GCCGGAAGGG
1	ACCGGAAGNS	DDX18	+	149	158	1.79E-05	0.094	GCCGGAAGGG
1	ACCGGAAGNS	AC011747.4	+	158	167	1.79E-05	0.094	CCCGGAAGCG
1	ACCGGAAGNS	SNRPD1	-	175	184	1.79E-05	0.094	GCCGGAAGCG
1	ACCGGAAGNS	ZNF621	-	181	190	1.79E-05	0.094	GCCGGAAGGG
1	ACCGGAAGNS	ZFP41	+	191	200	1.79E-05	0.094	GCCGGAAGGG
1	ACCGGAAGNS	PRRC2C	-	183	192	1.79E-05	0.094	GCCGGAAGGG
1	ACCGGAAGNS	FAM50B	-	4	13	1.88E-05	0.094	ACAGGAAGTG
1	ACCGGAAGNS	RP11-46F15.2	-	46	55	1.88E-05	0.094	ACAGGAAGTG
1	ACCGGAAGNS	RP4-591C20.9	-	77	86	1.88E-05	0.094	ACAGGAAGTG
1	ACCGGAAGNS	FAM50B	-	87	96	1.88E-05	0.094	ACAGGAAGTG
1	ACCGGAAGNS	PSMB5	-	154	163	1.88E-05	0.094	ACAGGAAGTG
1	ACCGGAAGNS	TOMM5	+	19	28	1.88E-05	0.094	ACAGGAAGTG
1	ACCGGAAGNS	PIK3R4	+	92	101	1.88E-05	0.094	ACAGGAAGTG
1	ACCGGAAGNS	SMIM6	+	123	132	1.88E-05	0.094	ACAGGAAGTG
1	ACCGGAAGNS	CTA-221G9.12	+	186	195	1.88E-05	0.094	ACAGGAAGTG
1	ACCGGAAGNS	RP3-325F22.5	-	11	20	2.03E-05	0.0986	ACCGGAAGCT
1	ACCGGAAGNS	RPL29P14	-	65	74	2.03E-05	0.0986	ACCGGAAGCT

1	ACCGGAAGNS	PHF5A	-	143	152	2.03E-05	0.0986	ACCGGAAGCT
1	ACCGGAAGNS	RP11-403P17.6	-	155	164	2.03E-05	0.0986	ACCGGAAGCT
1	ACCGGAAGNS	SNRPG	+	41	50	2.03E-05	0.0986	ACCGGAAGCT
1	ACCGGAAGNS	RP11-568K15.1	-	16	25	2.24E-05	0.104	AGCGGAAGGG
1	ACCGGAAGNS	ADSL	-	108	117	2.24E-05	0.104	AGCGGAAGCG
1	ACCGGAAGNS	RPS15	+	62	71	2.24E-05	0.104	AGCGGAAGCG
1	ACCGGAAGNS	ATP6AP2	+	124	133	2.24E-05	0.104	AGCGGAAGCG
1	ACCGGAAGNS	RP11-459F6.3	+	142	151	2.24E-05	0.104	AGCGGAAGGG
1	ACCGGAAGNS	MRPS18C	+	163	172	2.24E-05	0.104	AGCGGAAGGG
1	ACCGGAAGNS	PTPN23	-	2	11	2.37E-05	0.104	GCCGGAAGTC
1	ACCGGAAGNS	ZNF814	-	11	20	2.37E-05	0.104	GCCGGAAGTC
1	ACCGGAAGNS	CTC-487M23.8	-	82	91	2.37E-05	0.104	GCCGGAAGTC
1	ACCGGAAGNS	SFT2D1	-	112	121	2.37E-05	0.104	GCCGGAAGTC
1	ACCGGAAGNS	SNHG3	-	112	121	2.37E-05	0.104	CCCGGAAGTC
1	ACCGGAAGNS	AKAP17A	+	9	18	2.37E-05	0.104	CCCGGAAGTC
1	ACCGGAAGNS	HCCS	+	67	76	2.37E-05	0.104	CCCGGAAGTC
1	ACCGGAAGNS	ZDHHC4	+	69	78	2.37E-05	0.104	GCCGGAAGTC
1	ACCGGAAGNS	SAR1A	+	83	92	2.37E-05	0.104	CCCGGAAGTC
1	ACCGGAAGNS	COX5B	+	85	94	2.37E-05	0.104	CCCGGAAGTC
1	ACCGGAAGNS	VPS25	+	94	103	2.37E-05	0.104	GCCGGAAGTC
1	ACCGGAAGNS	TMEM223	+	110	119	2.37E-05	0.104	CCCGGAAGTC
1	ACCGGAAGNS	MTX2	+	141	150	2.37E-05	0.104	GCCGGAAGTC
1	ACCGGAAGNS	PSMB4	-	106	115	2.61E-05	0.113	AACGGAAGCG
1	ACCGGAAGNS	HSBP1	+	62	71	2.61E-05	0.113	AACGGAAGCG
1	ACCGGAAGNS	CTD-2024I7.13	+	79	88	2.61E-05	0.113	AACGGAAGGG
1	ACCGGAAGNS	FAM103A1	+	47	56	2.68E-05	0.115	AGCGGAAGTC
1	ACCGGAAGNS	SRP14	+	87	96	2.68E-05	0.115	AGCGGAAGTC
1	ACCGGAAGNS	POM121C	-	81	90	2.83E-05	0.116	ACAGGAAGCG
1	ACCGGAAGNS	SLC37A2	+	7	16	2.83E-05	0.116	ACAGGAAGCG
1	ACCGGAAGNS	SCAF4	+	27	36	2.83E-05	0.116	ACAGGAAGCG

1	ACCGGAAGNS	SFT2D1	+	92	101	2.83E-05	0.116	ACAGGAAGCG
1	ACCGGAAGNS	TCP1	-	13	22	2.92E-05	0.116	AACGGAAGTC
1	ACCGGAAGNS	RP11-459F6.3	-	29	38	2.92E-05	0.116	AACGGAAGTC
1	ACCGGAAGNS	MRPL18	+	41	50	2.92E-05	0.116	AACGGAAGTC
1	ACCGGAAGNS	CFAP20	+	41	50	2.92E-05	0.116	AACGGAAGTC
1	ACCGGAAGNS	ZBTB6	-	124	133	3.07E-05	0.116	GCCGGAAGAT
1	ACCGGAAGNS	MTRNR2L10	+	53	62	3.07E-05	0.116	CCCGGAAGAT
1	ACCGGAAGNS	SLC25A5-AS1	-	7	16	3.27E-05	0.116	GCCGGAAGCC
1	ACCGGAAGNS	B3GALNT1	-	12	21	3.27E-05	0.116	GCCGGAAGCC
1	ACCGGAAGNS	ZNF785	-	13	22	3.27E-05	0.116	GCCGGAAGGC
1	ACCGGAAGNS	RNF144A-AS1	-	15	24	3.27E-05	0.116	CCCGGAAGGC
1	ACCGGAAGNS	C4orf46	-	21	30	3.27E-05	0.116	CCCGGAAGCC
1	ACCGGAAGNS	XPOT	-	28	37	3.27E-05	0.116	GCCGGAAGGC
1	ACCGGAAGNS	UQCR11	-	30	39	3.27E-05	0.116	CCCGGAAGCC
1	ACCGGAAGNS	FAM21C	-	50	59	3.27E-05	0.116	CCCGGAAGCC
1	ACCGGAAGNS	UBL5	-	56	65	3.27E-05	0.116	GCCGGAAGCC
1	ACCGGAAGNS	EMC3	-	59	68	3.27E-05	0.116	GCCGGAAGCC
1	ACCGGAAGNS	NDUFS5	-	63	72	3.27E-05	0.116	GCCGGAAGCC
1	ACCGGAAGNS	SACM1L	-	71	80	3.27E-05	0.116	CCCGGAAGCC
1	ACCGGAAGNS	CTD-2619J13.14	-	75	84	3.27E-05	0.116	GCCGGAAGCC
1	ACCGGAAGNS	AC005785.2	-	79	88	3.27E-05	0.116	CCCGGAAGCC
1	ACCGGAAGNS	SLC22A23	-	93	102	3.27E-05	0.116	CCCGGAAGCC
1	ACCGGAAGNS	AC006548.28	-	94	103	3.27E-05	0.116	CCCGGAAGCC
1	ACCGGAAGNS	NUP54	-	107	116	3.27E-05	0.116	GCCGGAAGGC
1	ACCGGAAGNS	ZW10	-	113	122	3.27E-05	0.116	CCCGGAAGGC
1	ACCGGAAGNS	CHID1	-	125	134	3.27E-05	0.116	GCCGGAAGCC
1	ACCGGAAGNS	FAM21A	-	134	143	3.27E-05	0.116	CCCGGAAGCC
1	ACCGGAAGNS	RP1-101K10.6	-	137	146	3.27E-05	0.116	GCCGGAAGGC
1	ACCGGAAGNS	GRID2IP	-	152	161	3.27E-05	0.116	GCCGGAAGCC
1	ACCGGAAGNS	EMC7	-	171	180	3.27E-05	0.116	CCCGGAAGCC

1	ACCGGAAGNS	SSR3	+	4	13	3.27E-05	0.116	CCCGGAAGCC
1	ACCGGAAGNS	STIM2	+	14	23	3.27E-05	0.116	GCCGGAAGCC
1	ACCGGAAGNS	EHBP1L1	+	24	33	3.27E-05	0.116	CCCGGAAGCC
1	ACCGGAAGNS	UBR1	+	49	58	3.27E-05	0.116	GCCGGAAGCC
1	ACCGGAAGNS	RBM45	+	59	68	3.27E-05	0.116	CCCGGAAGGC
1	ACCGGAAGNS	PLEKHG2	+	60	69	3.27E-05	0.116	GCCGGAAGGC
1	ACCGGAAGNS	COX14	+	91	100	3.27E-05	0.116	CCCGGAAGCC
1	ACCGGAAGNS	PGM2	+	144	153	3.27E-05	0.116	GCCGGAAGGC
1	ACCGGAAGNS	GTF3C6	+	147	156	3.27E-05	0.116	GCCGGAAGCC
1	ACCGGAAGNS	AKAP17A	-	76	85	3.39E-05	0.117	CGCGGAAGAG
1	ACCGGAAGNS	TOMM7	+	5	14	3.39E-05	0.117	GGCGGAAGAG
1	ACCGGAAGNS	SLC25A3	+	19	28	3.39E-05	0.117	GGCGGAAGAG
1	ACCGGAAGNS	SRRD	+	103	112	3.39E-05	0.117	CGCGGAAGAG
1	ACCGGAAGNS	UMPS	+	115	124	3.39E-05	0.117	GGCGGAAGAG
1	ACCGGAAGNS	RPL23	+	119	128	3.39E-05	0.117	GGCGGAAGAG
1	ACCGGAAGNS	FBXW4P1	+	156	165	3.39E-05	0.117	GGCGGAAGAG
1	ACCGGAAGNS	TIMMDC1	-	106	115	3.48E-05	0.12	ACAGGAAGTC
1	ACCGGAAGNS	NABP2	+	135	144	3.48E-05	0.12	ACAGGAAGTC
1	ACCGGAAGNS	SRRM2	+	82	91	3.70E-05	0.124	AGCGGAAGGC
1	ACCGGAAGNS	RTN3P1	+	182	191	3.70E-05	0.124	AGCGGAAGCC
1	ACCGGAAGNS	RPL27	-	35	44	3.85E-05	0.124	GACGGAAGAG
1	ACCGGAAGNS	URM1	-	73	82	3.85E-05	0.124	GACGGAAGAG
1	ACCGGAAGNS	GRPEL1	-	107	116	3.85E-05	0.124	CACGGAAGAG
1	ACCGGAAGNS	SEP15	+	5	14	3.85E-05	0.124	GACGGAAGAG
1	ACCGGAAGNS	MIR941-1	+	17	26	3.85E-05	0.124	CACGGAAGAG
1	ACCGGAAGNS	ARHGAP19	+	60	69	3.85E-05	0.124	CACGGAAGAG
1	ACCGGAAGNS	MIR941-1	+	73	82	3.85E-05	0.124	CACGGAAGAG
1	ACCGGAAGNS	ASGR1	+	112	121	3.85E-05	0.124	CACGGAAGAG
1	ACCGGAAGNS	MIR941-1	+	129	138	3.85E-05	0.124	CACGGAAGAG
1	ACCGGAAGNS	MIR941-1	+	185	194	3.85E-05	0.124	CACGGAAGAG

1	ACCGGAAGNS	RP11-84D1.1	-	114	123	3.96E-05	0.124	AACGGAAGAT
1	ACCGGAAGNS	UNC50	-	1	10	4.41E-05	0.124	GCCGGAAGTT
1	ACCGGAAGNS	MRPS22	-	2	11	4.41E-05	0.124	CCAGGAAGAG
1	ACCGGAAGNS	CHCHD2	-	10	19	4.41E-05	0.124	CCCGGAAGTT
1	ACCGGAAGNS	DCTN3	-	18	27	4.41E-05	0.124	GCCGGAAGTT
1	ACCGGAAGNS	PRSS48	-	42	51	4.41E-05	0.124	CCAGGAAGAG
1	ACCGGAAGNS	RP11-568K15.1	-	53	62	4.41E-05	0.124	GCAGGAAGAG
1	ACCGGAAGNS	RP11-284F21.7	-	62	71	4.41E-05	0.124	AACGGAAGCC
1	ACCGGAAGNS	YARS	-	64	73	4.41E-05	0.124	GCAGGAAGAG
1	ACCGGAAGNS	TRMT10C	-	77	86	4.41E-05	0.124	GCCGGAAGTT
1	ACCGGAAGNS	COX8A	-	94	103	4.41E-05	0.124	GCCGGAAGTT
1	ACCGGAAGNS	SCARNA21	-	106	115	4.41E-05	0.124	CCCGGAAGTT
1	ACCGGAAGNS	CREG1	-	107	116	4.41E-05	0.124	CCAGGAAGAG
1	ACCGGAAGNS	COIL	-	112	121	4.41E-05	0.124	AACGGAAGCC
1	ACCGGAAGNS	SRP14	-	114	123	4.41E-05	0.124	GCCGGAAGTT
1	ACCGGAAGNS	CHID1	-	116	125	4.41E-05	0.124	CCAGGAAGAG
1	ACCGGAAGNS	RPL11	-	128	137	4.41E-05	0.124	GCAGGAAGAG
1	ACCGGAAGNS	RP11-466A19.1	-	165	174	4.41E-05	0.124	GCAGGAAGAG
1	ACCGGAAGNS	PSMC1	-	167	176	4.41E-05	0.124	GCCGGAAGTT
1	ACCGGAAGNS	RP3-395C13.1	-	170	179	4.41E-05	0.124	CCAGGAAGAG
1	ACCGGAAGNS	ADSS	-	175	184	4.41E-05	0.124	GCAGGAAGAG
1	ACCGGAAGNS	UQCR11	+	11	20	4.41E-05	0.124	GCCGGAAGTT
1	ACCGGAAGNS	NME1	+	15	24	4.41E-05	0.124	GCCGGAAGTT
1	ACCGGAAGNS	RP11-395B7.2	+	16	25	4.41E-05	0.124	CCAGGAAGAG
1	ACCGGAAGNS	CCDC74BP1	+	18	27	4.41E-05	0.124	CCAGGAAGAG
1	ACCGGAAGNS	TARS	+	24	33	4.41E-05	0.124	CCCGGAAGTT
1	ACCGGAAGNS	UQCRQ	+	28	37	4.41E-05	0.124	CCCGGAAGTT
1	ACCGGAAGNS	HMGN1P2	+	43	52	4.41E-05	0.124	GCAGGAAGAG
1	ACCGGAAGNS	PSMB3	+	71	80	4.41E-05	0.124	CCAGGAAGAG
1	ACCGGAAGNS	COX14	+	98	107	4.41E-05	0.124	GCCGGAAGTT

1	ACCGGAAGNS	RP11-802O23.3	+	103	112	4.41E-05	0.124	CCAGGAAGAG
1	ACCGGAAGNS	COX5B	+	117	126	4.41E-05	0.124	CCCGGAAGTT
1	ACCGGAAGNS	URM1	+	119	128	4.41E-05	0.124	GCCGGAAGTT
1	ACCGGAAGNS	JAGN1	+	147	156	4.41E-05	0.124	GCCGGAAGTT
1	ACCGGAAGNS	COPB2	+	155	164	4.41E-05	0.124	CCCGGAAGTT
1	ACCGGAAGNS	CTB-129O4.1	+	181	190	4.41E-05	0.124	GCAGGAAGAG
1	ACCGGAAGNS	RP4-734G22.3	+	185	194	4.41E-05	0.124	GCAGGAAGAG
1	ACCGGAAGNS	RP11-449P15.2	-	20	29	4.53E-05	0.124	GGCGGAAGTG
1	ACCGGAAGNS	ZDHHC4	-	62	71	4.53E-05	0.124	GGCGGAAGTG
1	ACCGGAAGNS	MAGOH	-	67	76	4.53E-05	0.124	CGCGGAAGTG
1	ACCGGAAGNS	TIMM17A	-	71	80	4.53E-05	0.124	GGCGGAAGTG
1	ACCGGAAGNS	RPS3	-	111	120	4.53E-05	0.124	GGCGGAAGTG
1	ACCGGAAGNS	RPS3A	-	148	157	4.53E-05	0.124	GGCGGAAGTG
1	ACCGGAAGNS	NGDN	+	4	13	4.53E-05	0.124	GGCGGAAGTG
1	ACCGGAAGNS	NECAP1	+	21	30	4.53E-05	0.124	CGCGGAAGTG
1	ACCGGAAGNS	ERVK3-1	+	28	37	4.53E-05	0.124	GGCGGAAGTG
1	ACCGGAAGNS	CLASRP	+	49	58	4.53E-05	0.124	GGCGGAAGTG
1	ACCGGAAGNS	NELFE	+	84	93	4.53E-05	0.124	GGCGGAAGTG
1	ACCGGAAGNS	LEPROTL1	+	92	101	4.53E-05	0.124	GGCGGAAGTG
1	ACCGGAAGNS	ATAT1	+	97	106	4.53E-05	0.124	GGCGGAAGTG
1	ACCGGAAGNS	TDG	+	123	132	4.53E-05	0.124	GGCGGAAGTG
1	ACCGGAAGNS	TMBIM4	+	157	166	4.53E-05	0.124	CGCGGAAGTG
1	ACCGGAAGNS	LRRC31	+	181	190	4.64E-05	0.127	ACAGGAAGAT
1	ACCGGAAGNS	PPIB	-	48	57	4.79E-05	0.13	ACAGGAAGGC
1	ACCGGAAGNS	TMEM179B	-	138	147	4.79E-05	0.13	ACAGGAAGGC
1	ACCGGAAGNS	TRBV6-1	+	37	46	4.79E-05	0.13	ACAGGAAGGC
1	ACCGGAAGNS	MTA1	-	8	17	4.97E-05	0.13	AGAGGAAGAG
1	ACCGGAAGNS	M6PR	-	38	47	4.97E-05	0.13	AGCGGAAGTT
1	ACCGGAAGNS	RPL17P11	-	57	66	4.97E-05	0.13	AGAGGAAGAG
1	ACCGGAAGNS	UBL5	-	76	85	4.97E-05	0.13	AGCGGAAGTT

1	ACCGGAAGNS	TM2D3	-	185	194	4.97E-05	0.13	AGAGGAAGAG
1	ACCGGAAGNS	TBC1D31	+	32	41	4.97E-05	0.13	AGAGGAAGAG
1	ACCGGAAGNS	MTPN	+	49	58	4.97E-05	0.13	AGAGGAAGAG
1	ACCGGAAGNS	RPS6	+	105	114	4.97E-05	0.13	AGCGGAAGTT
1	ACCGGAAGNS	CTC-260E6.6	+	121	130	4.97E-05	0.13	AGAGGAAGAG
1	ACCGGAAGNS	RP11-454L9.2	+	139	148	4.97E-05	0.13	AGAGGAAGAG
1	ACCGGAAGNS	RP11-436H11.5	+	182	191	4.97E-05	0.13	AGAGGAAGAG
1	ACCGGAAGNS	TCP1	-	96	105	5.12E-05	0.132	GACGGAAGTG
1	ACCGGAAGNS	SAP18	+	49	58	5.12E-05	0.132	CACGGAAGTG
1	ACCGGAAGNS	RPS14	+	81	90	5.12E-05	0.132	CACGGAAGTG
1	ACCGGAAGNS	TCEB1	+	103	112	5.12E-05	0.132	GACGGAAGTG
1	ACCGGAAGNS	TTC1	+	104	113	5.12E-05	0.132	GACGGAAGTG
1	ACCGGAAGNS	C16orf91	+	121	130	5.12E-05	0.132	GACGGAAGTG
1	ACCGGAAGNS	CDC26	+	79	88	5.24E-05	0.134	CGCGGAAGAC
1	ACCGGAAGNS	CTD-3099C6.11	-	26	35	5.46E-05	0.134	AAAGGAAGAG
1	ACCGGAAGNS	SCOC	-	51	60	5.46E-05	0.134	AAAGGAAGAG
1	ACCGGAAGNS	CREB3	-	70	79	5.46E-05	0.134	AAAGGAAGAG
1	ACCGGAAGNS	RBP1	-	84	93	5.46E-05	0.134	AAAGGAAGAG
1	ACCGGAAGNS	SLC25A5P6	-	87	96	5.46E-05	0.134	AAAGGAAGAG
1	ACCGGAAGNS	RPL39	-	119	128	5.46E-05	0.134	AAAGGAAGAG
1	ACCGGAAGNS	EEF1A1P42	+	101	110	5.46E-05	0.134	AAAGGAAGAG
1	ACCGGAAGNS	CHCHD2	+	126	135	5.46E-05	0.134	AACGGAAGTT
1	ACCGGAAGNS	ACSL1	+	166	175	5.46E-05	0.134	AAAGGAAGAG
1	ACCGGAAGNS	VDR	+	179	188	5.46E-05	0.134	AAAGGAAGAG
1	ACCGGAAGNS	BST2	-	3	12	5.61E-05	0.134	CCAGGAAGTG
1	ACCGGAAGNS	RIN2	-	46	55	5.61E-05	0.134	GCAGGAAGTG
1	ACCGGAAGNS	DCTN3	-	51	60	5.61E-05	0.134	GCAGGAAGTG
1	ACCGGAAGNS	STRAP	-	64	73	5.61E-05	0.134	GCAGGAAGTG
1	ACCGGAAGNS	RPS13	-	72	81	5.61E-05	0.134	GCAGGAAGTG
1	ACCGGAAGNS	COX4I1	-	83	92	5.61E-05	0.134	GCAGGAAGTG

1	ACCGGAAGNS	CTB-13L3.1	-	135	144	5.61E-05	0.134	CCAGGAAGTG
1	ACCGGAAGNS	ATP6V0E1	-	145	154	5.61E-05	0.134	CCAGGAAGTG
1	ACCGGAAGNS	SNORA62	+	17	26	5.61E-05	0.134	CCAGGAAGTG
1	ACCGGAAGNS	SNORA6	+	17	26	5.61E-05	0.134	CCAGGAAGTG
1	ACCGGAAGNS	RP11-1228E12.1	+	44	53	5.61E-05	0.134	CCAGGAAGTG
1	ACCGGAAGNS	IKBKB	+	112	121	5.61E-05	0.134	GCAGGAAGTG
1	ACCGGAAGNS	OMA1	+	132	141	5.61E-05	0.134	GCAGGAAGTG
1	ACCGGAAGNS	RPL37	+	158	167	5.61E-05	0.134	GCAGGAAGTG
1	ACCGGAAGNS	DLGAP4-AS1	+	162	171	5.61E-05	0.134	GCAGGAAGTG
1	ACCGGAAGNS	MINOS1	+	189	198	5.61E-05	0.134	GCAGGAAGTG
1	ACCGGAAGNS	SDHAF2	-	13	22	5.86E-05	0.135	GCCGGAAGGT
1	ACCGGAAGNS	CHMP2A	-	79	88	5.86E-05	0.135	CCCGGAAGGT
1	ACCGGAAGNS	TIMM23	-	91	100	5.86E-05	0.135	CCCGGAAGCT
1	ACCGGAAGNS	VPS25	-	114	123	5.86E-05	0.135	CCCGGAAGCT
1	ACCGGAAGNS	TMEM126A	+	16	25	5.86E-05	0.135	GCCGGAAGCT
1	ACCGGAAGNS	TMEM251	+	40	49	5.86E-05	0.135	GCCGGAAGCT
1	ACCGGAAGNS	MARS2	+	48	57	5.86E-05	0.135	GCCGGAAGCT
1	ACCGGAAGNS	NDUFB7	+	72	81	5.86E-05	0.135	CCCGGAAGCT
1	ACCGGAAGNS	SFSWAP	+	89	98	5.86E-05	0.135	GCCGGAAGCT
1	ACCGGAAGNS	C6orf99	+	90	99	5.86E-05	0.135	CCCGGAAGGT
1	ACCGGAAGNS	RPL11	+	111	120	5.86E-05	0.135	GCCGGAAGCT
1	ACCGGAAGNS	TIMM23	+	149	158	5.86E-05	0.135	CCCGGAAGGT
1	ACCGGAAGNS	RPL13AP5	+	189	198	5.86E-05	0.135	GCCGGAAGGT
1	ACCGGAAGNS	RAD51-AS1	-	11	20	6.21E-05	0.136	GGCGGAAGCG
1	ACCGGAAGNS	ATXN2L	-	31	40	6.21E-05	0.136	GGCGGAAGGG
1	ACCGGAAGNS	M6PR	-	44	53	6.21E-05	0.136	GGCGGAAGCG
1	ACCGGAAGNS	HSD17B10	-	51	60	6.21E-05	0.136	GGCGGAAGGG
1	ACCGGAAGNS	TMEM141	-	69	78	6.21E-05	0.136	GGCGGAAGGG
1	ACCGGAAGNS	ASNS	-	82	91	6.21E-05	0.136	GGCGGAAGGG
1	ACCGGAAGNS	C11orf49	-	121	130	6.21E-05	0.136	GGCGGAAGGG

1	ACCGGAAGNS	XPNPEP1	-	157	166	6.21E-05	0.136	CGCGGAAGGG
1	ACCGGAAGNS	RP4-761J14.8	-	192	201	6.21E-05	0.136	GGCGGAAGCG
1	ACCGGAAGNS	MACF1	+	34	43	6.21E-05	0.136	GGCGGAAGCG
1	ACCGGAAGNS	EP400	+	51	60	6.21E-05	0.136	CGCGGAAGCG
1	ACCGGAAGNS	SMG6	+	54	63	6.21E-05	0.136	GGCGGAAGGG
1	ACCGGAAGNS	MCM6	+	62	71	6.21E-05	0.136	GGCGGAAGCG
1	ACCGGAAGNS	UTP6	+	92	101	6.21E-05	0.136	GGCGGAAGCG
1	ACCGGAAGNS	MTX2	+	109	118	6.21E-05	0.136	GGCGGAAGCG
1	ACCGGAAGNS	ADSS	+	121	130	6.21E-05	0.136	CGCGGAAGGG
1	ACCGGAAGNS	DPM3	+	124	133	6.21E-05	0.136	CGCGGAAGCG
1	ACCGGAAGNS	CHERP	+	131	140	6.21E-05	0.136	GGCGGAAGCG
1	ACCGGAAGNS	FBXW4P1	+	173	182	6.21E-05	0.136	GGCGGAAGGG
1	ACCGGAAGNS	DOCK5	+	184	193	6.21E-05	0.136	GGCGGAAGCG
1	ACCGGAAGNS	RPL34	-	54	63	6.32E-05	0.138	ACAGGAAGTT
1	ACCGGAAGNS	RPS11	-	112	121	6.41E-05	0.139	AGAGGAAGTG
1	ACCGGAAGNS	BAZ2A	-	159	168	6.41E-05	0.139	AGAGGAAGTG
1	ACCGGAAGNS	NTS	+	135	144	6.41E-05	0.139	AGAGGAAGTG
1	ACCGGAAGNS	MDH1	+	42	51	6.56E-05	0.142	AGCGGAAGGT
1	ACCGGAAGNS	MIR6838	-	92	101	6.71E-05	0.143	GCAGGAAGAC
1	ACCGGAAGNS	RP11-266L9.6	-	103	112	6.71E-05	0.143	CCAGGAAGAC
1	ACCGGAAGNS	MIR6859-1	-	182	191	6.71E-05	0.143	CCAGGAAGAC
1	ACCGGAAGNS	TIMM21	+	92	101	6.71E-05	0.143	CCAGGAAGAC
1	ACCGGAAGNS	RPL7AP15	+	162	171	6.71E-05	0.143	GCAGGAAGAC
1	ACCGGAAGNS	ARL14EP	-	48	57	6.95E-05	0.146	GACGGAAGGG
1	ACCGGAAGNS	RARS	-	116	125	6.95E-05	0.146	GACGGAAGCG
1	ACCGGAAGNS	COBL	-	125	134	6.95E-05	0.146	GACGGAAGGG
1	ACCGGAAGNS	DYNLT1	+	176	185	6.95E-05	0.146	GACGGAAGGG
1	ACCGGAAGNS	ATXN10	-	7	16	7.08E-05	0.146	GGCGGAAGTC
1	ACCGGAAGNS	SNORD105B	-	38	47	7.08E-05	0.146	GGCGGAAGTC
1	ACCGGAAGNS	COX8A	-	55	64	7.08E-05	0.146	CGCGGAAGTC

1	ACCGGAAGNS	PSMA4	-	71	80	7.08E-05	0.146	CGCGGAAGTC
1	ACCGGAAGNS	TCTE3	-	79	88	7.08E-05	0.146	GGCGGAAGTC
1	ACCGGAAGNS	PSMC1	-	133	142	7.08E-05	0.146	GGCGGAAGTC
1	ACCGGAAGNS	UNC50	+	64	73	7.08E-05	0.146	GGCGGAAGTC
1	ACCGGAAGNS	CIAPIN1	+	89	98	7.08E-05	0.146	GGCGGAAGTC
1	ACCGGAAGNS	TMEM199	+	103	112	7.08E-05	0.146	GGCGGAAGTC
1	ACCGGAAGNS	SLC9B1	+	118	127	7.08E-05	0.146	CGCGGAAGTC
1	ACCGGAAGNS	XXbac-B476C20.11	-	25	34	7.19E-05	0.146	AAAGGAAGTG
1	ACCGGAAGNS	CELA2B	-	32	41	7.19E-05	0.146	AAAGGAAGTG
1	ACCGGAAGNS	RPS3	-	128	137	7.19E-05	0.146	AAAGGAAGTG
1	ACCGGAAGNS	RPL18A	-	141	150	7.19E-05	0.146	AAAGGAAGTG
1	ACCGGAAGNS	FEN1	+	1	10	7.19E-05	0.146	AAAGGAAGTG
1	ACCGGAAGNS	COX16	+	35	44	7.19E-05	0.146	AAAGGAAGTG
1	ACCGGAAGNS	SC5D	+	53	62	7.19E-05	0.146	AAAGGAAGTG
1	ACCGGAAGNS	CTD-2357A8.2	+	128	137	7.19E-05	0.146	AAAGGAAGTG
1	ACCGGAAGNS	SSBP3	-	13	22	7.70E-05	0.147	GCAGGAAGGG
1	ACCGGAAGNS	MUC12	-	24	33	7.70E-05	0.147	CCAGGAAGGG
1	ACCGGAAGNS	SETD1B	-	57	66	7.70E-05	0.147	CCAGGAAGGG
1	ACCGGAAGNS	RNU4ATAC	-	65	74	7.70E-05	0.147	GCAGGAAGCG
1	ACCGGAAGNS	TMEM179B	-	67	76	7.70E-05	0.147	CCAGGAAGCG
1	ACCGGAAGNS	TLN2	-	68	77	7.70E-05	0.147	GCAGGAAGCG
1	ACCGGAAGNS	SNORA71A	-	72	81	7.70E-05	0.147	CCAGGAAGGG
1	ACCGGAAGNS	RPS7	-	93	102	7.70E-05	0.147	CCAGGAAGCG
1	ACCGGAAGNS	POM121	-	101	110	7.70E-05	0.147	GCAGGAAGCG
1	ACCGGAAGNS	C11orf31	-	146	155	7.70E-05	0.147	GCAGGAAGGG
1	ACCGGAAGNS	DBI	-	166	175	7.70E-05	0.147	CCAGGAAGCG
1	ACCGGAAGNS	ACTBP9	-	169	178	7.70E-05	0.147	AGAGGAAGAC
1	ACCGGAAGNS	GLA	-	171	180	7.70E-05	0.147	CCAGGAAGCG
1	ACCGGAAGNS	DAPK1	-	183	192	7.70E-05	0.147	GCAGGAAGGG
1	ACCGGAAGNS	ZYX	-	192	201	7.70E-05	0.147	CCAGGAAGGG

1	ACCGGAAGNS	SNHG3	+	7	16	7.70E-05	0.147	CCAGGAAGGG
1	ACCGGAAGNS	ASGR1	+	29	38	7.70E-05	0.147	GCAGGAAGCG
1	ACCGGAAGNS	GMNN	+	41	50	7.70E-05	0.147	GCAGGAAGGG
1	ACCGGAAGNS	SHFM1	+	46	55	7.70E-05	0.147	GCAGGAAGCG
1	ACCGGAAGNS	PRSS48	+	69	78	7.70E-05	0.147	GCAGGAAGCG
1	ACCGGAAGNS	SARS	+	74	83	7.70E-05	0.147	GCAGGAAGGG
1	ACCGGAAGNS	SNRPF	+	75	84	7.70E-05	0.147	GCAGGAAGGG
1	ACCGGAAGNS	C14orf142	+	84	93	7.70E-05	0.147	GCAGGAAGGG
1	ACCGGAAGNS	FBXW4P1	+	84	93	7.70E-05	0.147	GCAGGAAGGG
1	ACCGGAAGNS	TPM3P9	+	104	113	7.70E-05	0.147	CCAGGAAGCG
1	ACCGGAAGNS	ZBTB7A	+	154	163	7.70E-05	0.147	CCAGGAAGGG
1	ACCGGAAGNS	CTD-2561B21.8	+	162	171	7.70E-05	0.147	CCAGGAAGCG
1	ACCGGAAGNS	DHX9	+	172	181	7.70E-05	0.147	CCAGGAAGCG
1	ACCGGAAGNS	MIR6838	+	180	189	7.70E-05	0.147	AGAGGAAGAC
1	ACCGGAAGNS	MIR6838	+	132	141	7.85E-05	0.149	CACGGAAGTC
1	ACCGGAAGNS	RP11-371E8.4	+	53	62	8.03E-05	0.152	ACAGGAAGGT
1	ACCGGAAGNS	WDR83	+	65	74	8.03E-05	0.152	ACAGGAAGGT
1	ACCGGAAGNS	TMEM251	+	97	106	8.03E-05	0.152	ACAGGAAGGT
1	ACCGGAAGNS	EIF4A1P11	-	11	20	8.29E-05	0.153	AAAGGAAGAC
1	ACCGGAAGNS	SH3GL1P1	-	27	36	8.29E-05	0.153	AGAGGAAGGG
1	ACCGGAAGNS	LINC01609	-	27	36	8.29E-05	0.153	AGAGGAAGGG
1	ACCGGAAGNS	SEMA4G	-	96	105	8.29E-05	0.153	AGAGGAAGGG
1	ACCGGAAGNS	SCOCP1	-	133	142	8.29E-05	0.153	AGAGGAAGGG
1	ACCGGAAGNS	CTD-2373N4.5	-	152	161	8.29E-05	0.153	AAAGGAAGAC
1	ACCGGAAGNS	RP11-697E2.6	+	1	10	8.29E-05	0.153	AGAGGAAGCG
1	ACCGGAAGNS	RC3H1	+	25	34	8.29E-05	0.153	AGAGGAAGGG
1	ACCGGAAGNS	RPS13	+	48	57	8.29E-05	0.153	AGAGGAAGCG
1	ACCGGAAGNS	NME1	+	67	76	8.29E-05	0.153	AGAGGAAGCG
1	ACCGGAAGNS	FBXW4P1	+	125	134	8.29E-05	0.153	AGAGGAAGGG
1	ACCGGAAGNS	ILF2	+	177	186	8.29E-05	0.153	AGAGGAAGGG

1	ACCGGAAGNS	ATP6V1E1	-	15	24	8.44E-05	0.153	CCAGGAAGTC
1	ACCGGAAGNS	CCL15-CCL14	-	17	26	8.44E-05	0.153	CCAGGAAGTC
1	ACCGGAAGNS	MRPS22	-	41	50	8.44E-05	0.153	GCAGGAAGTC
1	ACCGGAAGNS	SLC25A5P6	-	130	139	8.44E-05	0.153	CCAGGAAGTC
1	ACCGGAAGNS	ZDHHC4	+	115	124	8.44E-05	0.153	GCAGGAAGTC
1	ACCGGAAGNS	PAXBP1-AS1	+	8	17	8.59E-05	0.153	GGCGGAAGAT
1	ACCGGAAGNS	RP11-156P1.3	+	118	127	8.59E-05	0.153	GGCGGAAGAT
1	ACCGGAAGNS	CTD-2154B17.4	-	15	24	8.79E-05	0.153	CGCGGAAGCC
1	ACCGGAAGNS	METTL12	-	22	31	8.79E-05	0.153	CGCGGAAGGC
1	ACCGGAAGNS	PPIB	-	34	43	8.79E-05	0.153	GGCGGAAGGC
1	ACCGGAAGNS	ATP5E	-	42	51	8.79E-05	0.153	CGCGGAAGCC
1	ACCGGAAGNS	KIZ	-	63	72	8.79E-05	0.153	GGCGGAAGCC
1	ACCGGAAGNS	MSL3P1	-	87	96	8.79E-05	0.153	GGCGGAAGCC
1	ACCGGAAGNS	KBTBD7	-	144	153	8.79E-05	0.153	CGCGGAAGCC
1	ACCGGAAGNS	RNF7	-	186	195	8.79E-05	0.153	GGCGGAAGGC
1	ACCGGAAGNS	RPL10	+	10	19	8.79E-05	0.153	CGCGGAAGGC
1	ACCGGAAGNS	TMEM59	+	27	36	8.79E-05	0.153	GGCGGAAGGC
1	ACCGGAAGNS	DNMBP	+	33	42	8.79E-05	0.153	GGCGGAAGCC
1	ACCGGAAGNS	MAST3	+	60	69	8.79E-05	0.153	GGCGGAAGGC
1	ACCGGAAGNS	LINC00939	+	69	78	8.79E-05	0.153	GGCGGAAGCC
1	ACCGGAAGNS	TMEM223	+	77	86	8.79E-05	0.153	GGCGGAAGGC
1	ACCGGAAGNS	TRIM8	+	80	89	8.79E-05	0.153	CGCGGAAGGC
1	ACCGGAAGNS	ALG6	+	99	108	8.79E-05	0.153	GGCGGAAGGC
1	ACCGGAAGNS	MSRB1	+	131	140	8.79E-05	0.153	GGCGGAAGCC
1	ACCGGAAGNS	SLC25A20	+	134	143	8.79E-05	0.153	GGCGGAAGCC
1	ACCGGAAGNS	CHCHD1	+	176	185	8.79E-05	0.153	CGCGGAAGCC
1	ACCGGAAGNS	PSAP	-	39	48	8.98E-05	0.154	AAAGGAAGGG
1	ACCGGAAGNS	ZBTB4	-	49	58	8.98E-05	0.154	AAAGGAAGGG
1	ACCGGAAGNS	SLC34A2	-	62	71	8.98E-05	0.154	AAAGGAAGGG
1	ACCGGAAGNS	ST6GALNAC1	-	76	85	8.98E-05	0.154	AAAGGAAGGG

1	ACCGGAAGNS	DDX50	-	133	142	8.98E-05	0.154	AAAGGAAGCG
1	ACCGGAAGNS	RP11-474I11.8	+	17	26	8.98E-05	0.154	AAAGGAAGGG
1	ACCGGAAGNS	NUDT5	+	40	49	8.98E-05	0.154	AAAGGAAGCG
1	ACCGGAAGNS	CD14	+	53	62	8.98E-05	0.154	AAAGGAAGGG
1	ACCGGAAGNS	MIR6124	+	114	123	8.98E-05	0.154	AAAGGAAGGG
1	ACCGGAAGNS	RPL24	+	14	23	9.07E-05	0.154	AGAGGAAGTC
1	ACCGGAAGNS	AJ271736.10	+	18	27	9.07E-05	0.154	AGAGGAAGTC
1	ACCGGAAGNS	Z84812.4	+	18	27	9.07E-05	0.154	AGAGGAAGTC
1	ACCGGAAGNS	HNRNPUL1	+	130	139	9.07E-05	0.154	AGAGGAAGTC
1	ACCGGAAGNS	NDUFA7	+	31	40	9.25E-05	0.157	GACGGAAGAT
1	ACCGGAAGNS	RP11-384K6.6	-	5	14	9.49E-05	0.16	CACGGAAGCC
1	ACCGGAAGNS	RP11-206L10.2	-	24	33	9.49E-05	0.16	CACGGAAGGC
1	ACCGGAAGNS	IFT52	-	65	74	9.49E-05	0.16	CACGGAAGGC
1	ACCGGAAGNS	SMAD3	-	181	190	9.49E-05	0.16	CACGGAAGGC
1	ACCGGAAGNS	UPK3BP1	+	42	51	9.49E-05	0.16	CACGGAAGGC
1	ACCGGAAGNS	NHP2	-	124	133	9.60E-05	0.161	AAAGGAAGTC
1	ACCGGAAGNS	LINC00998	+	5	14	9.60E-05	0.161	AAAGGAAGTC
1	ACCGGAAGNS	ATP5E	-	146	155	9.79E-05	0.163	GCAGGAAGAT
1	ACCGGAAGNS	RP11-1060J15.4	+	83	92	9.79E-05	0.163	GCAGGAAGAT
1	ACCGGAAGNS	TCF3	+	108	117	9.79E-05	0.163	GCAGGAAGAT

Supplementary Table 10. FIMO motif scanning using JASPAR motif matrices using default p-value cutoff 1e-4 (NRF1)

NRF1 motif	Alt ID	Sequence Name	Strand	Start	End	p-value	q-value	Matched Sequence
1	GCGCVTGCGCV	HNRNPA1	+	2	12	1.13E-07	0.00218	GCGCCTGCGCA
1	GCGCVTGCGCV	EIF3K	+	4	14	1.13E-07	0.00218	GCGCCTGCGCA
1	GCGCVTGCGCV	PRDX5	+	5	15	1.13E-07	0.00218	GCGCCTGCGCA
1	GCGCVTGCGCV	RP1-101K10.6	+	28	38	1.13E-07	0.00218	GCGCCTGCGCA
1	GCGCVTGCGCV	PATL1	+	33	43	1.13E-07	0.00218	GCGCCTGCGCA
1	GCGCVTGCGCV	LSM2	+	42	52	1.13E-07	0.00218	GCGCCTGCGCA
1	GCGCVTGCGCV	TBC1D17	+	60	70	1.13E-07	0.00218	GCGCCTGCGCA
1	GCGCVTGCGCV	KDM2A	+	60	70	1.13E-07	0.00218	GCGCCTGCGCA
1	GCGCVTGCGCV	RP11-307E17.8	+	65	75	1.13E-07	0.00218	GCGCCTGCGCA
1	GCGCVTGCGCV	SARS	+	66	76	1.13E-07	0.00218	GCGCCTGCGCA
1	GCGCVTGCGCV	MINK1	+	69	79	1.13E-07	0.00218	GCGCCTGCGCA
1	GCGCVTGCGCV	EED	+	70	80	1.13E-07	0.00218	GCGCCTGCGCA
1	GCGCVTGCGCV	DYNLT1	+	77	87	1.13E-07	0.00218	GCGCCTGCGCA
1	GCGCVTGCGCV	DAD1	+	78	88	1.13E-07	0.00218	GCGCCTGCGCA
1	GCGCVTGCGCV	TMEM141	+	80	90	1.13E-07	0.00218	GCGCCTGCGCA
1	GCGCVTGCGCV	FUNDC1	+	84	94	1.13E-07	0.00218	GCGCCTGCGCA
1	GCGCVTGCGCV	TOMM7	+	90	100	1.13E-07	0.00218	GCGCCTGCGCA
1	GCGCVTGCGCV	RPL5	+	94	104	1.13E-07	0.00218	GCGCCTGCGCA
1	GCGCVTGCGCV	ZNF627	+	112	122	1.13E-07	0.00218	GCGCCTGCGCA
1	GCGCVTGCGCV	UQCR11	+	119	129	1.13E-07	0.00218	GCGCCTGCGCA
1	GCGCVTGCGCV	OMA1	+	149	159	1.13E-07	0.00218	GCGCCTGCGCA
1	GCGCVTGCGCV	BRK1	+	176	186	1.13E-07	0.00218	GCGCCTGCGCA
1	GCGCVTGCGCV	AC024560.3	-	1	11	1.13E-07	0.00218	GCGCCTGCGCA
1	GCGCVTGCGCV	RABGGTB	-	10	20	1.13E-07	0.00218	GCGCCTGCGCA
1	GCGCVTGCGCV	GTF2F2	-	52	62	1.13E-07	0.00218	GCGCCTGCGCA

1	GCGCVTGCGCV	AC024560.3	-	53	63	1.13E-07	0.00218	GCGCCTGCGCA
1	GCGCVTGCGCV	CNIH1	-	56	66	1.13E-07	0.00218	GCGCCTGCGCA
1	GCGCVTGCGCV	GTF2I	-	57	67	1.13E-07	0.00218	GCGCCTGCGCA
1	GCGCVTGCGCV	BCCIP	-	87	97	1.13E-07	0.00218	GCGCCTGCGCA
1	GCGCVTGCGCV	SLX4	-	90	100	1.13E-07	0.00218	GCGCCTGCGCA
1	GCGCVTGCGCV	ZNF483	-	98	108	1.13E-07	0.00218	GCGCCTGCGCA
1	GCGCVTGCGCV	AC024560.3	-	103	113	1.13E-07	0.00218	GCGCCTGCGCA
1	GCGCVTGCGCV	COPB2	-	108	118	1.13E-07	0.00218	GCGCCTGCGCA
1	GCGCVTGCGCV	RBX1	-	110	120	1.13E-07	0.00218	GCGCCTGCGCA
1	GCGCVTGCGCV	TM2D3	-	113	123	1.13E-07	0.00218	GCGCCTGCGCA
1	GCGCVTGCGCV	RPS15	-	124	134	1.13E-07	0.00218	GCGCCTGCGCA
1	GCGCVTGCGCV	PDRG1	-	127	137	1.13E-07	0.00218	GCGCCTGCGCA
1	GCGCVTGCGCV	NDUFA8	-	130	140	1.13E-07	0.00218	GCGCCTGCGCA
1	GCGCVTGCGCV	TCTE3	+	5	15	2.07E-07	0.00264	GCGCCTGCGCG
1	GCGCVTGCGCV	CANX	+	5	15	2.07E-07	0.00264	GCGCCTGCGCG
1	GCGCVTGCGCV	RP1-122P22.4	+	10	20	2.07E-07	0.00264	GCGCCTGCGCG
1	GCGCVTGCGCV	CSTF1	-	12	22	2.07E-07	0.00264	GCGCCTGCGCG
1	GCGCVTGCGCV	RPS23	+	27	37	2.07E-07	0.00264	GCGCCTGCGCG
1	GCGCVTGCGCV	UXS1	+	35	45	2.07E-07	0.00264	GCGCCTGCGCG
1	GCGCVTGCGCV	MRPL14	-	26	36	2.07E-07	0.00264	GCGCCTGCGCG
1	GCGCVTGCGCV	GMNN	-	32	42	2.07E-07	0.00264	GCGCCTGCGCG
1	GCGCVTGCGCV	NEIL2	+	44	54	2.07E-07	0.00264	GCGCCTGCGCG
1	GCGCVTGCGCV	SLX4	+	46	56	2.07E-07	0.00264	GCGCCTGCGCG
1	GCGCVTGCGCV	NDUFB4	-	64	74	2.07E-07	0.00264	GCGCCTGCGCG
1	GCGCVTGCGCV	FAM220A	-	84	94	2.07E-07	0.00264	GCGCCTGCGCG
1	GCGCVTGCGCV	JAGN1	+	95	105	2.07E-07	0.00264	GCGCCTGCGCG
1	GCGCVTGCGCV	PSAP	-	86	96	2.07E-07	0.00264	GCGCCTGCGCG

1	GCGCVTGCGCV	LRRC75A-AS1	-	96	106	2.07E-07	0.00264	GCGCCTGCGCG
1	GCGCVTGCGCV	TMEM106C	+	119	129	2.07E-07	0.00264	GCGCCTGCGCG
1	GCGCVTGCGCV	NDUFB4	-	119	129	2.07E-07	0.00264	GCGCCTGCGCG
1	GCGCVTGCGCV	UBE2T	-	125	135	2.07E-07	0.00264	GCGCCTGCGCG
1	GCGCVTGCGCV	WRB	-	162	172	2.07E-07	0.00264	GCGCCTGCGCG
1	GCGCVTGCGCV	MED18	-	11	21	3.45E-07	0.00314	GCGCATGCGCA
1	GCGCVTGCGCV	CHMP2A	-	13	23	3.45E-07	0.00314	GCGCATGCGCA
1	GCGCVTGCGCV	CKLF	-	14	24	3.45E-07	0.00314	GCGCATGCGCA
1	GCGCVTGCGCV	TEKT4P2	-	64	74	3.45E-07	0.00314	GCGCATGCGCA
1	GCGCVTGCGCV	MARS2	-	73	83	3.45E-07	0.00314	GCGCATGCGCA
1	GCGCVTGCGCV	MRPL17	-	80	90	3.45E-07	0.00314	GCGCATGCGCA
1	GCGCVTGCGCV	UROS	-	88	98	3.45E-07	0.00314	GCGCATGCGCA
1	GCGCVTGCGCV	PSMA4	-	122	132	3.45E-07	0.00314	GCGCATGCGCA
1	GCGCVTGCGCV	NDUFA8	-	124	134	3.45E-07	0.00314	GCGCATGCGCA
1	GCGCVTGCGCV	DAD1	-	128	138	3.45E-07	0.00314	GCGCATGCGCA
1	GCGCVTGCGCV	ELP6	-	155	165	3.45E-07	0.00314	GCGCATGCGCA
1	GCGCVTGCGCV	TIMM8B	+	27	37	3.45E-07	0.00314	GCGCATGCGCA
1	GCGCVTGCGCV	GTF2IP1	+	53	63	3.45E-07	0.00314	GCGCATGCGCA
1	GCGCVTGCGCV	GTF2IP4	+	53	63	3.45E-07	0.00314	GCGCATGCGCA
1	GCGCVTGCGCV	GATS	+	56	66	3.45E-07	0.00314	GCGCATGCGCA
1	GCGCVTGCGCV	RPL10A	+	74	84	3.45E-07	0.00314	GCGCATGCGCA
1	GCGCVTGCGCV	NNAT	+	78	88	3.45E-07	0.00314	GCGCATGCGCA
1	GCGCVTGCGCV	MRPL17	+	81	91	3.45E-07	0.00314	GCGCATGCGCA
1	GCGCVTGCGCV	SLX4	+	85	95	3.45E-07	0.00314	GCGCATGCGCA
1	GCGCVTGCGCV	USO1	+	98	108	3.45E-07	0.00314	GCGCATGCGCA
1	GCGCVTGCGCV	NDUFA8	+	125	135	3.45E-07	0.00314	GCGCATGCGCA
1	GCGCVTGCGCV	DAD1	+	129	139	3.45E-07	0.00314	GCGCATGCGCA

1	GCGCVTGCGCV	NDUFAF5	+	138	148	3.45E-07	0.00314	GCGCATGCGCA
1	GCGCVTGCGCV	FAM220A	-	15	25	4.58E-07	0.00334	GCGCATGCGCG
1	GCGCVTGCGCV	TIMM8B	-	26	36	4.58E-07	0.00334	GCGCATGCGCG
1	GCGCVTGCGCV	GTF2IP4	-	52	62	4.58E-07	0.00334	GCGCATGCGCG
1	GCGCVTGCGCV	GTF2IP1	-	52	62	4.58E-07	0.00334	GCGCATGCGCG
1	GCGCVTGCGCV	GATS	-	55	65	4.58E-07	0.00334	GCGCATGCGCG
1	GCGCVTGCGCV	NNAT	-	77	87	4.58E-07	0.00334	GCGCATGCGCG
1	GCGCVTGCGCV	ORAOV1	-	85	95	4.58E-07	0.00334	GCGCATGCGCG
1	GCGCVTGCGCV	USO1	-	97	107	4.58E-07	0.00334	GCGCATGCGCG
1	GCGCVTGCGCV	MSL3P1	-	110	120	4.58E-07	0.00334	GCGCATGCGCG
1	GCGCVTGCGCV	ALG6	-	137	147	4.58E-07	0.00334	GCGCATGCGCG
1	GCGCVTGCGCV	RAN	+	3	13	4.58E-07	0.00334	GCGCATGCGCG
1	GCGCVTGCGCV	MAGI1	+	13	23	4.58E-07	0.00334	GCGCATGCGCG
1	GCGCVTGCGCV	CKLF	+	15	25	4.58E-07	0.00334	GCGCATGCGCG
1	GCGCVTGCGCV	FAM220A	+	16	26	4.58E-07	0.00334	GCGCATGCGCG
1	GCGCVTGCGCV	TMEM14A	+	54	64	4.58E-07	0.00334	GCGCATGCGCG
1	GCGCVTGCGCV	UROS	+	89	99	4.58E-07	0.00334	GCGCATGCGCG
1	GCGCVTGCGCV	CASZ1	+	99	109	4.58E-07	0.00334	GCGCATGCGCG
1	GCGCVTGCGCV	PSMA4	+	123	133	4.58E-07	0.00334	GCGCATGCGCG
1	GCGCVTGCGCV	ATP5C1	+	127	137	4.58E-07	0.00334	GCGCATGCGCG
1	GCGCVTGCGCV	ELP6	+	156	166	4.58E-07	0.00334	GCGCATGCGCG
1	GCGCVTGCGCV	MMADHC	+	1	11	5.71E-07	0.00359	GCGCGTGCGCA
1	GCGCVTGCGCV	HSDL1	-	14	24	5.71E-07	0.00359	GCGCGTGCGCA
1	GCGCVTGCGCV	EZH1	-	39	49	5.71E-07	0.00359	GCGCGTGCGCA
1	GCGCVTGCGCV	DIP2A	-	45	55	5.71E-07	0.00359	GCGCGTGCGCA
1	GCGCVTGCGCV	TMEM263	+	56	66	5.71E-07	0.00359	GCGCGTGCGCA
1	GCGCVTGCGCV	NDUFA12	-	57	67	5.71E-07	0.00359	GCGCGTGCGCA

1	GCGCVTGCG CV	SNRNP70	+	76	86	5.71E-07	0.00359	GCGCGTGCGCA
1	GCGCVTGCG CV	EIF3K	-	69	79	5.71E-07	0.00359	GCGCGTGCGCA
1	GCGCVTGCG CV	TMEM14B	-	74	84	5.71E-07	0.00359	GCGCGTGCGCA
1	GCGCVTGCG CV	ELP6	+	88	98	5.71E-07	0.00359	GCGCGTGCGCA
1	GCGCVTGCG CV	BNIP3	-	83	93	5.71E-07	0.00359	GCGCGTGCGCA
1	GCGCVTGCG CV	SNHG3	-	87	97	5.71E-07	0.00359	GCGCGTGCGCA
1	GCGCVTGCG CV	N6AMT2	-	123	133	5.71E-07	0.00359	GCGCGTGCGCA
1	GCGCVTGCG CV	PDRG1	+	159	169	5.71E-07	0.00359	GCGCGTGCGCA
1	GCGCVTGCG CV	PTGES3	-	167	177	5.71E-07	0.00359	GCGCGTGCGCA
1	GCGCVTGCG CV	VDAC3	-	168	178	5.71E-07	0.00359	GCGCGTGCGCA
1	GCGCVTGCG CV	CYTH1	-	30	40	6.65E-07	0.00381	GCGCCTGCGCC
1	GCGCVTGCG CV	SKP1	+	42	52	6.65E-07	0.00381	GCGCCTGCGCC
1	GCGCVTGCG CV	CYTH1	-	36	46	6.65E-07	0.00381	GCGCCTGCGCC
1	GCGCVTGCG CV	SAP18	+	58	68	6.65E-07	0.00381	GCGCCTGCGCC
1	GCGCVTGCG CV	FAM195B	+	80	90	6.65E-07	0.00381	GCGCCTGCGCC
1	GCGCVTGCG CV	GTF2H5	-	71	81	6.65E-07	0.00381	GCGCCTGCGCC
1	GCGCVTGCG CV	CHAC2	+	88	98	6.65E-07	0.00381	GCGCCTGCGCC
1	GCGCVTGCG CV	NBPF9	+	88	98	6.65E-07	0.00381	GCGCCTGCGCC
1	GCGCVTGCG CV	NBPF15	+	93	103	6.65E-07	0.00381	GCGCCTGCGCC
1	GCGCVTGCG CV	STIM2	-	125	135	6.65E-07	0.00381	GCGCCTGCGCC
1	GCGCVTGCG CV	KDM3A	+	180	190	6.65E-07	0.00381	GCGCCTGCGCC
1	GCGCVTGCG CV	DYNLL1	+	7	17	7.58E-07	0.00381	GCGCGTGCGCG
1	GCGCVTGCG CV	PCNT	+	20	30	7.58E-07	0.00381	GCGCGTGCGCG
1	GCGCVTGCG CV	CHERP	+	23	33	7.58E-07	0.00381	GCGCGTGCGCG
1	GCGCVTGCG CV	ZXDC	-	18	28	7.58E-07	0.00381	GCGCGTGCGCG
1	GCGCVTGCG CV	CLPTM1L	+	45	55	7.58E-07	0.00381	GCGCGTGCGCG
1	GCGCVTGCG CV	CTSC	-	43	53	7.58E-07	0.00381	GCGCGTGCGCG

1	GCGCVTGCGCV	BNIP3	-	44	54	7.58E-07	0.00381	GCGCGTGCGCG
1	GCGCVTGCGCV	RIOK2	+	58	68	7.58E-07	0.00381	GCGCGTGCGCG
1	GCGCVTGCGCV	TMEM135	-	64	74	7.58E-07	0.00381	GCGCGTGCGCG
1	GCGCVTGCGCV	NIPA2	+	77	87	7.58E-07	0.00381	GCGCGTGCGCG
1	GCGCVTGCGCV	HMGN1P20	-	71	81	7.58E-07	0.00381	GCGCGTGCGCG
1	GCGCVTGCGCV	RALGAPB	-	71	81	7.58E-07	0.00381	GCGCGTGCGCG
1	GCGCVTGCGCV	DIP2A	-	78	88	7.58E-07	0.00381	GCGCGTGCGCG
1	GCGCVTGCGCV	OSTC	-	85	95	7.58E-07	0.00381	GCGCGTGCGCG
1	GCGCVTGCGCV	UQCR11	+	100	110	7.58E-07	0.00381	GCGCGTGCGCG
1	GCGCVTGCGCV	AKAP1	-	122	132	7.58E-07	0.00381	GCGCGTGCGCG
1	GCGCVTGCGCV	TMEM263	+	168	178	7.58E-07	0.00381	GCGCGTGCGCG
1	GCGCVTGCGCV	RAN	-	171	181	7.58E-07	0.00381	GCGCGTGCGCG
1	GCGCVTGCGCV	MED18	+	12	22	8.71E-07	0.00404	GCGCATGCGCC
1	GCGCVTGCGCV	MAGI1	-	12	22	8.71E-07	0.00404	GCGCATGCGCC
1	GCGCVTGCGCV	TEKT4P2	+	65	75	8.71E-07	0.00404	GCGCATGCGCC
1	GCGCVTGCGCV	MARS2	+	74	84	8.71E-07	0.00404	GCGCATGCGCC
1	GCGCVTGCGCV	ORAOV1	+	86	96	8.71E-07	0.00404	GCGCATGCGCC
1	GCGCVTGCGCV	SLX4	-	84	94	8.71E-07	0.00404	GCGCATGCGCC
1	GCGCVTGCGCV	MSL3P1	+	111	121	8.71E-07	0.00404	GCGCATGCGCC
1	GCGCVTGCGCV	ATP5C1	-	126	136	8.71E-07	0.00404	GCGCATGCGCC
1	GCGCVTGCGCV	ALG6	+	138	148	8.71E-07	0.00404	GCGCATGCGCC
1	GCGCVTGCGCV	NDUFAF5	-	137	147	8.71E-07	0.00404	GCGCATGCGCC
1	GCGCVTGCGCV	GEMIN2	+	180	190	8.71E-07	0.00404	GCGCATGCGCC
1	GCGCVTGCGCV	GEMIN2	-	179	189	8.71E-07	0.00404	GCGCATGCGCC
1	GCGCVTGCGCV	CHMP2A	-	51	61	9.64E-07	0.00439	GCGCGTGCGCC
1	GCGCVTGCGCV	TMEM38B	-	87	97	9.64E-07	0.00439	GCGCGTGCGCC
1	GCGCVTGCGCV	TARS	-	111	121	9.64E-07	0.00439	GCGCGTGCGCC

1	GCGCVTGCGCV	TCEB2	-	44	54	1.06E-06	0.0047	GCGCCGGCGCA
1	GCGCVTGCGCV	LUC7L	-	70	80	1.06E-06	0.0047	GCGCCGGCGCA
1	GCGCVTGCGCV	SARS	+	93	103	1.06E-06	0.0047	GCGCCGGCGCA
1	GCGCVTGCGCV	CCDC84	+	160	170	1.06E-06	0.0047	GCGCCGGCGCA
1	GCGCVTGCGCV	NDUFB2-AS1	-	30	40	1.13E-06	0.00483	GCGCCGGCGCG
1	GCGCVTGCGCV	TCEB2	+	45	55	1.13E-06	0.00483	GCGCCGGCGCG
1	GCGCVTGCGCV	LSM4	+	69	79	1.13E-06	0.00483	GCGCCGGCGCG
1	GCGCVTGCGCV	LSM4	-	68	78	1.13E-06	0.00483	GCGCCGGCGCG
1	GCGCVTGCGCV	MTX2	-	71	81	1.13E-06	0.00483	GCGCCGGCGCG
1	GCGCVTGCGCV	SARS	-	92	102	1.13E-06	0.00483	GCGCCGGCGCG
1	GCGCVTGCGCV	AC011747.4	+	115	125	1.13E-06	0.00483	GCGCCGGCGCG
1	GCGCVTGCGCV	CANX	+	13	23	1.27E-06	0.00524	GCGCTTGCGCA
1	GCGCVTGCGCV	COMMD8	-	6	16	1.27E-06	0.00524	GCGCTTGCGCA
1	GCGCVTGCGCV	FNBP4	+	87	97	1.27E-06	0.00524	GCGCTTGCGCA
1	GCGCVTGCGCV	FNBP4	-	92	102	1.27E-06	0.00524	GCGCTTGCGCA
1	GCGCVTGCGCV	UBA52	-	181	191	1.27E-06	0.00524	GCGCTTGCGCA
1	GCGCVTGCGCV	ZNF865	-	191	201	1.27E-06	0.00524	GCGCTTGCGCA
1	GCGCVTGCGCV	TMEM87A	+	70	80	1.41E-06	0.00577	ACGCCTGCGCA
1	GCGCVTGCGCV	PDRG1	+	100	110	1.64E-06	0.00661	GAGCCTGCGCA
1	GCGCVTGCGCV	FAM3D	+	132	142	1.64E-06	0.00661	GAGCCTGCGCA
1	GCGCVTGCGCV	NDUFB4	+	178	188	1.64E-06	0.00661	GAGCCTGCGCA
1	GCGCVTGCGCV	MLH1	-	65	75	1.75E-06	0.00697	GCGCTTGCGCG
1	GCGCVTGCGCV	COPB1	-	80	90	1.75E-06	0.00697	GCGCTTGCGCG
1	GCGCVTGCGCV	C1GALT1C1	+	94	104	1.87E-06	0.00697	ACGCCTGCGCG
1	GCGCVTGCGCV	NSF	+	110	120	1.87E-06	0.00697	ACGCCTGCGCG
1	GCGCVTGCGCV	HNRNPA1	-	1	11	2.10E-06	0.00697	GCGCAGGCGCA
1	GCGCVTGCGCV	CSTF1	+	13	23	2.10E-06	0.00697	GCGCAGGCGCA

1	GCGCVTGCGCV	CCDC84	-	5	15	2.10E-06	0.00697	GCGGCTGCGCA
1	GCGCVTGCGCV	CYTH1	+	31	41	2.10E-06	0.00697	GCGCAGGCGCA
1	GCGCVTGCGCV	GMNN	+	33	43	2.10E-06	0.00697	GCGCAGGCGCA
1	GCGCVTGCGCV	IQSEC3	-	29	39	2.10E-06	0.00697	GCGGCTGCGCA
1	GCGCVTGCGCV	GTF2F2	+	53	63	2.10E-06	0.00697	GCGCAGGCGCA
1	GCGCVTGCGCV	NEIL2	-	43	53	2.10E-06	0.00697	GCGCAGGCGCA
1	GCGCVTGCGCV	AC024560.3	+	54	64	2.10E-06	0.00697	GCGCAGGCGCA
1	GCGCVTGCGCV	SLX4	-	45	55	2.10E-06	0.00697	GCGCAGGCGCA
1	GCGCVTGCGCV	GTF2I	+	58	68	2.10E-06	0.00697	GCGCAGGCGCA
1	GCGCVTGCGCV	NDUFB4	+	65	75	2.10E-06	0.00697	GCGCAGGCGCA
1	GCGCVTGCGCV	SAP18	-	57	67	2.10E-06	0.00697	GCGCAGGCGCA
1	GCGCVTGCGCV	TBC1D17	-	59	69	2.10E-06	0.00697	GCGCAGGCGCA
1	GCGCVTGCGCV	GTF2H5	+	72	82	2.10E-06	0.00697	GCGCAGGCGCA
1	GCGCVTGCGCV	N6AMT2	-	63	73	2.10E-06	0.00697	GCGCAGGCGCA
1	GCGCVTGCGCV	SARS	-	65	75	2.10E-06	0.00697	GCGCAGGCGCA
1	GCGCVTGCGCV	STX1A	-	66	76	2.10E-06	0.00697	GCGGCTGCGCA
1	GCGCVTGCGCV	DYNLT1	-	76	86	2.10E-06	0.00697	GCGCAGGCGCA
1	GCGCVTGCGCV	ATP6V1G1	+	86	96	2.10E-06	0.00697	GCGCAGGCGCA
1	GCGCVTGCGCV	BCCIP	+	88	98	2.10E-06	0.00697	GCGCAGGCGCA
1	GCGCVTGCGCV	TMEM141	-	79	89	2.10E-06	0.00697	GCGCAGGCGCA
1	GCGCVTGCGCV	FAM195B	-	79	89	2.10E-06	0.00697	GCGCAGGCGCA
1	GCGCVTGCGCV	NBPF9	-	87	97	2.10E-06	0.00697	GCGCAGGCGCA
1	GCGCVTGCGCV	ZNF483	+	99	109	2.10E-06	0.00697	GCGCAGGCGCA
1	GCGCVTGCGCV	NBPF15	-	92	102	2.10E-06	0.00697	GCGCAGGCGCA
1	GCGCVTGCGCV	RPL5	-	93	103	2.10E-06	0.00697	GCGCAGGCGCA
1	GCGCVTGCGCV	AC024560.3	+	104	114	2.10E-06	0.00697	GCGCAGGCGCA
1	GCGCVTGCGCV	ZNF865	+	119	129	2.10E-06	0.00697	GCGCAGGCGCA

1	GCGCVTGCGCV	NDUFB4	+	120	130	2.10E-06	0.00697	GCGCAGGCGCA
1	GCGCVTGCGCV	ZNF627	-	111	121	2.10E-06	0.00697	GCGCAGGCGCA
1	GCGCVTGCGCV	TMED9	+	132	142	2.10E-06	0.00697	GCGGCTGCGCA
1	GCGCVTGCGCV	BRK1	+	144	154	2.10E-06	0.00697	GCGCAGGCGCA
1	GCGCVTGCGCV	KDM3A	-	179	189	2.10E-06	0.00697	GCGCAGGCGCA
1	GCGCVTGCGCV	C19orf70	-	18	28	2.17E-06	0.0071	GCGCCCGCGCG
1	GCGCVTGCGCV	C6orf48	+	52	62	2.17E-06	0.0071	GCGCCCGCGCG
1	GCGCVTGCGCV	GJB2	+	75	85	2.17E-06	0.0071	GCGCCCGCGCG
1	GCGCVTGCGCV	FAM63B	+	149	159	2.17E-06	0.0071	GCGCCCGCGCG
1	GCGCVTGCGCV	MFSD1	+	45	55	2.28E-06	0.00736	GAGCCTGCGCG
1	GCGCVTGCGCV	RP11-46F15.2	-	36	46	2.28E-06	0.00736	GAGCCTGCGCG
1	GCGCVTGCGCV	ZBTB4	+	83	93	2.40E-06	0.00736	CCGCCTGCGCA
1	GCGCVTGCGCV	AC024560.3	+	2	12	2.58E-06	0.00736	GCGCAGGCGCG
1	GCGCVTGCGCV	PRDX5	-	4	14	2.58E-06	0.00736	GCGCAGGCGCG
1	GCGCVTGCGCV	HIST1H1C	-	12	22	2.58E-06	0.00736	GCGCAGGCGCG
1	GCGCVTGCGCV	CYTH1	+	37	47	2.58E-06	0.00736	GCGCAGGCGCG
1	GCGCVTGCGCV	PATL1	-	32	42	2.58E-06	0.00736	GCGCAGGCGCG
1	GCGCVTGCGCV	UXS1	-	34	44	2.58E-06	0.00736	GCGCAGGCGCG
1	GCGCVTGCGCV	KLHL9	-	37	47	2.58E-06	0.00736	GCGGCTGCGCG
1	GCGCVTGCGCV	LSM2	-	41	51	2.58E-06	0.00736	GCGCAGGCGCG
1	GCGCVTGCGCV	CNIH1	+	57	67	2.58E-06	0.00736	GCGCAGGCGCG
1	GCGCVTGCGCV	FOXP2	+	73	83	2.58E-06	0.00736	GCGGCTGCGCG
1	GCGCVTGCGCV	RP11-307E17.8	-	64	74	2.58E-06	0.00736	GCGCAGGCGCG
1	GCGCVTGCGCV	ULK1	+	75	85	2.58E-06	0.00736	GCGGCTGCGCG
1	GCGCVTGCGCV	MINK1	-	68	78	2.58E-06	0.00736	GCGCAGGCGCG
1	GCGCVTGCGCV	LDHAL6B	-	72	82	2.58E-06	0.00736	GCGGCTGCGCG
1	GCGCVTGCGCV	R3HDM2	-	72	82	2.58E-06	0.00736	GCGGCTGCGCG

1	GCGCVTGCGCV	FAM220A	+	85	95	2.58E-06	0.00736	GCGCAGGCGCG
1	GCGCVTGCGCV	SLC12A6	+	89	99	2.58E-06	0.00736	GCGCAGGCGCG
1	GCGCVTGCGCV	SLX4	+	91	101	2.58E-06	0.00736	GCGCAGGCGCG
1	GCGCVTGCGCV	UQCR10	+	93	103	2.58E-06	0.00736	GCGCAGGCGCG
1	GCGCVTGCGCV	FUNDC1	-	83	93	2.58E-06	0.00736	GCGCAGGCGCG
1	GCGCVTGCGCV	CHAC2	-	87	97	2.58E-06	0.00736	GCGCAGGCGCG
1	GCGCVTGCGCV	RTCB	-	94	104	2.58E-06	0.00736	GCGCAGGCGCG
1	GCGCVTGCGCV	NEO1	+	106	116	2.58E-06	0.00736	GCGGCTGCGCG
1	GCGCVTGCGCV	CDC42BPG	+	109	119	2.58E-06	0.00736	GCGGCTGCGCG
1	GCGCVTGCGCV	RBX1	+	111	121	2.58E-06	0.00736	GCGCAGGCGCG
1	GCGCVTGCGCV	RPS15	+	125	135	2.58E-06	0.00736	GCGCAGGCGCG
1	GCGCVTGCGCV	UQCR11	-	118	128	2.58E-06	0.00736	GCGCAGGCGCG
1	GCGCVTGCGCV	PDRG1	+	128	138	2.58E-06	0.00736	GCGCAGGCGCG
1	GCGCVTGCGCV	OMA1	-	148	158	2.58E-06	0.00736	GCGCAGGCGCG
1	GCGCVTGCGCV	WRB	+	163	173	2.58E-06	0.00736	GCGCAGGCGCG
1	GCGCVTGCGCV	HELQ	+	1	11	2.75E-06	0.00751	ACGCATGCGCA
1	GCGCVTGCGCV	ZNF576	+	13	23	2.75E-06	0.00751	ACGCATGCGCA
1	GCGCVTGCGCV	MRPL36	+	30	40	2.75E-06	0.00751	ACGCATGCGCA
1	GCGCVTGCGCV	DYRK1B	-	23	33	2.75E-06	0.00751	ACGCATGCGCA
1	GCGCVTGCGCV	DHX9	-	31	41	2.75E-06	0.00751	ACGCATGCGCA
1	GCGCVTGCGCV	HNRNPA1	+	50	60	2.75E-06	0.00751	ACGCATGCGCA
1	GCGCVTGCGCV	MRPS18C	-	65	75	2.75E-06	0.00751	ACGCATGCGCA
1	GCGCVTGCGCV	DISP2	+	81	91	2.75E-06	0.00751	ACGCATGCGCA
1	GCGCVTGCGCV	SPOP	+	84	94	2.75E-06	0.00751	ACGCATGCGCA
1	GCGCVTGCGCV	MRPL22	-	79	89	2.75E-06	0.00751	ACGCATGCGCA
1	GCGCVTGCGCV	SRSF7	+	117	127	2.75E-06	0.00751	ACGCATGCGCA
1	GCGCVTGCGCV	HSDL1	+	15	25	2.96E-06	0.00762	GCGCACGCGCA

1	GCGCVTGCGCV	DYNLL1	-	6	16	2.96E-06	0.00762	GCGCACGCGCA
1	GCGCVTGCGCV	CHERP	-	22	32	2.96E-06	0.00762	GCGCACGCGCA
1	GCGCVTGCGCV	SNHG1	-	44	54	2.96E-06	0.00762	GCGCACGCGCA
1	GCGCVTGCGCV	CLPTM1L	-	44	54	2.96E-06	0.00762	GCGCACGCGCA
1	GCGCVTGCGCV	AFF1	+	58	68	2.96E-06	0.00762	CCGCCTGCGCG
1	GCGCVTGCGCV	NDUFA12	+	58	68	2.96E-06	0.00762	GCGCACGCGCA
1	GCGCVTGCGCV	DYRK1B	-	52	62	2.96E-06	0.00762	CCGCCTGCGCG
1	GCGCVTGCGCV	RALGAPB	+	72	82	2.96E-06	0.00762	GCGCACGCGCA
1	GCGCVTGCGCV	BNIP3	+	84	94	2.96E-06	0.00762	GCGCACGCGCA
1	GCGCVTGCGCV	OSTC	+	86	96	2.96E-06	0.00762	GCGCACGCGCA
1	GCGCVTGCGCV	NIPA2	-	76	86	2.96E-06	0.00762	GCGCACGCGCA
1	GCGCVTGCGCV	SNHG3	+	88	98	2.96E-06	0.00762	GCGCACGCGCA
1	GCGCVTGCGCV	TARS	+	112	122	2.96E-06	0.00762	GCGCACGCGCA
1	GCGCVTGCGCV	AKAP1	+	123	133	2.96E-06	0.00762	GCGCACGCGCA
1	GCGCVTGCGCV	GRPEL2	-	176	186	2.96E-06	0.00762	GCGCACGCGCA
1	GCGCVTGCGCV	NATD1	-	159	169	3.05E-06	0.00781	GCGCGGGCGCA
1	GCGCVTGCGCV	NCOR2	-	170	180	3.05E-06	0.00781	GCGCGGGCGCA
1	GCGCVTGCGCV	NDUFA6	+	16	26	3.22E-06	0.00809	GAGCATGCGCA
1	GCGCVTGCGCV	ATP6V1F	-	73	83	3.22E-06	0.00809	GAGCATGCGCA
1	GCGCVTGCGCV	DOHH	-	83	93	3.22E-06	0.00809	GAGCATGCGCA
1	GCGCVTGCGCV	CCDC167	+	95	105	3.22E-06	0.00809	GAGCATGCGCA
1	GCGCVTGCGCV	NDUFAF4	-	96	106	3.22E-06	0.00809	GAGCATGCGCA
1	GCGCVTGCGCV	LRCH1	+	183	193	3.33E-06	0.00835	GCGCCTGCCCA
1	GCGCVTGCGCV	TMEM30A	+	7	17	3.47E-06	0.00841	ACGCATGCGCG
1	GCGCVTGCGCV	EZH1	-	96	106	3.47E-06	0.00841	ACGCATGCGCG
1	GCGCVTGCGCV	AC007191.4	-	167	177	3.47E-06	0.00841	ACGCATGCGCG
1	GCGCVTGCGCV	GTF2I	+	52	62	3.61E-06	0.00841	GCGGATGCGCA

1	GCGCVTGCGCV	ZXDC	+	19	29	3.70E-06	0.00841	GCGCACGCGCG
1	GCGCVTGCGCV	SNRNP70	-	11	21	3.70E-06	0.00841	GCGCACGCGCG
1	GCGCVTGCGCV	PCNT	-	19	29	3.70E-06	0.00841	GCGCACGCGCG
1	GCGCVTGCGCV	DIP2A	+	46	56	3.70E-06	0.00841	GCGCACGCGCG
1	GCGCVTGCGCV	CHMP2A	+	52	62	3.70E-06	0.00841	GCGCACGCGCG
1	GCGCVTGCGCV	TMEM263	-	55	65	3.70E-06	0.00841	GCGCACGCGCG
1	GCGCVTGCGCV	TMEM106C	+	70	80	3.70E-06	0.00841	GCGCACGCGCG
1	GCGCVTGCGCV	TMEM38B	+	88	98	3.70E-06	0.00841	GCGCACGCGCG
1	GCGCVTGCGCV	ELP6	-	87	97	3.70E-06	0.00841	GCGCACGCGCG
1	GCGCVTGCGCV	UQCR11	-	99	109	3.70E-06	0.00841	GCGCACGCGCG
1	GCGCVTGCGCV	N6AMT2	+	124	134	3.70E-06	0.00841	GCGCACGCGCG
1	GCGCVTGCGCV	PTGES3	+	168	178	3.70E-06	0.00841	GCGCACGCGCG
1	GCGCVTGCGCV	VDAC3	+	169	179	3.70E-06	0.00841	GCGCACGCGCG
1	GCGCVTGCGCV	RAN	+	172	182	3.70E-06	0.00841	GCGCACGCGCG
1	GCGCVTGCGCV	TMEM263	-	167	177	3.70E-06	0.00841	GCGCACGCGCG
1	GCGCVTGCGCV	NDUFB2-AS1	+	31	41	3.78E-06	0.00841	GCGCCGGCGCC
1	GCGCVTGCGCV	BMS1P1	+	45	55	3.78E-06	0.00841	GCGCCGGCGCC
1	GCGCVTGCGCV	BMS1P1	-	44	54	3.78E-06	0.00841	GCGCCGGCGCC
1	GCGCVTGCGCV	LUC7L	+	71	81	3.78E-06	0.00841	GCGCCGGCGCC
1	GCGCVTGCGCV	MTX2	+	72	82	3.78E-06	0.00841	GCGCCGGCGCC
1	GCGCVTGCGCV	AC011747.4	-	114	124	3.78E-06	0.00841	GCGCCGGCGCC
1	GCGCVTGCGCV	GLUD1P3	+	138	148	3.78E-06	0.00841	GCGCCGGCGCC
1	GCGCVTGCGCV	GLUD1P3	-	137	147	3.78E-06	0.00841	GCGCCGGCGCC
1	GCGCVTGCGCV	XPOT	+	162	172	3.78E-06	0.00841	GCGCCGGCGCC
1	GCGCVTGCGCV	CCDC84	-	159	169	3.78E-06	0.00841	GCGCCGGCGCC
1	GCGCVTGCGCV	XPOT	-	161	171	3.78E-06	0.00841	GCGCCGGCGCC
1	GCGCVTGCGCV	AGAP1	+	1	11	3.86E-06	0.00841	GCGCGGGCGCG

1	GCGCVTGCGCV	AGAP1	+	7	17	3.86E-06	0.00841	GCGCGGGCGCG
1	GCGCVTGCGCV	C19orf70	+	19	29	3.86E-06	0.00841	GCGCGGGCGCG
1	GCGCVTGCGCV	C6orf48	-	51	61	3.86E-06	0.00841	GCGCGGGCGCG
1	GCGCVTGCGCV	NRBP2	-	85	95	3.86E-06	0.00841	GCGCGGGCGCG
1	GCGCVTGCGCV	PXN	+	106	116	3.86E-06	0.00841	GCGCGGGCGCG
1	GCGCVTGCGCV	WDR88	+	107	117	3.86E-06	0.00841	GCGCGGGCGCG
1	GCGCVTGCGCV	PXN	+	112	122	3.86E-06	0.00841	GCGCGGGCGCG
1	GCGCVTGCGCV	WDR88	+	113	123	3.86E-06	0.00841	GCGCGGGCGCG
1	GCGCVTGCGCV	KMT2C	-	109	119	3.86E-06	0.00841	GCGCGGGCGCG
1	GCGCVTGCGCV	FAM63B	-	148	158	3.86E-06	0.00841	GCGCGGGCGCG
1	GCGCVTGCGCV	TCF20	-	156	166	3.86E-06	0.00841	GCGCGGGCGCG
1	GCGCVTGCGCV	C17orf58	-	184	194	3.86E-06	0.00841	GCGCGGGCGCG
1	GCGCVTGCGCV	SFT2D1	+	55	65	3.99E-06	0.00866	GAGCATGCGCG
1	GCGCVTGCGCV	RPS10	-	143	153	3.99E-06	0.00866	GAGCATGCGCG
1	GCGCVTGCGCV	GOLT1A	-	34	44	4.09E-06	0.00881	GCGCCTGCCCG
1	GCGCVTGCGCV	C14orf166	+	159	169	4.09E-06	0.00881	GCGCCTGCCCG
1	GCGCVTGCGCV	TMEM116	-	1	11	4.36E-06	0.00933	ACGCGTGCGCA
1	GCGCVTGCGCV	ERP29	+	60	70	4.36E-06	0.00933	ACGCGTGCGCA
1	GCGCVTGCGCV	SYPL1	+	80	90	4.36E-06	0.00933	ACGCGTGCGCA
1	GCGCVTGCGCV	TMEM116	-	81	91	4.57E-06	0.00963	GCGCGCGCGCA
1	GCGCVTGCGCV	SSTR2	-	122	132	4.57E-06	0.00963	GCGCGCGCGCA
1	GCGCVTGCGCV	MBD6	-	147	157	4.57E-06	0.00963	GCGCGCGCGCA
1	GCGCVTGCGCV	DHX15	+	77	87	4.57E-06	0.00963	GCGCGCGCGCA
1	GCGCVTGCGCV	TMEM116	+	82	92	4.57E-06	0.00963	GCGCGCGCGCA
1	GCGCVTGCGCV	NDUFA8	-	68	78	4.71E-06	0.00986	GAGCGTGCGCA
1	GCGCVTGCGCV	ATG10	-	103	113	4.71E-06	0.00986	GAGCGTGCGCA
1	GCGCVTGCGCV	PSMB4	-	116	126	4.82E-06	0.0101	GCGCTTGCGCC

1	GCGCVTGCGCV	ATP6V1F	-	3	13	5.05E-06	0.0101	ACGCCTGCGCC
1	GCGCVTGCGCV	STX1A	-	88	98	5.05E-06	0.0101	CCGCATGCGCG
1	GCGCVTGCGCV	ALG8	+	70	80	5.05E-06	0.0101	CCGCATGCGCG
1	GCGCVTGCGCV	ALG8	+	113	123	5.05E-06	0.0101	CCGCATGCGCG
1	GCGCVTGCGCV	METTL5	+	103	113	5.16E-06	0.0101	ACGCGTGCGCG
1	GCGCVTGCGCV	AGAP1	-	6	16	5.35E-06	0.0101	GCGCCCGCGCC
1	GCGCVTGCGCV	PXN	-	105	115	5.35E-06	0.0101	GCGCCCGCGCC
1	GCGCVTGCGCV	WDR88	-	106	116	5.35E-06	0.0101	GCGCCCGCGCC
1	GCGCVTGCGCV	PXN	-	111	121	5.35E-06	0.0101	GCGCCCGCGCC
1	GCGCVTGCGCV	WDR88	-	112	122	5.35E-06	0.0101	GCGCCCGCGCC
1	GCGCVTGCGCV	TBC1D2B	-	123	133	5.35E-06	0.0101	GCGCCCGCGCC
1	GCGCVTGCGCV	RP11-700A24.1	+	56	66	5.35E-06	0.0101	GCGCCCGCGCC
1	GCGCVTGCGCV	HSD17B10	+	84	94	5.35E-06	0.0101	GCGCCCGCGCC
1	GCGCVTGCGCV	NRBP2	+	86	96	5.35E-06	0.0101	GCGCCCGCGCC
1	GCGCVTGCGCV	KMT2C	+	110	120	5.35E-06	0.0101	GCGCCCGCGCC
1	GCGCVTGCGCV	FOXK1	+	126	136	5.35E-06	0.0101	GCGCCCGCGCC
1	GCGCVTGCGCV	TCF20	+	157	167	5.35E-06	0.0101	GCGCCCGCGCC
1	GCGCVTGCGCV	NCOR2	+	171	181	5.35E-06	0.0101	GCGCCCGCGCC
1	GCGCVTGCGCV	C17orf58	+	185	195	5.35E-06	0.0101	GCGCCCGCGCC
1	GCGCVTGCGCV	PANK1	-	21	31	5.43E-06	0.0101	GCGCGCGCGCG
1	GCGCVTGCGCV	NSF	-	39	49	5.43E-06	0.0101	GCGCGCGCGCG
1	GCGCVTGCGCV	PRR12	-	48	58	5.43E-06	0.0101	GCGCGCGCGCG
1	GCGCVTGCGCV	C6orf48	-	57	67	5.43E-06	0.0101	GCGCGCGCGCG
1	GCGCVTGCGCV	DHX15	-	74	84	5.43E-06	0.0101	GCGCGCGCGCG
1	GCGCVTGCGCV	DHX15	-	76	86	5.43E-06	0.0101	GCGCGCGCGCG
1	GCGCVTGCGCV	PPP5D1	-	83	93	5.43E-06	0.0101	GCGCGCGCGCG
1	GCGCVTGCGCV	KDELR2	-	134	144	5.43E-06	0.0101	GCGCGCGCGCG

1	GCGCVTGCGCV	KDELR2	-	136	146	5.43E-06	0.0101	GCGCGCGCGCG
1	GCGCVTGCGCV	KDELR2	-	138	148	5.43E-06	0.0101	GCGCGCGCGCG
1	GCGCVTGCGCV	ZDHHC14	-	141	151	5.43E-06	0.0101	GCGCGCGCGCG
1	GCGCVTGCGCV	MIF4GD	-	160	170	5.43E-06	0.0101	GCGCGCGCGCG
1	GCGCVTGCGCV	PANK1	+	22	32	5.43E-06	0.0101	GCGCGCGCGCG
1	GCGCVTGCGCV	PRR12	+	47	57	5.43E-06	0.0101	GCGCGCGCGCG
1	GCGCVTGCGCV	C6orf48	+	58	68	5.43E-06	0.0101	GCGCGCGCGCG
1	GCGCVTGCGCV	DHX15	+	73	83	5.43E-06	0.0101	GCGCGCGCGCG
1	GCGCVTGCGCV	DHX15	+	75	85	5.43E-06	0.0101	GCGCGCGCGCG
1	GCGCVTGCGCV	PPP5D1	+	82	92	5.43E-06	0.0101	GCGCGCGCGCG
1	GCGCVTGCGCV	KDELR2	+	135	145	5.43E-06	0.0101	GCGCGCGCGCG
1	GCGCVTGCGCV	NSF	+	137	147	5.43E-06	0.0101	GCGCGCGCGCG
1	GCGCVTGCGCV	KDELR2	+	137	147	5.43E-06	0.0101	GCGCGCGCGCG
1	GCGCVTGCGCV	MBD6	+	148	158	5.43E-06	0.0101	GCGCGCGCGCG
1	GCGCVTGCGCV	AGO2	+	165	175	5.43E-06	0.0101	GCGCGCGCGCG
1	GCGCVTGCGCV	MRPL34	-	87	97	5.54E-06	0.0103	GAGCCTGCGCC
1	GCGCVTGCGCV	AHNAK	+	18	28	5.54E-06	0.0103	GAGCCTGCGCC
1	GCGCVTGCGCV	MIR1199	+	100	110	5.54E-06	0.0103	GAGCCTGCGCC
1	GCGCVTGCGCV	PIK3C2B	-	190	200	5.66E-06	0.0104	GAGCGTGCGCG
1	GCGCVTGCGCV	RP11-68I18.10	+	138	148	5.66E-06	0.0104	GAGCGTGCGCG
1	GCGCVTGCGCV	FAM195B	+	86	96	5.93E-06	0.0108	GCGCCTGTGCA
1	GCGCVTGCGCV	EIF3K	-	3	13	6.23E-06	0.0108	GCGCAGGCGCC
1	GCGCVTGCGCV	CANX	-	4	14	6.23E-06	0.0108	GCGCAGGCGCC
1	GCGCVTGCGCV	TCTE3	-	4	14	6.23E-06	0.0108	GCGCAGGCGCC
1	GCGCVTGCGCV	DLGAP4	-	6	16	6.23E-06	0.0108	GCGGCTGCGCC
1	GCGCVTGCGCV	RP1-122P22.4	-	9	19	6.23E-06	0.0108	GCGCAGGCGCC
1	GCGCVTGCGCV	RP1-101K10.6	-	27	37	6.23E-06	0.0108	GCGCAGGCGCC

1	GCGCVTGCGCV	SKP1	-	41	51	6.23E-06	0.0108	GCGCAGGCGCC
1	GCGCVTGCGCV	KDM2A	-	59	69	6.23E-06	0.0108	GCGCAGGCGCC
1	GCGCVTGCGCV	EED	-	69	79	6.23E-06	0.0108	GCGCAGGCGCC
1	GCGCVTGCGCV	ACTL6A	-	77	87	6.23E-06	0.0108	GCGGCTGCGCC
1	GCGCVTGCGCV	TOMM7	-	89	99	6.23E-06	0.0108	GCGCAGGCGCC
1	GCGCVTGCGCV	JAGN1	-	94	104	6.23E-06	0.0108	GCGCAGGCGCC
1	GCGCVTGCGCV	TMEM106C	-	118	128	6.23E-06	0.0108	GCGCAGGCGCC
1	GCGCVTGCGCV	BRK1	-	175	185	6.23E-06	0.0108	GCGCAGGCGCC
1	GCGCVTGCGCV	CISD1	-	191	201	6.23E-06	0.0108	GCGCAGGCGCC
1	GCGCVTGCGCV	GS1-124K5.13	+	10	20	6.23E-06	0.0108	GCGGCTGCGCC
1	GCGCVTGCGCV	RABGGTB	+	11	21	6.23E-06	0.0108	GCGCAGGCGCC
1	GCGCVTGCGCV	TCEB2	+	39	49	6.23E-06	0.0108	GCGGCTGCGCC
1	GCGCVTGCGCV	PSAP	+	87	97	6.23E-06	0.0108	GCGCAGGCGCC
1	GCGCVTGCGCV	UGP2	+	91	101	6.23E-06	0.0108	GCGGCTGCGCC
1	GCGCVTGCGCV	LRRC75A-AS1	+	97	107	6.23E-06	0.0108	GCGCAGGCGCC
1	GCGCVTGCGCV	MACF1	+	126	136	6.23E-06	0.0108	GCGGCTGCGCC
1	GCGCVTGCGCV	STIM2	+	126	136	6.23E-06	0.0108	GCGCAGGCGCC
1	GCGCVTGCGCV	NDUFA8	+	131	141	6.23E-06	0.0108	GCGCAGGCGCC
1	GCGCVTGCGCV	SFXN4	+	32	42	6.32E-06	0.0109	GCGGGTGCGCG
1	GCGCVTGCGCV	OMA1	+	141	151	6.32E-06	0.0109	GCGGGTGCGCG
1	GCGCVTGCGCV	PSMA7	+	165	175	6.32E-06	0.0109	GCGGGTGCGCG
1	GCGCVTGCGCV	SMG7	-	6	16	6.55E-06	0.0112	GCGCCTGTGCG
1	GCGCVTGCGCV	YARS2	-	23	33	6.55E-06	0.0112	GCGCATGCCCG
1	GCGCVTGCGCV	STIM2	-	103	113	6.55E-06	0.0112	GCGCATGCCCG
1	GCGCVTGCGCV	UBR7	-	52	62	6.76E-06	0.0113	CCGCCTGCGCC
1	GCGCVTGCGCV	FOXP4	-	76	86	6.76E-06	0.0113	CCGCCTGCGCC
1	GCGCVTGCGCV	DAZAP1	-	85	95	6.76E-06	0.0113	GCCCCTGCGCA

1	GCGCVTGCGCV	CDKN1C	-	186	196	6.76E-06	0.0113	CCGCCTGCGCC
1	GCGCVTGCGCV	AFF1	+	39	49	6.76E-06	0.0113	GCCCCTGCGCA
1	GCGCVTGCGCV	PLEC	+	53	63	6.76E-06	0.0113	CCGCCTGCGCC
1	GCGCVTGCGCV	TMEM141	+	74	84	6.76E-06	0.0113	CCGCCTGCGCC
1	GCGCVTGCGCV	BNIP3	+	78	88	6.76E-06	0.0113	GCCCCTGCGCA
1	GCGCVTGCGCV	NATD1	+	154	164	6.76E-06	0.0113	CCGCCTGCGCC
1	GCGCVTGCGCV	ASCC3	-	44	54	6.85E-06	0.0114	CCGCGTGCGCG
1	GCGCVTGCGCV	LRP4-AS1	-	50	60	6.96E-06	0.0116	GCGCGTGCCCA
1	GCGCVTGCGCV	UCHL3	-	41	51	7.10E-06	0.0118	ACGCATGCGCC
1	GCGCVTGCGCV	MRPL17	-	118	128	7.10E-06	0.0118	ACGCATGCGCC
1	GCGCVTGCGCV	EZH1	+	40	50	7.29E-06	0.0118	GCGCACGCGCC
1	GCGCVTGCGCV	CTSC	+	44	54	7.29E-06	0.0118	GCGCACGCGCC
1	GCGCVTGCGCV	MAST3	+	45	55	7.29E-06	0.0118	GCCCCTGCGCG
1	GCGCVTGCGCV	BNIP3	+	45	55	7.29E-06	0.0118	GCGCACGCGCC
1	GCGCVTGCGCV	TMEM135	+	65	75	7.29E-06	0.0118	GCGCACGCGCC
1	GCGCVTGCGCV	EIF3K	+	70	80	7.29E-06	0.0118	GCGCACGCGCC
1	GCGCVTGCGCV	HMGN1P20	+	72	82	7.29E-06	0.0118	GCGCACGCGCC
1	GCGCVTGCGCV	TMEM14B	+	75	85	7.29E-06	0.0118	GCGCACGCGCC
1	GCGCVTGCGCV	SNRNP70	-	75	85	7.29E-06	0.0118	GCGCACGCGCC
1	GCGCVTGCGCV	KMT2C	+	88	98	7.29E-06	0.0118	GCCCCTGCGCG
1	GCGCVTGCGCV	SLC9B1	+	111	121	7.29E-06	0.0118	GCCCCTGCGCG
1	GCGCVTGCGCV	PDRG1	-	158	168	7.29E-06	0.0118	GCGCACGCGCC
1	GCGCVTGCGCV	TBC1D2B	+	124	134	7.50E-06	0.012	GCGCGGGCGCC
1	GCGCVTGCGCV	FOXK1	-	125	135	7.50E-06	0.012	GCGCGGGCGCC
1	GCGCVTGCGCV	RPS12	-	169	179	7.81E-06	0.0125	GCGCATGTGCA
1	GCGCVTGCGCV	PDZD2	-	4	14	7.90E-06	0.0126	GCGCCTGCCCC
1	GCGCVTGCGCV	RP11-1055B8.4	+	47	57	7.90E-06	0.0126	GCGCCTGCCCC

1	GCGCVTGCGCV	RP11-15I20.1	+	176	186	7.90E-06	0.0126	GCGCCTGCCCC
1	GCGCVTGCGCV	MIF4GD	-	154	164	8.00E-06	0.0127	GCGCGTGCCCG
1	GCGCVTGCGCV	BTBD3	-	172	182	8.00E-06	0.0127	GCGCGTGCCCG
1	GCGCVTGCGCV	POM121C	-	51	61	8.36E-06	0.0132	GCGCATGTGCG
1	GCGCVTGCGCV	POM121	-	71	81	8.36E-06	0.0132	GCGCATGTGCG
1	GCGCVTGCGCV	EID3	-	96	106	8.61E-06	0.0135	GCCCATGCGCA
1	GCGCVTGCGCV	TMEM251	-	18	28	8.80E-06	0.0135	GCGCGCGCGCC
1	GCGCVTGCGCV	DMXL2	-	27	37	8.80E-06	0.0135	GCGCGCGCGCC
1	GCGCVTGCGCV	NSF	+	40	50	8.80E-06	0.0135	GCGCGCGCGCC
1	GCGCVTGCGCV	PRR12	+	49	59	8.80E-06	0.0135	GCGCGCGCGCC
1	GCGCVTGCGCV	AGO4	-	66	76	8.80E-06	0.0135	GCGCGCGCGCC
1	GCGCVTGCGCV	PPP5D1	-	81	91	8.80E-06	0.0135	GCGCGCGCGCC
1	GCGCVTGCGCV	KDELR2	+	139	149	8.80E-06	0.0135	GCGCGCGCGCC
1	GCGCVTGCGCV	ZDHHC14	+	142	152	8.80E-06	0.0135	GCGCGCGCGCC
1	GCGCVTGCGCV	NSF	-	136	146	8.80E-06	0.0135	GCGCGCGCGCC
1	GCGCVTGCGCV	MIF4GD	+	161	171	8.80E-06	0.0135	GCGCGCGCGCC
1	GCGCVTGCGCV	AGO2	-	164	174	8.80E-06	0.0135	GCGCGCGCGCC
1	GCGCVTGCGCV	DAD1	+	38	48	8.94E-06	0.0137	GCGTCTGCGCA
1	GCGCVTGCGCV	MRPL18	-	99	109	8.94E-06	0.0137	GCGTCTGCGCA
1	GCGCVTGCGCV	POM121	+	26	36	9.19E-06	0.014	GCGCGTGTGCA
1	GCGCVTGCGCV	RP4-591C20.9	+	52	62	9.19E-06	0.014	GCGCGTGTGCA
1	GCGCVTGCGCV	RPS12	+	170	180	9.47E-06	0.0144	GCACATGCGCA
1	GCGCVTGCGCV	NDUFAF5	+	132	142	9.79E-06	0.0149	GCGCTGGCGCA
1	GCGCVTGCGCV	THNSL1	-	83	93	1.01E-05	0.0153	GCGCCTGTGCC
1	GCGCVTGCGCV	BTBD3	+	104	114	1.01E-05	0.0153	GCGCATGCCCC
1	GCGCVTGCGCV	ZMIZ1	+	173	183	1.01E-05	0.0153	ACGCCGGCGCA
1	GCGCVTGCGCV	XPOT	+	50	60	1.02E-05	0.0154	GCGCGTGTGCG

1	GCGCVTGCGCV	RPS15	+	69	79	1.02E-05	0.0154	GCGCGTGTGCG
1	GCGCVTGCGCV	RAN	-	37	47	1.05E-05	0.0156	CCGCGTGCGCC
1	GCGCVTGCGCV	KMT2B	-	48	58	1.05E-05	0.0156	GCCCGTGCGCA
1	GCGCVTGCGCV	AP000253.1	+	66	76	1.05E-05	0.0156	CCGCGTGCGCC
1	GCGCVTGCGCV	ITGB3BP	-	82	92	1.06E-05	0.0157	GAGCCGGCGCA
1	GCGCVTGCGCV	MACF1	-	131	141	1.06E-05	0.0157	GAGCCGGCGCA
1	GCGCVTGCGCV	POM121C	+	52	62	1.07E-05	0.0158	GCACATGCGCG
1	GCGCVTGCGCV	POM121	+	72	82	1.07E-05	0.0158	GCACATGCGCG
1	GCGCVTGCGCV	PDIA3P2	-	2	12	1.08E-05	0.0158	GCGCTGGCGCG
1	GCGCVTGCGCV	RSPH10B	+	22	32	1.08E-05	0.0158	GCGCTGGCGCG
1	GCGCVTGCGCV	ARHGAP32	+	84	94	1.08E-05	0.0158	GCGCTGGCGCG
1	GCGCVTGCGCV	FOXP2	+	116	126	1.09E-05	0.0158	ACGCCGGCGCG
1	GCGCVTGCGCV	ST13	+	32	42	1.10E-05	0.0158	GCGGCGGCGCA
1	GCGCVTGCGCV	SYNJ2	+	62	72	1.10E-05	0.0158	GCGGCGGCGCA
1	GCGCVTGCGCV	MPRIPP1	-	74	84	1.10E-05	0.0158	GCGGCGGCGCA
1	GCGCVTGCGCV	ATXN10	+	90	100	1.10E-05	0.0158	GCGGCGGCGCA
1	GCGCVTGCGCV	ADCY9	+	116	126	1.10E-05	0.0158	GCGGCGGCGCA
1	GCGCVTGCGCV	H1F0	+	131	141	1.10E-05	0.0158	GCGGCGGCGCA
1	GCGCVTGCGCV	NDUFAB1	+	133	143	1.10E-05	0.0158	GCGGCGGCGCA
1	GCGCVTGCGCV	ZNHIT3	+	136	146	1.10E-05	0.0158	GCGGCGGCGCA
1	GCGCVTGCGCV	SPTB	-	133	143	1.10E-05	0.0158	GCGGCGGCGCA
1	GCGCVTGCGCV	ABTB2	-	161	171	1.10E-05	0.0158	GCGGCGGCGCA
1	GCGCVTGCGCV	MAPK8IP3	+	16	26	1.11E-05	0.0159	GCCCCTGCGCC
1	GCGCVTGCGCV	LRCH1	-	50	60	1.11E-05	0.0159	GCCCCTGCGCC
1	GCGCVTGCGCV	RBP1	+	167	177	1.11E-05	0.0159	GCCCCTGCGCC
1	GCGCVTGCGCV	NRBP2	-	48	58	1.16E-05	0.0164	GAGCCGGCGCG
1	GCGCVTGCGCV	ZNF767P	-	71	81	1.16E-05	0.0164	GAGCCGGCGCG

1	GCGCVTGCGCV	TMEM38B	+	105	115	1.16E-05	0.0164	GAGCCGGCGCG
1	GCGCVTGCGCV	DDX18	-	137	147	1.16E-05	0.0164	GCACGTGCGCA
1	GCGCVTGCGCV	UROS	-	94	104	1.19E-05	0.0165	GCGCTCGCGCA
1	GCGCVTGCGCV	ULK1	-	80	90	1.20E-05	0.0165	ACGCCCGCGCA
1	GCGCVTGCGCV	NRBP2	-	191	201	1.21E-05	0.0165	CCGCCGGCGCA
1	GCGCVTGCGCV	KCTD16	+	1	11	1.23E-05	0.0165	GAGCTTGCGCA
1	GCGCVTGCGCV	AL353644.10	+	84	94	1.23E-05	0.0165	GCGCGTGCCCC
1	GCGCVTGCGCV	LDHAL6B	-	101	111	1.23E-05	0.0165	GCGCGTGCCCC
1	GCGCVTGCGCV	MLLT4	+	5	15	1.26E-05	0.0165	GCGGCGGCGCG
1	GCGCVTGCGCV	FAM193B	+	9	19	1.26E-05	0.0165	GCGGCGGCGCG
1	GCGCVTGCGCV	BCORL1	+	9	19	1.26E-05	0.0165	GCGGCGGCGCG
1	GCGCVTGCGCV	ZNF503	+	16	26	1.26E-05	0.0165	GCGGCGGCGCG
1	GCGCVTGCGCV	MLLT4	+	24	34	1.26E-05	0.0165	GCGGCGGCGCG
1	GCGCVTGCGCV	TMEM135	-	16	26	1.26E-05	0.0165	GCGGCGGCGCG
1	GCGCVTGCGCV	CTD-2154B17.4	+	33	43	1.26E-05	0.0165	GCGGCGGCGCG
1	GCGCVTGCGCV	MIR1281	+	41	51	1.26E-05	0.0165	GCGGCGGCGCG
1	GCGCVTGCGCV	MEX3D	+	53	63	1.26E-05	0.0165	GCGGCGGCGCG
1	GCGCVTGCGCV	USP42	+	53	63	1.26E-05	0.0165	GCGGCGGCGCG
1	GCGCVTGCGCV	FOXK1	-	49	59	1.26E-05	0.0165	GCGGCGGCGCG
1	GCGCVTGCGCV	AC073046.25	+	65	75	1.26E-05	0.0165	GCGGCGGCGCG
1	GCGCVTGCGCV	BRD4	+	65	75	1.26E-05	0.0165	GCGGCGGCGCG
1	GCGCVTGCGCV	MPRIP	+	71	81	1.26E-05	0.0165	GCGGCGGCGCG
1	GCGCVTGCGCV	RP11-700A24.1	-	61	71	1.26E-05	0.0165	GCGGCGGCGCG
1	GCGCVTGCGCV	IRS2	+	72	82	1.26E-05	0.0165	GCGGCGGCGCG
1	GCGCVTGCGCV	MCM6	+	72	82	1.26E-05	0.0165	GCGGCGGCGCG
1	GCGCVTGCGCV	COBL	-	63	73	1.26E-05	0.0165	GCGGCGGCGCG
1	GCGCVTGCGCV	MAGOH	-	75	85	1.26E-05	0.0165	GCGGCGGCGCG

1	GCGCVTGCG CV	FOXP4	+	86	96	1.26E-05	0.0165	GCGGCGGCG CG
1	GCGCVTGCG CV	RP11-214D15.2	+	91	101	1.26E-05	0.0165	GCGGCGGCG CG
1	GCGCVTGCG CV	DAZAP1	+	94	104	1.26E-05	0.0165	GCGGCGGCG CG
1	GCGCVTGCG CV	USP46	-	91	101	1.26E-05	0.0165	GCGGCGGCG CG
1	GCGCVTGCG CV	DAZAP1	+	102	112	1.26E-05	0.0165	GCGGCGGCG CG
1	GCGCVTGCG CV	SMG1	+	109	119	1.26E-05	0.0165	GCGGCGGCG CG
1	GCGCVTGCG CV	CCT6A	+	111	121	1.26E-05	0.0165	GCGGCGGCG CG
1	GCGCVTGCG CV	CHRDL2	-	106	116	1.26E-05	0.0165	GCGGCGGCG CG
1	GCGCVTGCG CV	EP400	-	109	119	1.26E-05	0.0165	GCGGCGGCG CG
1	GCGCVTGCG CV	PXN	+	127	137	1.26E-05	0.0165	GCGGCGGCG CG
1	GCGCVTGCG CV	C1orf233	+	129	139	1.26E-05	0.0165	GCGGCGGCG CG
1	GCGCVTGCG CV	DIP2A	+	131	141	1.26E-05	0.0165	GCGGCGGCG CG
1	GCGCVTGCG CV	CEP350	-	124	134	1.26E-05	0.0165	GCGGCGGCG CG
1	GCGCVTGCG CV	POLR2L	-	126	136	1.26E-05	0.0165	GCGGCGGCG CG
1	GCGCVTGCG CV	ORAOV1	-	149	159	1.26E-05	0.0165	GCGGCGGCG CG
1	GCGCVTGCG CV	C17orf58	-	158	168	1.26E-05	0.0165	GCGGCGGCG CG
1	GCGCVTGCG CV	PRRC2B	-	170	180	1.26E-05	0.0165	GCGGCGGCG CG
1	GCGCVTGCG CV	C17orf58	-	190	200	1.26E-05	0.0165	GCGGCGGCG CG
1	GCGCVTGCG CV	PSMB3	+	116	126	1.33E-05	0.0174	GCGGTTGCGC A
1	GCGCVTGCG CV	DIRAS3	+	43	53	1.38E-05	0.0179	ACGCAGGCG CA
1	GCGCVTGCG CV	MICAL3	+	68	78	1.38E-05	0.0179	GCGCTCGCGC G
1	GCGCVTGCG CV	TMEM106C	-	75	85	1.38E-05	0.0179	GCGCTCGCGC G
1	GCGCVTGCG CV	ERGIC3	-	80	90	1.38E-05	0.0179	ACGCAGGCG CA
1	GCGCVTGCG CV	CLASRP	-	134	144	1.38E-05	0.0179	GCGCTCGCGC G
1	GCGCVTGCG CV	TMEM38B	-	158	168	1.38E-05	0.0179	GCGCTCGCGC G
1	GCGCVTGCG CV	TRAK1	+	70	80	1.39E-05	0.018	ACGCCCGCGC G
1	GCGCVTGCG CV	HIST2H4A	+	25	35	1.41E-05	0.0181	CCGCCGGCGC G

1	GCGCVTGCGCV	PRKACA	-	49	59	1.41E-05	0.0181	GCGGCCGCGCA
1	GCGCVTGCGCV	TJP2	-	24	34	1.47E-05	0.0188	GAGCCCGCGCG
1	GCGCVTGCGCV	RPS5	-	115	125	1.47E-05	0.0188	GAGCCCGCGCG
1	GCGCVTGCGCV	NSF	+	34	44	1.47E-05	0.0188	GAGCCCGCGCG
1	GCGCVTGCGCV	AKAP17A	+	85	95	1.47E-05	0.0188	GAGCCCGCGCG
1	GCGCVTGCGCV	COA7	-	125	135	1.51E-05	0.0192	GCGCCGGCCCA
1	GCGCVTGCGCV	ARRDC2	+	141	151	1.51E-05	0.0192	GCGCCGGCCCA
1	GCGCVTGCGCV	SLC39A9	-	8	18	1.53E-05	0.0193	GCGGTTGCGCG
1	GCGCVTGCGCV	POFUT1	-	48	58	1.53E-05	0.0193	CCGCCCGCGCA
1	GCGCVTGCGCV	MAST3	-	50	60	1.53E-05	0.0193	CCGCCCGCGCA
1	GCGCVTGCGCV	C1GALT1C1	-	99	109	1.53E-05	0.0193	CCGCCCGCGCA
1	GCGCVTGCGCV	C6orf132	-	142	152	1.53E-05	0.0193	CCGCCCGCGCA
1	GCGCVTGCGCV	ING5	+	95	105	1.53E-05	0.0193	CCGCCCGCGCA
1	GCGCVTGCGCV	TM2D3	-	53	63	1.55E-05	0.0194	ACGCAGGCGCG
1	GCGCVTGCGCV	LRP4-AS1	-	84	94	1.55E-05	0.0194	ACGGCTGCGCG
1	GCGCVTGCGCV	CCNT1	-	165	175	1.55E-05	0.0194	ACGCAGGCGCG
1	GCGCVTGCGCV	DRG1	-	166	176	1.55E-05	0.0194	ACGCAGGCGCG
1	GCGCVTGCGCV	TEX22	+	12	22	1.55E-05	0.0194	ACGCAGGCGCG
1	GCGCVTGCGCV	SAP18	+	19	29	1.55E-05	0.0194	ACGCAGGCGCG
1	GCGCVTGCGCV	RPL18A	+	122	132	1.55E-05	0.0194	ACGGCTGCGCG
1	GCGCVTGCGCV	PDIA3P2	-	102	112	1.57E-05	0.0194	GCGGAGGCGCA
1	GCGCVTGCGCV	TBC1D2B	+	111	121	1.57E-05	0.0194	GCGGAGGCGCA
1	GCGCVTGCGCV	IQSEC2	-	10	20	1.60E-05	0.0195	GCGGCCGCGCG
1	GCGCVTGCGCV	WRB	-	117	127	1.60E-05	0.0195	GCGCCTGCGAA
1	GCGCVTGCGCV	RP11-52A20.2	-	130	140	1.60E-05	0.0195	CAGCCTGCGCA
1	GCGCVTGCGCV	AGO2	-	170	180	1.60E-05	0.0195	GCGGCCGCGCG
1	GCGCVTGCGCV	RPL41P2	-	171	181	1.60E-05	0.0195	CAGCCTGCGCA

1	GCGCVTGCGCV	STX1A	-	188	198	1.60E-05	0.0195	GCGGCCGCGCG
1	GCGCVTGCGCV	DOT1L	+	19	29	1.60E-05	0.0195	GCGGCCGCGCG
1	GCGCVTGCGCV	PRR5	+	52	62	1.60E-05	0.0195	CAGCCTGCGCA
1	GCGCVTGCGCV	FBXO6	+	95	105	1.60E-05	0.0195	GCGGCCGCGCG
1	GCGCVTGCGCV	ACTR10	+	114	124	1.60E-05	0.0195	GCGGCCGCGCG
1	GCGCVTGCGCV	HSDL1	+	156	166	1.60E-05	0.0195	GCGGCCGCGCG
1	GCGCVTGCGCV	ENO1	-	30	40	1.63E-05	0.0198	GAGGCTGCGCG
1	GCGCVTGCGCV	VWCE	-	32	42	1.63E-05	0.0198	GAGGCTGCGCG
1	GCGCVTGCGCV	PPIH	-	35	45	1.63E-05	0.0198	GAGCAGGCGCG
1	GCGCVTGCGCV	CRYBA2	-	95	105	1.63E-05	0.0198	GAGGCTGCGCG
1	GCGCVTGCGCV	MINOS1	+	58	68	1.65E-05	0.0199	GCGTCTGCGCC
1	GCGCVTGCGCV	YAE1D1	-	33	43	1.67E-05	0.0201	GCGTGTGCGCG
1	GCGCVTGCGCV	RPS15	+	71	81	1.67E-05	0.0201	GCGTGTGCGCG
1	GCGCVTGCGCV	CCT8	-	42	52	1.69E-05	0.0202	ACGCACGCGCA
1	GCGCVTGCGCV	LRP4	-	84	94	1.69E-05	0.0202	GCGCCGGCCCG
1	GCGCVTGCGCV	MRPL20	-	152	162	1.69E-05	0.0202	ACGCACGCGCA
1	GCGCVTGCGCV	APPL1	-	184	194	1.69E-05	0.0202	GCGCCGGCCCG
1	GCGCVTGCGCV	PIP5K1C	+	83	93	1.69E-05	0.0202	GCGCCGGCCCG
1	GCGCVTGCGCV	ATP5O	-	90	100	1.71E-05	0.0204	CCGCAGGCGCA
1	GCGCVTGCGCV	MARS2	+	68	78	1.71E-05	0.0204	CCGGCTGCGCA
1	GCGCVTGCGCV	PSMB1	+	181	191	1.71E-05	0.0204	CCGCAGGCGCA
1	GCGCVTGCGCV	PKD1P6	-	51	61	1.74E-05	0.0206	CCGCCCGCGCG
1	GCGCVTGCGCV	MAPRE1	-	142	152	1.74E-05	0.0206	CCGCCCGCGCG
1	GCGCVTGCGCV	ACAA2	+	39	49	1.74E-05	0.0206	CCGCCCGCGCG
1	GCGCVTGCGCV	FGF18	+	52	62	1.74E-05	0.0206	CCGCCCGCGCG
1	GCGCVTGCGCV	XPOT	+	172	182	1.74E-05	0.0206	CCGCCCGCGCG
1	GCGCVTGCGCV	SNHG3	-	75	85	1.76E-05	0.0207	AAGCATGCGCA

1	GCGCVTGCG CV	IMPDH1P10	-	41	51	1.77E-05	0.0207	GCGGAGGCG CG
1	GCGCVTGCG CV	SDHC	-	187	197	1.77E-05	0.0207	GCGGAGGCG CG
1	GCGCVTGCG CV	SLC6A20	+	98	108	1.77E-05	0.0207	GCGGAGGCG CG
1	GCGCVTGCG CV	TSN	+	140	150	1.77E-05	0.0207	GCGGAGGCG CG
1	GCGCVTGCG CV	RP4-758J18.2	+	144	154	1.77E-05	0.0207	GCGGAGGCG CG
1	GCGCVTGCG CV	RPL10	-	2	12	1.81E-05	0.0209	GCGCCTGCGA G
1	GCGCVTGCG CV	RP11-454L9.2	-	63	73	1.81E-05	0.0209	GCGCCTGCGA G
1	GCGCVTGCG CV	MKKS	-	89	99	1.81E-05	0.0209	GAGCACGCG CA
1	GCGCVTGCG CV	TACC2	+	22	32	1.81E-05	0.0209	GAGCACGCG CA
1	GCGCVTGCG CV	EIF3K	+	85	95	1.81E-05	0.0209	GCGCCTGCGA G
1	GCGCVTGCG CV	QPCT	+	104	114	1.81E-05	0.0209	CAGCCTGCGC G
1	GCGCVTGCG CV	MNAT1	+	189	199	1.81E-05	0.0209	GCGCCTGCGA G
1	GCGCVTGCG CV	SPINT2	-	141	151	1.82E-05	0.021	GCGCTTGCCC A
1	GCGCVTGCG CV	CKS2	-	146	156	1.86E-05	0.0212	GAGCGGGCG CA
1	GCGCVTGCG CV	KMT2E	+	48	58	1.86E-05	0.0212	GAGCGGGCG CA
1	GCGCVTGCG CV	ITGB4	-	51	61	1.87E-05	0.0212	GCGCCCGCCC A
1	GCGCVTGCG CV	ARL3	-	52	62	1.87E-05	0.0212	GCGCCCGCCC A
1	GCGCVTGCG CV	NGRN	-	80	90	1.87E-05	0.0212	GCGCCCGCCC A
1	GCGCVTGCG CV	SH3BP2	-	96	106	1.87E-05	0.0212	GCGCCCGCCC A
1	GCGCVTGCG CV	ZNF767P	-	183	193	1.87E-05	0.0212	GCGCCCGCCC A
1	GCGCVTGCG CV	RAN	-	190	200	1.87E-05	0.0212	GCGCCCGCCC A
1	GCGCVTGCG CV	FOXK1	+	16	26	1.87E-05	0.0212	GCGCCCGCCC A
1	GCGCVTGCG CV	TMEM126A	+	91	101	1.87E-05	0.0212	GCGCCCGCCC A
1	GCGCVTGCG CV	FOXP1	-	9	19	1.92E-05	0.0216	GCGCTGGCGC C
1	GCGCVTGCG CV	POM121	-	57	67	1.92E-05	0.0216	ACGCACGCGC G
1	GCGCVTGCG CV	XPNPEP1	-	67	77	1.92E-05	0.0216	GCGCTGGCGC C
1	GCGCVTGCG CV	PACS1	-	80	90	1.92E-05	0.0216	ACGCACGCGC G

1	GCGCVTGCG CV	EIF4EBP1	-	88	98	1.92E-05	0.0216	GCGCTGGCGC C
1	GCGCVTGCG CV	TEX30	-	108	118	1.92E-05	0.0216	ACGCACGCGC G
1	GCGCVTGCG CV	SDHAF2	-	84	94	1.96E-05	0.0218	CCGGCTGCGC G
1	GCGCVTGCG CV	PSMC4	-	95	105	1.96E-05	0.0218	CCGCAGGCGC G
1	GCGCVTGCG CV	RP11-307E17.8	-	103	113	1.96E-05	0.0218	CCGGCTGCGC G
1	GCGCVTGCG CV	TMEM187	-	128	138	1.96E-05	0.0218	CCGCAGGCGC G
1	GCGCVTGCG CV	NR4A3	-	130	140	1.96E-05	0.0218	CCGGCTGCGC G
1	GCGCVTGCG CV	MSL3P1	-	147	157	1.96E-05	0.0218	CCGGCTGCGC G
1	GCGCVTGCG CV	PEBP4	+	7	17	1.96E-05	0.0218	ACGCCGGCGC C
1	GCGCVTGCG CV	SNHG9	+	175	185	1.96E-05	0.0218	ACGCCGGCGC C
1	GCGCVTGCG CV	TMEM106C	-	85	95	1.97E-05	0.0218	ACGCGGGCG CG
1	GCGCVTGCG CV	ZNHIT3	+	109	119	1.97E-05	0.0218	ACGCGGGCG CG
1	GCGCVTGCG CV	LRR1	-	40	50	2.00E-05	0.022	GCGGGGGCG CA
1	GCGCVTGCG CV	APTX	+	49	59	2.00E-05	0.022	GCGGGGGCG CA
1	GCGCVTGCG CV	CHAD	+	61	71	2.00E-05	0.022	GCGGGGGCG CA
1	GCGCVTGCG CV	JAGN1	+	179	189	2.00E-05	0.022	GCGGGGGCG CA
1	GCGCVTGCG CV	GDAP1	+	83	93	2.03E-05	0.0224	GCCCGTGCGC C
1	GCGCVTGCG CV	SLX4	-	34	44	2.08E-05	0.0227	GAGCGGGCG CG
1	GCGCVTGCG CV	KIAA1522	-	49	59	2.08E-05	0.0227	GAGCGGGCG CG
1	GCGCVTGCG CV	SEMA5A	-	75	85	2.08E-05	0.0227	GAGCGGGCG CG
1	GCGCVTGCG CV	EPPK1	+	83	93	2.08E-05	0.0227	GAGCGGGCG CG
1	GCGCVTGCG CV	HIVEP2	-	140	150	2.11E-05	0.0227	GCGCCGGTGC A
1	GCGCVTGCG CV	LINC00674	-	178	188	2.11E-05	0.0227	GCGGCTGCCC A
1	GCGCVTGCG CV	VMAC	-	189	199	2.11E-05	0.0227	GCGCCGGTGC A
1	GCGCVTGCG CV	ARPC5	+	45	55	2.11E-05	0.0227	GCGCAGGCCC A
1	GCGCVTGCG CV	DHX15	+	83	93	2.11E-05	0.0227	GCGCAGGCCC A
1	GCGCVTGCG CV	DMXL2	+	129	139	2.11E-05	0.0227	GCGCCGGTGC A

1	GCGCVTGCGCV	PSMA1	-	3	13	2.12E-05	0.0227	GCGCCCGCCCG
1	GCGCVTGCGCV	ERI3	+	57	67	2.12E-05	0.0227	GCGCCCGCCCG
1	GCGCVTGCGCV	BRD1	+	72	82	2.12E-05	0.0227	GCGCCCGCCCG
1	GCGCVTGCGCV	DRD4	+	94	104	2.12E-05	0.0227	GCGCCCGCCCG
1	GCGCVTGCGCV	KAZN	+	101	111	2.12E-05	0.0227	GCGCCCGCCCG
1	GCGCVTGCGCV	PKD1P6	+	113	123	2.12E-05	0.0227	GCGCCCGCCCG
1	GCGCVTGCGCV	XPOT	+	168	178	2.12E-05	0.0227	GCGCCCGCCCG
1	GCGCVTGCGCV	MIR1199	-	159	169	2.13E-05	0.0228	CCGCACGCGCA
1	GCGCVTGCGCV	MAGI1	-	18	28	2.15E-05	0.023	ACGCGCGCGCA
1	GCGCVTGCGCV	TEX30	-	104	114	2.15E-05	0.023	ACGCGCGCGCA
1	GCGCVTGCGCV	EP400	+	74	84	2.22E-05	0.023	GCGGACGCGCG
1	GCGCVTGCGCV	PEBP4	-	12	22	2.24E-05	0.023	GCGGCGGCGCC
1	GCGCVTGCGCV	RP11-521B24.3	-	28	38	2.24E-05	0.023	GCGGCGGCGCC
1	GCGCVTGCGCV	TRIO	-	110	120	2.24E-05	0.023	GCGGCGGCGCC
1	GCGCVTGCGCV	ANKRD11	-	151	161	2.24E-05	0.023	GCGGCGGCGCC
1	GCGCVTGCGCV	POFUT1	-	154	164	2.24E-05	0.023	GCGGCGGCGCC
1	GCGCVTGCGCV	AGO4	-	187	197	2.24E-05	0.023	GCGGCGGCGCC
1	GCGCVTGCGCV	PLEKHG2	-	191	201	2.24E-05	0.023	GCGGCGGCGCC
1	GCGCVTGCGCV	UBE2H	+	15	25	2.24E-05	0.023	GCGGCGGCGCC
1	GCGCVTGCGCV	SETD1B	+	44	54	2.24E-05	0.023	GCGGCGGCGCC
1	GCGCVTGCGCV	IRF2BP2	+	73	83	2.24E-05	0.023	GCGGCGGCGCC
1	GCGCVTGCGCV	FZD7	+	75	85	2.24E-05	0.023	GCGGCGGCGCC
1	GCGCVTGCGCV	FAM160B1	+	86	96	2.24E-05	0.023	GCGGCGGCGCC
1	GCGCVTGCGCV	RP4-734G22.3	+	88	98	2.24E-05	0.023	GCGGCGGCGCC
1	GCGCVTGCGCV	C6orf132	+	112	122	2.24E-05	0.023	GCGGCGGCGCC
1	GCGCVTGCGCV	SLX4	+	125	135	2.24E-05	0.023	GCGGCGGCGCC
1	GCGCVTGCGCV	CDC42BPB	+	146	156	2.24E-05	0.023	GCGGCGGCGCC

1	GCGCVTGCG CV	LEPROTL1	-	65	75	2.25E-05	0.023	GCGGGGGCG CG
1	GCGCVTGCG CV	KIF1B	-	111	121	2.25E-05	0.023	GCGGGGGCG CG
1	GCGCVTGCG CV	TCF20	-	135	145	2.25E-05	0.023	GCGGGGGCG CG
1	GCGCVTGCG CV	PACS1	-	139	149	2.25E-05	0.023	GCGGGGGCG CG
1	GCGCVTGCG CV	SCMH1	-	175	185	2.25E-05	0.023	GCGGGGGCG CG
1	GCGCVTGCG CV	COBL	+	24	34	2.25E-05	0.023	GCGGGGGCG CG
1	GCGCVTGCG CV	SLX4	+	70	80	2.25E-05	0.023	GCGGGGGCG CG
1	GCGCVTGCG CV	UXS1	+	79	89	2.25E-05	0.023	GCGGGGGCG CG
1	GCGCVTGCG CV	RP1-66C13.4	+	81	91	2.25E-05	0.023	GCGGGGGCG CG
1	GCGCVTGCG CV	RAB28	+	82	92	2.25E-05	0.023	GCGGGGGCG CG
1	GCGCVTGCG CV	CYTH1	+	108	118	2.25E-05	0.023	GCGGGGGCG CG
1	GCGCVTGCG CV	NSF	+	131	141	2.25E-05	0.023	GCGGGGGCG CG
1	GCGCVTGCG CV	TMEM98	+	166	176	2.25E-05	0.023	GCGGGGGCG CG
1	GCGCVTGCG CV	SLC6A7	+	173	183	2.25E-05	0.023	GCGGGGGCG CG
1	GCGCVTGCG CV	SNRPD3	-	41	51	2.35E-05	0.0235	GCGGCTGCCC G
1	GCGCVTGCG CV	RP4-591C20.9	-	45	55	2.35E-05	0.0235	GCGCAGGCCC G
1	GCGCVTGCG CV	MPRIPP1	-	68	78	2.35E-05	0.0235	GCGCAGGCCC G
1	GCGCVTGCG CV	GPR180	-	71	81	2.35E-05	0.0235	GCGGCTGCCC G
1	GCGCVTGCG CV	IGFBP4	-	85	95	2.35E-05	0.0235	GCGGCTGCCC G
1	GCGCVTGCG CV	TP53TG1	-	98	108	2.35E-05	0.0235	GCGCAGGCCC G
1	GCGCVTGCG CV	DNAJB3	-	125	135	2.35E-05	0.0235	GCGCAGGCCC G
1	GCGCVTGCG CV	EEF1G	+	17	27	2.35E-05	0.0235	GCGCAGGCCC G
1	GCGCVTGCG CV	IRS1	+	41	51	2.35E-05	0.0235	GCGGCTGCCC G
1	GCGCVTGCG CV	CHMP3	+	62	72	2.35E-05	0.0235	GCGGCTGCCC G
1	GCGCVTGCG CV	TMEM141	+	86	96	2.35E-05	0.0235	GCGCAGGCCC G
1	GCGCVTGCG CV	MIR1281	+	88	98	2.35E-05	0.0235	GCGCAGGCCC G
1	GCGCVTGCG CV	ZBTB4	+	89	99	2.35E-05	0.0235	GCGCAGGCCC G

1	GCGCVTGCGCV	USP2	+	100	110	2.35E-05	0.0235	GCGGCTGCCCG
1	GCGCVTGCGCV	CDC42BPG	+	150	160	2.35E-05	0.0235	GCGGCTGCCCG
1	GCGCVTGCGCV	CAMSAP3	-	130	140	2.38E-05	0.0237	GCGCTCGCGCC
1	GCGCVTGCGCV	NDUFA8	+	119	129	2.38E-05	0.0237	CCGGATGCGCA
1	GCGCVTGCGCV	METTL5	-	110	120	2.41E-05	0.0239	CCGCACGCGCG
1	GCGCVTGCGCV	PSMA7	-	138	148	2.41E-05	0.0239	ACGCCCGCGCC
1	GCGCVTGCGCV	ATXN10	+	12	22	2.44E-05	0.0239	CCGCCGGCGCC
1	GCGCVTGCGCV	PANK1	+	20	30	2.44E-05	0.0239	ACGCGCGCGCG
1	GCGCVTGCGCV	PANK1	-	23	33	2.44E-05	0.0239	ACGCGCGCGCG
1	GCGCVTGCGCV	LRP4	-	27	37	2.44E-05	0.0239	CCGCCGGCGCC
1	GCGCVTGCGCV	GLG1	-	40	50	2.44E-05	0.0239	CCGCCGGCGCC
1	GCGCVTGCGCV	IQSEC3	-	84	94	2.44E-05	0.0239	CCGCCGGCGCC
1	GCGCVTGCGCV	NCOR2	+	147	157	2.44E-05	0.0239	CCGCCGGCGCC
1	GCGCVTGCGCV	MIF4GD	+	159	169	2.44E-05	0.0239	ACGCGCGCGCG
1	GCGCVTGCGCV	STRN	-	158	168	2.44E-05	0.0239	CCGCCGGCGCC
1	GCGCVTGCGCV	KMT2B	-	176	186	2.44E-05	0.0239	CCGCCGGCGCC
1	GCGCVTGCGCV	SLC39A9	+	3	13	2.47E-05	0.0239	GCGGGCGCGCA
1	GCGCVTGCGCV	TBC1D2B	-	43	53	2.47E-05	0.0239	GCGGGCGCGCA
1	GCGCVTGCGCV	TBC1D2B	-	45	55	2.47E-05	0.0239	CCGCGGGCGCG
1	GCGCVTGCGCV	RRM2	-	78	88	2.47E-05	0.0239	CCGCGGGCGCG
1	GCGCVTGCGCV	CASZ1	-	104	114	2.47E-05	0.0239	GCGGGCGCGCA
1	GCGCVTGCGCV	CASZ1	-	106	116	2.47E-05	0.0239	CCGCGGGCGCG
1	GCGCVTGCGCV	PDXDC2P	+	164	174	2.47E-05	0.0239	CCGCGGGCGCG
1	GCGCVTGCGCV	ITGB4	-	190	200	2.47E-05	0.0239	GCGGGCGCGCA
1	GCGCVTGCGCV	CYCS	+	31	41	2.51E-05	0.0243	CAGCATGCGCG
1	GCGCVTGCGCV	POM121C	+	20	30	2.54E-05	0.0246	GCGCTTGTGCA
1	GCGCVTGCGCV	HCRTR1	+	88	98	2.59E-05	0.025	GAGCCCGCGCC

1	GCGCVTGCGCV	DAD1	+	123	133	2.59E-05	0.025	GAGGGTGCGCA
1	GCGCVTGCGCV	FOXJ2	+	136	146	2.59E-05	0.025	GAGCCCGCGCC
1	GCGCVTGCGCV	PRR12	+	45	55	2.60E-05	0.025	GAGCGCGCGCG
1	GCGCVTGCGCV	C17orf58	-	65	75	2.60E-05	0.025	GAGCGCGCGCG
1	GCGCVTGCGCV	PPP5D1	-	85	95	2.60E-05	0.025	GAGCGCGCGCG
1	GCGCVTGCGCV	SSTR2	-	124	134	2.60E-05	0.025	GAGCGCGCGCG
1	GCGCVTGCGCV	PLEKHN1	+	61	71	2.64E-05	0.025	CCGCCTGCCCG
1	GCGCVTGCGCV	PSMC4	+	66	76	2.64E-05	0.025	GCGCACGCCCA
1	GCGCVTGCGCV	AGAP1	-	85	95	2.64E-05	0.025	CCGCCTGCCCG
1	GCGCVTGCGCV	PACS1	-	114	124	2.64E-05	0.025	CCGCCTGCCCG
1	GCGCVTGCGCV	RP9P	-	117	127	2.64E-05	0.025	CCGCCTGCCCG
1	GCGCVTGCGCV	SYNPO	-	122	132	2.64E-05	0.025	CCGCCTGCCCG
1	GCGCVTGCGCV	MAOB	-	152	162	2.64E-05	0.025	CCGCCTGCCCG
1	GCGCVTGCGCV	SYPL1	+	53	63	2.65E-05	0.0251	GCGCGGGCCCA
1	GCGCVTGCGCV	SH3D21	+	72	82	2.65E-05	0.0251	GCGCGGGCCCA
1	GCGCVTGCGCV	AC096772.6	+	160	170	2.69E-05	0.0252	GAGCCTGTGCA
1	GCGCVTGCGCV	TRIM8	-	32	42	2.73E-05	0.0252	CCGCGCGCGCA
1	GCGCVTGCGCV	MRPL15	-	60	70	2.73E-05	0.0252	CCGCGCGCGCA
1	GCGCVTGCGCV	BRD4	+	79	89	2.73E-05	0.0252	CCGCGCGCGCA
1	GCGCVTGCGCV	HNRNPA1	+	85	95	2.73E-05	0.0252	ACGCAGGCGCC
1	GCGCVTGCGCV	CETN2	-	87	97	2.73E-05	0.0252	CCGCGCGCGCA
1	GCGCVTGCGCV	CREG1	+	149	159	2.73E-05	0.0252	CCGCGCGCGCA
1	GCGCVTGCGCV	PSMB1	-	186	196	2.73E-05	0.0252	ACGGCTGCGCC
1	GCGCVTGCGCV	MINK1	+	12	22	2.76E-05	0.0252	GCCCCGGCGCG
1	GCGCVTGCGCV	RAP1GDS1	+	14	24	2.76E-05	0.0252	GCGGCCGCGCC
1	GCGCVTGCGCV	PSMA7	+	35	45	2.76E-05	0.0252	GCCCCGGCGCG
1	GCGCVTGCGCV	TMEM242	+	46	56	2.76E-05	0.0252	GCCCCGGCGCG

1	GCGCVTGCGCV	CSNK2A2	-	40	50	2.76E-05	0.0252	GCGGCCGCGCC
1	GCGCVTGCGCV	NRARP	+	59	69	2.76E-05	0.0252	GCCCCGGCGCG
1	GCGCVTGCGCV	LINC00998	-	84	94	2.76E-05	0.0252	GCCCCGGCGCG
1	GCGCVTGCGCV	AGO2	+	99	109	2.76E-05	0.0252	GCGGCCGCGCC
1	GCGCVTGCGCV	CDC42BPB	-	90	100	2.76E-05	0.0252	GCGGCCGCGCC
1	GCGCVTGCGCV	ATP11A	-	98	108	2.76E-05	0.0252	GCGGCCGCGCC
1	GCGCVTGCGCV	TRIO	-	98	108	2.76E-05	0.0252	GCGGCCGCGCC
1	GCGCVTGCGCV	UQCR11	+	113	123	2.76E-05	0.0252	GCGGCCGCGCC
1	GCGCVTGCGCV	AGO2	-	104	114	2.76E-05	0.0252	GCCCCGGCGCG
1	GCGCVTGCGCV	RP11-47A8.5	+	147	157	2.76E-05	0.0252	GCGGCCGCGCC
1	GCGCVTGCGCV	MLLT4	-	167	177	2.76E-05	0.0252	GCGGCCGCGCC
1	GCGCVTGCGCV	FZD7	-	174	184	2.76E-05	0.0252	GCGGCCGCGCC
1	GCGCVTGCGCV	AGAP1	+	187	197	2.76E-05	0.0252	GCCCCGGCGCG
1	GCGCVTGCGCV	SCAF1	-	184	194	2.76E-05	0.0252	GCCCCGGCGCG
1	GCGCVTGCGCV	PXN	+	10	20	2.81E-05	0.0252	CAGCGTGCGCA
1	GCGCVTGCGCV	ZDHHC14	-	25	35	2.81E-05	0.0252	GCACCGGCGCA
1	GCGCVTGCGCV	SLX4	-	32	42	2.81E-05	0.0252	GCGGGCGCGCG
1	GCGCVTGCGCV	C6orf48	-	49	59	2.81E-05	0.0252	GCGGGCGCGCG
1	GCGCVTGCGCV	AGO4	+	63	73	2.81E-05	0.0252	GCGGGCGCGCG
1	GCGCVTGCGCV	AC132812.1	-	55	65	2.81E-05	0.0252	GCGGGCGCGCG
1	GCGCVTGCGCV	EPPK1	+	85	95	2.81E-05	0.0252	GCGGGCGCGCG
1	GCGCVTGCGCV	NRBP2	-	83	93	2.81E-05	0.0252	GCGGGCGCGCG
1	GCGCVTGCGCV	TMEM106C	-	83	93	2.81E-05	0.0252	GCGGGCGCGCG
1	GCGCVTGCGCV	SYNJ2	+	93	103	2.81E-05	0.0252	GCGGGCGCGCG
1	GCGCVTGCGCV	PXN	+	114	124	2.81E-05	0.0252	GCGGGCGCGCG
1	GCGCVTGCGCV	WDR88	+	115	125	2.81E-05	0.0252	GCGGGCGCGCG
1	GCGCVTGCGCV	KMT2C	-	107	117	2.81E-05	0.0252	GCGGGCGCGCG

1	GCGCVTGCGCV	DMXL2	-	128	138	2.81E-05	0.0252	GCACCGGCGCA
1	GCGCVTGCGCV	PHLPP1	+	139	149	2.81E-05	0.0252	GCGGGCGCGCG
1	GCGCVTGCGCV	RAPGEF1	+	140	150	2.81E-05	0.0252	GCGGGCGCGCG
1	GCGCVTGCGCV	FAM63B	-	146	156	2.81E-05	0.0252	GCGGGCGCGCG
1	GCGCVTGCGCV	MIR611	-	14	24	2.84E-05	0.0253	GAGCAGGCGCC
1	GCGCVTGCGCV	CRIM1	+	49	59	2.84E-05	0.0253	GAGGCTGCGCC
1	GCGCVTGCGCV	MAPK8IP3	-	108	118	2.84E-05	0.0253	GAGGCTGCGCC
1	GCGCVTGCGCV	RNF139	-	108	118	2.84E-05	0.0253	GAGGCTGCGCC
1	GCGCVTGCGCV	SP5	+	154	164	2.84E-05	0.0253	GAGCAGGCGCC
1	GCGCVTGCGCV	FAM84A	+	163	173	2.84E-05	0.0253	GAGCAGGCGCC
1	GCGCVTGCGCV	NRBP2	+	186	196	2.84E-05	0.0253	GAGGCTGCGCC
1	GCGCVTGCGCV	N6AMT2	-	37	47	2.98E-05	0.0253	GCCCTTGCGCA
1	GCGCVTGCGCV	XPOT	+	52	62	2.98E-05	0.0253	GCGTGTGCGCC
1	GCGCVTGCGCV	CTD-2154B17.4	-	58	68	2.98E-05	0.0253	GCGCACGCCCG
1	GCGCVTGCGCV	DAZAP1	-	79	89	2.98E-05	0.0253	GCGCACGCCCG
1	GCGCVTGCGCV	EIF4EBP1	+	95	105	2.98E-05	0.0253	GCGCCCGTGCG
1	GCGCVTGCGCV	RPL5	-	99	109	2.98E-05	0.0253	GCCCTTGCGCA
1	GCGCVTGCGCV	MAP4	+	121	131	2.98E-05	0.0253	GCGTGTGCGCC
1	GCGCVTGCGCV	C6orf99	-	23	33	3.00E-05	0.0253	GCGCCGGCCCC
1	GCGCVTGCGCV	BRD1	+	60	70	3.00E-05	0.0253	GCGCCGGCCCC
1	GCGCVTGCGCV	PTGES3	-	71	81	3.00E-05	0.0253	GCGCCGGCCCC
1	GCGCVTGCGCV	PHLPP1	-	71	81	3.00E-05	0.0253	GCGCCGGCCCC
1	GCGCVTGCGCV	TEX22	+	85	95	3.00E-05	0.0253	GCGCCGGCCCC
1	GCGCVTGCGCV	HIST1H3J	-	90	100	3.00E-05	0.0253	GCGCCGGCCCC
1	GCGCVTGCGCV	RAI14	-	114	124	3.00E-05	0.0253	GCGCCGGCCCC
1	GCGCVTGCGCV	IRS1	-	118	128	3.00E-05	0.0253	GCGCCGGCCCC
1	GCGCVTGCGCV	VRK1	-	159	169	3.00E-05	0.0253	GCGCCGGCCCC

1	GCGCVTGCGCV	DIP2A	+	26	36	3.01E-05	0.0253	GCGCGGGCCCG
1	GCGCVTGCGCV	GSE1	-	81	91	3.01E-05	0.0253	GCGCGGGCCCG
1	GCGCVTGCGCV	CDKN1C	-	144	154	3.01E-05	0.0253	GCGCGGGCCCG
1	GCGCVTGCGCV	C8orf33	+	13	23	3.06E-05	0.0253	CCGCCCGCGCC
1	GCGCVTGCGCV	CLPTM1L	-	4	14	3.06E-05	0.0253	CCGCCCGCGCC
1	GCGCVTGCGCV	MTA1	+	19	29	3.06E-05	0.0253	CCGCCCGCGCC
1	GCGCVTGCGCV	C8orf33	+	24	34	3.06E-05	0.0253	CCGCCCGCGCC
1	GCGCVTGCGCV	NDUFB2-AS1	+	25	35	3.06E-05	0.0253	CCGCCCGCGCC
1	GCGCVTGCGCV	NOL4L	+	29	39	3.06E-05	0.0253	CCGCCCGCGCC
1	GCGCVTGCGCV	C8orf33	+	35	45	3.06E-05	0.0253	CCGCCCGCGCC
1	GCGCVTGCGCV	C8orf33	+	46	56	3.06E-05	0.0253	CCGCCCGCGCC
1	GCGCVTGCGCV	RP1-102K2.8	+	48	58	3.06E-05	0.0253	CCGCCCGCGCC
1	GCGCVTGCGCV	MEX3D	-	40	50	3.06E-05	0.0253	CCGCCCGCGCC
1	GCGCVTGCGCV	CYTH1	-	42	52	3.06E-05	0.0253	CCGCCCGCGCC
1	GCGCVTGCGCV	RP11-40E6.1	+	54	64	3.06E-05	0.0253	CCGCCCGCGCC
1	GCGCVTGCGCV	CDKN1C	-	45	55	3.06E-05	0.0253	GCCCCCGCGCA
1	GCGCVTGCGCV	C8orf33	+	57	67	3.06E-05	0.0253	CCGCCCGCGCC
1	GCGCVTGCGCV	MLLT4	-	50	60	3.06E-05	0.0253	CCGCCCGCGCC
1	GCGCVTGCGCV	HSPA9	-	57	67	3.06E-05	0.0253	CCGCCCGCGCC
1	GCGCVTGCGCV	C8orf33	+	68	78	3.06E-05	0.0253	CCGCCCGCGCC
1	GCGCVTGCGCV	MEX3D	-	58	68	3.06E-05	0.0253	CCGCCCGCGCC
1	GCGCVTGCGCV	SLC7A5	-	63	73	3.06E-05	0.0253	CCGCCCGCGCC
1	GCGCVTGCGCV	C8orf33	+	79	89	3.06E-05	0.0253	CCGCCCGCGCC
1	GCGCVTGCGCV	DIAPH2	-	75	85	3.06E-05	0.0253	GAGCATGCCCG
1	GCGCVTGCGCV	TCF3	-	75	85	3.06E-05	0.0253	CCGCCCGCGCC
1	GCGCVTGCGCV	SLX4	-	75	85	3.06E-05	0.0253	CCGCCCGCGCC
1	GCGCVTGCGCV	C8orf33	+	90	100	3.06E-05	0.0253	CCGCCCGCGCC

1	GCGCVTGCGCV	C8orf33	+	101	111	3.06E-05	0.0253	CCGCCCGCGCC
1	GCGCVTGCGCV	C8orf33	+	112	122	3.06E-05	0.0253	CCGCCCGCGCC
1	GCGCVTGCGCV	SH3BP2	-	102	112	3.06E-05	0.0253	CCGCCCGCGCC
1	GCGCVTGCGCV	THNSL1	-	103	113	3.06E-05	0.0253	GCCCCCGCGCA
1	GCGCVTGCGCV	AC011747.4	-	120	130	3.06E-05	0.0253	CCGCCCGCGCC
1	GCGCVTGCGCV	DOCK5	-	125	135	3.06E-05	0.0253	CCGCCCGCGCC
1	GCGCVTGCGCV	PSMA4	-	128	138	3.06E-05	0.0253	GCCCCCGCGCA
1	GCGCVTGCGCV	ARHGAP32	-	152	162	3.06E-05	0.0253	CCGCCCGCGCC
1	GCGCVTGCGCV	NCOA6	-	154	164	3.06E-05	0.0253	CCGCCCGCGCC
1	GCGCVTGCGCV	CROT	-	164	174	3.06E-05	0.0253	CCGCCCGCGCC
1	GCGCVTGCGCV	DOT1L	+	23	33	3.07E-05	0.0253	CCGCGCGCGCG
1	GCGCVTGCGCV	DOT1L	-	24	34	3.07E-05	0.0253	CCGCGCGCGCG
1	GCGCVTGCGCV	NSF	+	38	48	3.07E-05	0.0253	CCGCGCGCGCG
1	GCGCVTGCGCV	REL	-	30	40	3.07E-05	0.0253	CCGCGCGCGCG
1	GCGCVTGCGCV	C6orf48	+	56	66	3.07E-05	0.0253	CCGCGCGCGCG
1	GCGCVTGCGCV	AL353644.10	+	68	78	3.07E-05	0.0253	CCGCGCGCGCG
1	GCGCVTGCGCV	C6orf48	-	59	69	3.07E-05	0.0253	CCGCGCGCGCG
1	GCGCVTGCGCV	AL353644.10	-	69	79	3.07E-05	0.0253	CCGCGCGCGCG
1	GCGCVTGCGCV	KDELR2	+	133	143	3.07E-05	0.0253	CCGCGCGCGCG
1	GCGCVTGCGCV	ZDHHC14	+	140	150	3.07E-05	0.0253	CCGCGCGCGCG
1	GCGCVTGCGCV	NSF	-	138	148	3.07E-05	0.0253	CCGCGCGCGCG
1	GCGCVTGCGCV	MBD6	-	149	159	3.07E-05	0.0253	CCGCGCGCGCG
1	GCGCVTGCGCV	AGO2	-	166	176	3.07E-05	0.0253	CCGCGCGCGCG
1	GCGCVTGCGCV	ZFAS1	-	47	57	3.08E-05	0.0253	GCGGAGGCGCC
1	GCGCVTGCGCV	PPP1R7	-	75	85	3.08E-05	0.0253	GCGGAGGCGCC
1	GCGCVTGCGCV	NUDT15	-	182	192	3.08E-05	0.0253	GCGGAGGCGCC
1	GCGCVTGCGCV	PCBP2	+	35	45	3.12E-05	0.0255	CAGCCTGCGCC

1	GCGCVTGCGCV	GCH1	-	76	86	3.12E-05	0.0255	CAGCCTGCGCC
1	GCGCVTGCGCV	AGAP1	+	132	142	3.12E-05	0.0255	GCGCCTGCGAC
1	GCGCVTGCGCV	C8orf33	-	137	147	3.12E-05	0.0255	GCGCCTGCGAC
1	GCGCVTGCGCV	PARK7	+	6	16	3.15E-05	0.0256	GCACCGGCGCG
1	GCGCVTGCGCV	BRD4	+	88	98	3.15E-05	0.0256	CAGCGTGCGCG
1	GCGCVTGCGCV	TDRD12	+	120	130	3.15E-05	0.0256	CAGCGTGCGCG
1	GCGCVTGCGCV	HIVEP2	+	141	151	3.15E-05	0.0256	GCACCGGCGCG
1	GCGCVTGCGCV	NDUFV2	-	138	148	3.15E-05	0.0256	CAGCGTGCGCG
1	GCGCVTGCGCV	VMAC	+	190	200	3.15E-05	0.0256	GCACCGGCGCG
1	GCGCVTGCGCV	RP11-350G8.5	-	61	71	3.18E-05	0.0258	CCGCATGCCCA
1	GCGCVTGCGCV	ATP5E	+	12	22	3.23E-05	0.0261	GCGCGCGCCCA
1	GCGCVTGCGCV	SYT2	+	21	31	3.23E-05	0.0261	GCGCGCGCCCA
1	GCGCVTGCGCV	DMXL2	-	25	35	3.23E-05	0.0261	GCGCGCGCCCA
1	GCGCVTGCGCV	PSMA1	+	40	50	3.23E-05	0.0261	GCGCGCGCCCA
1	GCGCVTGCGCV	RPL32	+	125	135	3.25E-05	0.0262	GCCCTTGCGCG
1	GCGCVTGCGCV	ARL3	+	69	79	3.29E-05	0.0265	GCACTTGCGCA
1	GCGCVTGCGCV	NDUFA7	+	7	17	3.36E-05	0.0267	CCGGCTGCGCC
1	GCGCVTGCGCV	SERPINI1	+	43	53	3.36E-05	0.0267	CCGCAGGCGCC
1	GCGCVTGCGCV	KIAA1671	-	150	160	3.36E-05	0.0267	GCCGCTGCGCA
1	GCGCVTGCGCV	CDK13	-	169	179	3.36E-05	0.0267	CCGCAGGCGCC
1	GCGCVTGCGCV	IQSEC3	+	181	191	3.36E-05	0.0267	GCCCAGGCGCA
1	GCGCVTGCGCV	NDUFV2	+	45	55	3.38E-05	0.0267	GCCCCCGCGCG
1	GCGCVTGCGCV	TRAK1	+	46	56	3.38E-05	0.0267	GCCCCCGCGCG
1	GCGCVTGCGCV	LEPROTL1	+	68	78	3.38E-05	0.0267	GCCCCCGCGCG
1	GCGCVTGCGCV	UXS1	-	70	80	3.38E-05	0.0267	GCCCCCGCGCG
1	GCGCVTGCGCV	TEX30	-	82	92	3.38E-05	0.0267	CCGGGTGCGCG
1	GCGCVTGCGCV	KIF1B	+	114	124	3.38E-05	0.0267	GCCCCCGCGCG

1	GCGCVTGCGCV	ABTB1	+	135	145	3.38E-05	0.0267	CCGGGTGCGCG
1	GCGCVTGCGCV	TCF20	+	138	148	3.38E-05	0.0267	GCCCCCGCGCG
1	GCGCVTGCGCV	MAD2L2	-	146	156	3.38E-05	0.0267	CCGGGTGCGCG
1	GCGCVTGCGCV	UXS1	+	157	167	3.38E-05	0.0267	GCCCCCGCGCG
1	GCGCVTGCGCV	EXT1	-	164	174	3.38E-05	0.0267	GCCCCCGCGCG
1	GCGCVTGCGCV	AGAP1	-	178	188	3.38E-05	0.0267	GCCCCCGCGCG
1	GCGCVTGCGCV	AC114812.5	-	111	121	3.41E-05	0.0269	GCACCCGCGCA
1	GCGCVTGCGCV	RAN	+	32	42	3.47E-05	0.0273	GCGTCGGCGCA
1	GCGCVTGCGCV	SLC35E1P1	+	22	32	3.49E-05	0.0275	GCGCTTGCCCC
1	GCGCVTGCGCV	MICAL3	-	46	56	3.55E-05	0.0277	GAGCGGGCGCC
1	GCGCVTGCGCV	CDC42BPG	-	48	58	3.55E-05	0.0277	CCGCATGCCCG
1	GCGCVTGCGCV	SNHG15	+	68	78	3.55E-05	0.0277	CCGCCTGTGCG
1	GCGCVTGCGCV	CCT6A	+	80	90	3.55E-05	0.0277	GAGCGGGCGCC
1	GCGCVTGCGCV	TAF4	+	19	29	3.59E-05	0.0277	GCGCCCGCCCC
1	GCGCVTGCGCV	CYTH1	-	24	34	3.59E-05	0.0277	GCGCCCGCCCC
1	GCGCVTGCGCV	ATXN7L1	+	34	44	3.59E-05	0.0277	GCGCCCGCCCC
1	GCGCVTGCGCV	MRPL20	-	41	51	3.59E-05	0.0277	GCGGGTGCCCA
1	GCGCVTGCGCV	MGRN1	-	41	51	3.59E-05	0.0277	GCGCCCGCCCC
1	GCGCVTGCGCV	KCNK3	-	64	74	3.59E-05	0.0277	GCGCCCGCCCC
1	GCGCVTGCGCV	HMGCS1	-	66	76	3.59E-05	0.0277	GCGCCCGCCCC
1	GCGCVTGCGCV	ANKMY1	+	77	87	3.59E-05	0.0277	GCGCCCGCCCC
1	GCGCVTGCGCV	POFUT1	-	80	90	3.59E-05	0.0277	GCGCCCGCCCC
1	GCGCVTGCGCV	FAM83H	-	82	92	3.59E-05	0.0277	GCGCCCGCCCC
1	GCGCVTGCGCV	ANAPC16	-	2	12	3.63E-05	0.0277	GCGCGCGCCCG
1	GCGCVTGCGCV	AC093620.5	-	11	21	3.63E-05	0.0277	GCGCGCGCCCG
1	GCGCVTGCGCV	PXN	-	15	25	3.63E-05	0.0277	CCCCCTGCGCA
1	GCGCVTGCGCV	SLX4	+	31	41	3.63E-05	0.0277	GCGCGCGCCCG

1	GCGCVTGCG CV	AFF1	-	27	37	3.63E-05	0.0277	GCGCGCGCCC G
1	GCGCVTGCG CV	B3GALNT1	+	43	53	3.63E-05	0.0277	GCGCGCGCCC G
1	GCGCVTGCG CV	PSMA1	-	37	47	3.63E-05	0.0277	GCGCGCGCCC G
1	GCGCVTGCG CV	C6orf48	+	48	58	3.63E-05	0.0277	GCGCGCGCCC G
1	GCGCVTGCG CV	GPATCH8	+	50	60	3.63E-05	0.0277	GCGCGCGCCC G
1	GCGCVTGCG CV	AC132812.1	+	54	64	3.63E-05	0.0277	GCGCGCGCCC G
1	GCGCVTGCG CV	AGO4	-	64	74	3.63E-05	0.0277	GCGCGCGCCC G
1	GCGCVTGCG CV	TMEM106C	+	82	92	3.63E-05	0.0277	GCGCGCGCCC G
1	GCGCVTGCG CV	RAB28	+	88	98	3.63E-05	0.0277	GCGCGCGCCC G
1	GCGCVTGCG CV	PPP5D1	-	79	89	3.63E-05	0.0277	GCGCGCGCCC G
1	GCGCVTGCG CV	KMT2C	+	106	116	3.63E-05	0.0277	GCGCGCGCCC G
1	GCGCVTGCG CV	PXN	-	115	125	3.63E-05	0.0277	GCGCGCGCCC G
1	GCGCVTGCG CV	FAM63B	+	145	155	3.63E-05	0.0277	GCGCGCGCCC G
1	GCGCVTGCG CV	LRP1-AS	-	17	27	3.67E-05	0.0279	GAGCCTGCCC C
1	GCGCVTGCG CV	NDUFS6	+	108	118	3.67E-05	0.0279	GAGCCTGCCC C
1	GCGCVTGCG CV	TAF4	+	48	58	3.72E-05	0.0282	GCCCAGGCGC G
1	GCGCVTGCG CV	UNC50	-	53	63	3.72E-05	0.0282	GCGGACGCG CC
1	GCGCVTGCG CV	USP2	+	84	94	3.72E-05	0.0282	GCCCAGGCGC G
1	GCGCVTGCG CV	LRP4	+	104	114	3.72E-05	0.0282	GCCCAGGCGC G
1	GCGCVTGCG CV	GDF9	-	32	42	3.77E-05	0.0282	GCGCCTGTCC A
1	GCGCVTGCG CV	THNSL1	+	84	94	3.77E-05	0.0282	GCACAGGCG CA
1	GCGCVTGCG CV	FAM195B	-	85	95	3.77E-05	0.0282	GCACAGGCG CA
1	GCGCVTGCG CV	PNO1	+	145	155	3.77E-05	0.0282	GCAGCTGCGC A
1	GCGCVTGCG CV	DDX3X	-	30	40	3.78E-05	0.0282	GCGGGGGCG CC
1	GCGCVTGCG CV	ETV6	+	50	60	3.78E-05	0.0282	GCGGGGGCG CC
1	GCGCVTGCG CV	ARHGAP32	-	40	50	3.78E-05	0.0282	GCGGGGGCG CC
1	GCGCVTGCG CV	TMEM223	+	59	69	3.78E-05	0.0282	GCGGGGGCG CC

1	GCGCVTGCG CV	COBL	-	50	60	3.78E-05	0.0282	GCGGGGGCG CC
1	GCGCVTGCG CV	COBL	-	94	104	3.78E-05	0.0282	GCGGGGGCG CC
1	GCGCVTGCG CV	GATS	+	131	141	3.78E-05	0.0282	GCACCCGCGC G
1	GCGCVTGCG CV	CTC-507E2.2	+	144	154	3.78E-05	0.0282	GCGGGGGCG CC
1	GCGCVTGCG CV	PIP5K1C	-	161	171	3.78E-05	0.0282	GCGGGGGCG CC
1	GCGCVTGCG CV	ISOC1	+	171	181	3.78E-05	0.0282	GCGGGGGCG CC
1	GCGCVTGCG CV	DIP2A	-	165	175	3.78E-05	0.0282	GCACCCGCGC G
1	GCGCVTGCG CV	DNAJB9	+	64	74	3.80E-05	0.0283	GAGGATGCG CC
1	GCGCVTGCG CV	WRB	+	126	136	3.82E-05	0.0284	GCGTCGGCGC G
1	GCGCVTGCG CV	MYDGF	+	165	175	3.82E-05	0.0284	GCGTCGGCGC G
1	GCGCVTGCG CV	DDX18	+	138	148	3.88E-05	0.0288	GCGCACGTGC G
1	GCGCVTGCG CV	ZDHHC14	+	26	36	3.92E-05	0.029	GCGCCGGTGC C
1	GCGCVTGCG CV	ST6GALNAC4	+	33	43	3.92E-05	0.029	GCGCAGGCCC C
1	GCGCVTGCG CV	BNIP3	+	90	100	3.92E-05	0.029	GCGCAGGCCC C
1	GCGCVTGCG CV	SAP130	-	85	95	3.92E-05	0.029	GCGCAGGCCC C
1	GCGCVTGCG CV	ZYX	-	22	32	3.94E-05	0.029	GCGGGTGCCC G
1	GCGCVTGCG CV	SMDT1	+	146	156	3.94E-05	0.029	GCGGGTGCCC G
1	GCGCVTGCG CV	KCNK3	+	157	167	3.94E-05	0.029	GCGGGTGCCC G
1	GCGCVTGCG CV	DIP2A	+	166	176	3.94E-05	0.029	GCGCGGGTGC G
1	GCGCVTGCG CV	YY1	+	41	51	3.99E-05	0.0293	CCGCACGCGC C
1	GCGCVTGCG CV	SOX13	+	52	62	3.99E-05	0.0293	CCCCCTGCGC G
1	GCGCVTGCG CV	AL353644.4	-	63	73	3.99E-05	0.0293	CCGCACGCGC C
1	GCGCVTGCG CV	MIR4523	-	70	80	3.99E-05	0.0293	GCCCACGCGC A
1	GCGCVTGCG CV	NRARP	-	5	15	4.03E-05	0.0295	ACGCGCGCGC C
1	GCGCVTGCG CV	ZNF516	+	21	31	4.06E-05	0.0296	GCCCGGGCGC A
1	GCGCVTGCG CV	BRD1	+	31	41	4.06E-05	0.0296	CCGCGGGCGC C
1	GCGCVTGCG CV	SKI	+	79	89	4.06E-05	0.0296	CCGCGGGCGC C

1	GCGCVTGCGCV	TRIO	+	105	115	4.06E-05	0.0296	CCGCGGGCGCC
1	GCGCVTGCGCV	SLC12A6	-	145	155	4.06E-05	0.0296	CCGCGGGCGCC
1	GCGCVTGCGCV	DLGAP4	-	148	158	4.06E-05	0.0296	CCGCGGGCGCC

Supplementary Table 11. FIMO motif scanning using JASPAR motif matrices using default p-value cutoff 1e-4 (YY1)

YY1 motif MA0095.2	Alt ID	Sequence Name	Strand	Start	End	p-value	q-value	Matched Sequence
1	MAAVATGGCBGM	RP11-512M8.5	+	12	23	3.79E-08	0.00218	CAAGATGGCGGC
1	MAAVATGGCBGM	SPCS2	+	82	93	3.79E-08	0.00218	CAAGATGGCGGC
1	MAAVATGGCBGM	SPCS2P4	+	97	108	3.79E-08	0.00218	CAAGATGGCGGC
1	MAAVATGGCBGM	KMT2B	+	97	108	3.79E-08	0.00218	CAAGATGGCGGC
1	MAAVATGGCBGM	UBA52	+	114	125	3.79E-08	0.00218	CAAGATGGCGGC
1	MAAVATGGCBGM	YARS2	+	125	136	3.79E-08	0.00218	CAAGATGGCGGC
1	MAAVATGGCBGM	NDUFS4	+	127	138	3.79E-08	0.00218	CAAGATGGCGGC
1	MAAVATGGCBGM	PDHB	+	145	156	3.79E-08	0.00218	CAAGATGGCGGC
1	MAAVATGGCBGM	GLG1	+	147	158	3.79E-08	0.00218	CAAGATGGCGGC
1	MAAVATGGCBGM	PPP1R8	+	155	166	3.79E-08	0.00218	CAAGATGGCGGC
1	MAAVATGGCBGM	TJAP1	+	169	180	3.79E-08	0.00218	CAAGATGGCGGC
1	MAAVATGGCBGM	ZC3HC1	+	182	193	3.79E-08	0.00218	CAAGATGGCGGC
1	MAAVATGGCBGM	RPN1	-	41	52	3.79E-08	0.00218	CAAGATGGCGGC
1	MAAVATGGCBGM	SF3A1	-	51	62	3.79E-08	0.00218	CAAGATGGCGGC
1	MAAVATGGCBGM	PRRC2C	-	77	88	3.79E-08	0.00218	CAAGATGGCGGC
1	MAAVATGGCBGM	EP300	-	93	104	3.79E-08	0.00218	CAAGATGGCGGC
1	MAAVATGGCBGM	SRP9	-	114	125	3.79E-08	0.00218	CAAGATGGCGGC
1	MAAVATGGCBGM	CREBBP	-	130	141	3.79E-08	0.00218	CAAGATGGCGGC
1	MAAVATGGCBGM	NDUFS6	+	48	59	8.41E-08	0.00348	CAAAATGGCGGC
1	MAAVATGGCBGM	KDM2A	+	115	126	8.41E-08	0.00348	CAAAATGGCGGC

1	MAAVATGGC BGM	RBX1	+	149	160	8.41 E-08	0.003 48	CAAAATGGC GGC
1	MAAVATGGC BGM	CIAPIN1	-	16	27	8.41 E-08	0.003 48	CAAAATGGC GGC
1	MAAVATGGC BGM	ANAPC7	-	17	28	8.41 E-08	0.003 48	CAAAATGGC GGC
1	MAAVATGGC BGM	RBM23	-	42	53	8.41 E-08	0.003 48	CAAAATGGC GGC
1	MAAVATGGC BGM	ENSA	-	111	122	8.41 E-08	0.003 48	CAAAATGGC GGC
1	MAAVATGGC BGM	ZBTB40	+	123	134	1.22 E-07	0.004 51	CAAGATGGC CGC
1	MAAVATGGC BGM	MARK3	-	113	124	1.22 E-07	0.004 51	CAAGATGGC CGC
1	MAAVATGGC BGM	ESD	-	166	177	1.22 E-07	0.004 51	CAAGATGGC CGC
1	MAAVATGGC BGM	COMMD9	+	134	145	1.68 E-07	0.005 62	CAAGATGGC TGC
1	MAAVATGGC BGM	DIDO1	+	178	189	1.68 E-07	0.005 62	CAAGATGGC TGC
1	MAAVATGGC BGM	BTF3L4P1	-	34	45	1.68 E-07	0.005 62	CAAGATGGC TGC
1	MAAVATGGC BGM	SRCAP	-	16	27	2.14 E-07	0.006 73	CAAAATGGC CGC
1	MAAVATGGC BGM	ZNF814	-	32	43	2.14 E-07	0.006 73	CAAAATGGC CGC
1	MAAVATGGC BGM	COX8A	-	108	119	2.71 E-07	0.008 24	CAAAATGGC TGC
1	MAAVATGGC BGM	RRM1	+	100	111	3.09 E-07	0.008 41	CAACATGGC GGC
1	MAAVATGGC BGM	MRPS14	+	114	125	3.09 E-07	0.008 41	CAACATGGC GGC
1	MAAVATGGC BGM	MYDGF	+	133	144	3.09 E-07	0.008 41	CAACATGGC GGC
1	MAAVATGGC BGM	RP5-901A4.1	-	51	62	3.09 E-07	0.008 41	CAACATGGC GGC
1	MAAVATGGC BGM	UBE2V2P 1	+	97	108	3.55 E-07	0.008 75	CAAGATGGC AGC
1	MAAVATGGC BGM	HSD17B10	+	125	136	3.55 E-07	0.008 75	CAAGATGGC AGC
1	MAAVATGGC BGM	COIL	+	132	143	3.55 E-07	0.008 75	CAAGATGGC AGC
1	MAAVATGGC BGM	MRPL20	+	135	146	3.55 E-07	0.008 75	CAAGATGGC AGC
1	MAAVATGGC BGM	KIAA1328	+	30	41	4.01 E-07	0.009 23	CAAGATGGC GGA

1	MAAVATGGCBGM	ATL2	+	122	133	4.01E-07	0.00923	CAAGATGGCGGA
1	MAAVATGGCBGM	UBR1	+	188	199	4.01E-07	0.00923	CAAGATGGCGGA
1	MAAVATGGCBGM	TRMT12	+	10	21	4.85E-07	0.00985	AAAGATGGCGGC
1	MAAVATGGCBGM	NDUFA9	+	29	40	4.85E-07	0.00985	AAAGATGGCGGC
1	MAAVATGGCBGM	TAF4	+	97	108	4.85E-07	0.00985	AAAGATGGCGGC
1	MAAVATGGCBGM	LMAN2L	+	121	132	4.85E-07	0.00985	AAAGATGGCGGC
1	MAAVATGGCBGM	MRPL22	+	138	149	4.85E-07	0.00985	AAAGATGGCGGC
1	MAAVATGGCBGM	PSMB6	+	148	159	4.85E-07	0.00985	AAAGATGGCGGC
1	MAAVATGGCBGM	MPDU1	+	4	15	5.69E-07	0.0103	CAAGATGGCGCC
1	MAAVATGGCBGM	PSMB4	+	44	55	5.69E-07	0.0103	CAACATGGCTGC
1	MAAVATGGCBGM	URM1	+	159	170	5.69E-07	0.0103	CAACATGGCTGC
1	MAAVATGGCBGM	PTRHD1	+	44	55	6.07E-07	0.0103	GAAGATGGCGGC
1	MAAVATGGCBGM	SLIRP	+	88	99	6.07E-07	0.0103	GAAGATGGCGGC
1	MAAVATGGCBGM	PSMB7	+	116	127	6.07E-07	0.0103	GAAGATGGCGGC
1	MAAVATGGCBGM	MRPL50	+	119	130	6.07E-07	0.0103	GAAGATGGCGGC
1	MAAVATGGCBGM	ZCCHC4	+	122	133	6.07E-07	0.0103	GAAGATGGCGGC
1	MAAVATGGCBGM	NGDN	+	125	136	6.07E-07	0.0103	GAAGATGGCGGC
1	MAAVATGGCBGM	PRDX3	+	140	151	6.07E-07	0.0103	GAAGATGGCGGC
1	MAAVATGGCBGM	RP11-98L12.2	+	95	106	6.63E-07	0.0107	CAAAATGGCAGC
1	MAAVATGGCBGM	RP11-21L23.2	+	102	113	6.63E-07	0.0107	CAAAATGGCAGC
1	MAAVATGGCBGM	PHB2	-	46	57	6.63E-07	0.0107	CAAAATGGCAGC
1	MAAVATGGCBGM	CCDC167	+	25	36	7.56E-07	0.0117	CAAGATGGCGAC
1	MAAVATGGCBGM	NECAP1	+	122	133	7.56E-07	0.0117	CAAGATGGCGAC

1	MAAVATGGC BGM	SLC25A5P 6	+	165	176	7.56 E-07	0.011 7	CAAGATGGC GGT
1	MAAVATGGC BGM	PSMC5	-	1	12	8.12 E-07	0.012	CAAAATGGC GGA
1	MAAVATGGC BGM	KDM3A	+	139	150	8.12 E-07	0.012	CAAAATGGC GGA
1	MAAVATGGC BGM	GPATCH8	+	157	168	8.12 E-07	0.012	CAAAATGGC GGA
1	MAAVATGGC BGM	AP000304. 12	+	8	19	8.58 E-07	0.012 3	CAAGATGGC CGA
1	MAAVATGGC BGM	RP11- 689C9.1	+	11	22	8.58 E-07	0.012 3	CAAGATGGC CGA
1	MAAVATGGC BGM	ERH	-	28	39	1.02 E-06	0.014 4	AAAAATGGC GGC
1	MAAVATGGC BGM	RPN1	-	96	107	1.06 E-06	0.014 7	AAAGATGGC CGC
1	MAAVATGGC BGM	RPA2	+	128	139	1.06 E-06	0.014 7	AAAGATGGC CGC
1	MAAVATGGC BGM	MAML1	+	93	104	1.20 E-06	0.016	TAAGATGGC GGC
1	MAAVATGGC BGM	JPX_1	+	119	130	1.20 E-06	0.016	TAAGATGGC GGC
1	MAAVATGGC BGM	SLC17A5	+	180	191	1.20 E-06	0.016	TAAGATGGC GGC
1	MAAVATGGC BGM	RC3H1	+	96	107	1.24 E-06	0.016 2	CAAGATGGC CCC
1	MAAVATGGC BGM	TMEM126 A	+	122	133	1.30 E-06	0.016 8	AAAGATGGC TGC
1	MAAVATGGC BGM	KMT2E	-	13	24	1.34 E-06	0.016 9	GAAAATGGC GGC
1	MAAVATGGC BGM	COPS6	+	134	145	1.34 E-06	0.016 9	GAAAATGGC GGC
1	MAAVATGGC BGM	DYRK1B	-	146	157	1.58 E-06	0.019 5	CAAAATGGC GAC
1	MAAVATGGC BGM	RNF139	+	163	174	1.58 E-06	0.019 5	CAAGATGGT GGC
1	MAAVATGGC BGM	SGSM2	+	16	27	1.72 E-06	0.020 5	GAAGATGGC TGC
1	MAAVATGGC BGM	ISCA2	+	52	63	1.72 E-06	0.020 5	GAAGATGGC TGC
1	MAAVATGGC BGM	RBM8A	+	56	67	1.72 E-06	0.020 5	CAAGATGGC CAC
1	MAAVATGGC BGM	MIR632	-	135	146	1.78 E-06	0.020 9	CAAAATGGC CGA
1	MAAVATGGC BGM	TMED9	+	154	165	1.89 E-06	0.022	CAAGATGGC TGT

1	MAAVATGGC BGM	TM2D3	+	127	138	1.98 E-06	0.022 7	CAAGATGGC GGG
1	MAAVATGGC BGM	ATP5J2-PTCD1	+	53	64	2.10 E-06	0.023 9	CAAAATGGC GTC
1	MAAVATGGC BGM	DHX15	-	106	117	2.36 E-06	0.026 6	TAAAATGGC GGC
1	MAAVATGGC BGM	KARS	+	8	19	2.56 E-06	0.028 2	CAAGATGGA CGC
1	MAAVATGGC BGM	HNRNPC	-	165	176	2.56 E-06	0.028 2	CAAAATGGC CCC
1	MAAVATGGC BGM	UQCR10	+	127	138	2.67 E-06	0.028 8	AAACATGGC GGC
1	MAAVATGGC BGM	SCMH1	+	143	154	2.67 E-06	0.028 8	AAACATGGC GGC
1	MAAVATGGC BGM	FAM193B	-	73	84	2.76 E-06	0.029 5	CAATATGGC GGC
1	MAAVATGGC BGM	PLEKHA7	+	39	50	3.34 E-06	0.034 9	GAACATGGC GGC
1	MAAVATGGC BGM	ISOC1	+	113	124	3.34 E-06	0.034 9	GAACATGGC GGC
1	MAAVATGGC BGM	AC108004. 3	-	20	31	3.76 E-06	0.039	AAAGATGGC AGC
1	MAAVATGGC BGM	PSMA2	+	62	73	4.00 E-06	0.040 2	AAAGATGGC GGA
1	MAAVATGGC BGM	RPS15A	-	55	66	4.00 E-06	0.040 2	AAAGATGGC GGA
1	MAAVATGGC BGM	RPL23AP6 4	+	97	108	4.00 E-06	0.040 2	CAAGATGGC ACC
1	MAAVATGGC BGM	RP4-761J14.8	-	5	16	4.11 E-06	0.040 6	CAAAATGGC TTC
1	MAAVATGGC BGM	COA3	+	107	118	4.42 E-06	0.040 6	CAACATGGC GTC
1	MAAVATGGC BGM	BCCIP	+	109	120	4.42 E-06	0.040 6	CAACATGGC GTC
1	MAAVATGGC BGM	HECTD4	+	120	131	4.42 E-06	0.040 6	CAACATGGC GTC
1	MAAVATGGC BGM	RLIMP1	+	181	192	4.42 E-06	0.040 6	GAAGATGGC AGC
1	MAAVATGGC BGM	SMG6	+	48	59	4.51 E-06	0.040 6	GAAGATGGC GGA
1	MAAVATGGC BGM	RPL13A	+	73	84	4.51 E-06	0.040 6	GAAGATGGC GGA
1	MAAVATGGC BGM	PFDN2	+	82	93	4.51 E-06	0.040 6	GAAGATGGC GGA
1	MAAVATGGC BGM	RP3-340B19.2	-	76	87	4.51 E-06	0.040 6	GAAGATGGC GGA

1	MAAVATGGC BGM	RPL13AP5	+	97	108	4.51 E-06	0.040 6	GAAGATGGC GGA
1	MAAVATGGC BGM	HIST1H2A C	-	104	115	4.51 E-06	0.040 6	AAACATGGC CGC
1	MAAVATGGC BGM	RPS11	+	189	200	4.51 E-06	0.040 6	GAAGATGGC GGA
1	MAAVATGGC BGM	RPS3	+	159	170	4.81 E-06	0.042 9	CAAGATGGC AGT
1	MAAVATGGC BGM	KBTBD4	-	17	28	4.90 E-06	0.043	TAACATGGC GGC
1	MAAVATGGC BGM	ABT1	+	128	139	4.90 E-06	0.043	CAACATGGA GGC
1	MAAVATGGC BGM	MRPL47	+	62	73	5.22 E-06	0.045 4	AAACATGGC TGC
1	MAAVATGGC BGM	PSMD8	-	33	44	5.75 E-06	0.048 5	GAACATGGC TGC
1	MAAVATGGC BGM	UFC1	+	89	100	5.75 E-06	0.048 5	CAAAATGGC CGG
1	MAAVATGGC BGM	CTD-2262B20.1	+	125	136	5.75 E-06	0.048 5	GAAGATGGC GCC
1	MAAVATGGC BGM	YWHAQP 5	+	136	147	5.75 E-06	0.048 5	CAACATGGC CAC
1	MAAVATGGC BGM	TMX2	+	116	127	5.94 E-06	0.049 6	AAAGATGGC GGT
1	MAAVATGGC BGM	ZNF621	+	133	144	6.02 E-06	0.049 9	CGAGATGGC GGC
1	MAAVATGGC BGM	RAB28	+	146	157	6.51 E-06	0.053 1	CAAGATGGC GCT
1	MAAVATGGC BGM	RPL39L	-	139	150	6.51 E-06	0.053 1	CAACATGGC TGT
1	MAAVATGGC BGM	PSMB5	+	107	118	6.60 E-06	0.053 4	AAAGATGGC CGA
1	MAAVATGGC BGM	TIMM17A	+	133	144	6.81 E-06	0.053 7	CAAGATGGA GGA
1	MAAVATGGC BGM	RP11-411B6.6	+	27	38	7.00 E-06	0.053 7	GAAGATGGC GGT
1	MAAVATGGC BGM	ZNFX1-AS1_2	+	37	48	7.00 E-06	0.053 7	GAAGATGGC GGT
1	MAAVATGGC BGM	INTS7	+	48	59	7.00 E-06	0.053 7	GAAGATGGC GGT
1	MAAVATGGC BGM	COMMD1 0	+	59	70	7.00 E-06	0.053 7	GAAGATGGC GGT
1	MAAVATGGC BGM	ING5	+	128	139	7.00 E-06	0.053 7	GAAGATGGC GAC
1	MAAVATGGC BGM	ARHGAP1 9	+	143	154	7.00 E-06	0.053 7	GAAGATGGC GAC

1	MAAVATGGCBGM	RP11-571F15.2	-	44	55	8.00E-06	0.061	CAAGATGGTGGA
1	MAAVATGGCBGM	NDUFA7	+	35	46	8.21E-06	0.0621	GAAGATGGCGTC
1	MAAVATGGCBGM	ANXA7	-	113	124	8.41E-06	0.063	CAAGATGGAGCC
1	MAAVATGGCBGM	RPL6P27	+	102	113	8.46E-06	0.063	CAAGATGGCAGG
1	MAAVATGGCBGM	AL590762.11	+	109	120	8.90E-06	0.0659	GAAGATGGAGGC
1	MAAVATGGCBGM	COX6A1	+	134	145	9.14E-06	0.0671	AAAAATGGCGGT
1	MAAVATGGCBGM	TMEM39A	+	17	28	9.35E-06	0.0682	AAAGATGGCCAC
1	MAAVATGGCBGM	DYRK1B	-	42	53	9.44E-06	0.0684	CGAGATGGCCGC
1	MAAVATGGCBGM	ATP5I	+	88	99	9.96E-06	0.0707	CAAGATGGTGCC
1	MAAVATGGCBGM	NUP205	+	123	134	9.96E-06	0.0707	TAAGATGGCGAC
1	MAAVATGGCBGM	KANSL2	+	154	165	9.96E-06	0.0707	TAAGATGGCGGT
1	MAAVATGGCBGM	PSMB1	-	146	157	1.07E-05	0.0752	CGAGATGGCTGC
1	MAAVATGGCBGM	RP11-730A19.5	+	28	39	1.10E-05	0.0767	GAAGATGGTGGC
1	MAAVATGGCBGM	XYLT1	+	182	193	1.10E-05	0.0767	GAAGATGGTGGC
1	MAAVATGGCBGM	DGKD	+	109	120	1.16E-05	0.0794	CAGCATGGCGGC
1	MAAVATGGCBGM	C1orf43	-	63	74	1.16E-05	0.0794	AAACATGGCGGA
1	MAAVATGGCBGM	RPL35A	-	83	94	1.19E-05	0.0811	CAAAATGGTAGC
1	MAAVATGGCBGM	RP11-846F4.6	-	70	81	1.23E-05	0.0823	TAAGATGGCTGA
1	MAAVATGGCBGM	PSMD8	-	125	136	1.23E-05	0.0823	CAAGATGGTGAC
1	MAAVATGGCBGM	SENP3-EIF4A1	+	89	100	1.24E-05	0.0823	CAAGATGGAGTC
1	MAAVATGGCBGM	RFT1	-	93	104	1.24E-05	0.0823	TAAGATGGCGTC
1	MAAVATGGCBGM	SFSWAP	-	104	115	1.28E-05	0.0847	CAAAATGGTGGA
1	MAAVATGGCBGM	DOCK5	+	178	189	1.33E-05	0.0872	GAACATGGCGGA

1	MAAVATGGC BGM	DOLK	+	98	109	1.39 E-05	0.089 4	CAAGATGGC GAG
1	MAAVATGGC BGM	RP11-8P13.1	-	20	31	1.39 E-05	0.089 4	CAAGATGGC GAG
1	MAAVATGGC BGM	MNT	-	153	164	1.39 E-05	0.089 4	CAAGATGGC GAG
1	MAAVATGGC BGM	RP11-640A1.4	+	97	108	1.44 E-05	0.090 1	CAAGATGGT GTC
1	MAAVATGGC BGM	UFC1	+	188	199	1.45 E-05	0.090 1	CAACATGGC GAA
1	MAAVATGGC BGM	RASL11A	-	43	54	1.45 E-05	0.090 1	CAACATGGC GAA
1	MAAVATGGC BGM	Z83851.4	-	86	97	1.45 E-05	0.090 1	GAAAATGGA GGC
1	MAAVATGGC BGM	CTA-243E7.4	-	111	122	1.45 E-05	0.090 1	CAACATGGC GAA
1	MAAVATGGC BGM	RP11-894P9.1	-	115	126	1.45 E-05	0.090 1	CAACATGGC GAA
1	MAAVATGGC BGM	RPL7P14	+	97	108	1.51 E-05	0.092 9	AAAGATGGC AGA
1	MAAVATGGC BGM	RP11-243J16.7	+	140	151	1.58 E-05	0.096 5	CAAAATGGA GGT
1	MAAVATGGC BGM	AC114812. 9	+	9	20	1.65 E-05	0.099 1	GAAAATGGC TCC
1	MAAVATGGC BGM	NDUFB5	+	68	79	1.65 E-05	0.099 1	TAAGATGGC CAC
1	MAAVATGGC BGM	CCDC175	+	153	164	1.65 E-05	0.099 1	CAAAATGGC CCT
1	MAAVATGGC BGM	NSL1	+	61	72	1.73 E-05	0.103	AAAAATGGC GGG
1	MAAVATGGC BGM	RPL13AP5	-	158	169	1.73 E-05	0.103	CACGATGGC CGC
1	MAAVATGGC BGM	HMBOX1	+	110	121	1.78 E-05	0.105	CAATATGGC GGT
1	MAAVATGGC BGM	TMED10	+	51	62	1.86 E-05	0.108	CAAAATGGC GCG
1	MAAVATGGC BGM	RP11-8P13.1	-	110	121	1.86 E-05	0.108	AAAAATGGC CTC
1	MAAVATGGC BGM	CSE1L	-	169	180	1.86 E-05	0.108	CAAAATGGC GCG
1	MAAVATGGC BGM	NPM3	+	120	131	1.87 E-05	0.108	CAGCATGGC CGC
1	MAAVATGGC BGM	MT-TY	+	15	26	1.90 E-05	0.108	TAAAATGGC TGA
1	MAAVATGGC BGM	TMEM126 B	+	132	143	1.90 E-05	0.108	CAAAATGGT GGT

1	MAAVATGGC BGM	MTCO1P1 1	-	74	85	1.90 E-05	0.108	TAAAATGGC TGA
1	MAAVATGGC BGM	MRFAP1L 1	-	116	127	1.94 E-05	0.11	CAAAATGGA GTC
1	MAAVATGGC BGM	RP11-420B22.1	-	43	54	2.03 E-05	0.115	AAAAATGGC TTC
1	MAAVATGGC BGM	TWISTNB	+	168	179	2.07 E-05	0.116	CAGCATGGC TGC
1	MAAVATGGC BGM	AC011816. 1	+	97	108	2.13 E-05	0.119	GAAAATGGC CTC
1	MAAVATGGC BGM	FOXK1	+	97	108	2.15 E-05	0.119	GAACATGGC CGA
1	MAAVATGGC BGM	KB-1247B1.1	+	97	108	2.19 E-05	0.121	CAAGATGGT GGG
1	MAAVATGGC BGM	AC007283. 5	+	36	47	2.27 E-05	0.125	CGAGATGGC AGC
1	MAAVATGGC BGM	AL132772. 1	-	39	50	2.29 E-05	0.125	CAAGATGGT CTC
1	MAAVATGGC BGM	CTD-2215L10.1	-	22	33	2.33 E-05	0.126	CAATATGGA GGC
1	MAAVATGGC BGM	RPL24P4	-	103	114	2.40 E-05	0.129	AAAGATGGC GAA
1	MAAVATGGC BGM	RPL24	-	172	183	2.40 E-05	0.129	AAAGATGGC GAA
1	MAAVATGGC BGM	HNRNPM P1	+	130	141	2.48 E-05	0.132	CAAGATGGA AGA
1	MAAVATGGC BGM	COX7B	-	120	131	2.48 E-05	0.132	CAAAATGGC TTT
1	MAAVATGGC BGM	RPL29P14	-	34	45	2.53 E-05	0.133	GAAAATGGA TGC
1	MAAVATGGC BGM	RP11-677I18.3	-	60	71	2.53 E-05	0.133	GAAAATGGA TGC
1	MAAVATGGC BGM	RPL21P12 3	-	73	84	2.57 E-05	0.134	GAAGATGGC AGT
1	MAAVATGGC BGM	SUCLA2P 1	-	107	118	2.58 E-05	0.134	GAACATGGA GGC
1	MAAVATGGC BGM	RPS27	+	28	39	2.61 E-05	0.134	AAACATGGT GGC
1	MAAVATGGC BGM	RP5-849H19.2	-	101	112	2.61 E-05	0.134	CACAATGGC CGC
1	MAAVATGGC BGM	RP11-391L3.5	+	47	58	2.65 E-05	0.136	AAACATGGC CAC
1	MAAVATGGC BGM	NPEPPS	-	105	116	2.71 E-05	0.138	TAAAATGGC TGT
1	MAAVATGGC BGM	RPN2	-	17	28	2.79 E-05	0.14	CAACATGGA GGT

1	MAAVATGGC BGM	RP11-512F24.1	+	97	108	2.79 E-05	0.14	CAAAATGGA GGG
1	MAAVATGGC BGM	OSTC	+	169	180	2.79 E-05	0.14	CAACATGGA GAC
1	MAAVATGGC BGM	CEP350	+	29	40	2.82 E-05	0.141	CAACATGGC CCT
1	MAAVATGGC BGM	RP4-781K5.2	-	108	119	2.91 E-05	0.145	AAACATGGC TGT
1	MAAVATGGC BGM	PSMC4	+	41	52	2.94 E-05	0.145	CGAGATGGC GCC
1	MAAVATGGC BGM	RP11-314D7.2	-	54	65	2.94 E-05	0.145	CGAGATGGC GCC
1	MAAVATGGC BGM	MTND4P23	+	75	86	2.98 E-05	0.146	CAATATGGC TAC
1	MAAVATGGC BGM	RP11-378E13.3	-	182	193	2.98 E-05	0.146	GAACATGGT GGC
1	MAAVATGGC BGM	ZNF785	-	143	154	3.04 E-05	0.147	CAAGATGGA ACC
1	MAAVATGGC BGM	NFAT5	-	166	177	3.04 E-05	0.147	AAAGATGGA GGA
1	MAAVATGGC BGM	HINT1	+	51	62	3.13 E-05	0.151	AAGGATGGC TGC
1	MAAVATGGC BGM	RPL23AP64	+	44	55	3.19 E-05	0.153	CAACATGGT GAC
1	MAAVATGGC BGM	RP11-21J18.1	-	3	14	3.32 E-05	0.158	GAACATGGC GGG
1	MAAVATGGC BGM	SRSF3	-	109	120	3.50 E-05	0.166	CGAAATGGC GGA
1	MAAVATGGC BGM	AC094019.4	-	71	82	3.67 E-05	0.173	GAAGATGGC GAT
1	MAAVATGGC BGM	RP11-216N14.7	+	99	110	3.72 E-05	0.174	AAAAATGGC ATC
1	MAAVATGGC BGM	RPL39	-	130	141	3.72 E-05	0.174	CACGATGGC GGA
1	MAAVATGGC BGM	TEKT4P2	-	87	98	3.78 E-05	0.177	GAAAATGGC GAA
1	MAAVATGGC BGM	YARS2	-	135	146	3.80 E-05	0.177	CAAGATGGG CGC
1	MAAVATGGC BGM	RP11-709D24.6	+	14	25	3.84 E-05	0.178	CAGAATGGC TGA
1	MAAVATGGC BGM	AC011816.1	+	5	16	3.90 E-05	0.178	CAACATGGC ACA
1	MAAVATGGC BGM	MTND5P11	+	14	25	3.90 E-05	0.178	CAACATGGC TTT
1	MAAVATGGC BGM	MT-TS2	+	44	55	3.90 E-05	0.178	CAACATGGC TTT

1	MAAVATGGCBGM	FUNDC2	+	76	87	3.97E-05	0.179	CAGGATGGCCTC
1	MAAVATGGCBGM	PSMB5	+	135	146	3.97E-05	0.179	CAACATGGACGT
1	MAAVATGGCBGM	RPL15P3	+	97	108	4.07E-05	0.182	CAAGATGGGTGC
1	MAAVATGGCBGM	LINC01420	-	124	135	4.07E-05	0.182	TGAGATGGCGGC
1	MAAVATGGCBGM	WDR38	+	82	93	4.09E-05	0.183	CACCATGGCCGC
1	MAAVATGGCBGM	SEC61G	+	13	24	4.17E-05	0.184	AAACATGGCCGG
1	MAAVATGGCBGM	PSMB4	+	151	162	4.17E-05	0.184	TAAGATGGAAGC
1	MAAVATGGCBGM	SMIM18	-	62	73	4.24E-05	0.185	CAAGATGGAGCT
1	MAAVATGGCBGM	RP11-235E17.3	-	67	78	4.24E-05	0.185	CAACATGGATGT
1	MAAVATGGCBGM	SPDYE1	+	150	161	4.24E-05	0.185	AAAGATGGCTTA
1	MAAVATGGCBGM	AC107081.5	-	61	72	4.28E-05	0.186	AAAAATGGCCCA
1	MAAVATGGCBGM	SF1	-	167	178	4.30E-05	0.186	GGAAATGGCGGC
1	MAAVATGGCBGM	SLC25A5P6	-	154	165	4.37E-05	0.188	GGAGATGGCCGC
1	MAAVATGGCBGM	RP13-638C3.2	-	10	21	4.40E-05	0.188	CAACATGGCAAA
1	MAAVATGGCBGM	GUSBP11	+	40	51	4.47E-05	0.188	AAAGATGGCGCG
1	MAAVATGGCBGM	AP000347.2	+	40	51	4.47E-05	0.188	AAAGATGGCGCG
1	MAAVATGGCBGM	CTNNB1	+	121	132	4.47E-05	0.188	AAAGATGGCGCG
1	MAAVATGGCBGM	RPL41P2	-	114	125	4.47E-05	0.188	GAAAATGGCGCT
1	MAAVATGGCBGM	TNRC6A	+	139	150	4.47E-05	0.188	GAAAATGGCGCT
1	MAAVATGGCBGM	WIZ	+	21	32	4.57E-05	0.19	CATCATGGCGGC
1	MAAVATGGCBGM	RPL31P58	+	103	114	4.57E-05	0.19	CAGAATGGCTCC
1	MAAVATGGCBGM	RP11-214N15.5	+	127	138	4.57E-05	0.19	GAAGATGGCCCT
1	MAAVATGGCBGM	RP11-58K22.4	+	177	188	4.57E-05	0.19	AAAGATGGTGGT

1	MAAVATGGCBGM	PER1	+	8	19	4.63 E-05	0.19	GACGATGGCGGC
1	MAAVATGGCBGM	RP1-292B18.1	-	75	86	4.65 E-05	0.19	GGAGATGGCTGC
1	MAAVATGGCBGM	RPL32	-	178	189	4.65 E-05	0.19	GGAGATGGCTGC
1	MAAVATGGCBGM	bP-2171C21.6	-	8	19	4.95 E-05	0.202	CAACATGGCCAG
1	MAAVATGGCBGM	CTD-2036J7.1	-	74	85	5.06 E-05	0.206	GAAGATGGTGGT
1	MAAVATGGCBGM	KMT2A	+	120	131	5.14 E-05	0.207	GAACATGGCGCA
1	MAAVATGGCBGM	MSANTD4	+	134	145	5.14 E-05	0.207	CAGAATGGCTAC
1	MAAVATGGCBGM	H2AFZP3	+	97	108	5.17 E-05	0.208	CGAAATGGCTGA
1	MAAVATGGCBGM	COX7A2L	+	103	114	5.23 E-05	0.209	CAGCATGGCGGT
1	MAAVATGGCBGM	XXbac-B476C20.13	+	142	153	5.30 E-05	0.21	CAAAATGGTAAC
1	MAAVATGGCBGM	WDR88	+	177	188	5.30 E-05	0.21	CGAGATGGCCTC
1	MAAVATGGCBGM	NSRP1P1	+	95	106	5.36 E-05	0.211	CAAGATGGTGAT
1	MAAVATGGCBGM	LINC00504	-	107	118	5.39 E-05	0.211	TAACATGGCAGA
1	MAAVATGGCBGM	NDUFA12	+	187	198	5.39 E-05	0.211	CAAGATGGAGTT
1	MAAVATGGCBGM	SNRPF	-	120	131	5.43 E-05	0.211	AGAAATGGCCGC
1	MAAVATGGCBGM	C17orf100	+	97	108	5.43 E-05	0.211	CAGAATGGCCTC
1	MAAVATGGCBGM	TPT1P10	-	92	103	5.45 E-05	0.211	CATGATGGCAGC
1	MAAVATGGCBGM	RP11-181G12.2	-	94	105	5.45 E-05	0.211	CAGCATGGCCGA
1	MAAVATGGCBGM	KDELR2	-	188	199	5.50 E-05	0.212	GAAGATGGCGAG
1	MAAVATGGCBGM	FP325331.1	-	87	98	5.82 E-05	0.223	CAGGATGGCGCA
1	MAAVATGGCBGM	RPL7A	-	166	177	5.86 E-05	0.223	GAGGATGGCAGC
1	MAAVATGGCBGM	ZXDC	+	151	162	5.86 E-05	0.223	CAAGATGGACCT
1	MAAVATGGC	LA16c-	-	113	124	5.98	0.227	CACGATGGA

	BGM	361A3.3				E-05		GGC
1	MAAVATGGC BGM	DHX9	-	76	87	6.03 E-05	0.227	CGAAATGGC TCC
1	MAAVATGGC BGM	RP11-326N17.2	-	99	110	6.03 E-05	0.227	CAAGATGGA TTA
1	MAAVATGGC BGM	SNX18P1 Y	-	73	84	6.21 E-05	0.232	CAGAATGGA TGC
1	MAAVATGGC BGM	SNX18P25	-	95	106	6.21 E-05	0.232	CAGAATGGA TGC
1	MAAVATGGC BGM	TMEM131	+	66	77	6.28 E-05	0.232	CAGTATGGC CGC
1	MAAVATGGC BGM	PARL	+	84	95	6.28 E-05	0.232	GAAGATGGC GTG
1	MAAVATGGC BGM	HMGB3P4	+	95	106	6.28 E-05	0.232	CAGGATGGC AAC
1	MAAVATGGC BGM	AC010642. 1	-	13	24	6.34 E-05	0.233	GACAATGGC GGC
1	MAAVATGGC BGM	SNORD17	-	60	71	6.34 E-05	0.233	CAACATGGA ACC
1	MAAVATGGC BGM	RP11-158H5.8	+	174	185	6.40 E-05	0.234	TAACATGGC TGG
1	MAAVATGGC BGM	RP11-351I24.3	-	95	106	6.52 E-05	0.236	GGACATGGC GGC
1	MAAVATGGC BGM	RBM8A	+	26	37	6.52 E-05	0.236	TAAGATGGA TGA
1	MAAVATGGC BGM	LINC0088 8	+	169	180	6.52 E-05	0.236	GGACATGGC GGC
1	MAAVATGGC BGM	RP11-354P11.4	-	129	140	6.65 E-05	0.24	GAAGATGGA TGT
1	MAAVATGGC BGM	RRM1	+	5	16	6.79 E-05	0.244	AAACATGGT AGC
1	MAAVATGGC BGM	AC010999. 1	-	71	82	6.90 E-05	0.246	AAAAATGGA CCC
1	MAAVATGGC BGM	RP11-378E13.3	+	96	107	6.90 E-05	0.246	AAACATGGC TCA
1	MAAVATGGC BGM	NGRN	+	51	62	6.94 E-05	0.247	CGACATGGC GGT
1	MAAVATGGC BGM	ATP5EP2	+	151	162	7.13 E-05	0.253	CAGCATGGT GGC
1	MAAVATGGC BGM	RP11-571F15.2	-	122	133	7.18 E-05	0.254	AAAAATGGC CTT
1	MAAVATGGC BGM	CCDC74B P1	+	95	106	7.23 E-05	0.255	CAGCATGGC CAC
1	MAAVATGGC BGM	RP11-16C1.1	-	176	187	7.37 E-05	0.257	AAGGATGGC GGT
1	MAAVATGGC	RP11-	-	74	85	7.45	0.257	GAAGATGGT

	BGM	91P24.1				E-05		TGT
1	MAAVATGGC BGM	SCAF4	-	91	102	7.45 E-05	0.257	CGGGATGGC GGC
1	MAAVATGGC BGM	RP11-368N21.5	-	128	139	7.45 E-05	0.257	CAACATGGA GAA
1	MAAVATGGC BGM	CTD-2561B21.8	+	52	63	7.45 E-05	0.257	CAACATGGA GAA
1	MAAVATGGC BGM	ATP5C1P1	-	95	106	7.49 E-05	0.257	GAACATGGT AGC
1	MAAVATGGC BGM	ATP5C1	-	174	185	7.49 E-05	0.257	GAACATGGT AGC
1	MAAVATGGC BGM	Z85986.1	+	70	81	7.49 E-05	0.257	CATAATGGC AGC
1	MAAVATGGC BGM	TRIB3	-	90	101	7.62 E-05	0.259	CAGGATGGA AGC
1	MAAVATGGC BGM	RP3-405J10.4	+	125	136	7.62 E-05	0.259	AAAAATGGC ACA
1	MAAVATGGC BGM	DYNLT1	+	128	139	7.62 E-05	0.259	AAAGATGGA AGA
1	MAAVATGGC BGM	DHCR7	-	75	86	7.70 E-05	0.261	AAACATGGC TAA
1	MAAVATGGC BGM	RP4-718P11.1	+	181	192	7.82 E-05	0.264	GGAGATGGC AGC
1	MAAVATGGC BGM	AC012513.4	+	97	108	7.88 E-05	0.264	TAAAATGGA GGT
1	MAAVATGGC BGM	SNORD6	+	151	162	7.88 E-05	0.264	GAAAATGGC CTT
1	MAAVATGGC BGM	G6PC2	+	189	200	7.96 E-05	0.265	CAAGATGGA TTT
1	MAAVATGGC BGM	AGER	+	106	117	8.03 E-05	0.265	AAAGATGGG GGC
1	MAAVATGGC BGM	RPL7A	+	109	120	8.03 E-05	0.265	CAAGATGGT GAG
1	MAAVATGGC BGM	RPS15	+	165	176	8.03 E-05	0.265	CAAGATGGT GAG
1	MAAVATGGC BGM	RPL7L1P2	-	42	53	8.08 E-05	0.265	GAAGATGGC ACT
1	MAAVATGGC BGM	CTC-518B2.9	-	78	89	8.08 E-05	0.265	GAGGATGGC GGT
1	MAAVATGGC BGM	RP11-15I20.1	+	124	135	8.15 E-05	0.265	CAGCATGGC CTC
1	MAAVATGGC BGM	DDX3X	-	7	18	8.31 E-05	0.265	CACAATGGC CCC
1	MAAVATGGC BGM	RPL13AP5	+	37	48	8.31 E-05	0.265	CAACATGGT GAA
1	MAAVATGGC	RP11-	-	27	38	8.31	0.265	CAACATGGT

	BGM	354E23.3				E-05		GAA
1	MAAVATGGC BGM	RPL41P2	+	55	66	8.31 E-05	0.265	CAACATGGT GAA
1	MAAVATGGC BGM	RNU1-134P	+	57	68	8.31 E-05	0.265	CAACATGGT GAA
1	MAAVATGGC BGM	RP11-530C5.2	-	113	124	8.31 E-05	0.265	CAACATGGT GAA
1	MAAVATGGC BGM	RP11-650L12.2	-	183	194	8.31 E-05	0.265	CAACATGGT GAA
1	MAAVATGGC BGM	EEF1A1P19	+	25	36	8.39 E-05	0.265	GAAAATGGC TTT
1	MAAVATGGC BGM	RP11-165N19.2	+	141	152	8.39 E-05	0.265	GAAAATGGC ACA
1	MAAVATGGC BGM	RPL29P14	+	152	163	8.39 E-05	0.265	GAAAATGGC ACA
1	MAAVATGGC BGM	RPL29P11	+	168	179	8.39 E-05	0.265	GAAAATGGC ACA
1	MAAVATGGC BGM	RP3-405J10.4	-	181	192	8.39 E-05	0.265	GAAGATGGA AGA
1	MAAVATGGC BGM	MT-ND2	-	31	42	8.58 E-05	0.269	AAAGATGGT AGA
1	MAAVATGGC BGM	RP11-660M5.1	+	112	123	8.58 E-05	0.269	AAAAATGGC AAA
1	MAAVATGGC BGM	MTND2P28	-	131	142	8.58 E-05	0.269	AAAGATGGT AGA
1	MAAVATGGC BGM	HNRNPA1P17	-	95	106	8.72 E-05	0.272	AGACATGGC TGC
1	MAAVATGGC BGM	RP11-158H5.2	-	31	42	8.77 E-05	0.273	CAACATGGC AAG
1	MAAVATGGC BGM	RP11-325L12.5	+	99	110	8.93 E-05	0.275	CAAGATGGA AAA
1	MAAVATGGC BGM	KB-1958F4.1	+	129	140	8.93 E-05	0.275	TAAAATGGA GTC
1	MAAVATGGC BGM	IKBKAP	-	144	155	8.93 E-05	0.275	AAGGATGGA GGC
1	MAAVATGGC BGM	IRF2BP2	+	128	139	9.04 E-05	0.277	GGACATGGC CGC
1	MAAVATGGC BGM	RP11-206L10.2	-	119	130	9.04 E-05	0.277	CACCATGGC GCC
1	MAAVATGGC BGM	CTD-2636A23.2	+	189	200	9.09 E-05	0.278	AAGGATGGC CCC
1	MAAVATGGC BGM	CTB-58E17.9	-	109	120	9.34 E-05	0.284	TAGGATGGC GCC
1	MAAVATGGC BGM	H3F3C	+	172	183	9.34 E-05	0.284	GAAAATGGC CCG
1	MAAVATGGC	HSPA8P5	-	95	106	9.40	0.285	CGACATGGT

	BGM					E-05		GGC
1	MAAVATGGC BGM	RN7SL3	+	87	98	9.47 E-05	0.286	CAATATGGT GAC
1	MAAVATGGC BGM	LYRM2	+	134	145	9.56 E-05	0.288	GGACATGGC TGC
1	MAAVATGGC BGM	SNORD39	-	117	128	9.74 E-05	0.292	CAGCATGGC AGA
1	MAAVATGGC BGM	PAPPA2	+	76	87	9.94 E-05	0.297	AAAGATGGA AAC
